# Publisher Correction: A multistage rotational speed changing molecular rotor regulated by pH and metal cations

**DOI:** 10.1038/s41467-018-04902-5

**Published:** 2018-06-13

**Authors:** Yingying Wu, Guangxia Wang, Qiaolian Li, Junfeng Xiang, Hua Jiang, Ying Wang

**Affiliations:** 1 College of Chemistry, Beijing Normal University, 100875 Beijing, China; 2 Institute of Chemistry, Chinese Academy of Sciences, 100190 Beijing, China

Correction to: *Nature Communications* 10.1038/s41467-018-04323-4, published online 16 May 2018

The original version of this article omitted the Received and Accepted dates; they should have been 22nd January 2018 and 19th April 2018, respectively. This has been corrected in the PDF and HTML versions of the Article.

