# Supplementary material for: A multistage rotational speed changing molecular rotor regulated by pH and metal cations
Source: Nat Commun. 2018 May 16;9:1953. doi: 10.1038/s41467-018-04323-4 (PMC5955901; doi:10.1038/s41467-018-04323-4)
Supplement: Supplementary file 1 — Supplementary Information [file 41467_2018_4323_MOESM1_ESM.pdf]

Supplementary Information

A Multistage Rotational Speed Changing Molecular Rotor Regulated by pH and Metal Cations

Wu et al.

Supplementary Note 1. The solvent model used in the computational calculations is not based on explicit solvent molecules that can form hydrogen bonds with solute molecules but instead model the solvent as a polarizable continuum.

Supplementary Note 2. In the presence of one equivalent of DBU, about 0.89%, 14 ppm and 0.28 ppm of **1**, **2** and **3**, respectively, is calculated to be still remained in the system, assuming the p*K*a of **1** – **3** is identical to their analog phenol, 4-nitrophenol and 2-chloro-4-nitrophenol, respectively (see the main text and reference 50)

Supplementary Note 3. A samll or large amount of protons released in this process would give a proton-catalyzed fast rotation of the rotor thus giving rise to no obvious decoalescences of the methyl protons in the corrresponding ^{1}H NMR spectrum. See the description about the properties of DBU-deprotonated **1** – **3** described in the main text and Supplementary Note 2.

Supplementary Note 4. It is notable that the rotor in this studied case exists as a mixture of two different species (the one at the native state and the one with metal cation complexed) in fast dynamic equilibrium. The apparent rotational barrier is thus an averaged one, weighted by the fraction ration of these two species and the catalytic efficiency of the cations. Therefore, the observed decrease of the measured rotational barrier upon the addition of metal cations does not mean that the rotational speed could be tuned by changing the concentration of the cations.

Supplementary Note 5. To reduce the accumulation of the stimuli in the system, initially only 1.0 equivalent of Li^{+} was added.

Supplementary Method 1. General Methods

Throughout the main text and the Supplementary Information, a series alphabets have been used to schematically illustrate the labelling of the protons of the studied molecules (see Fig. 1a and Supplementary Fig. 19), except that, in Section relating to the assignment of NMR signals (Supplementary Tables 2 – 4 and Supplementary Figures 7 – 18), an independent numeral system was used.

Anhydrous tetrahydrofuran (THF) was distilled over sodium benzophenone under inert atmosphere. Compounds **1**–**3** were prepared using procedures reported in the literature.[1] All starting chemicals and deuterated solvents were obtained from commercial sources and used without further purification. Column chromatography was carried out on flash grade silica gel, using 0 – 20 psig pressure.

NMR spectra were obtained with a Bruker spectrometer (^{1}H, 400, 500, and 600 MHz) or a JEOL spectrometer (^{1}H, 400 and 600 MHz) using acetone-d_6 (CD_3COCD_3), acetonitrile-d_3 (CD_3CN), chloroform-d ($CDCl_3$) as solvent. The chemical shift references were as follows: (^{1}H) acetone-d_5, 2.05 ppm; (^{13}C) acetone-d_6, 29.84 ppm; (^{1}H) acetonitrile-d_2, 1.94 ppm; (^{13}C) acetonitrile-d_3, 1.32 ppm; (^{1}H) chloroform-*h*, 7.26 ppm; (^{13}C) chloroform-d, 77.16 ppm. VT ^{1}H NMR Investigations were carried out using acetone-d_6 or acetonitrile-d_3 as the solvent. In a typical measurement, a 4 mM solution of a rotor in stated deuterated solvents was contained in 5-mm NMR sample tubes. When elevated temperatures are involved in the investigation, a J. Young tube was used. In the measurements, a serial of ^{1}H NMR spectra of the sample at various temperature were recorded. Each ^{1}H NMR spectrum was recorded after the sample was maintained at the stated temperature for at least 10 minutes. Typically, more than 7 spectra were recorded at each run. The probe temperatures for the VT NMR measurements (VT ^{1}H NMR and 2D EXSY) were calibrated with neat ethylene glycol (for temperature in range of 313−393 K) and neat methanol (for temperature in range of 203−313 K) according to the literature,[2] by observation of the chemical-shift separation between the OH resonances and CH_2 (or CH_3) resonances in ethylene glycol or methanol. High resolution mass (HR-MS-ESI) spectra were acquired on FT-ICR spectrometer. IR spectra were recorded on FT-IR spectrometer with thin KBr disk.

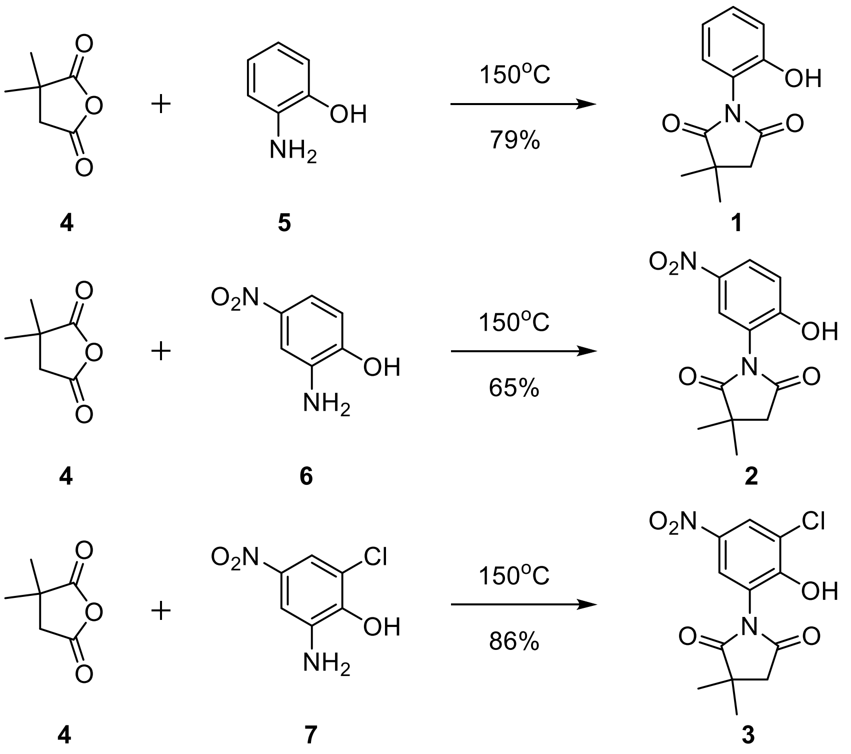

Supplementary Figure 1. Synthetic scheme for compound **1** – **3**.

Supplementary Method 2. General Methodology for the Synthesis of Compounds **1** – **3.**

The compounds were at first prepared according to a known procedure[1]: 2,2-dimethylsuccinic anhydride **4** (1.18 mmol) and the corresponding *o*-hydroxyanilines (compound **5** or **6** or **7**) (1.30 mmol) were dissolved in tetrahydrofuran (THF, 5 mL). The solvent was removed in vacuo and the residue was heated at 150 °C for overnight under N_2 atmosphere. Purification of the black solid mixture provides the corresponding products **1** – **3**.

We also tried to avoid the use of the solvent THF for mixing the reactants. In such cases, the succinic anhydride and the *o*-hydroxyaniline were directly put into a round-bottom flask equipped with a condenser, heated at 150 °C for overnight under N_2 atmosphere. Then the resultant mixtures were purified. This procedure provides the products with very similar yields as those from the known procedure.

Compound 1. The product was purified by silica gel column chromatography (hexane/EtOAc = 20:1) to give **1** as a white solid in 86% (206 mg) yield. M.p. 135–138 °C (under air). TLC (hexane: ethyl acetate, 2:1 (v/v)): R_f = 0.27. ^{1}H NMR (400 MHz, $CDCl_3$, 298 K): δ 7.29-7.31 (m, 1H), 7.20-

7.22 (m, 1H), 7.03-7.07 (m, 2H), 5.77 (s, 1H), 2.78 (s, 2H), 1.46 (s, 6H). ^{13}C NMR (100 MHz, $CDCl_3$, 298 K): δ 183.2, 175.9, 151.4, 130.6, 128.3, 121.2, 120.3, 118.7, 43.9, 40.8, 25.8. IR (KBr): 3284, 2969, 2930, 2873, 1776, 1710, 1688, 1596, 1604, 1464, 1411, 1375, 1302, 1242, 1215, 1511, 1020, 903, 853, 761, 682 cm^{-1}. HRMS (ESI-FT-ICR) *m/z*: [M – H]$^-$ calcd for $C_{12}H_{12}NO_3$ 218.0817, found 218.0818 (–0.1 ppm).

Compound 2. The product was purified by silica gel column chromatography (hexane/EtOAc = 5:1) to give **2** as a gray solid in 65% (226 mg) yield. M.p. 177–179 °C (under air). TLC (hexane: ethyl acetate, 1:1 (v/v)): R_f = 0.40. ^{1}H NMR (400 MHz, $CDCl_3$, 298 K): δ 8.20-8.22 (m, 2H), 7.13 (d, J = 9.6 Hz, 1H), 6.94 (s, 1H), 2.84 (s, 2H), 1.49 (s, 6H). ^{13}C NMR (100 MHz, $CDCl_3$, 298 K): δ 182.8, 175.3, 157.1, 141.5, 126.3, 125.1, 120.4, 118.8, 43.9, 41.1, 25.8. IR (KBr): 3236, 3099, 2975, 2937, 2877, 1777, 1695, 1600, 1533, 1504, 1410, 1341, 1301, 1220, 1158, 1081, 904, 836, 708, 629 cm^{-1}. HRMS (ESI-FT-ICR) *m/z*: [M – H]$^-$ calcd for $C_{12}H_{11}N_2O_5$ 263.0668, found 263.0673 (–0.5 ppm).

Compound 3. The product was purified by silica gel column chromatography (hexane/EtOAc = 2:1) to give **3** as a brown solid in 86% yield, which was further recrystallized from ethyl acetate and hexane to give a white crystaline in yield of 79% (283 mg). M.p. > 300 °C (under air). TLC (ethyl acetate: CH_3OH, 30:1 (v/v)): R_f = 0.49. ^{1}H NMR (400 MHz, $CDCl_3$, 298 K): δ 8.38 (d, J = 2.4 Hz, 1H), 7.08 (d, J = 2.4 Hz, 1H), 6.60 (s, 1H), 2.81 (s, 2H), 1.48 (s, 6H). ^{13}C NMR (100 MHz, $CDCl_3$, 298 K): δ 181.5, 174.0, 153.1, 141.1, 125.9, 124.6, 122.4, 120.8, 44.1, 41.2, 25.8. IR (KBr): 3090, 2982, 2932, 2872, 1778, 1715, 1702, 1606, 1592, 1532, 1491, 1463, 1444, 1395, 1378, 1348, 1326, 1212, 1137, 1076, 1024, 987, 948, 903, 856, 767, 741, 624 cm^{-1}. HRMS (ESI-FT-ICR) *m/z*: [M – H]$^-$ calcd for $C_{12}H_{10}ClN_2O_5$ 297.0277, found 297.0284 (–0.7 ppm).

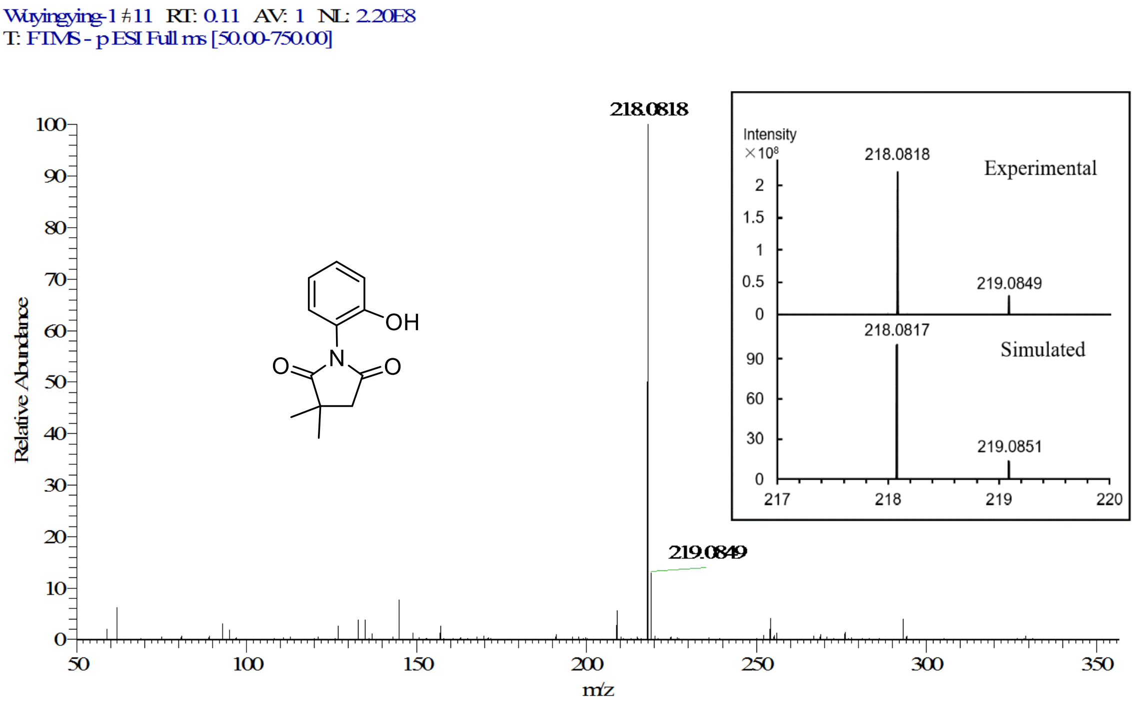

Supplementary Figure 2. HRMS-ESI spectrum of molecular **1**. Inset: experimental and simulated spectra that show the expansion of the isotopic cluster for [M–H]$^{-}$ ions (formula: $C_{12}H_{12}NO_3$). High resolution mass spectrometry with electrospray ionization (HRMS-ESI) were obtained in the negtive ion mode using acetonitrile as the mobile flow phase.

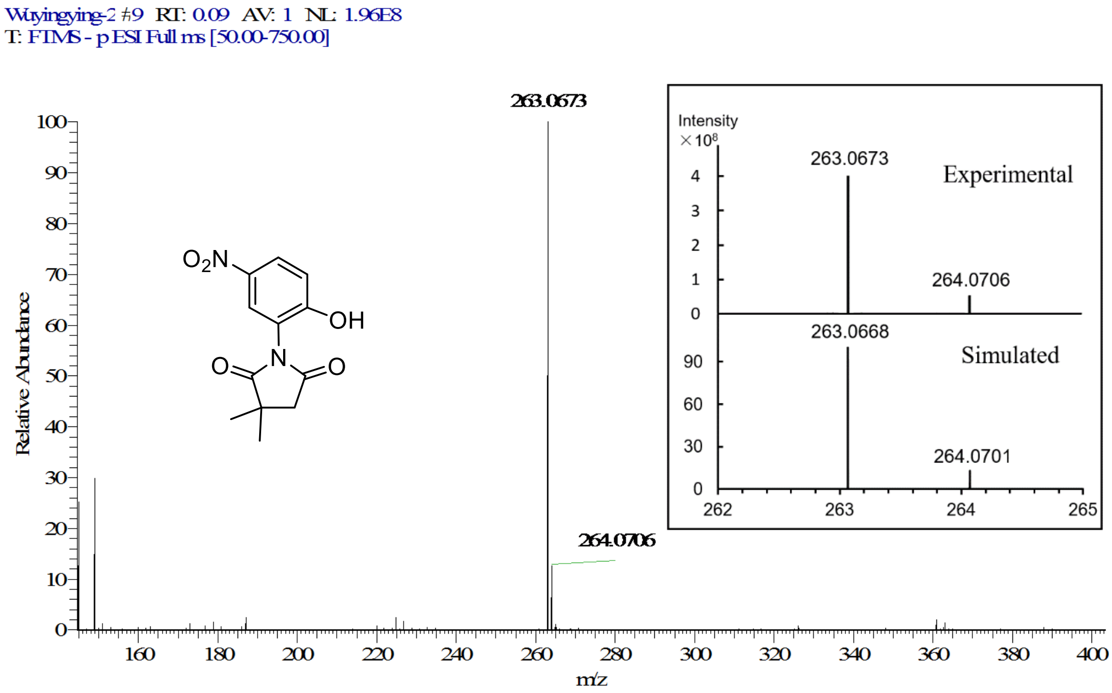

Supplementary Figure 3. HRMS-ESI spectrum of molecular **2**. Inset: experimental and simulated spectra that show the expansion of the isotopic cluster for [M–H]$^{-}$ ions (formula: $C_{12}H_{11}N_2O_5$). High resolution mass spectrometry with electrospray ionization (HRMS-ESI) were obtained in the negtive ion mode using acetonitrile as the mobile flow phase.

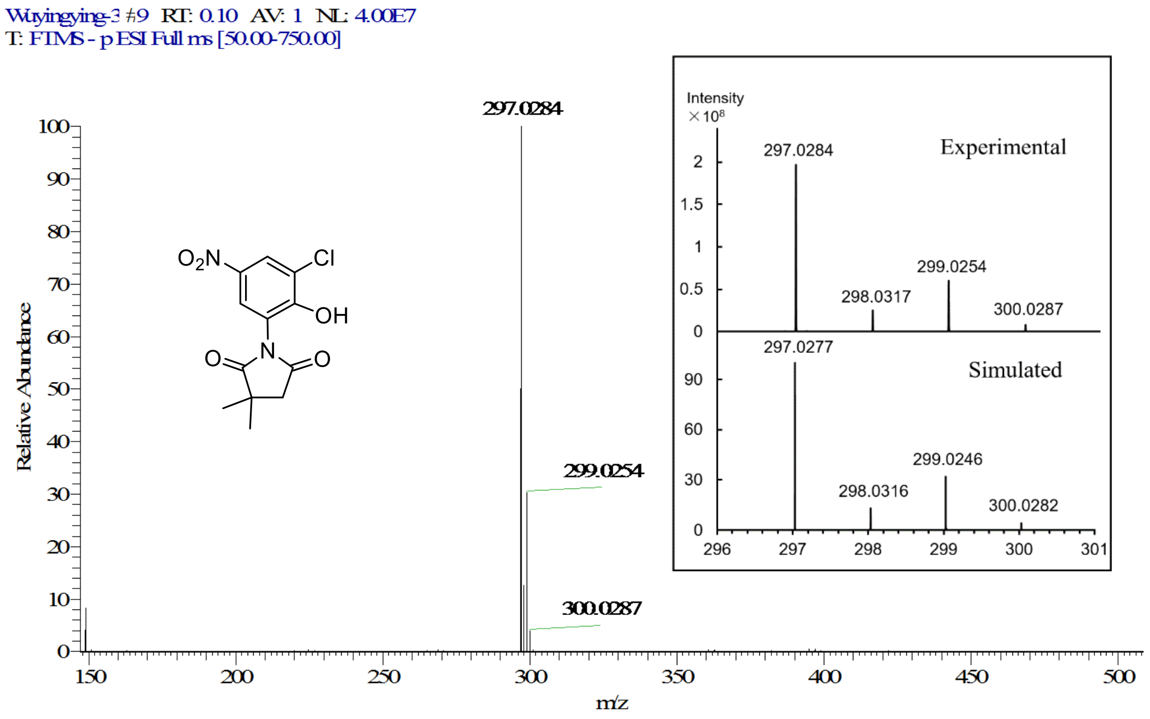

Supplementary Figure 4. HRMS-ESI spectrum of molecular **3**. Inset: experimental and simulated spectra that show the expansion of the isotopic cluster for $[M–H]^-$ ions (formula: $C_{12}H_{10}ClN_2O_5$). High resolution mass spectrometry with electrospray ionization (HRMS-ESI) were obtained in the negtive ion mode using acetonitrile as the mobile flow phase.

Supplementary Method 3. X-ray Crystallography of **1 – 3.**

Crystal structures of **1**, **2** and **3** were all obtained by slowly evaporating a solvent mixture of 1:1 (v/v) hexane and ethyl acetate at ambient temperature.

X-ray single crystal diffraction for compounds **1–3** was performed on Saturn724 CCD X-ray diffractometer equipped with Mo Kα radiation (λ = 0.71073 Å) source. The crystal was kept at 173.15(10) K during data collection. Their structures were solved by direct methods using SHELXTL and refined with full-matrix least-squares calculations on F^2 using SHELXL-97.[3] All non-hydrogen atoms were refined anisotropically. All hydrogen atoms were positioned by geometric idealization. The space groups were determined based on intensity statistics and the lack of systematic absences. Additional crystal and refinement information is summarized in Supplementary Table 1.

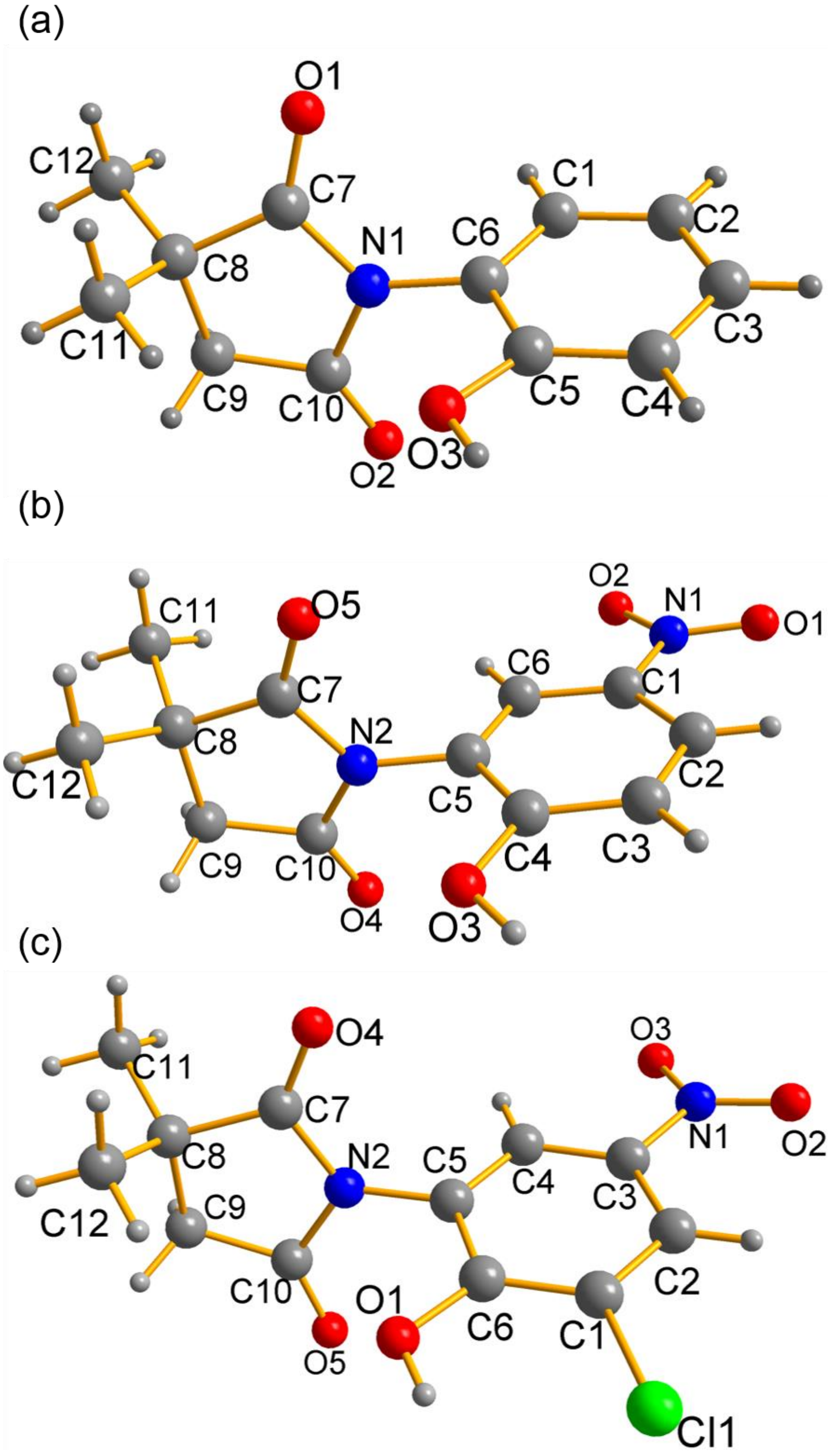

Supplementary Figure 5. Diamond 3.0-generated ball and stick diagrams for molecular structure of (a) **1**, (b) **2** and (c) **3** which crystallized from the mixture of hexane and ethyl acetate.

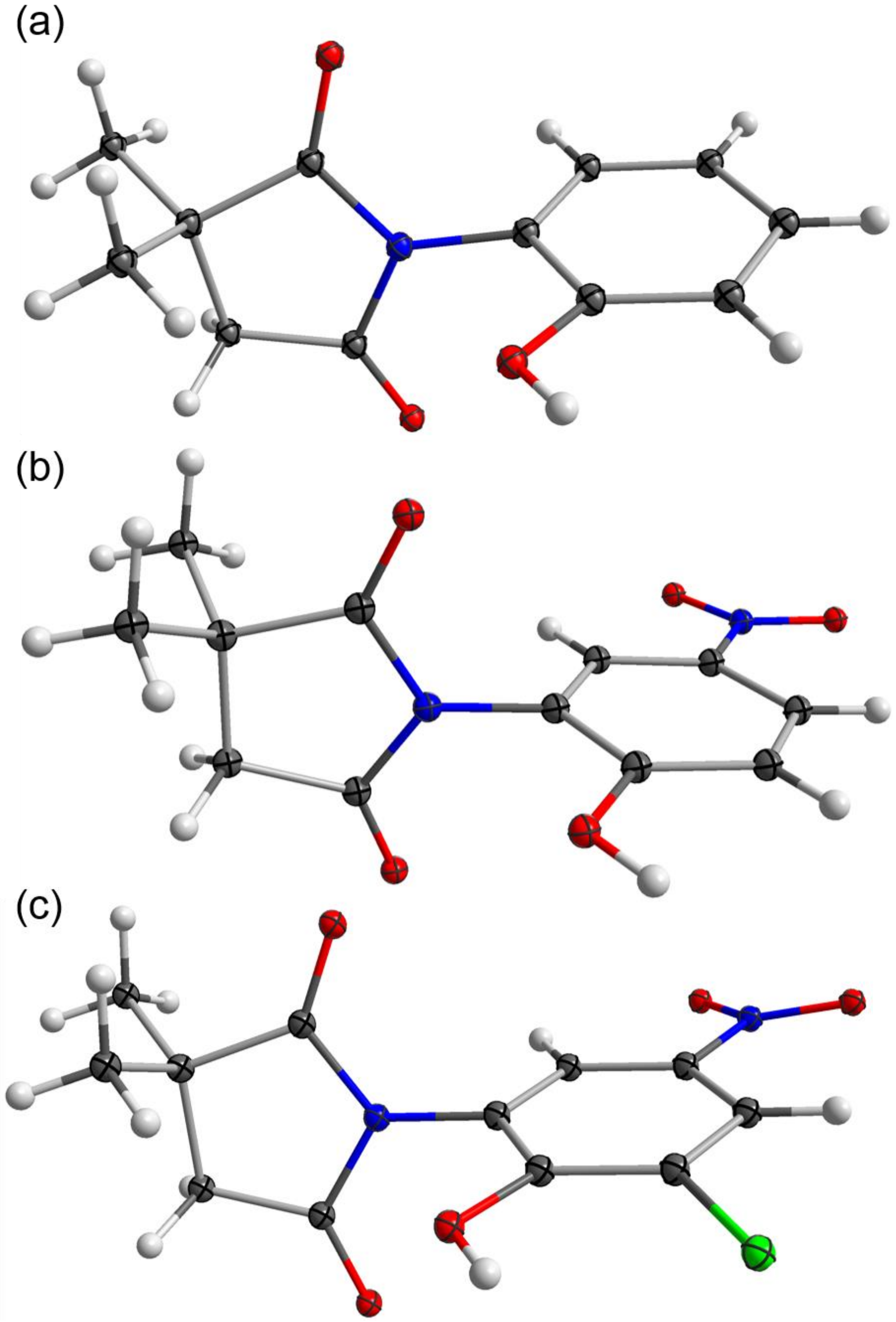

Supplementary Figure 6. The ellipsoid plots for molecular structure of (a) **1**, (b) **2** and (c) **3** which crystallized from the mixture of hexane and ethyl acetate. The thermal ellipsoids are set at a 50% probability level.

Supplementary Table 1. Crystal Data and Structure Refinement of Compounds **1 – 3**.

Compd.	**1**	**2**	**3**
Formula	$C_{12}H_{13}NO_3$	$C_{12}H_{12}N_2O_5$	$C_{12}H_{11}ClN_2O_5$
fw	219.23	264.24	298.68
Temp (K)	173.15	173.15	173.15
Wavelength (Å)	0.71073	0.71073	0.71073
crystal system	Orthorhombic	Monoclinic	monoclinic
space group	P2(1)2(1)2(1)	C(1)2/c(1)	P(1)21/c(1)
a (Å)	6.7648(14)	11.039(5)	7.4961(15)
b (Å)	15.467(3)	20.066(8)	18.219(4)
c (Å)	32.578(6)	12.073(6)	9.7283(19)
α (deg)	90	90	90
β (deg)	90	113.576(6)	96.59(3)
γ (deg)	90	90	90
V (Å^3)	3408.7(12)	2451.2(18)	1319.8(5)
Z	12	8	4
D_c (mg/m^3)	1.282	1.432	1.503
μ (mm^{-1})	0.093	0.113	0.311
F (000)	1392	1105	616
crystal size (mm)	0.582× 0.232×0.128	0.25× 0.21×0.17	0.53 x 0.29 x 0.27
reflns collected	23643	11733	8303
unique reflns (R_{int})	7643(0.0674)	2821(0.0461)	2984(0.0249)
θ range (deg)	1.457-27.439	2.254-27.526	3.073-27.486
data/restraints/params	7643/14/506	2821/0/178	2984/0/184
final R indices	0.0805/0.1885	0.0600/0.1437	0.0384/0.0886
R indices (all data)	0.0910/0.1975	0.0640/0.1465	0.0403/0.0897
GOF on F^2	1.148	1.195	1.134
ρ_{max}/ρ_{min}(e·Å^{-3})	0.505/-0.357	0.226/-0.237	0.263/-0.294

Supplementary Table 2. ^{1}H and ^{13}C NMR spectral assignments for **1** in acetonitrile-d_3: Calculated NMR isotropic shieldings (σ_{DFT}),[a] as well as the experimental (δ_{expt}), calculated (δ_{DFT})[b] and scaled (δ_{scaled})[c] NMR chemical shifts.

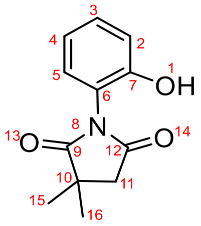

No.	[1]H					[13]C				
	σ_{DFT}	δ_{expt}	δ_{calc}	δ_{scaled}	δ_{scaled}-δ_{expt}	σ_{DFT}	δ_{expt}	δ_{calc}	δ_{scaled}	δ_{scaled}-δ_{expt}
1	25.576	7.270	6.055	/	/	/	/	/	/	/
2	24.275	6.992	7.356	6.928	-0.064	80.03	117.30	113.06	113.64	-3.66
3	23.793	7.306	7.839	7.375	0.069	63.95	131.34	129.14	130.09	-1.24
4	24.1922	6.972	7.439	7.005	0.033	75.24	121.10	117.85	118.54	-2.56
5	24.131	7.104	7.501	7.062	-0.042	64.53	130.41	128.56	129.50	-0.91
6	/	/	/	/	/	74.82	121.15	118.27	118.97	-2.18
7	/	/	/	/	/	41.81	153.53	151.28	152.74	-0.79
9	/	/	/	/	/	8.41	183.58	184.68	186.89	3.32
10	/	/	/	/	/	146.66	41.25	46.43	45.49	4.24
11	28.836	2.711	2.795	2.708	-0.003	147.46	44.07	45.63	44.68	0.61
12	/	/	/	/	/	15.70	176.03	177.39	179.44	3.41
15	30.276	1.366	1.355	1.376	0.010	166.38	25.59	26.71	25.33	-0.27
16	30.292	1.366	1.340	1.361	-0.005	166.11	25.59	26.98	25.61	0.01

[a] Calculated at the B3LYP/6-31G(d,p)/IEF-PCM-UFF level of theory using gauge invariant atomic, based on the calculated global minimum geometries (perpendicular conformation) (Supplementary Table 25) of the compounds in acetonitrile-d_3. [b] σ_{DFT} for TMS: 31.6316 (^{1}H, in acetonitrile), 193.090 (^{13}C, in acetonitrile). [c] δ_{scaled} = (δ_{DFT} – intercept)/slope. The intercept and the slope are from correlations between δ_{DFT} and δ_{expt}.

Supplementary Table 3. ^{1}H and ^{13}C NMR spectral assignments for **2** in acetonitrile-d_3: Calculated NMR isotropic shieldings (σ_{DFT}),[a] as well as the experimental (δ_{expt}), calculated (δ_{DFT})[b] and scaled (δ_{scaled})[c] NMR chemical shifts.

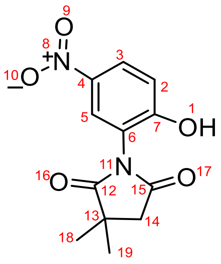

No.	^{1}H					^{13}C				
	σ_{DFT}	δ_{expt}	δ_{calc}	δ_{scaled}	δ_{scaled}-δ_{expt}	σ_{DFT}	δ_{expt}	δ_{calc}	δ_{scaled}	δ_{scaled}-δ_{expt}
1	24.476	8.655	8.013	7.559	-1.069	/	/	/	/	/
2	24.172	7.138	7.459	7.044	-0.094	78.77	117.39	114.32	113.99	-3.40
3	22.800	8.193	8.832	8.321	0.128	65.45	127.29	127.65	127.56	0.27
4	/	/	/	/	/	53.45	141.65	139.64	139.77	-1.88
5	23.084	8.110	8.547	8.057	-0.053	65.49	126.86	127.60	127.52	0.66
6	/	/	/	/	/	73.59	121.19	119.50	119.27	-1.92
7	/	/	/	/	/	34.51	159.53	158.58	159.05	-0.48
12	/	/	/	/	/	8.99	182.99	184.10	185.04	2.05
13	/	/	/	/	/	146.15	41.47	46.94	45.40	3.93
14	28.771	2.748	2.861	2.763	0.015	147.50	44.07	45.59	44.03	-0.04
15	/	/	/	/	/	16.38	175.52	176.71	177.51	1.99
18	30.256	1.381	1.376	1.381	0.000	166.24	25.54	26.85	24.95	-0.58
19	30.253	1.381	1.379	1.384	0.003	166.26	25.54	26.83	24.93	-0.61

[a] Calculated at the B3LYP/6-31G(d,p)/IEF-PCM-UFF level of theory using gauge invariant atomic, based on the calculated global minimum geometries (perpendicular conformation) (Supplementary Table 25) of the compounds in acetonitrile-d_3. [b] σ_{DFT} for TMS: 31.6316 (^{1}H, in acetonitrile), 193.090 (^{13}C, in acetonitrile). [c] δ_{scaled} = (δ_{DFT} – intercept)/slope. The intercept and the slope are from correlations between δ_{DFT} and δ_{expt}.

Supplementary Table 4. ^{1}H and ^{13}C NMR spectral assignments for **3** in acetonitrile-d_3: Calculated NMR isotropic shieldings (σ_{DFT}),[a] as well as the experimental (δ_{expt}), calculated (δ_{DFT})[b] and scaled (δ_{scaled})[c] NMR chemical shifts.

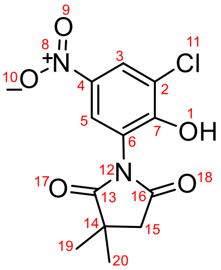

No.	^{1}H					^{13}C				
	σ_{DFT}	δ_{expt}	δ_{calc}	δ_{scaled}	δ_{scaled}-δ_{expt}	σ_{DFT}	δ_{expt}	δ_{calc}	δ_{scaled}	δ_{scaled}-δ_{expt}
1	24.434	8.290	7.198	6.815	-1.475	/	/	/	/	/
2	/	/	/	/	/	65.21	122.90	127.88	127.30	4.40
3	22.690	8.370	8.942	8.441	0.071	66.60	127.10	126.49	125.89	-1.21
4	/	/	/	/	/	54.36	141.36	138.73	138.33	-3.02
5	23.154	8.085	8.478	8.008	-0.077	66.78	125.37	126.31	125.70	0.33
6	/	/	/	/	/	72.97	122.29	120.12	119.40	-2.89
7	/	/	/	/	/	38.96	155.54	154.13	153.99	-1.55
13	/	/	/	/	/	9.27	182.71	183.82	184.19	1.47
14	/	/	/	/	/	145.98	41.62	47.11	45.16	3.54
15	28.750	2.780	2.882	2.793	0.013	147.52	44.08	45.57	43.60	-0.47
16	/	/	/	/	/	16.70	175.22	176.39	176.63	1.41
19	30.253	1.401	1.379	1.393	-0.008	166.06	25.56	27.03	24.74	-0.82
20	30.244	1.401	1.388	1.401	0.000	166.45	25.56	26.64	24.35	-1.21

[a] Calculated at the B3LYP/6-31G(d,p)/IEF-PCM-UFF level of theory using gauge invariant atomic, based on the calculated global minimum geometries (perpendicular conformation) (Supplementary Table 25) of the compounds in acetonitrile-d_3. [b] σ_{DFT} for TMS: 31.6316 (^{1}H, in acetonitrile), 193.090 (^{13}C, in acetonitrile). [c] δ_{scaled} = (δ_{DFT} – intercept)/slope. The intercept and the slope are from correlations between δ_{DFT} and δ_{expt}.

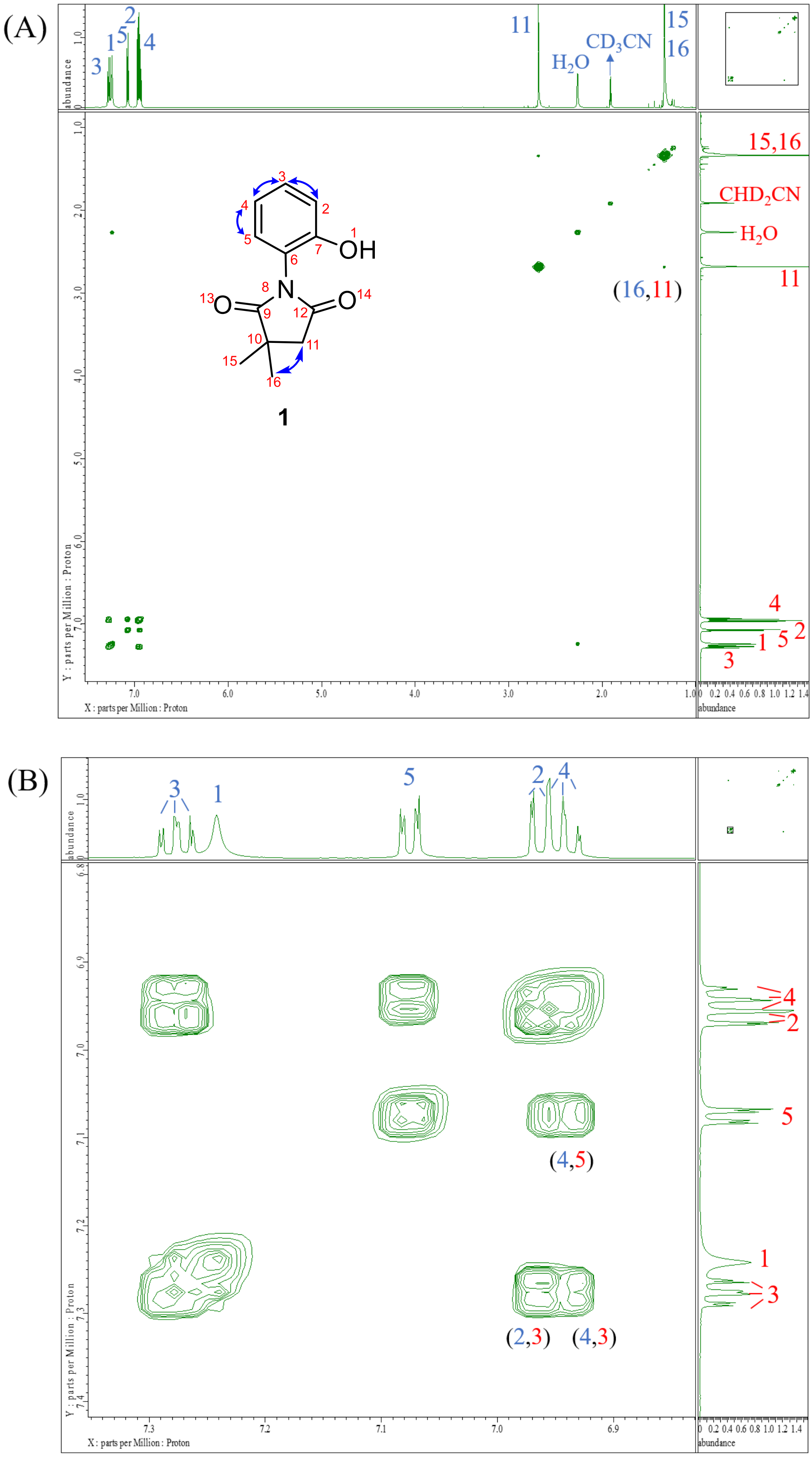

Supplementary Figure 7. (A) ^{1}H–^{1}H COSY NMR (600 MHz, CD_3CN) spectrum of **1** at 298 K. (B) Zoom of the spectrum A.

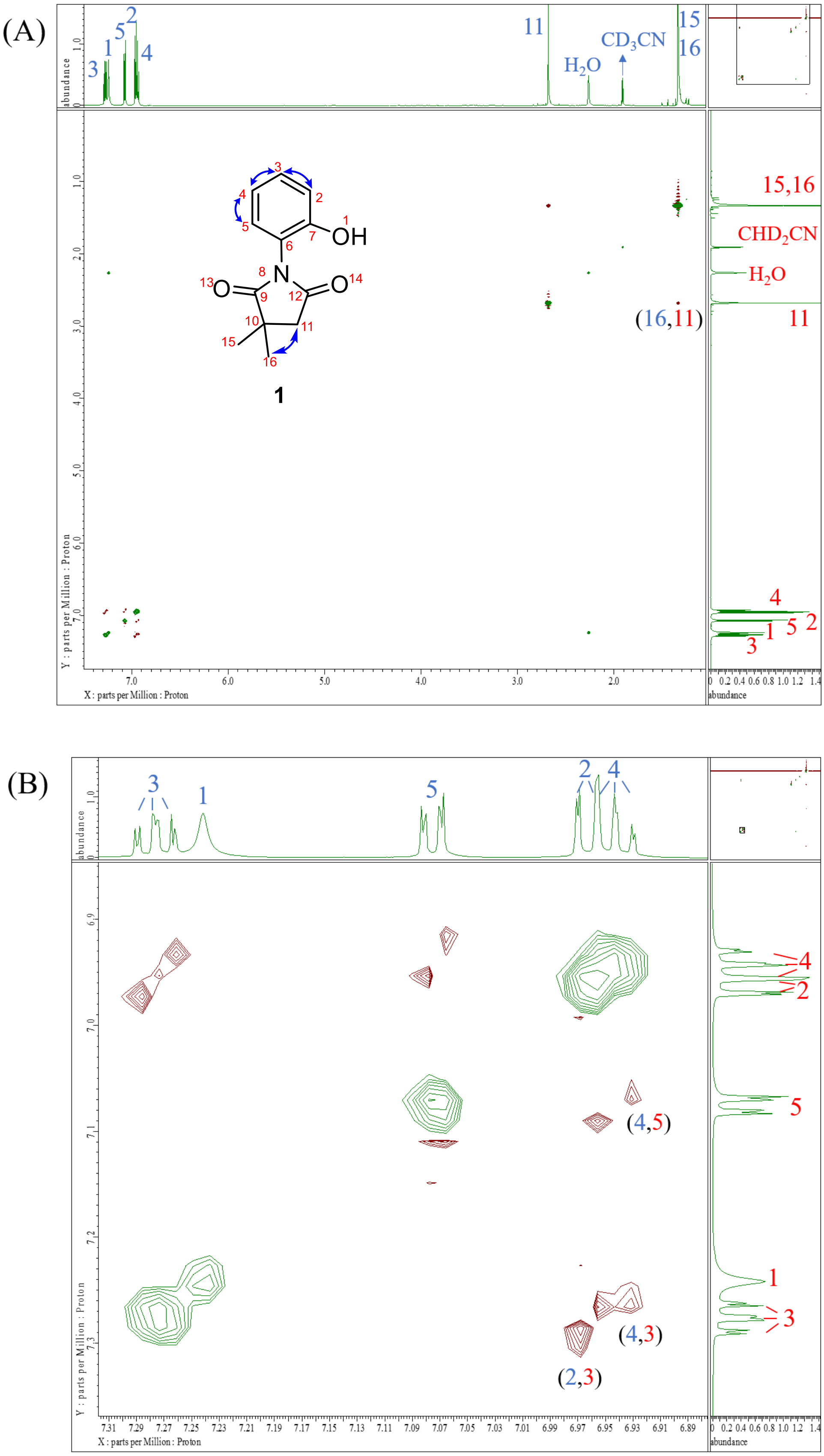

Supplementary Figure 8. (A) ^{1}H–^{1}H NOESY NMR (600 MHz, CD_3CN) spectrum of **1** at 298 K. (B) Zoom of the spectrum A.

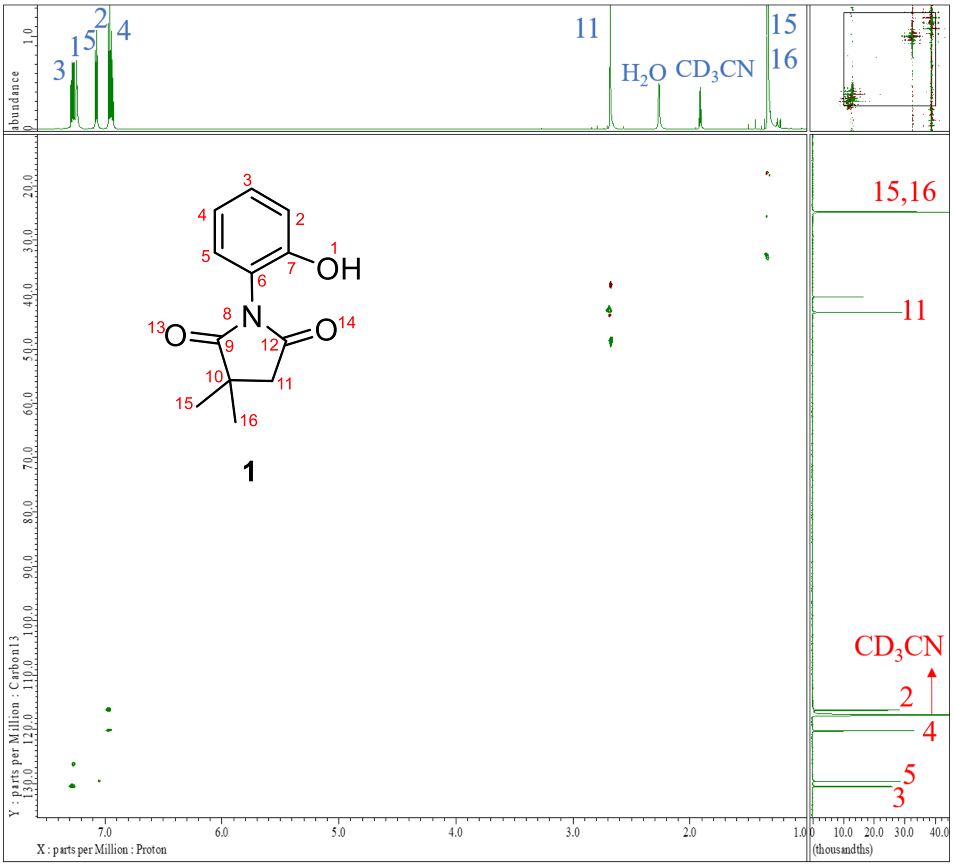

Supplementary Figure 9. ^{1}H–^{13}C HSQC NMR (600 MHz, CD_3CN) spectrum of **1** at 298 K.

(A)

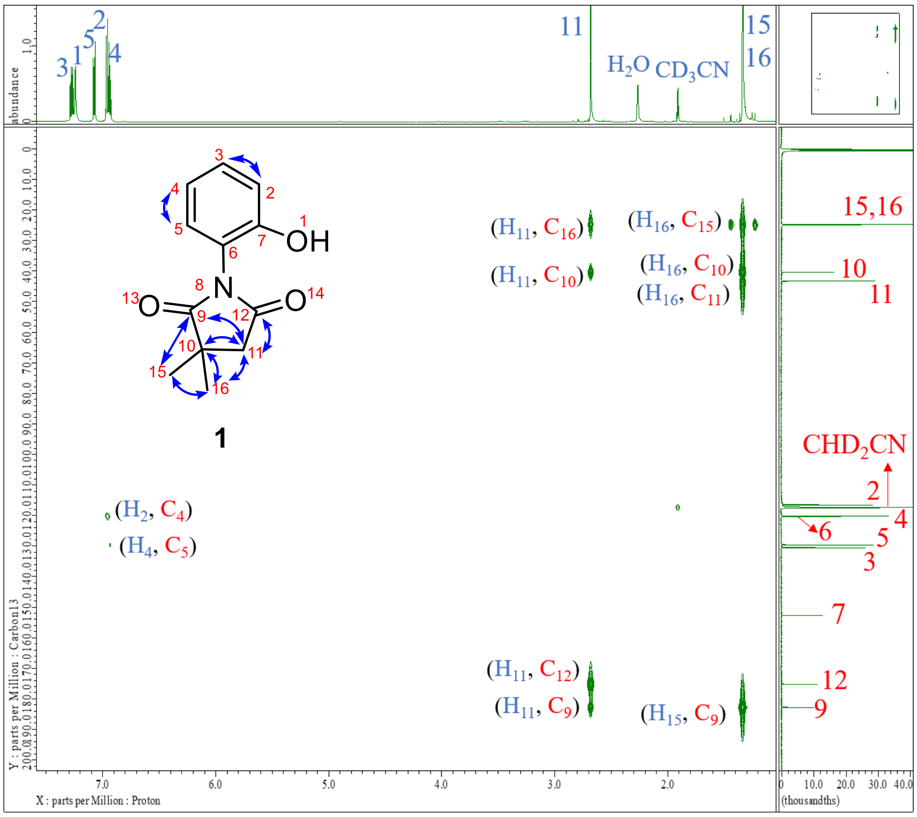

(to be continued)

(B)

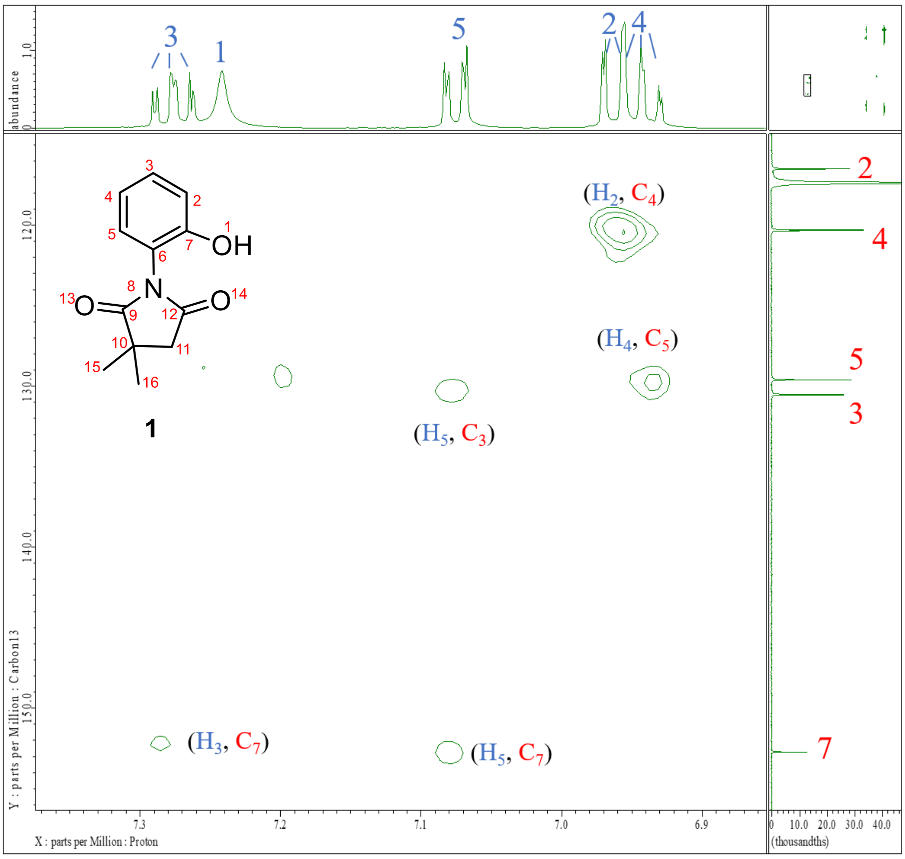

Supplementary Figure 10. (A) ^{1}H–^{13}C HMBC NMR (600 MHz, CD_3CN) spectrum of **1** at 298 K. (B) Zoom of the spectrum A.

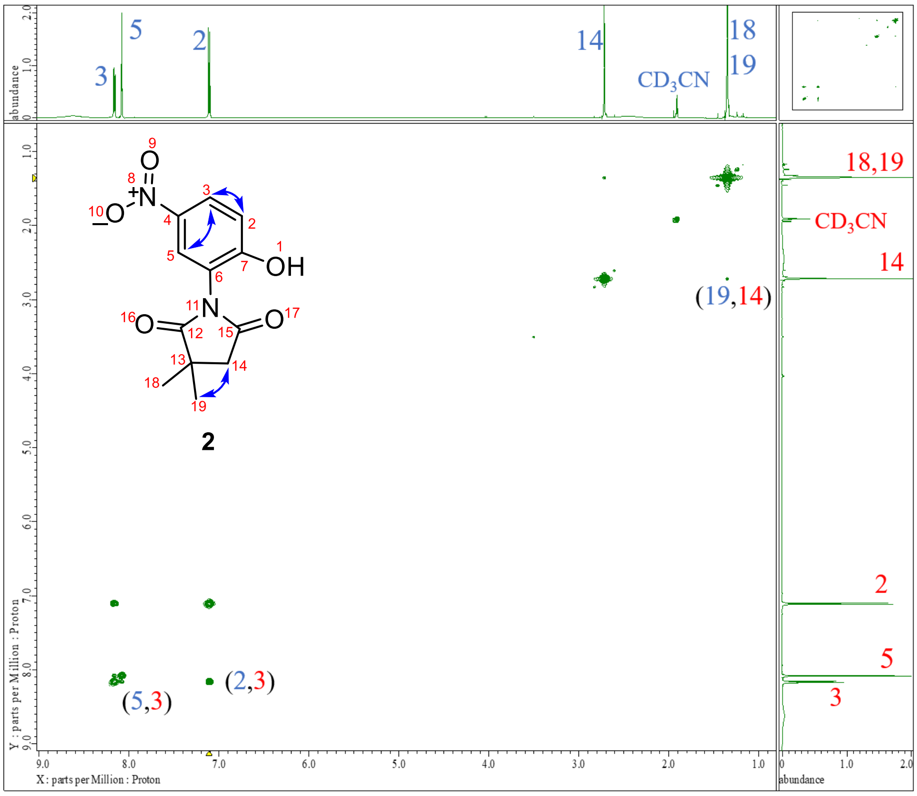

Supplementary Figure 11. ^{1}H–^{1}H COSY NMR (600 MHz, CD_3CN) spectrum of **2** at 298 K.

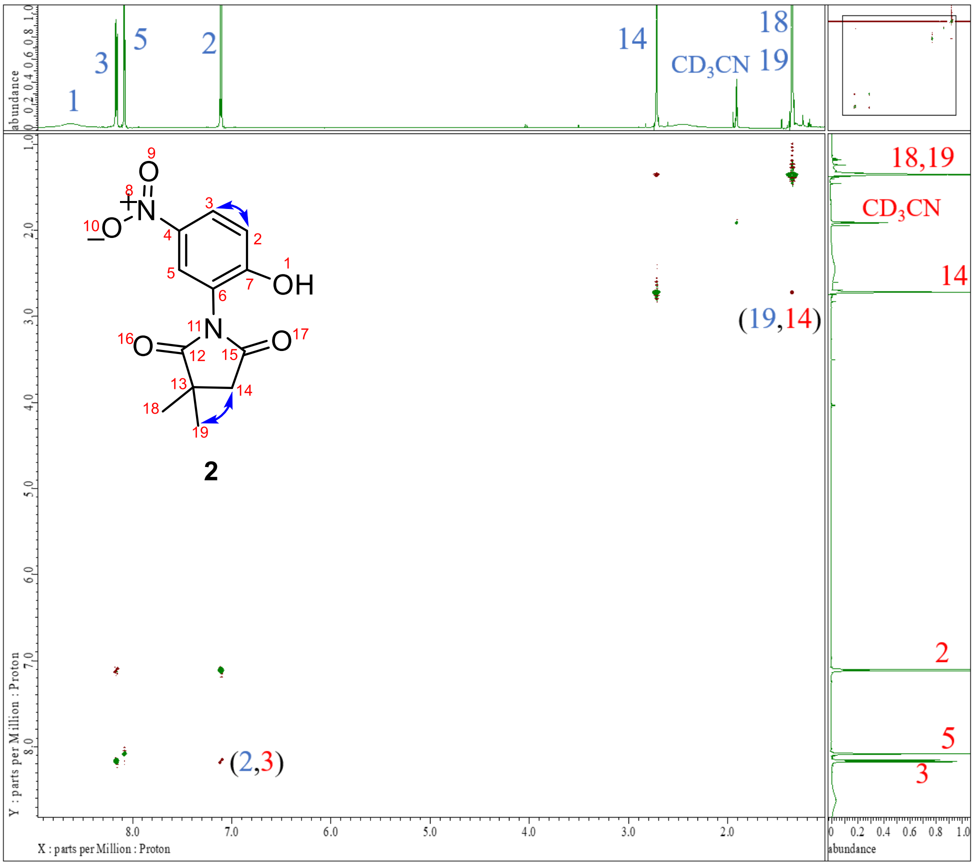

Supplementary Figure 12. ^{1}H–^{1}H NOESY NMR (600 MHz, CD3CN) spectrum of **2** at 298 K.

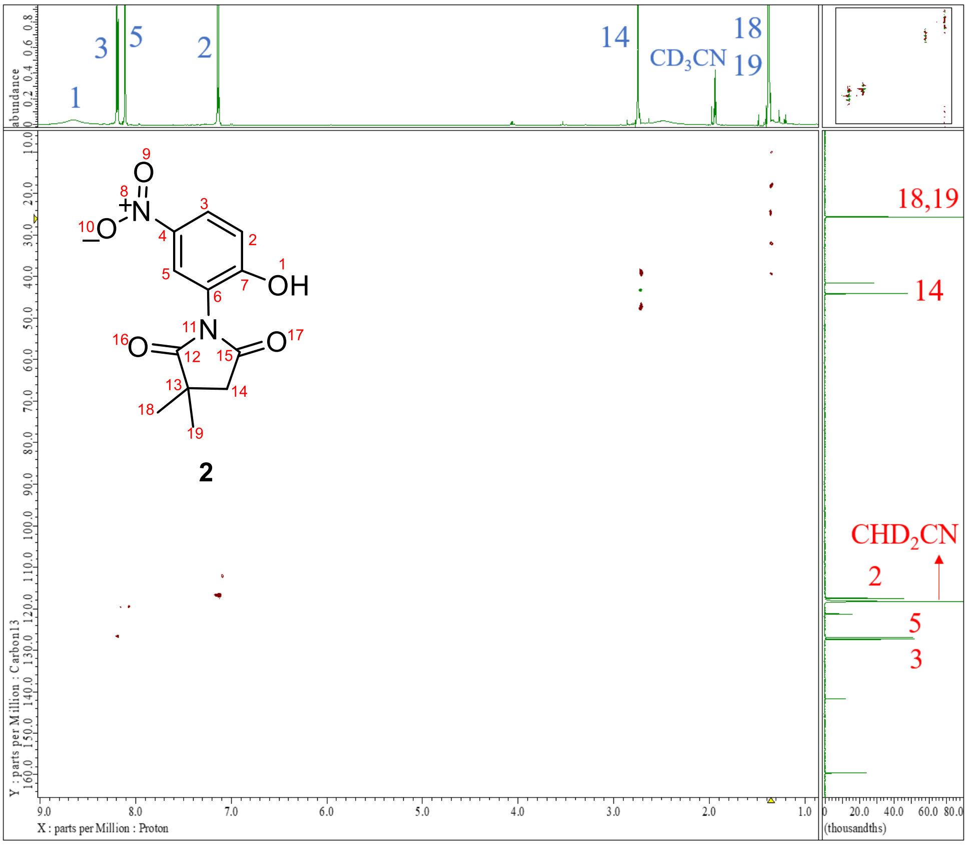

Supplementary Figure 13. ^{1}H–^{13}C HSQC NMR (600 MHz, CD3CN) spectrum of **2** at 298 K.

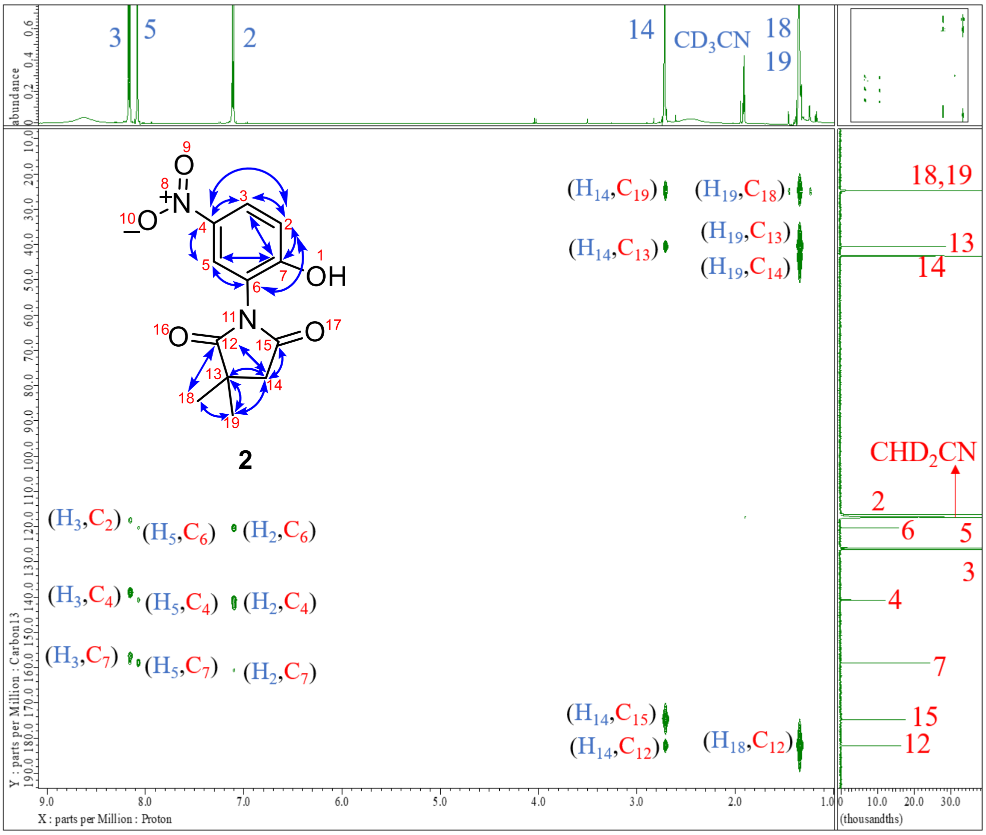

Supplementary Figure 14. ^{1}H–^{13}C HMBC NMR (600 MHz, CD_3CN) spectrum of **2** at 298 K.

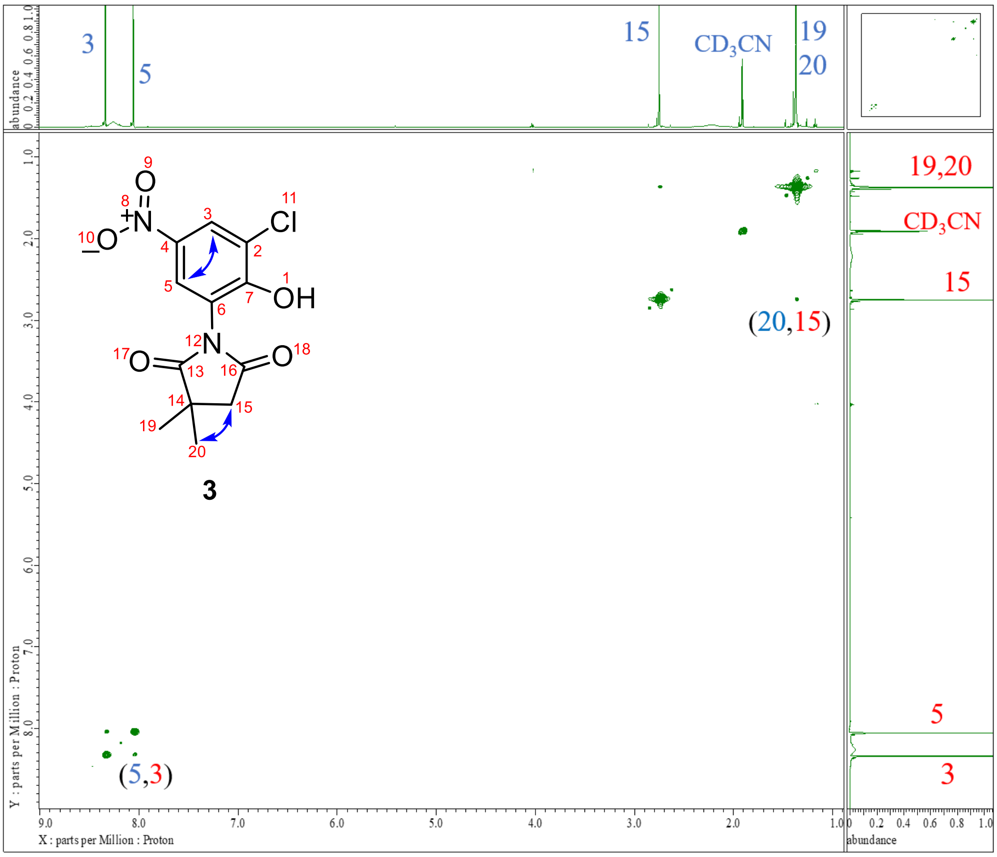

Supplementary Figure 15. ^{1}H–^{1}H COSY NMR (600 MHz, CD_3CN) spectrum of **3** at 298 K.

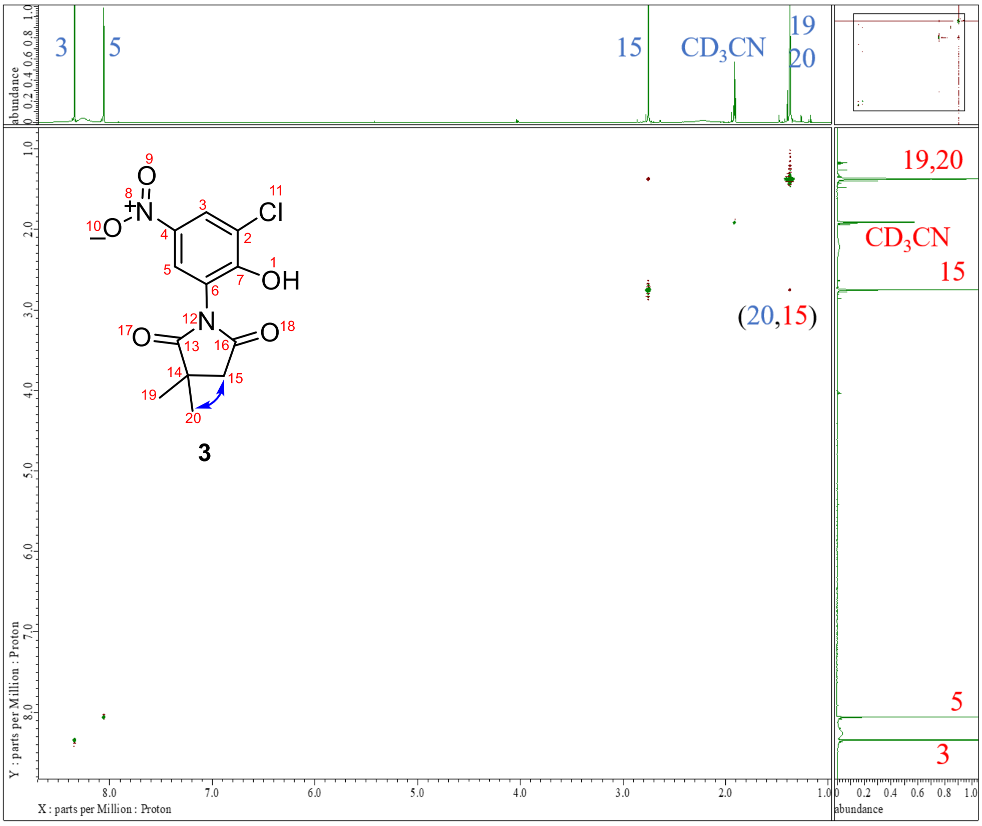

Supplementary Figure 16. ^{1}H–^{1}H NOESY NMR (600 MHz, CD3CN) spectrum of **3** at 298 K.

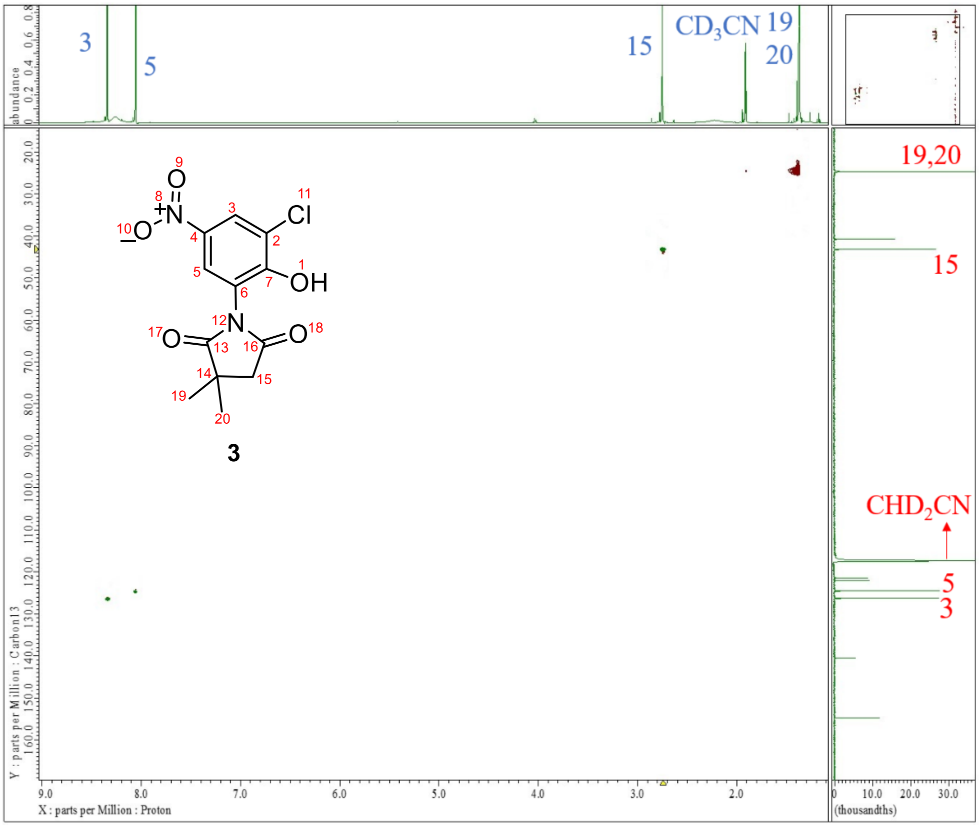

Supplementary Figure 17. ^{1}H–^{13}C HSQC NMR (600 MHz, CD3CN) spectrum of **3** at 298 K.

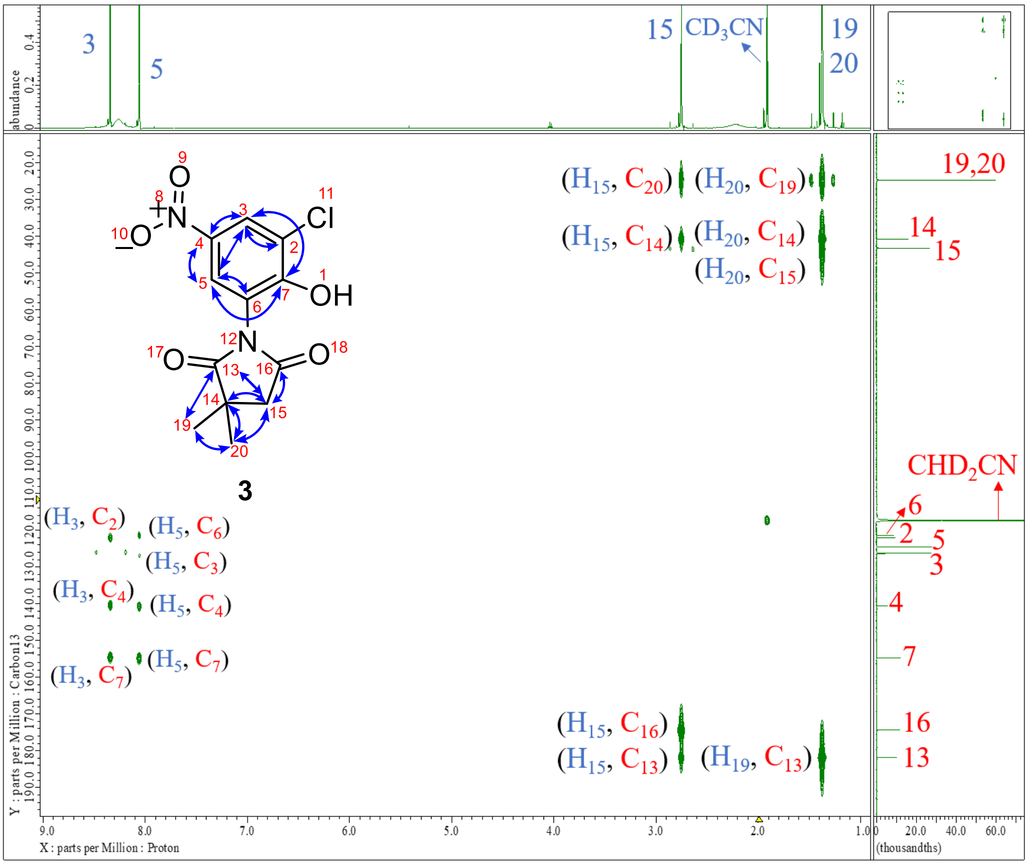

Supplementary Figure 18. ^{1}H–^{13}C HMBC NMR (600 MHz, CD_3CN) spectrum of **3** at 298 K.

Supplementary Note 6. Summary of Activation Parameters for Rotors under Different Conditions.

The activation parameters of **1** – **3** in acetone-d_6 and Li^+·(**3**–H)$^–$ in acetonitrile-d_3 derived from VT ^{1}H NMR spectra and line-shape analyses are summarized in Supplementary Table 5; the activation parameters of (**3**–H)$^–$, Na^+·(**3**–H)$^–$ and K^+·(**3**–H)$^–$ derived from 2D EXSY are summarized in Supplementary Table 6. For experimental information and more detailed fundamental data, see Pages 24 – 87 in this Supplementary Information.

Supplementary Table 5. Summary of the Activation Parameters of Some Studied Rotors that Derived from VT ^{1}H NMR Measurements and Line-shape Analyses.[a]

Studied System	Rotor	Li^+ [e] (equiv)	Range [f] of Temp (K)	Run	T_{Coales} [g] (K)	$\Delta H^‡$ (kcal mol^{-1})	$\Delta S^‡$ (cal mol^{-1} K^{-1})	$\Delta G^‡_{298\ K}$ (kcal mol^{-1})	$\Delta G^‡_{338\ K}$ (kcal mol^{-1})	Mean $\Delta H^‡$ (kcal mol^{-1})	STD [h] (Mean $\Delta H^‡$) (kcal mol^{-1})	Mean $\Delta S^‡$ (cal mol^{-1} K^{-1})	STD [i] (Mean $\Delta S^‡$) (cal mol^{-1} K^{-1})	Mean $\Delta G^‡_{298\ K}$ ($\Delta G^‡_{338\ K}$) (kcal mol^{-1})	STD [j] (Mean $\Delta G^‡_{298\ K}$) ((Mean $\Delta G^‡_{338\ K}$)) (kcal mol^{-1})
**1**[b]	**1**	/	208–243	1	228	10.94	–1.56	11.40	11.47	11.93	0.86	2.03	3.11	11.32 (11.25)	0.07 (0.20)
				2	228	12.39	3.64	11.30	11.16						
				3	228	12.47	4.00	11.27	11.11						
**2**[b]	**2**	/	208–243	1	223	11.47	1.49	11.03	10.97	11.65	0.16	2.27	0.67	10.98 (10.89)	0.04 (0.07)
				2	223	11.74	2.67	10.95	10.84						
				3	223	11.75	2.64	10.97	10.86						
**3**[b]	**3**	/	208–243	1	218	12.24	6.32	10.23	9.93	12.33	0.13	6.74	0.66	10.32 (10.05)	0.08 (0.11)
				2	218	12.47	7.50	10.36	10.12						
				3	218	12.27	6.40	10.37	10.11						
**3-DBU-LiClO$_4$**[c]	Li^+·(**3**–H)$^–$	4.0	308-353	1	328	14.13	–8.60	16.70	17.04	/	/	/	/	/	/
		2.0	308-343	1	318	14.47	–6.50	16.40	16.66	15.08	0.99	–4.75	3.07	16.56 (16.76)	0.14 (0.13)
			308-348	2		14.35	–7.59	16.61	16.91						
			308-348	3		16.21	–1.49	16.66	16.72						
		1.0	308-348	1	323	14.03	–9.02	16.72	17.08	/	/	/	/	/	/
		0.8	313-348	1	328	14.19	–8.87	16.83	17.18	/	/	/	/	/	/
		0.6	313-353	1	333	13.79	–10.75	17.00	17.43	/	/	/	/	/	/
		0.4	323-353	1	343	13.48	–12.67	17.25	17.76	/	/	/	/	/	/
		0.2	333-353	1	/	12.05	–18.99	17.71	18.47	/	/	/	/	/	/
**3-*t*BuOLi**[d]	Li^+·(**3**–H)$^–$	1.0	298-343	1	328	15.23	–5.47	16.86	17.08	15.59	1.71	–4.02	5.14	16.84 (17.00)	0.28 (0.11)
				2	323	14.08	–8.27	16.55	16.88						
				3	328	17.45	1.69	17.1	17.05						

[a] The VT ^{1}H NMR experiments were carried out in acetonitrile-d_3, unless indicated otherwise. [b] In acetone-d_6. [c] **3** in the presence of 1.2 equiv. of DBU and different equiv of lithium cations. [d] **3** in the presence of 1.0 equiv. of lithium *tert*-butoxides. [e] The amount (equiv) of lithium cations added to system of DBU-deprotonated **3**. [f] The range of temperature of the ^{1}H NMR spectra used for LSA and thermodynamic analysis. [g] T_{Coales} = coalescence temperature. [h] STD (Mean $\Delta H^‡$) = standard deviation of the enthalpy of activation. [i] STD (Mean $\Delta S^‡$) = standard deviation of the mean entropy of activation. [j] STD (Mean $\Delta G^‡_{298\ K\ (or\ 388\ K)}$) = standard deviation of the mean standard Gibbs free energy of activation at the stated temperature.

Supplementary Table 6. Summary of the Activation Parameters that Derived from 2D EXSY Measurements on **3** under Different Conditions.[a]

Studied System	Rotor	M^+ [e] (equiv)	Run	$\Delta H^‡$ (kcal mol^{-1})	$\Delta S^‡$ (cal mol^{-1} K^{-1})	$\Delta G^‡_{298\ K}$ (kcal mol^{-1})	$\Delta G^‡_{338\ K}$ (kcal mol^{-1})	Mean $\Delta H^‡$ (kcal mol^{-1})	STD [f] (Mean $\Delta H^‡$) (kcal mol^{-1})	Mean $\Delta S^‡$ (cal mol^{-1} K^{-1})	STD [g] (Mean $\Delta S^‡$) (cal mol^{-1} K^{-1})	Mean $\Delta G^‡_{298\ K}$ ($\Delta G^‡_{338\ K}$) (kcal mol^{-1})	STD [h] (Mean $\Delta G^‡_{298\ K}$) ((Mean $\Delta G^‡_{338\ K}$)) (kcal mol^{-1})
**3-DBU**[b]	(**3**–H)$^-$	/	1	21.07	4.63	19.67	19.5	20.56	0.73	3.19	2.10	19.60 (16.48)	0.10 (0.02)
			2	19.72	0.78	19.49	19.46						
			3	20.89	4.15	19.65	19.49						
**3-DBU-NaClO$_4$**[c]	Na^+·(**3**–H)$^-$	4.0	1	15.68	–7.37	17.88	18.18	/	/	/	/	/	/
		2.0	1	15.07	–9.08	17.78	18.14	14.59	1.60	–10.46	4.47	17.70 (18.12)	0.26 (0.09)
			2	15.89	–6.84	17.92	18.20						
			3	12.8	–15.46	17.41	18.03						
		1.0	1	15.55	–8.51	18.09	18.43	/	/	/	/	/	/
		0.75	1	14.79	–11.35	18.17	18.62	/	/	/	/	/	/
		0.5	1	14.99	–11.07	18.29	18.73	/	/	/	/	/	/
		0.25	1	17.48	–4.78	18.90	19.09	/	/	/	/	/	/
**3-*t*BuONa**[d]	Na^+·(**3**–H)$^-$	1.0	1	16.29	–6.30	18.17	18.42	15.28	1.43	–9.38	4.36	18.08 (18.45)	0.13 (0.04)
			2	14.27	–12.46	17.98	18.48						
**3-DBU-KClO$_4$**[c]	K^+·(**3**–H)$^-$	4.0	1	20.35	2.96	19.46	19.35	/	/	/	/	/	/
		2.0	1	23.77	13.05	19.87	19.36	21.05	2.44	5.05	7.16	19.54 (19.34)	0.30 (0.04)
			2	19.05	–0.74	19.27	19.30						
			3	20.33	2.84	19.48	19.37						
		1.0	1	21.75	7.11	19.63	19.35	/	/	/	/	/	/
		0.5	1	20.91	4.28	19.63	19.46	/	/	/	/	/	/
**3-*t*BuOK**[d]	K^+·(**3**–H)$^-$	1.0	1	18.49	–3.39	19.50	19.64	19.85	1.33	1.28	4.40	19.46 (19.42)	0.13 (0.19)
			2	19.93	1.9	19.36	19.29						
			3	21.14	5.34	19.55	19.34						

[a] The 2D EXSY experiments were carried out in acetonitrile-d_3. [b] **3** in the presence of 1.2 equiv of DBU. [c] **3** in the presence of 1.2 equiv of DBU and different equiv of metal cations. [d] **3** in the presence of alkali metal *tert*-butoxides. [e] The amount (equiv) of metal cations presented in the system. [f] STD (Mean $\Delta H^‡$) = standard deviation of the enthalpy of activation. [g] STD (Mean $\Delta S^‡$) = standard deviation of the mean entropy of activation. [h] STD (Mean $\Delta G^‡_{298\ K\ (or\ 388\ K)}$) = standard deviation of the mean standard Gibbs free energy of activation at the stated temperature.

Supplementary Method 4. Kinetics Studies on Rotors **1 – 3**

Variable-temperature (VT) ^{1}H NMR investigations were first carried out to characterize the dynamic features of **1** – **3**. In acetonitrile-d_3 (m.p. –45 °C, 228 K), the VT ^{1}H NMR signals of the methyl and the methylene protons of all of the studied molecules showed no clear signs of decoalescence, even at the low-temperature limit of 233 K (Supplementary Fig. 20–22). Thus, VT ^{1}H NMR were then carried in acetone-d_6, a solvent that has a much lower freezing-point (m.p. –94 °C, 179 K). At ambient temperature, as expected for the fast rotation of imide rotator around the C(aryl)−N(imide) linkage on the NMR time scale, the two diastereotopic methyl groups are magnetically equivalent in all cases. Consequently, a sharp singlet for methyl protons were observed (Supplementary Fig. 23, 26 and 29). Decreasing the temperature slowed down the rotation of rotators, leading to considerable broadening followed by splitting of the resonance of the methyl and the methylene protons. At low-temperature limit of 188 K, the rotor displayed a highly restricted rotation.

The investigation on each rotor was totally repeated for three times with three independent samples. Calculations of the exchange rates (k, s^{-1}) were performed by line shape analysis(LSA)[4] of the experimental ^{1}H NMR signals of the methylene protons; the enthalpic ($\Delta H^{\ddagger}$) and entropic ($\Delta S^{\ddagger}$) contributions to the transition state of $\Delta H^{\ddagger}$ and $\Delta S^{\ddagger}$ were derived from Eyring plots[5] (Supplementary Fig. 25, 28 and 31). The values of $\Delta G^{\ddagger}$ and the rational speed (k_{rot}, s^{-1}) at 298 K and 338 K, respectively, were also calculated. These results are summarized in Supplementary Table 5 in this Supplementary Information; more concise results are summarized in Table 1 in the main text.

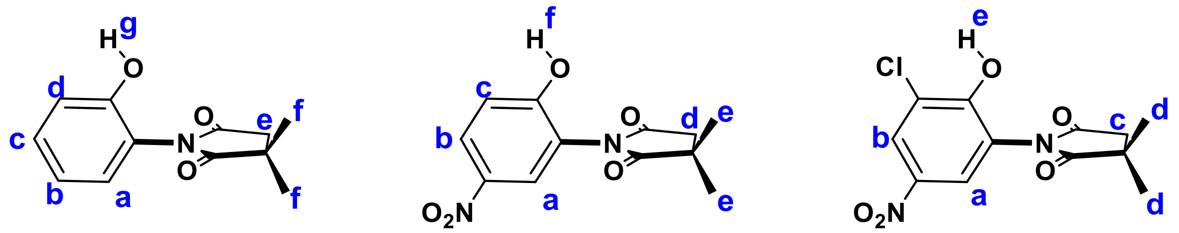

1 **2** **3**

Supplementary Figure 19. Structures of **1** – **3** and the labels of the protons.

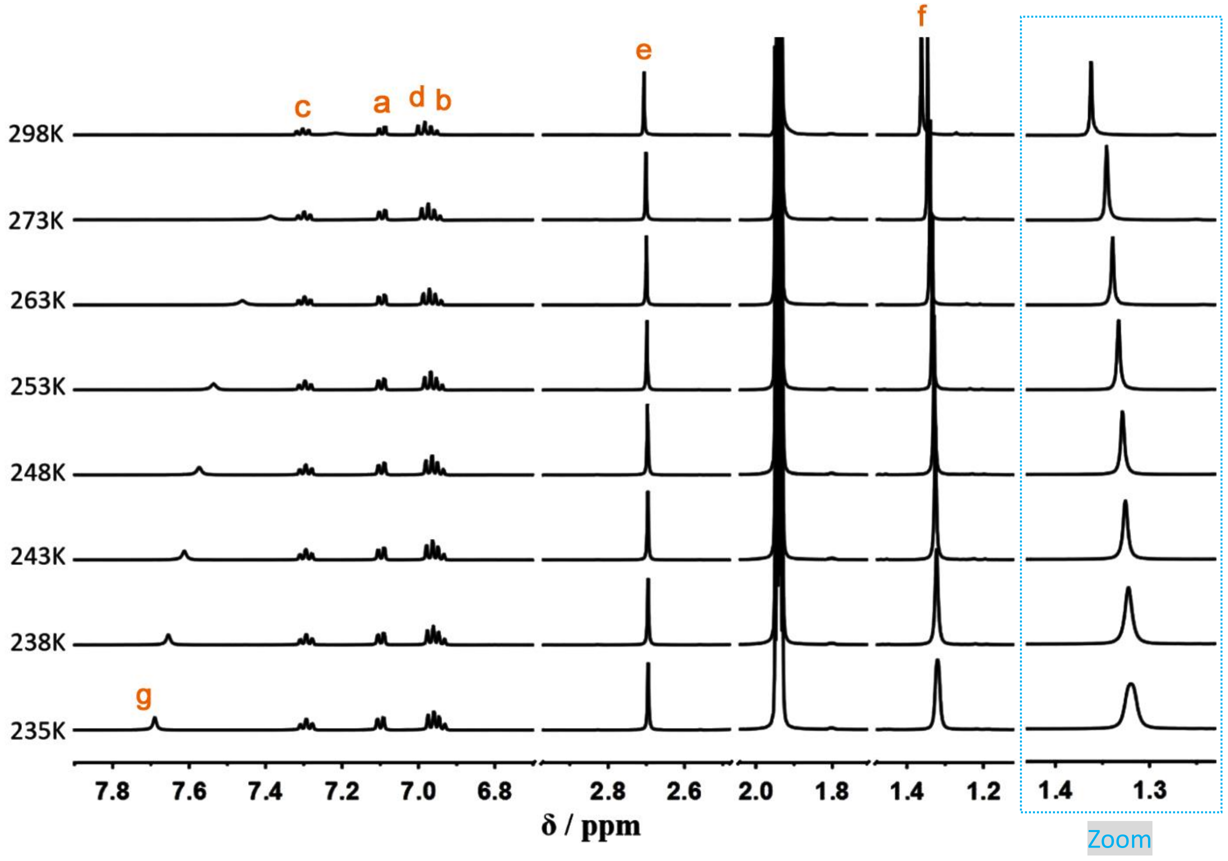

Supplementary Figure 20. VT ^{1}H NMR spectra (500 MHz, CD_3CN) of **1** (4 mM).

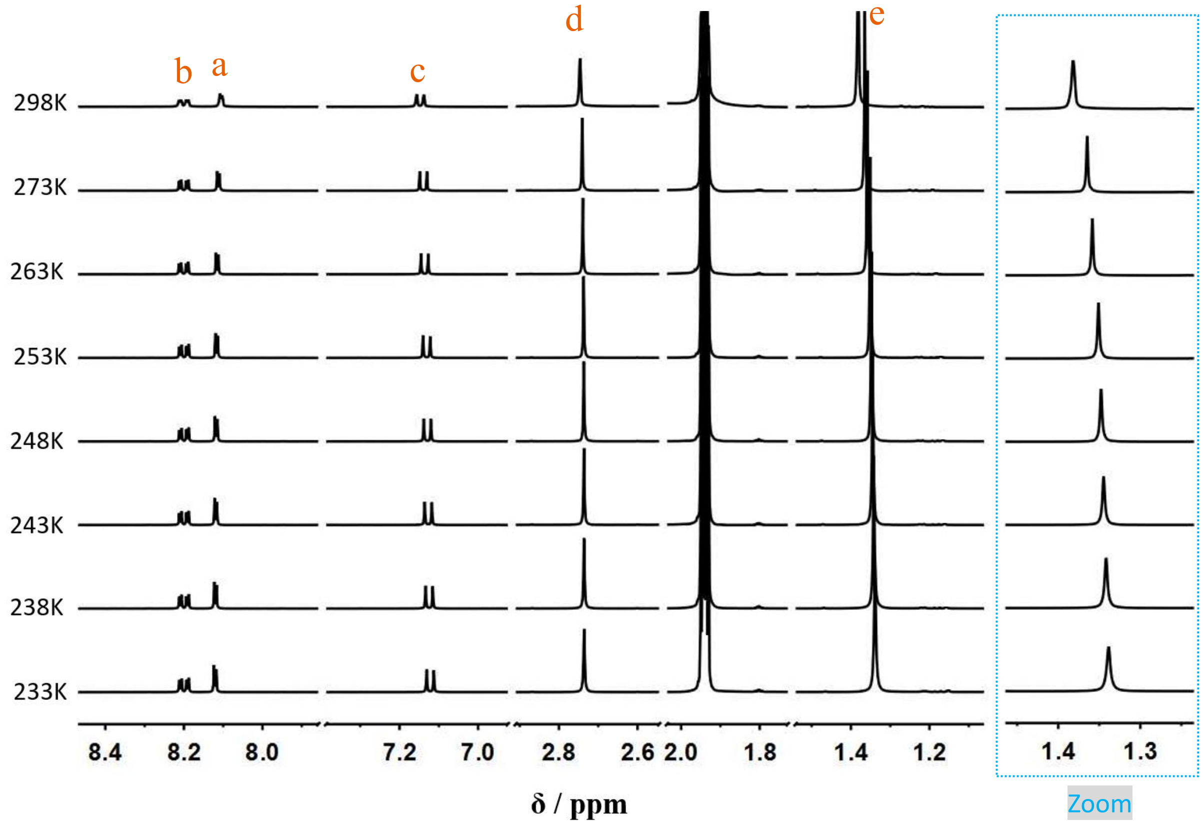

Supplementary Figure 21. VT ^{1}H NMR spectra (500 MHz, CD_3CN) of **2** (4 mM).

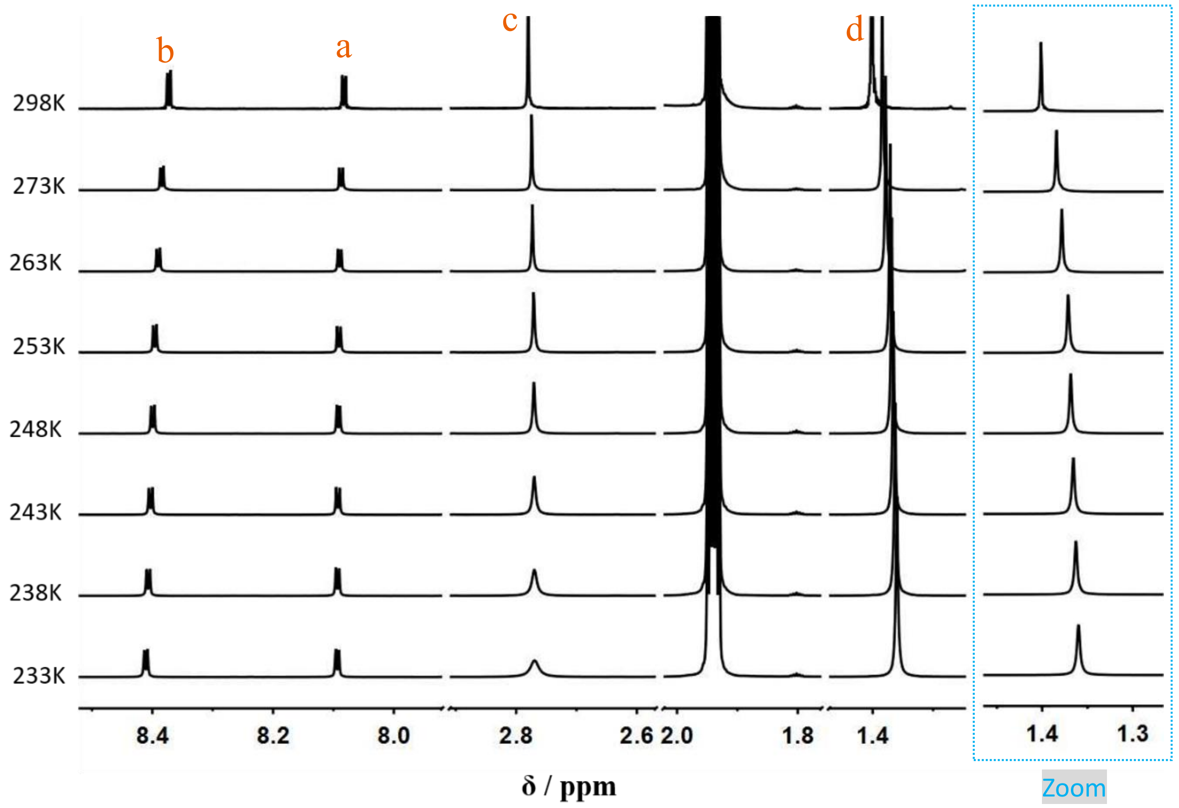

Supplementary Figure 22. VT ^{1}H NMR spectra (500 MHz, CD_3CN) of **3** (4 mM).

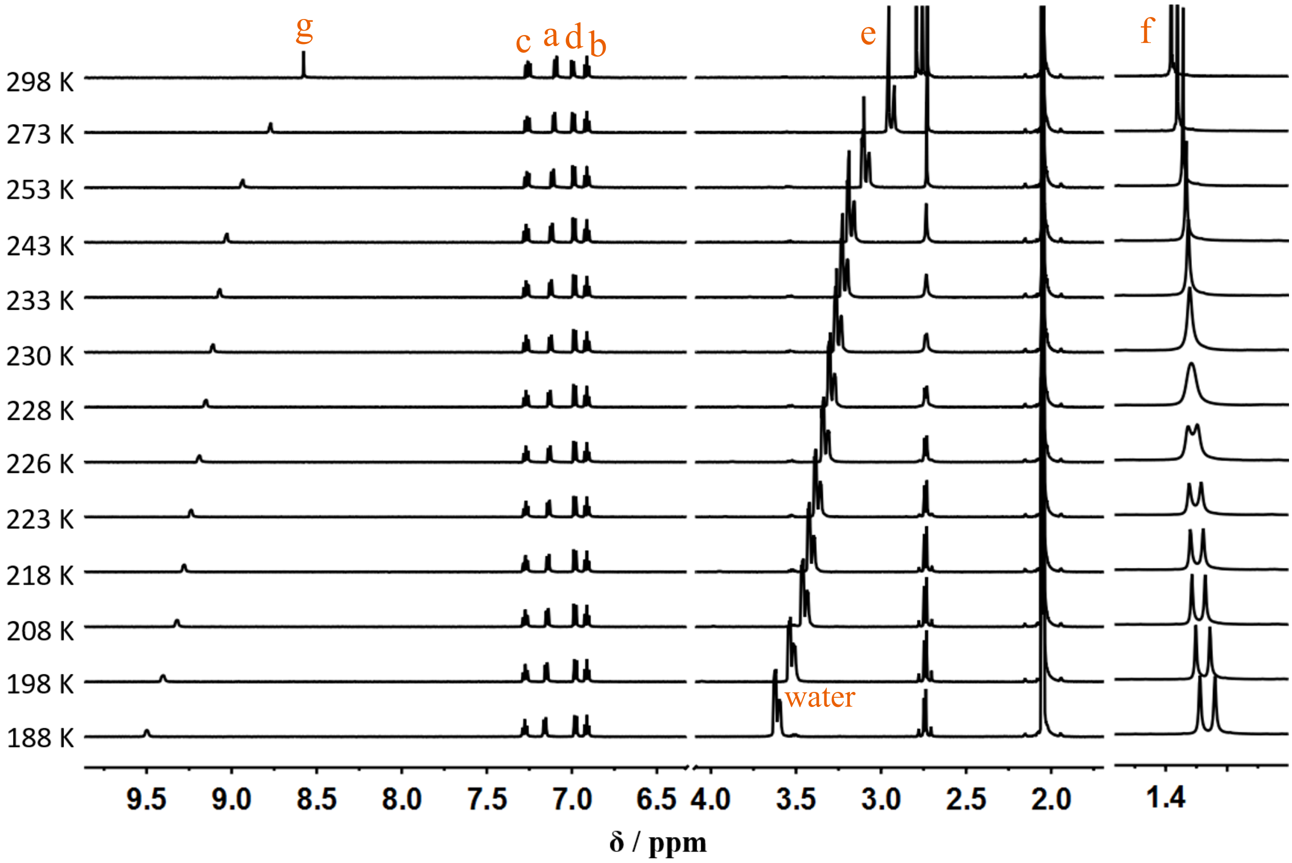

Supplementary Figure 23. VT ^{1}H NMR spectra (500 MHz, CD_3COCD_3) of **1** (4 mM).

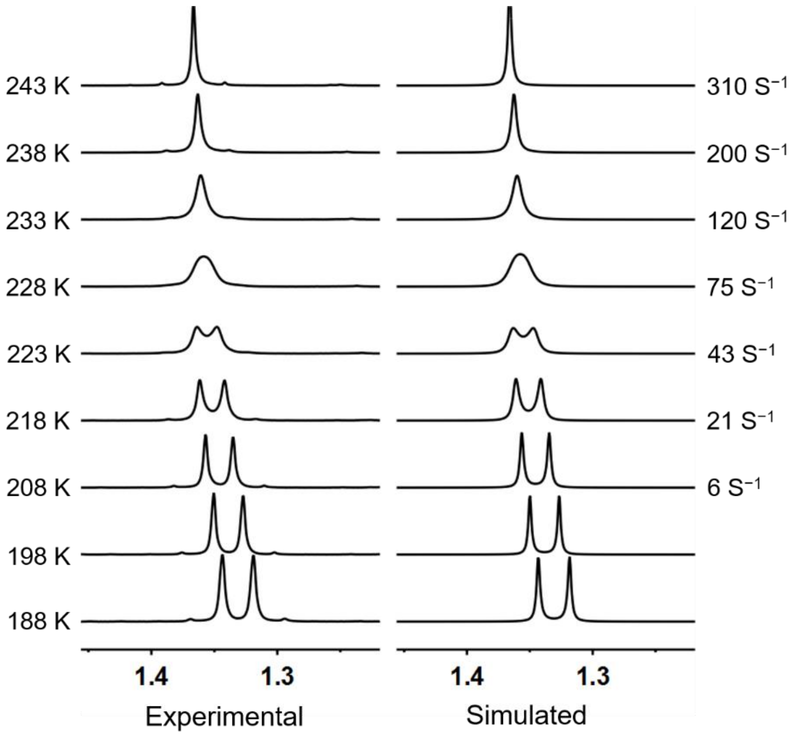

Supplementary Figure 24. Experimental and simulated VT ^{1}H NMR spectra (500 MHz) of **1** (4 mM) at the region of methyl protons H_f in acetone-d_6. The temperature (K) and calculated exchange rate constants (k, s^{-1}) are given for each trace.

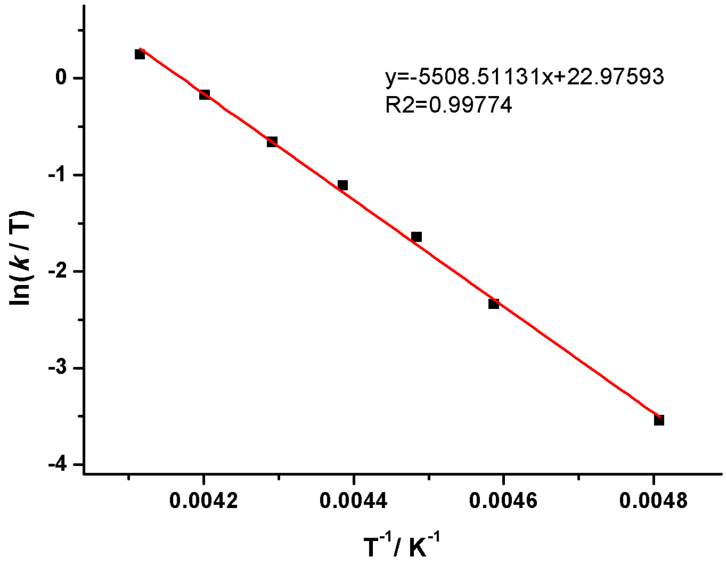

Supplementary Figure 25. Eyring plot of the corresponding chemical exchange obtained from line width analysis of methyl protons H_f signals in VT ^{1}H NMR spectra of rotor **1** in acetone-d_6.

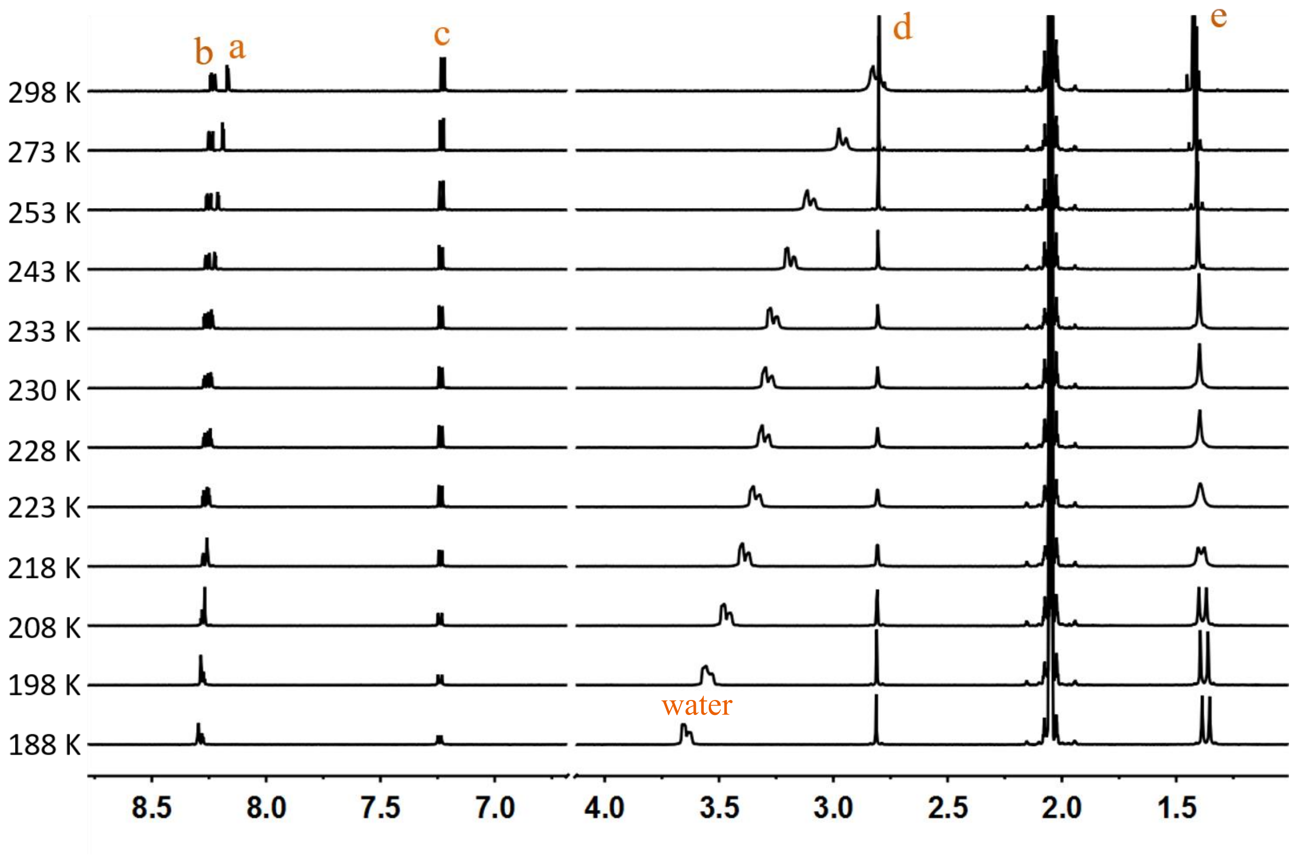

Supplementary Figure 26. VT ^{1}H NMR spectra (500 MHz, CD_3COCD_3) of **2** (4 mM).

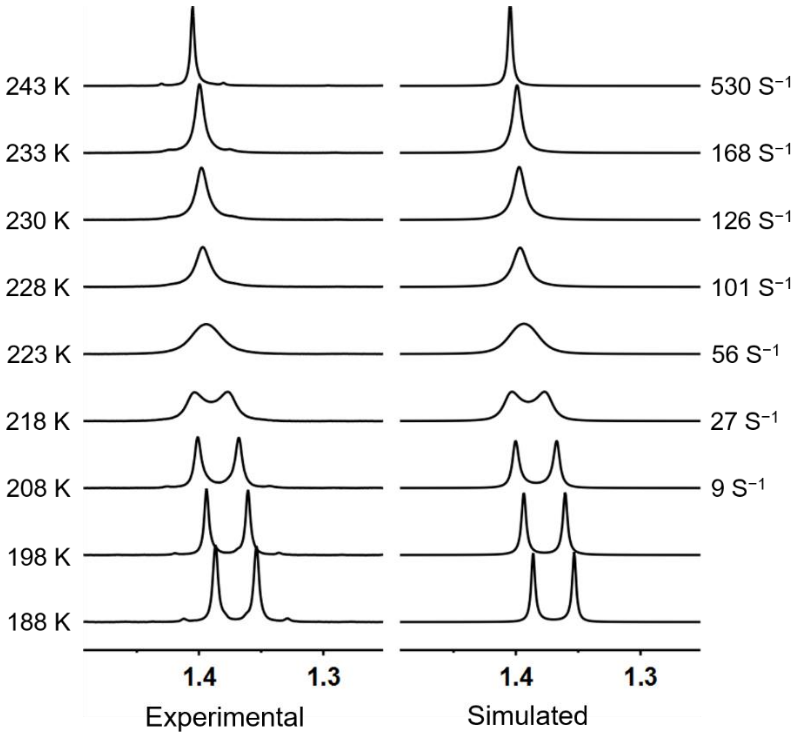

Supplementary Figure 27. Experimental and simulated VT ^{1}H NMR spectra (500 MHz) of **2** (4 mM) at the region of methyl protons H_e in acetone-d_6. The temperature (K) and calculated exchange rate constants (k, s^{-1}) are given for each trace.

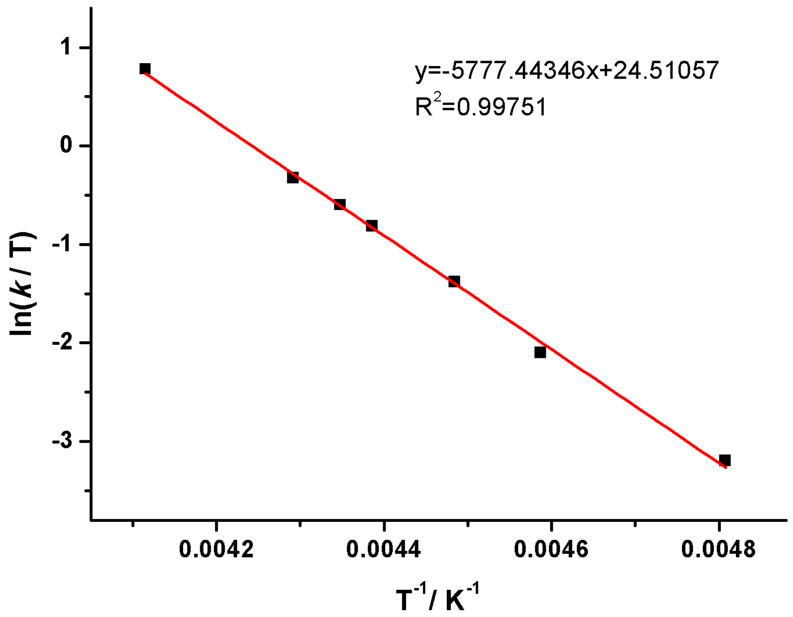

Supplementary Figure 28. Eyring plot of the corresponding chemical exchange obtained from line width analysis of methyl protons H_e signals on VT ^{1}H NMR spectra of rotor **2** in acetone-d_6.

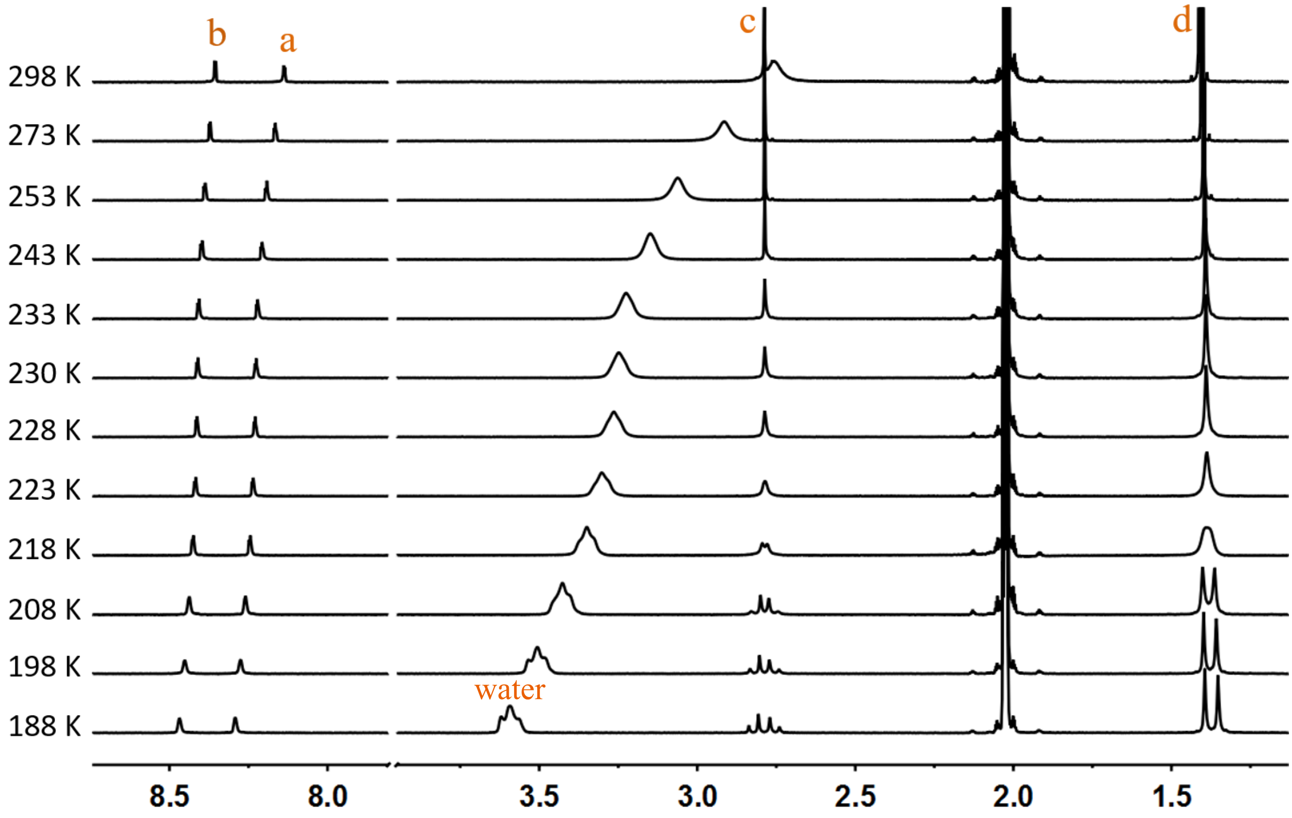

Supplementary Figure 29. VT ^{1}H NMR spectra (500 MHz, CD_3COCD_3) of **3** (4 mM).

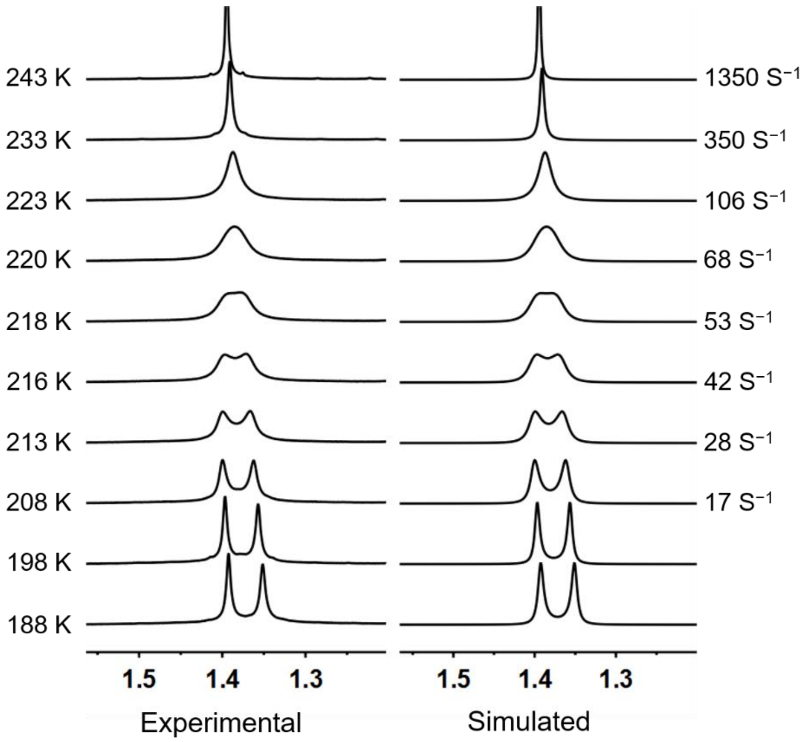

Supplementary Figure 30. Experimental and simulated VT ^{1}H NMR spectra (500 MHz) of **3** (4 mM) at the region of methyl protons H_d in acetone-d_6. The temperature (K) and calculated exchange rate constants (k, s^{-1}) are given for each trace.

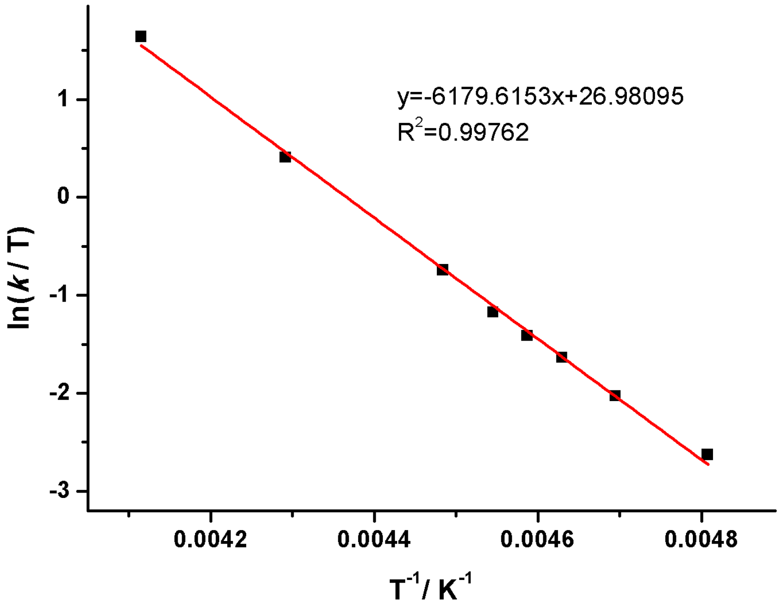

Supplementary Figure 31. Eyring plot of the corresponding chemical exchange obtained from line width analysis of methyl protons H_d signals in VT ^{1}H NMR spectra of rotor **3** in acetone-d_6.

Supplementary Method 5. Studies on Base-mediated Rotation of **1 – 3**

^{1}H NMR DBU titration Experiments. To select a good system that suitable for establishing molecular rotor with multistage speed from candidates **1 – 3**, we carried out ^{1}H NMR titration Experiments. The experiments were carried out by a gradual addition of the concentrated solution of 1,8-diazabicyclo[5.4.0]undec-7-ene (DBU, 500 mM) in acetonitrile-d_3 to the rotor **1 – 3** (4 mM, 0.5 mL) in acetonitrile-d_3. In this process, a serial of ^{1}H NMR spectra of the sample was recorded by a JEOL 600 MHz (^{1}H) spectrometer at 298 K. Typically, more than 10 points were recorded in these processes. The obtained titration spectra are shown in Supplementary Fig. 32 – 34, which showed a clear superiority of **3** to serve as a speed-tunable rotor over **1** and **2** ascribed to its lower pKa.

We also carried out the DBU titration experiment on **3** in acetone-d_6 (Supplementary Fig. 35). In contrast to that in the case of acetonitrile-d_3, the methylene protons showed up as an AB quartet, but the methyl protons appeared a singlet only, probably ascribed to the interaction between the solvent and the carbonyl groups on the molecules. Considering the non-coupled decoalescent methyl signal is more favorable for dynamic analysis (especially for 2D EXSY measurements), acetonitrile-d_3 seems to be a better solvent for the following studies.

Rotational Barrier of 3-DBU. Samples of **3-DBU** were prepared by mixing **3** with 1.2 equivalents of DBU, using acetonitrile-d_3 as the solvent. VT ^{1}H NMR measurements were first performed on such solution to explore the changes of ^{1}H NMR signal pattern of methyl protons upon the change of the temperature. However, with the increase of the temperature, the methyl protons appeared as a doublet at all the temperatures studied, even at the high-temperature limit of 353 K (Supplementary Fig. 36). This preliminarily indicates that the rotation of rotor **3-DBU** is highly restricted in the whole range of temperatures examined.

To obtain an exact rotational barrier of **3-DBU**, 2D EXSY experiments were carried out at totally 7 different temperatures, including 348 K, 343 K, 338 K, 328 K, 318 K, 308 K and 298 K. However, at 298 K, no clear diagonal peak could be observed. The obtained exchange rates at other 6 different temperatures were thus used for the following calculations. The investigation was totally repeated for three times with three independent samples. Examples of the obtained 2D EXSY NMR spectra are shown in Supplementary Fig. 37 – 42. The normalized intensities of the cross peaks and the diagonal ones as well as the calculated exchange rates (k, s^{-1}) at every single 2D EXSY NMR spectrum are shown in Supplementary Table 7. Example of the corresponding Eyring plot is shown in

Supplementary Fig. 43. The obtained kinetic parameters, including the rotational speed of **3-DBU** (k_{rot}, s^{-1}), are shown in Supplementary Table 6 in this Supplementary Information and Table 1 in the main text.

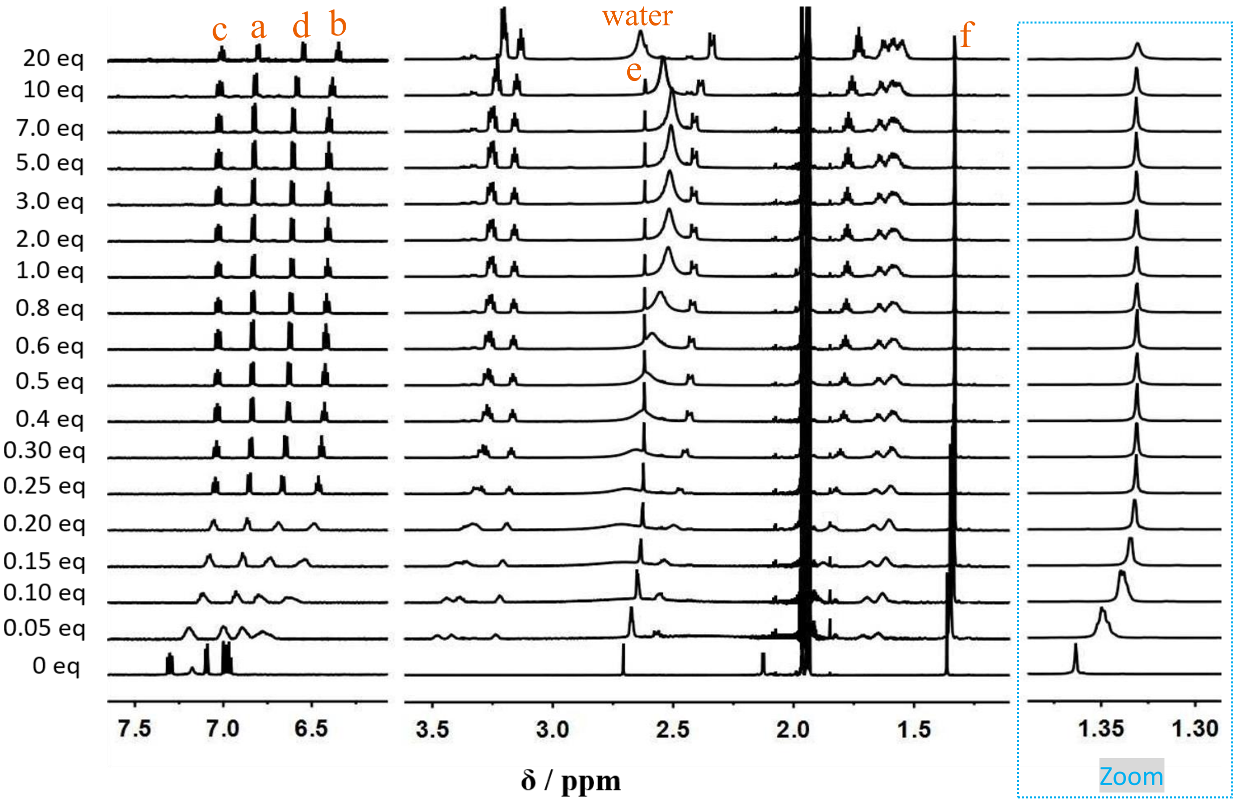

Supplementary Figure 32. Changes of ^{1}H NMR spectra (400 MHz, 298 K) of compound **1** (4 mM) in CD_3CN upon addition of up to 20 equivalents of DBU.

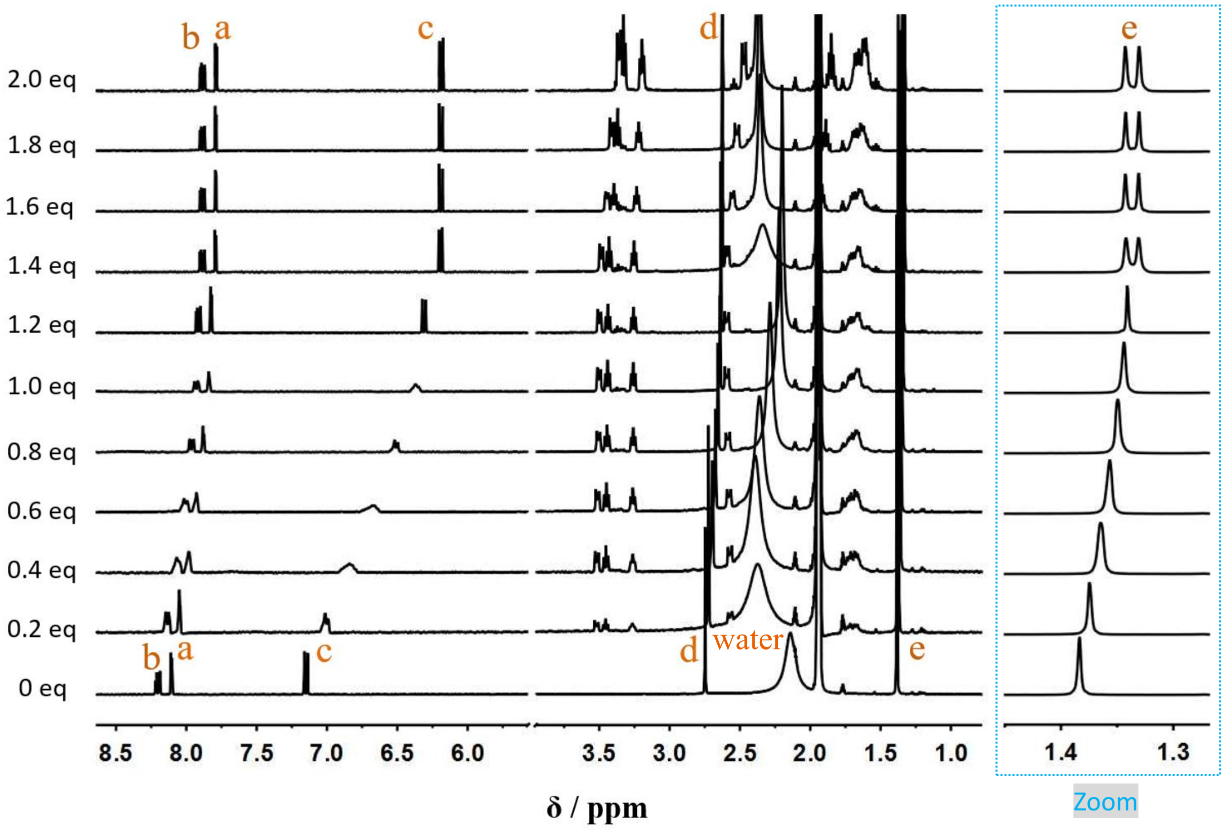

Supplementary Figure 33. Changes of ^{1}H NMR spectra (400 MHz, 298 K) of compound **2** (4 mM) in CD_3CN upon addition of DBU.

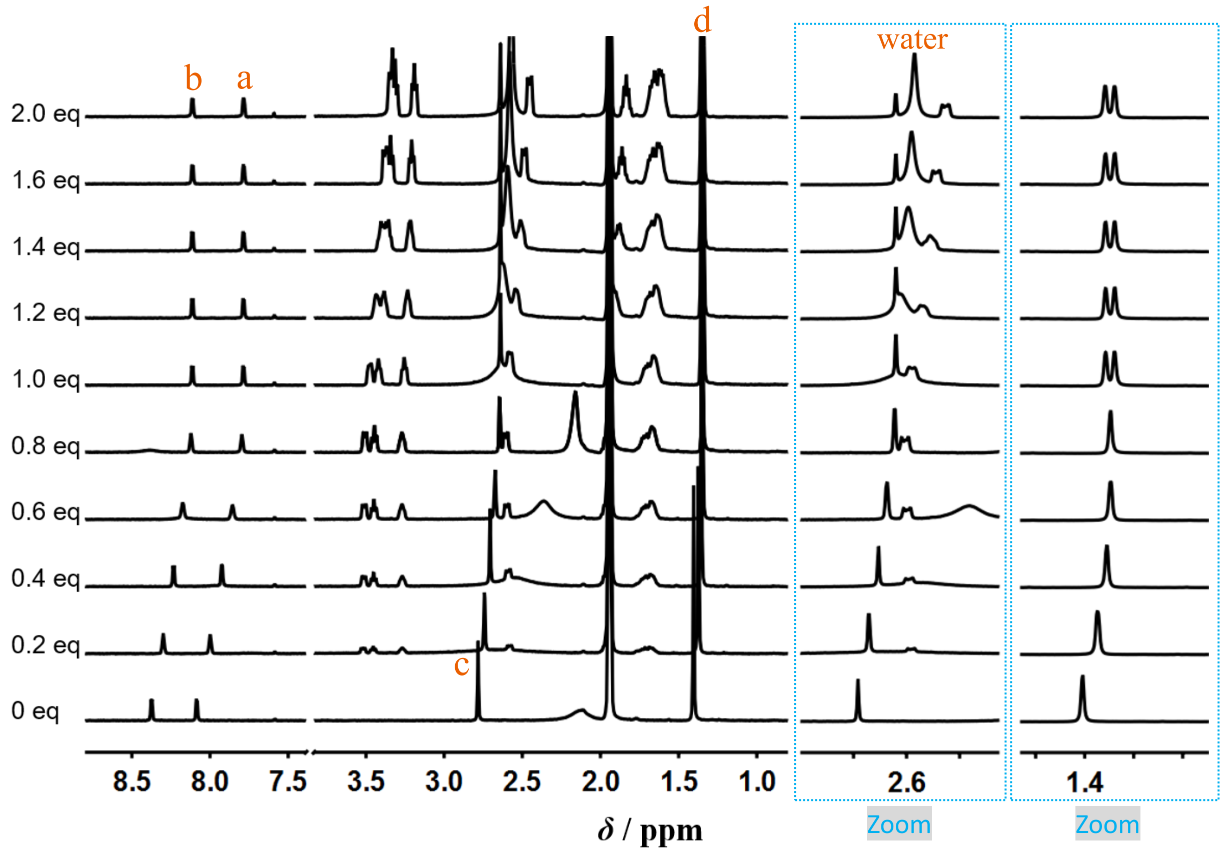

Supplementary Figure 34. Changes of ^{1}H NMR spectra (400 MHz, 298 K) of compound **3** (4 mM) in CD_3CN upon addition of DBU.

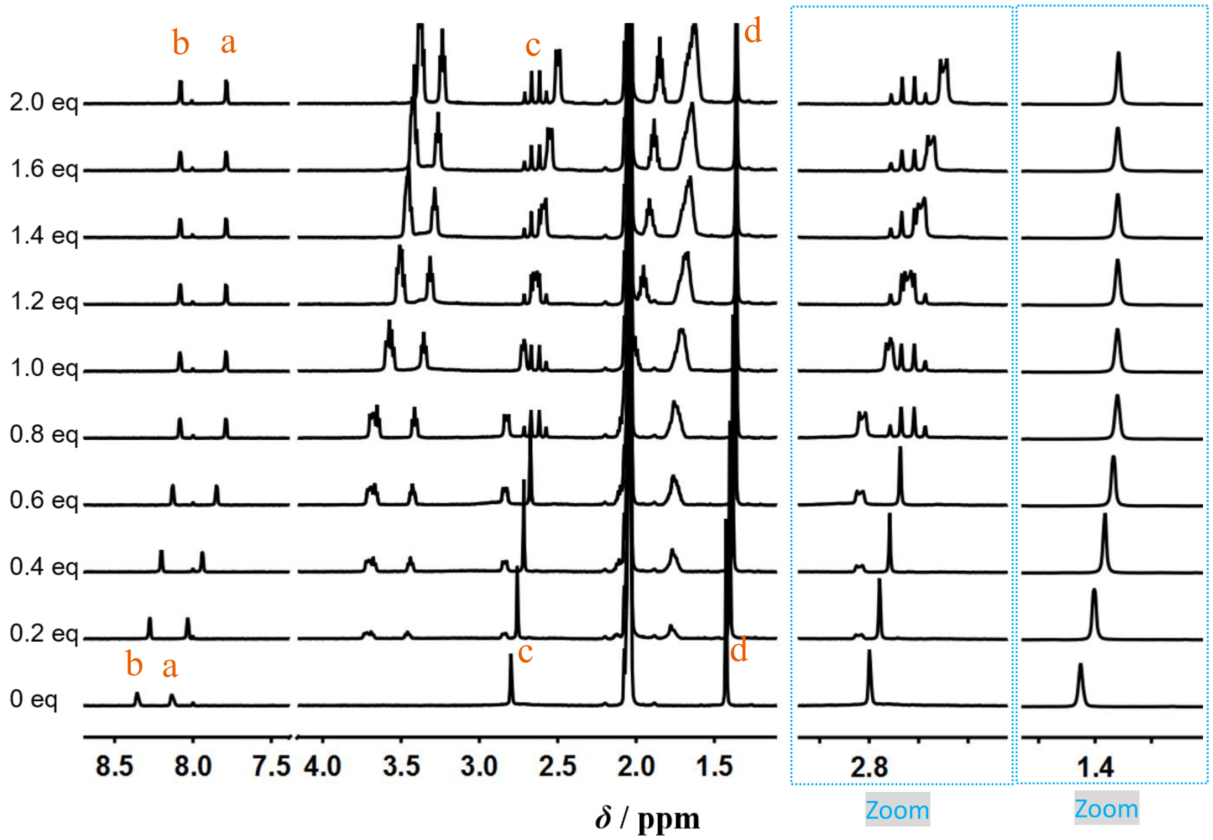

Supplementary Figure 35. Changes of ^{1}H NMR spectra (400 MHz, 298 K) of compound **3** (4 mM) in acetone-d_6 upon addition of DBU.

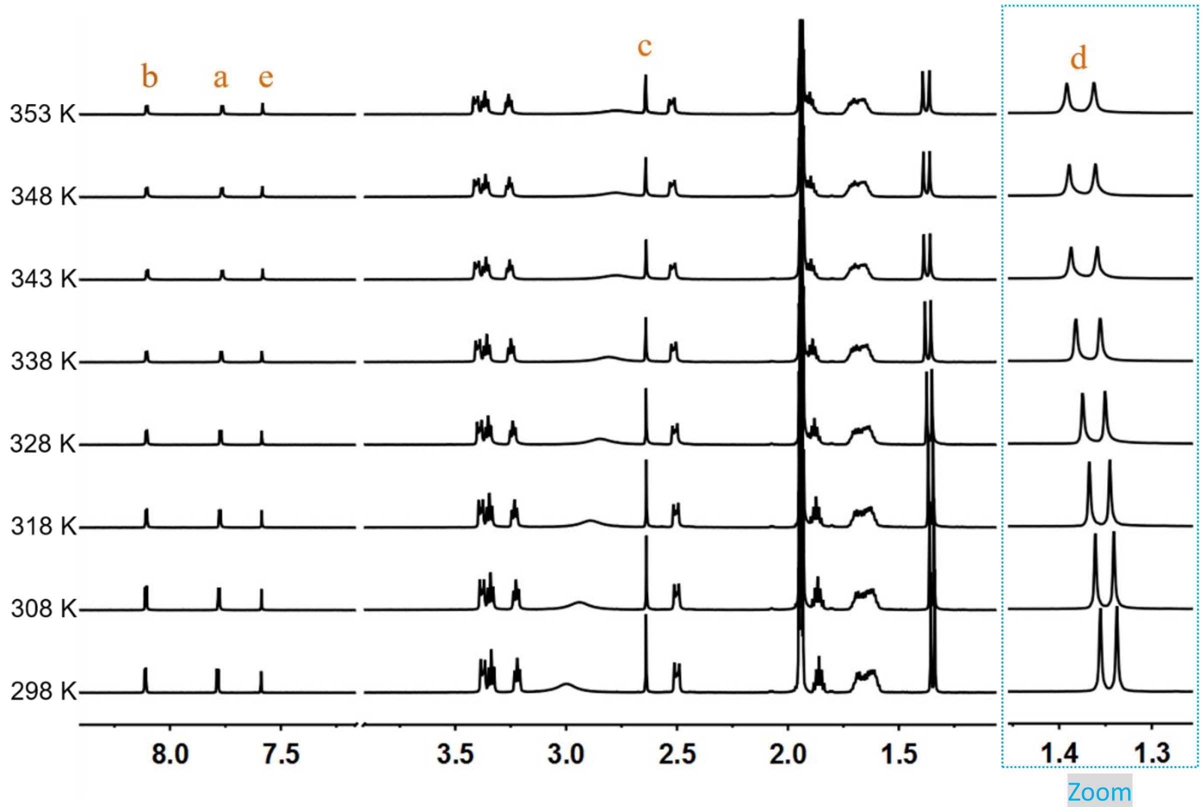

Supplementary Figure 36. VT ^{1}H NMR spectra (500 MHz, CD_3CN) of **3-DBU**. [**3**] = 4 mM, [**DBU**] = 4.8 mM (1.2 equiv).

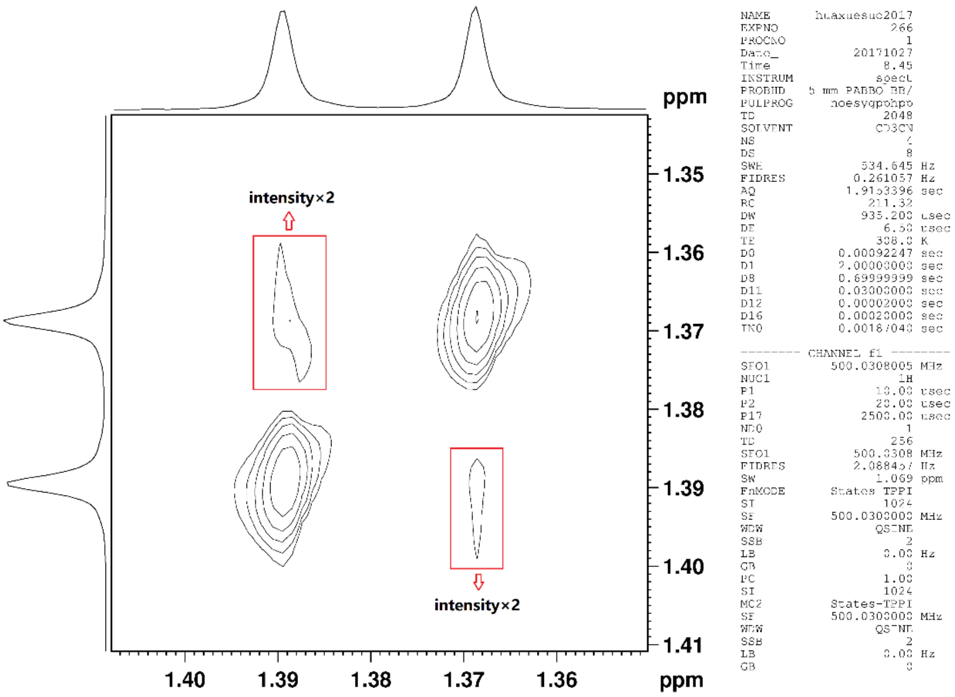

Supplementary Figure 37. EXSY NMR (500 MHz, $t_m = 0.7$ s) spectrum of **3-DBU** at 308 K in CD_3CN. [**3**] = 4 mM, [**DBU**] = 4.8 mM (1.2 equiv).

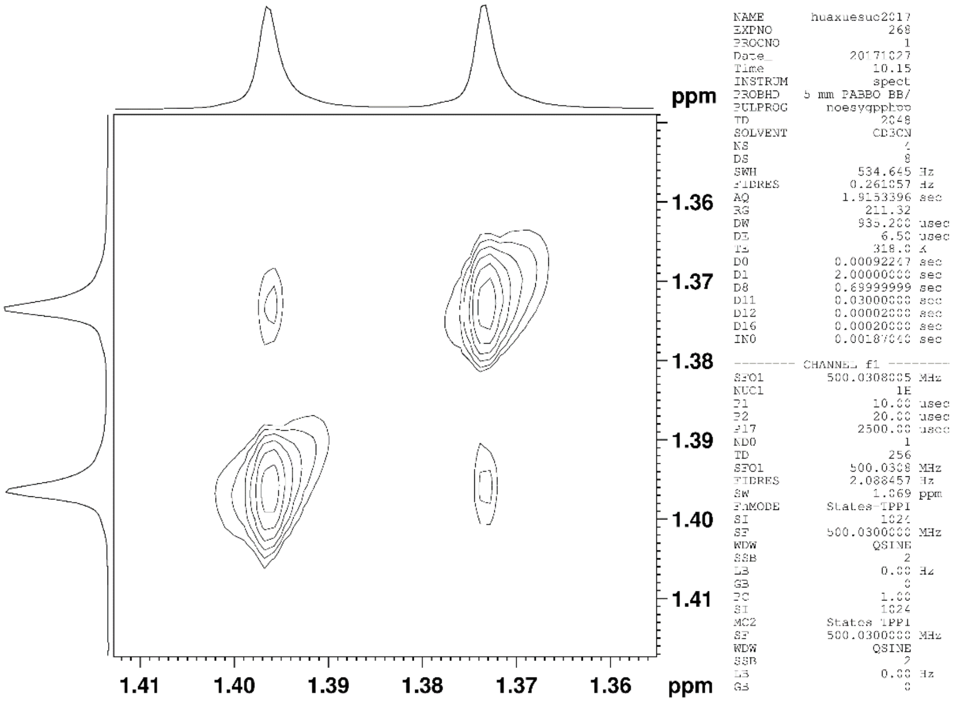

Supplementary Figure 38. EXSY NMR (500 MHz, $t_m = 0.7$ s) spectrum of **3-DBU** at 318 K in CD_3CN. [**3**] = 4 mM, [**DBU**] = 4.8 mM (1.2 equiv).

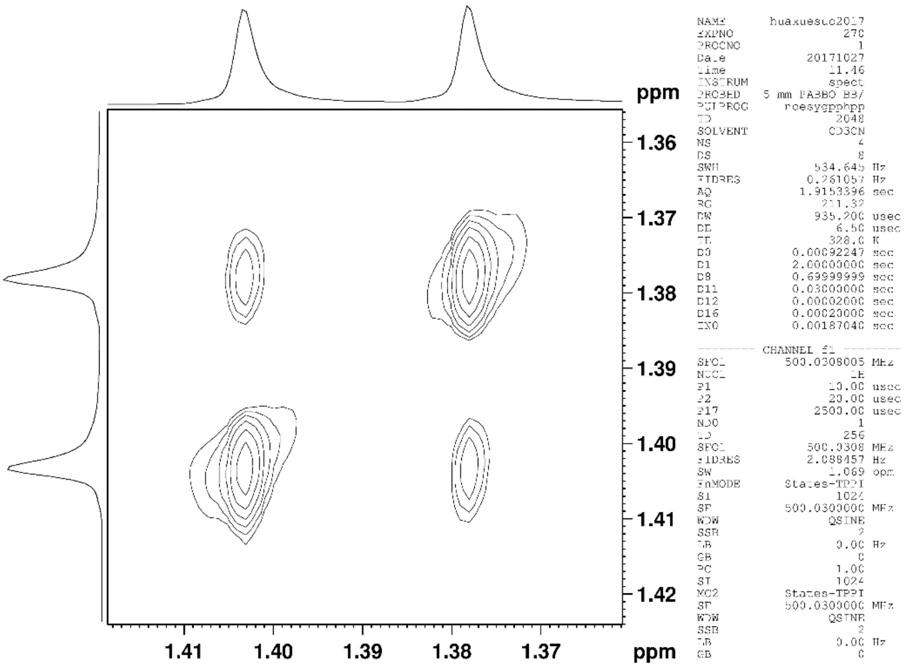

Supplementary Figure 39. EXSY NMR (500 MHz, t_m= 0.7 s) spectrum of **3-DBU** at 328 K in CD_3CN. [**3**] = 4 mM, [**DBU**] = 4.8 mM (1.2 equiv).

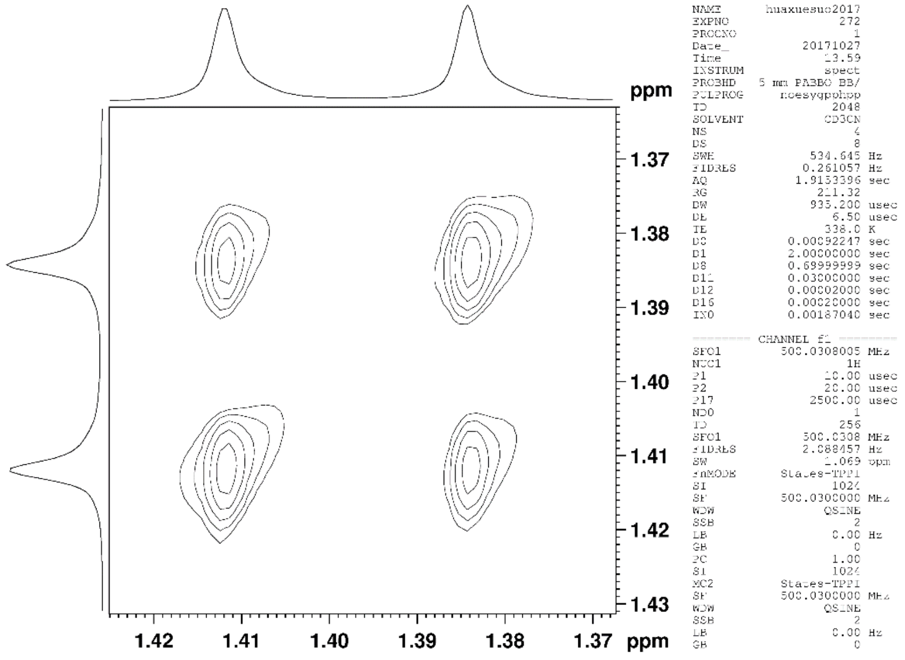

Supplementary Figure 40. EXSY NMR (500 MHz, t_m= 0.7 s) spectrum of **3-DBU** at 338 K in CD_3CN. [**3**] = 4 mM, [**DBU**] = 4.8 mM (1.2 equiv).

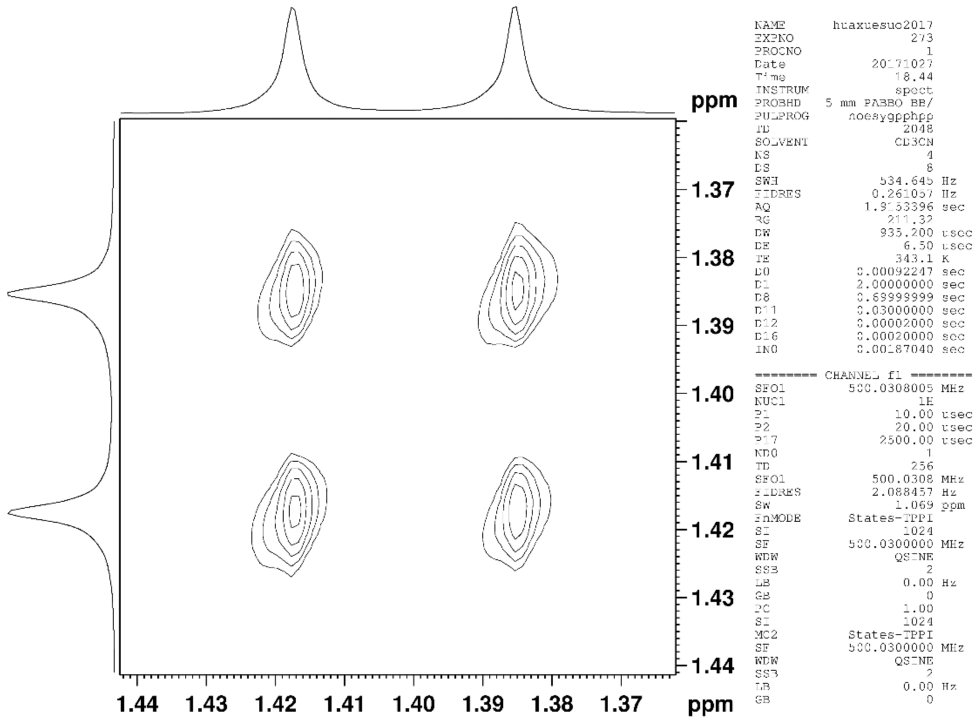

Supplementary Figure 41. EXSY NMR (500 MHz, t_m= 0.7 s) spectrum of **3-DBU** at 343 K in CD_3CN. [**3**] = 4 mM, [**DBU**] = 4.8 mM (1.2 equiv).

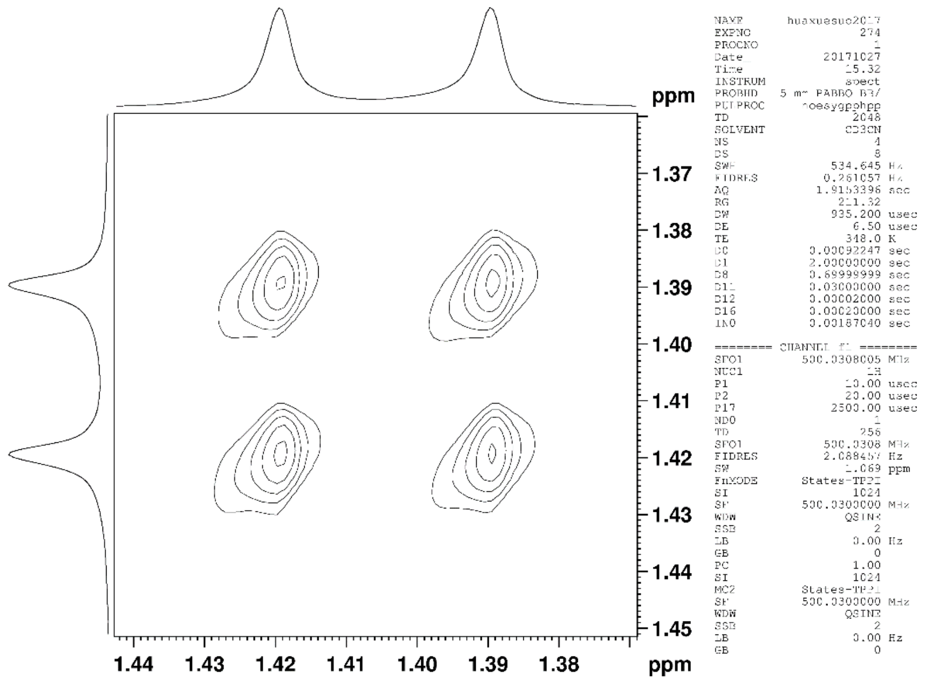

Supplementary Figure 42. EXSY NMR (500 MHz, t_m= 0.7 s) spectrum of **3-DBU** at 348 K in CD_3CN. [**3**] = 4 mM, [**DBU**] = 4.8 mM (1.2 equiv).

Supplementary Table 7. The integral and dynamic parameters derived from 2D EXSY NMR of **3-DBU.**

T(K)	I_A	I_B	I_{AB}	I_{BA}	k (s^{-1})
**308**	1.0000	1.0079	0.0672	0.0507	0.17
**318**	1.0000	1.0001	0.0887	0.0782	0.24
**328**	1.0000	0.9997	0.2109	0.1980	0.59
**338**	1.0000	0.9930	0.5254	0.5355	1.70
**343**	1.0000	0.9763	0.7358	0.7309	2.73
**348**	1.0000	0.9872	0.89923	0.9196	4.45

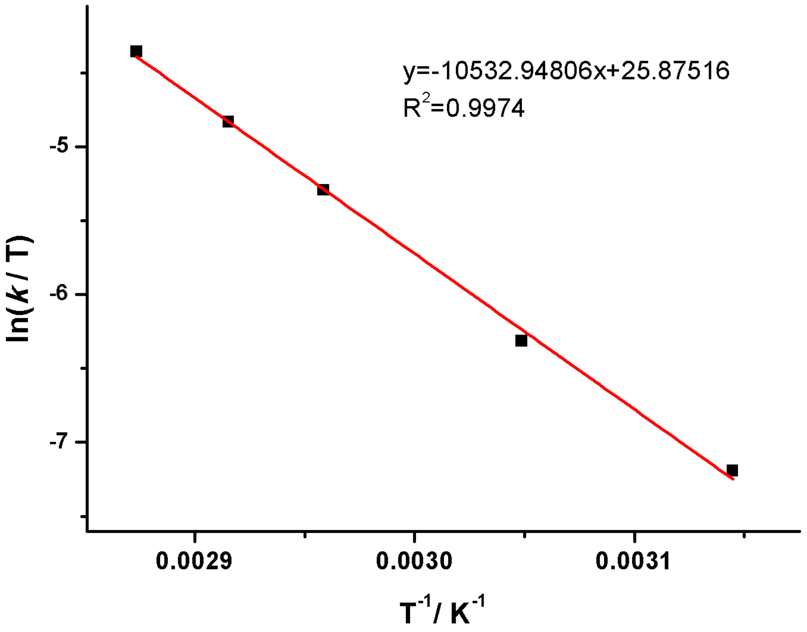

Supplementary Figure 43. Eyring plot of the chemical exchange between two diastereotopic methyl groups derived from 2D EXSY (from 308 K to 348 K) experiments on **3-DBU** in acetonitrile-d_3.

Supplementary Method 6. Studies on the Metal-Cation-Mediated Rotation of (**3**–H)$^{-}$

Three alkali-metal cations, including Li^{+}, Na^{+} and K^{+}, were chosen in our studies to verify the abilities of metal cations to regulate the rotation of (**3**–H)$^{-}$.

Dynamic properties of Li^{+}-cation-regulated system were measured by VT ^{1}H NMR. The samples for the measurements were prepared by adding 1.2 equivalents of DBU to a solution of **3** (4 mM, 0.5 mL) in acetonitrile-d_3, followed by the addition of 2.0 equivalents of $LiClO_4$ (8 μL, 500 mM in acetonitrile-d_3), in which excess base and Li^{+} cations were added in order to make sure that molecule **3** has converted completely to lithium-cation-paired (**3**–H)$^{-}$ ion pairs (Li^{+}·(**3**–H)$^{-}$) in this process. The investigation was totally repeated for three times with three independent samples. Example of the obtained VT ^{1}H NMR spectra are shown in Supplementary Fig. 44. The corresponding Eyring plot is shown in Supplementary Fig. 46. The calculated dynamic parameters are shown in Supplementary Table 5 in this Supplementary Information and Table 1 in the main text.

Dynamic properties of Na^{+}·(**3**–H)$^{-}$ and K^{+}·(**3**–H)$^{-}$ were measured by 2D EXSY NMR. The samples for the investigations were prepared in the same way as those in the case of Li^{+}·(**3**–H)$^{-}$. The investigations were totally repeated for three times with three independent samples. Example of the obtained 2D EXSY NMR spectra are shown in Supplementary Fig. 47 – 50 and Supplementary Fig. 52 – 54. Summaries of the corresponding integral parameters derived from 2D EXSY experiments and the calculated exchange rates (k, s^{-1}) are shown in Supplementary Table 8 and 9 for Na^{+}·(**3**–H)$^{-}$ and K^{+}·(**3**–H)$^{-}$, respectively. The corresponding Eyring plots are shown in Supplementary Fig. 51 and 55. The obtained kinetic parameters, including the rotational speed of (k_{rot}, s^{-1}) of the rotor systems, are shown in Supplementary Table 6 in this Supplementary Information and Table 1 in the main text.

It is worth noting that, in all above cases, there were multiple species presented in the rotor systems. These included deprotonated **3** ((**3**–H)$^{-}$), metal cations, protonated DBU (DBU-H^{+}), and perchlorate anions (ClO_4^{-}). Despite that, in theory, DBU-H^{+} is sterically hindered thus hardly accelerating the rotation of (**3**–H)$^{-}$, we still designed and carried out a series of parallel experiments on metal-cation-regulated rotor systems, in which the (Li/Na/K)$^{+}$·(**3**–H)$^{-}$ samples were prepared directly by mixing **3** with alkali metal *tert*-butoxides. By this way, the influence of DBU-H^{+} on the rotation of the rotor could be completely ruled out. Considering the alkali metal *tert*-butoxides is not soluble in acetonitrile-d_3, the samples for the investigations were prepared by mixing **3** with equivalent lithium tert-butoxide or excess sodium or potassium tert-butoxides powder, followed the addition of

acetonitrile-d_3 to give finally homogeneous solutions of **3**-*t*BuOM (M = Li^+, Na^+ or K^+) (4 mM, 0.5 mL) in acetonitrile-d_3 with some precipitates of alkali metal *tert*-butoxides at the bottom of the tube in the cases of Na^+ or K^+. Integrating of ^{1}H NMR signals confirmed there were only one equivalent of alkali metal cations presented in the rotor solution systems. Next, VT ^{1}H NMR or 2D EXSY experiments were carrried out. All the investigations were totally repeated for three times with three independent samples. Example of the obtained VT ^{1}H NMR spectra for $Li^+\cdot$(**3**–H)$^-$ is shown in Supplementary Fig. 56. Example of the obtained 2D EXSY NMR spectra for $Na^+\cdot$(**3**–H)$^-$ and $K^+\cdot$(**3**–H)$^-$ are shown in Supplementary Fig. 59 – 63 and Supplementary Fig. 65 – 67, respectively. Summaries of the corresponding integral parameters derived from 2D EXSY experiments and the calculated exchange rates (k, s^{-1}) are shown in Supplementary Table 10 and 11 for $Na^+\cdot$(**3**–H)$^-$ and $K^+\cdot$(**3**–H)$^-$, respectively. The corresponding Eyring plots are shown in Supplementary Fig. 58, 64 and 68. The obtained kinetic parameters are shown in Supplementary Table 5 – 6 in this Supplementary Information and Table 1 in the main text.

For comparison, we also measured the activation parameters of DBU-deprotonated **3** in the presence of only one equivalent of of alkali metal cations. The obtained VT ^{1}H NMR spectra for $Li^+\cdot$(**3**–H)$^-$ is shown in Supplementary Fig. 69. Example of the obtained 2D EXSY NMR spectra for $Na^+\cdot$(**3**–H)$^-$ and $K^+\cdot$(**3**–H)$^-$ are shown in Supplementary Fig. 72 – 75 and Supplementary Fig. 77 – 79, respectively. Summaries of the corresponding integral parameters derived from 2D EXSY experiments and the calculated exchange rates (k, s^{-1}) are shown in Supplementary Table 12 and 13 for $Na^+\cdot$(**3**–H)$^-$ and $K^+\cdot$(**3**–H)$^-$, respectively. The corresponding Eyring plots are shown in Supplementary Fig. 71, 76 and 80. The obtained kinetic parameters are shown in Supplementary Table 5 – 6, SI and Table 1 in the main text.

To further explore the dependence of the motion of metal-cation-regulated system on the amount of metal cations in the systems, we supplemented a series of VT ^{1}H NMR investigations on samples of **3** (4 mM in acetonitrile-d_3) in the presence of 1.2 equivalents of DBU and $LiClO_4$ with varying amounts (Specifically, 4.0, 0.8, 0.6, 0.4 and 0.2 equivalents). The obtained experimental and simulated spectra at the region of methyl protons as well as the corresponding Eyring plots are shown or re-shown in Supplementary Fig. 81 – 94. Similar titration experiments were also taken for $NaClO_4$ and $KClO_4$. The obtained 2D EXSY spectra as well as the corresponding Eyring plots are shown or re-shown in Supplementary Fig. 95 – 122. Summary of the thermodynamic parameters is shown in

Supplementary Table 5 and Table 6. The plots of the calculated apparent free energy of activation ($\Delta G^{\ddagger}$) at 338 K and 298 K for the rotation of rotor system versus the amount (equivalents) of alkali metal cations presented in the solution is shown in Fig. 4b in the main text and Supplementary Fig. 123.

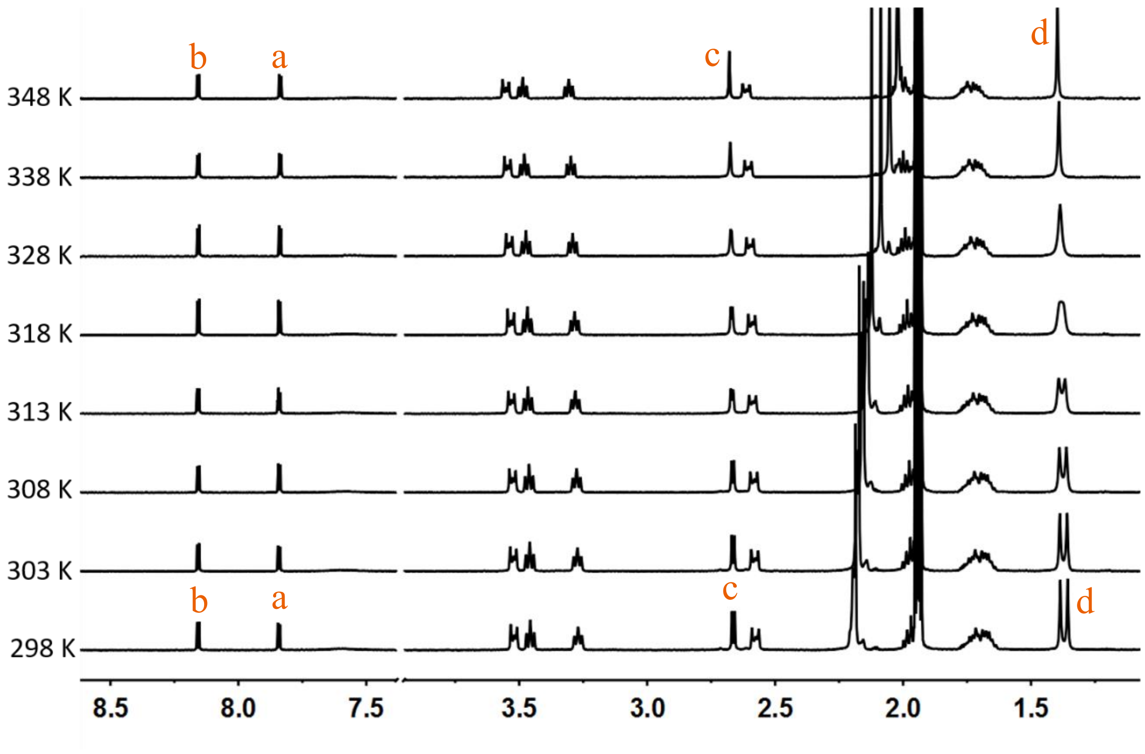

Supplementary Figure 44. VT ^{1}H NMR spectra (500 MHz, CD_3CN) of **3-DBU-LiClO$_4$**. [**3**] = 4 mM, [**DBU**] = 4.8 mM (1.2 equiv), [**LiClO$_4$**] = 8 mM (2.0 equiv).

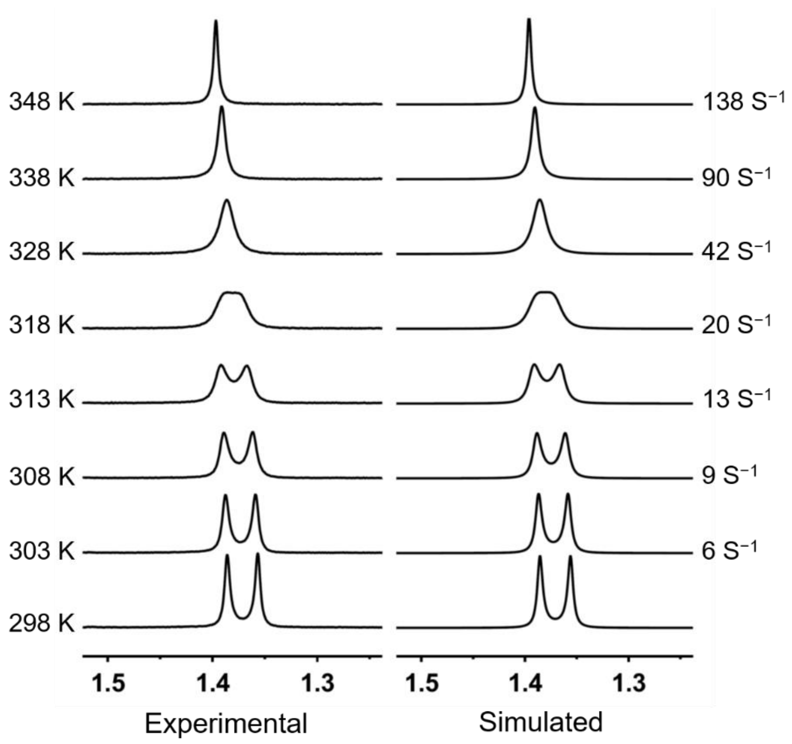

Supplementary Figure 45. Experimental and simulated VT ^{1}H NMR spectra (500 MHz, CD_3CN) of methyl peaks of **3-DBU-LiClO$_4$**; [**3**] = 4 mM, [**DBU**] = 4.8 mM (1.2 equiv), [**LiClO$_4$**] = 8 mM (2.0 equiv). The temperature (K) and calculated interconversion rate constants (k_r, s^{-1}) are given for each trace.

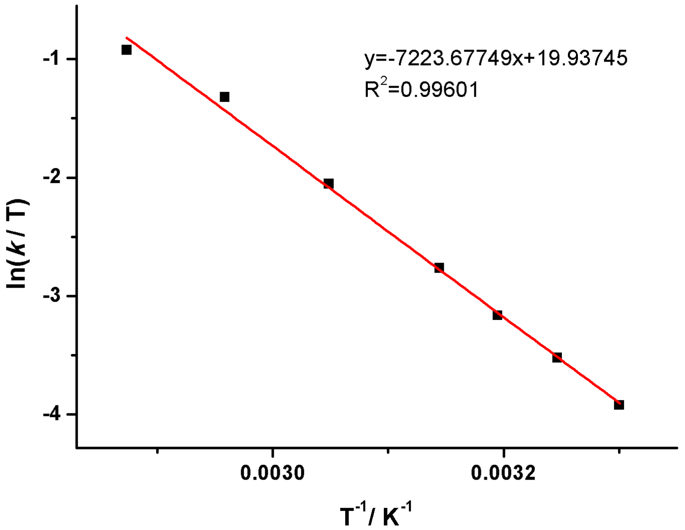

Supplementary Figure 46. Eyring plot of the rates of exchange obtained from line width analysis of methyl peaks signal in VT ^{1}H NMR spectra of **3-DBU-2.0eq LiClO$_4$** in acetonitrile-d_3.

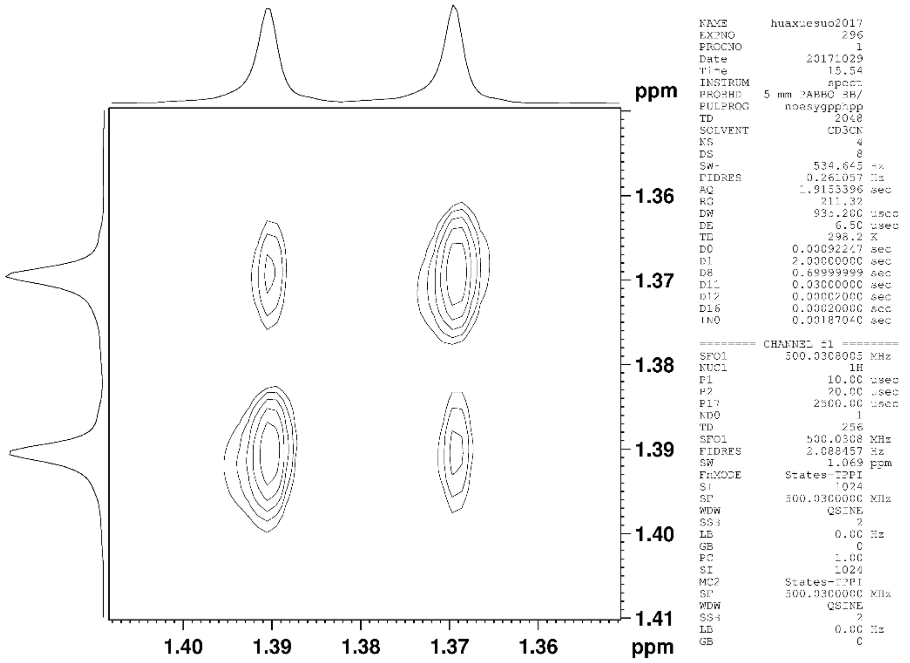

Supplementary Figure 47. EXSY NMR (500 MHz, t_m = 0.7 s) spectrum of **3-DBU-NaClO$_4$** at 298 K in CD_3CN. [**3**] = 4 mM, [**DBU**] = 4.8 mM (1.2 equiv), [**NaClO$_4$**] = 8 mM (2.0 equiv).

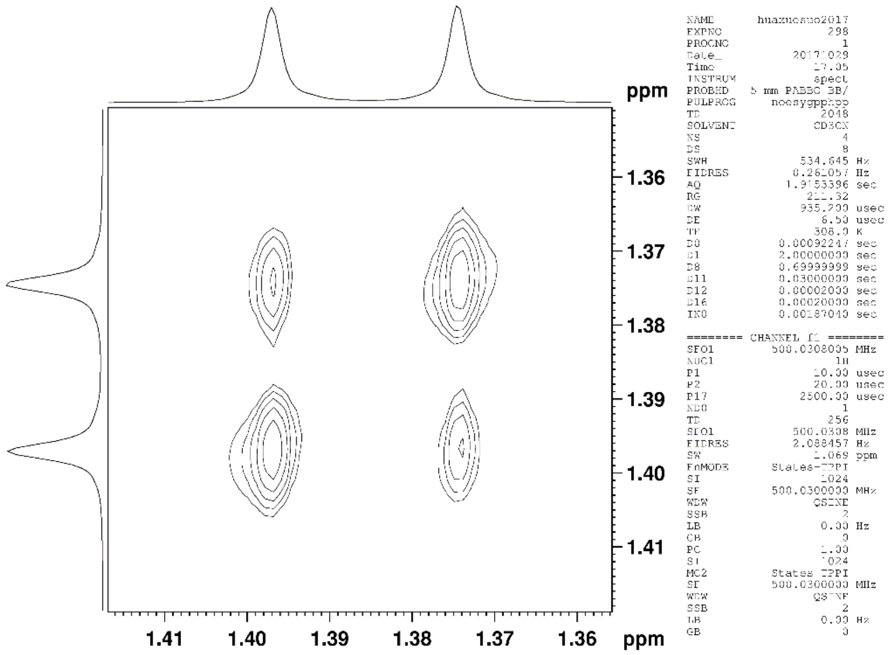

Supplementary Figure 48. EXSY NMR (500 MHz, t_m = 0.7 s) spectrum of **3-DBU-NaClO$_4$** at 308 K in CD_3CN. [**3**] = 4 mM, [**DBU**] = 4.8 mM (1.2 equiv), [**NaClO$_4$**] = 8 mM (2.0 equiv).

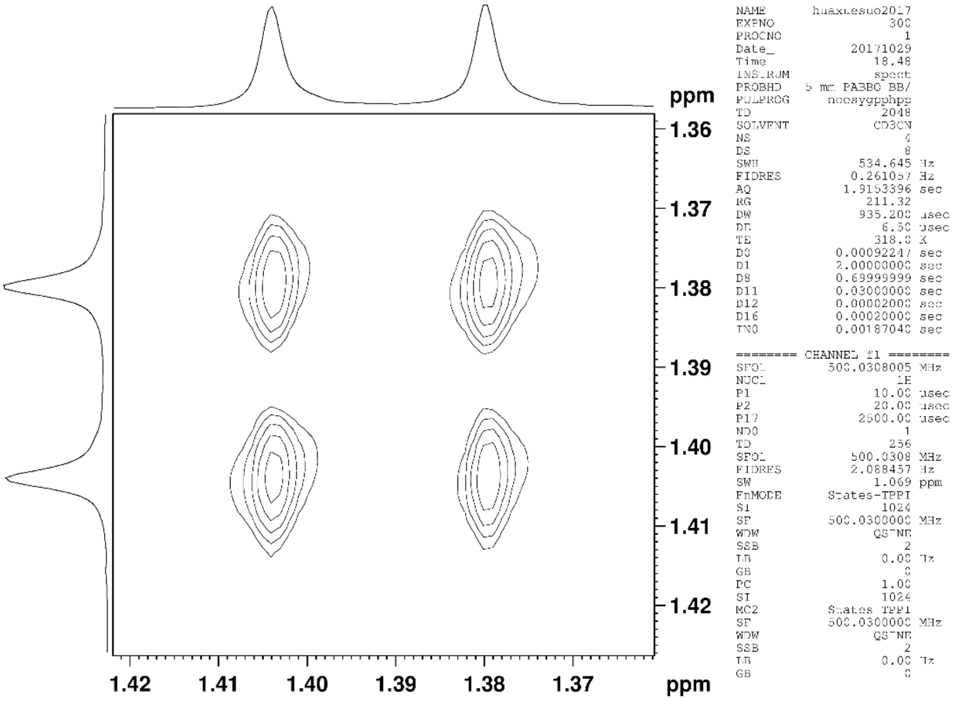

Supplementary Figure 49. EXSY NMR (500 MHz, t_m= 0.7 s) spectrum of **3-DBU-NaClO₄** at 318 K in CD_3CN. [**3**] = 4 mM, [**DBU**] = 4.8 mM (1.2 equiv), [**$NaClO_4$**] = 8 mM (2.0 equiv).

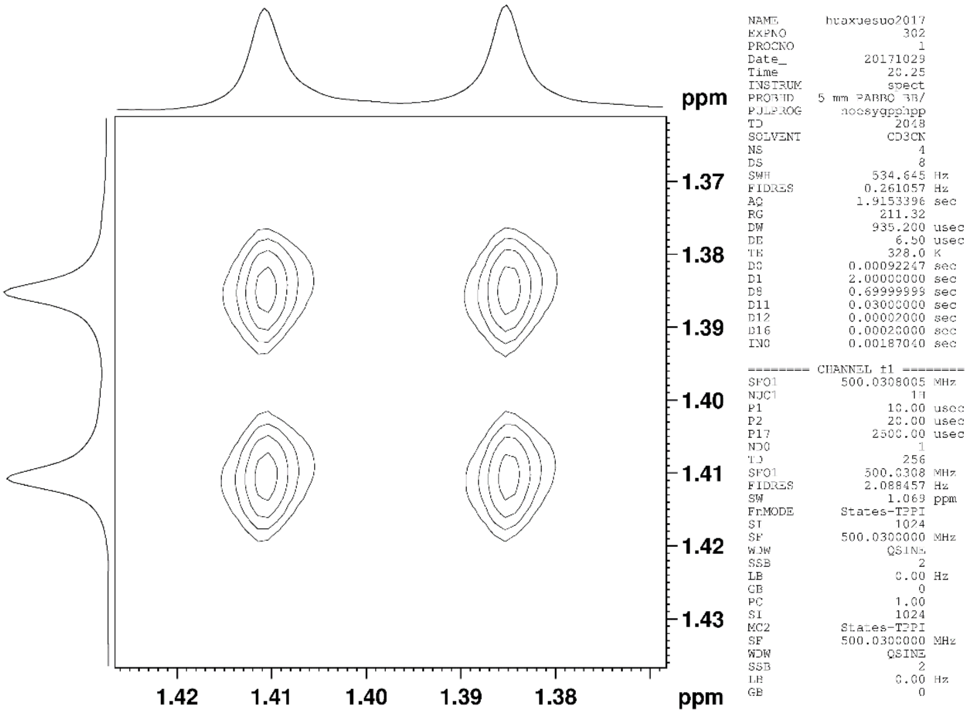

Supplementary Figure 50. EXSY NMR (500 MHz, t_m = 0.7 s) spectrum of **3-DBU-$NaClO_4$** at 328 K in CD_3CN. [**3**] = 4 mM, [**DBU**] = 4.8 mM (1.2 equiv), [**$NaClO_4$**] = 8 mM (2.0 equiv).

Supplementary Table 8. The integral parameters from 2D EXSY NMR and calculated *k* values of **3-DBU-2.0eq NaClO$_4$**.

T(K)	I_A	I_B	I_{AB}	I_{BA}	k (s^{-1})	$\Delta G^{\#}$ (kcal mol^{-1})
**298**	1.0000	0.9881	0.1734	0.1916	0.53	17.78
**308**	1.0000	0.9878	0.4493	0.4641	1.42	17.87
**318**	1.0000	0.9953	0.7695	0.7787	2.96	17.96
**328**	1.0000	1.0187	0.9687	0.9954	6.13	18.05

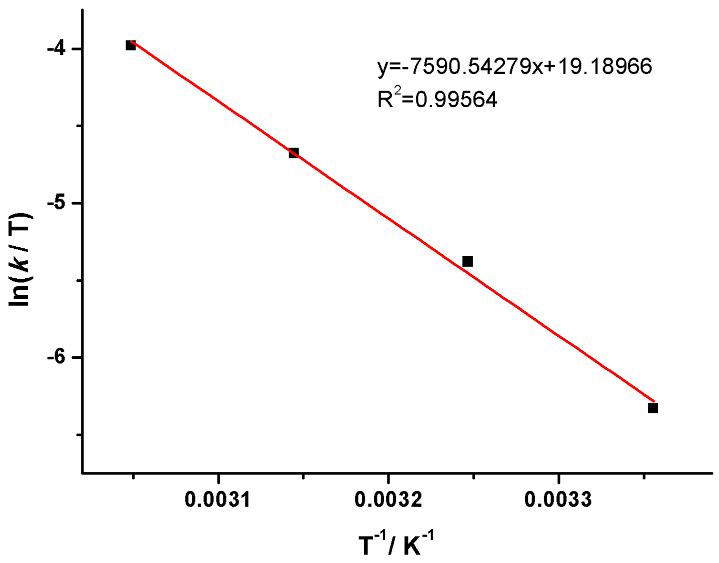

Supplementary Figure 51. Eyring plot of the rates of exchange between two diastereotopic methyl protons derived from VT 2D EXSY (from 308 K to 338 K) experiments on **3-DBU-2.0eq NaClO$_4$** in acetonitrile-d_3.

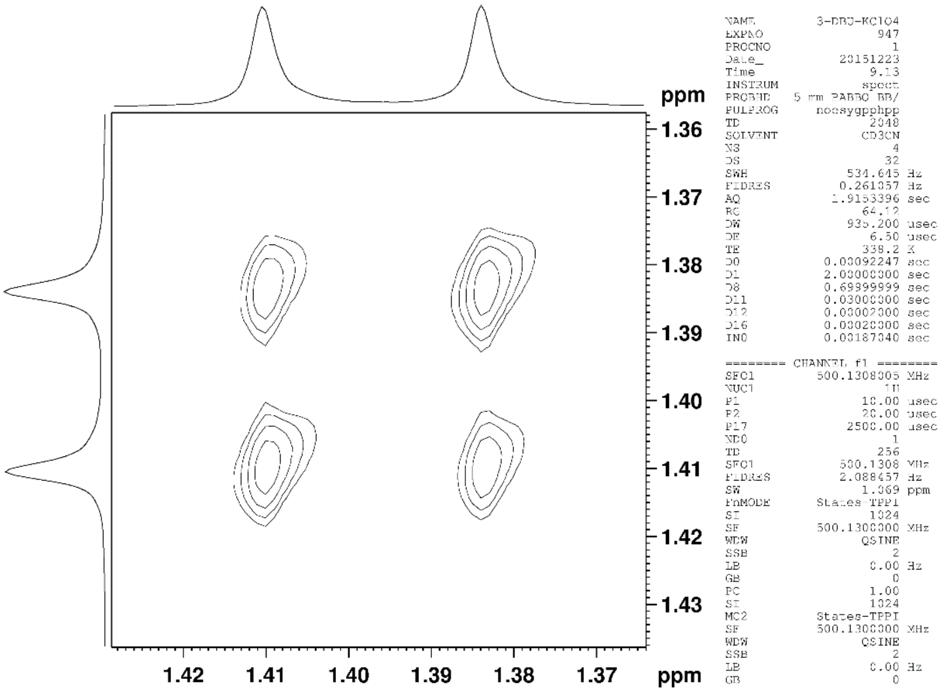

Supplementary Figure 52. EXSY NMR (500 MHz, t_m= 0.7 s) spectrum of **3-DBU-KClO$_4$** at 338 K in CD_3CN. [**3**] = 4 mM, [**DBU**] = 4.8 mM (1.2 equiv), [**KClO$_4$**] = 8 mM (2.0 equiv).

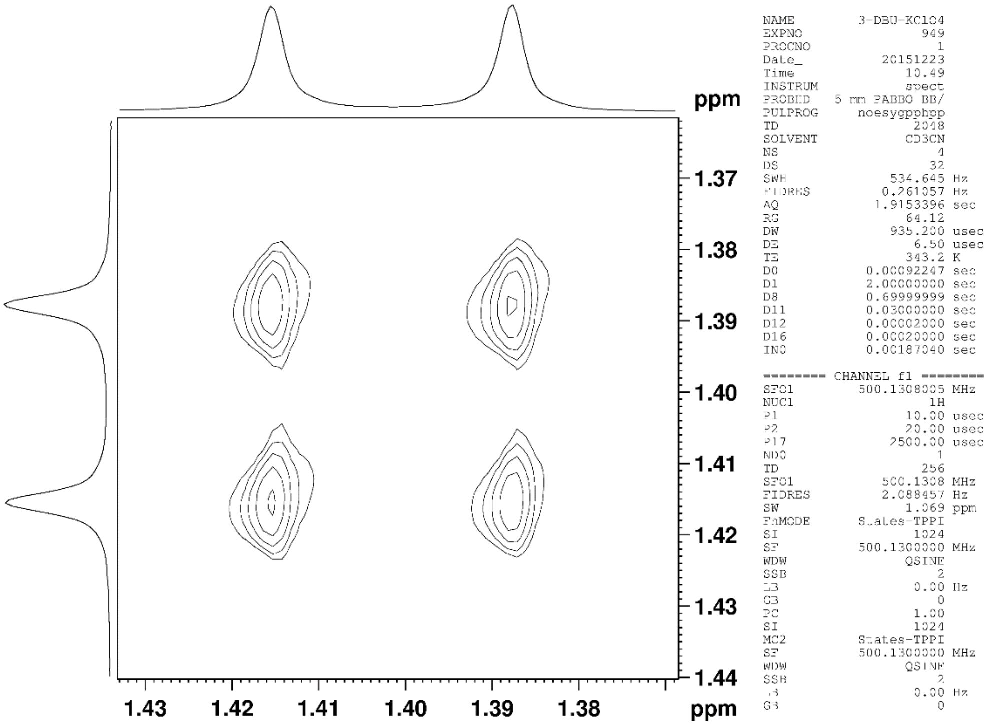

Supplementary Figure 53. EXSY NMR (500 MHz, t_m= 0.7 s) spectrum of **3-DBU-KClO$_4$** at 343 K in CD_3CN. [**3**] = 4 mM, [**DBU**] = 4.8 mM (1.2 equiv), [**KClO$_4$**] = 8 mM (2.0 equiv).

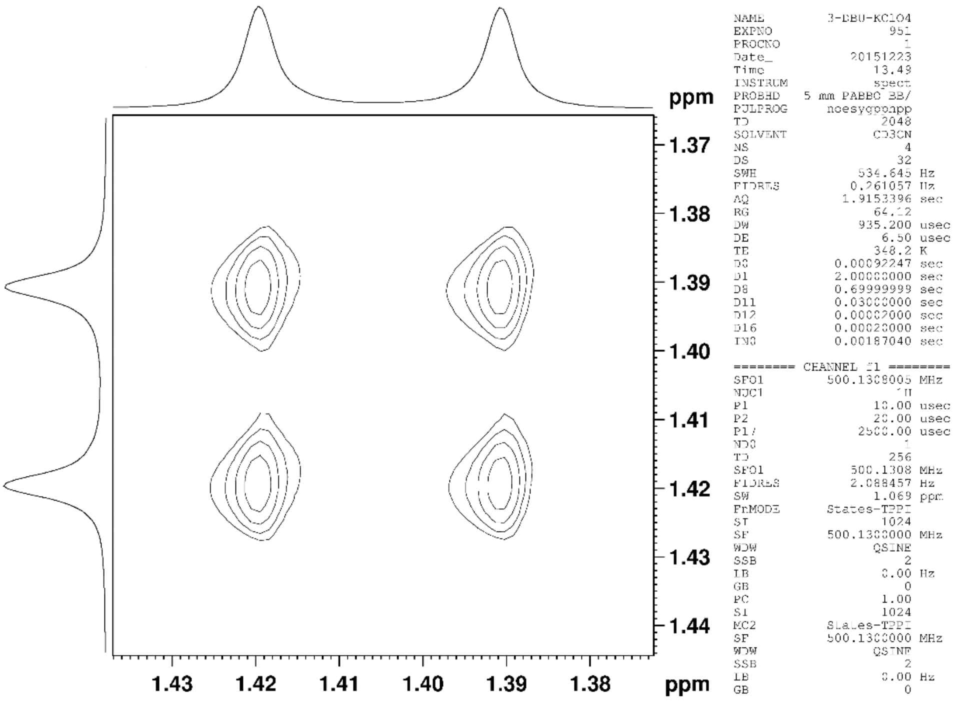

Supplementary Figure 54. EXSY NMR (500 MHz, t_m= 0.7 s) spectrum of **3-DBU-KClO$_4$** at 348 K in CD_3CN. [**3**] = 4 mM, [**DBU**] = 4.8 mM (1.2 equiv), [**KClO$_4$**] = 8 mM (2.0 equiv).

Supplementary Table 9. The integral parameters from 2D EXSY NMR and calculated k values of **3-DBU-2.0eq KClO$_4$**.

T(K)	I_A	I_B	I_{AB}	I_{BA}	k (s^{-1})	$\Delta G^{\#}$ (kcal mol^{-1})
**338**	1.0000	0.9938	0.6228	0.6267	2.1	19.36
**343**	1.0000	0.9637	0.8209	0.8503	3.6	19.29
**348**	1.0000	0.9640	0.9375	0.9680	6.0	19.23

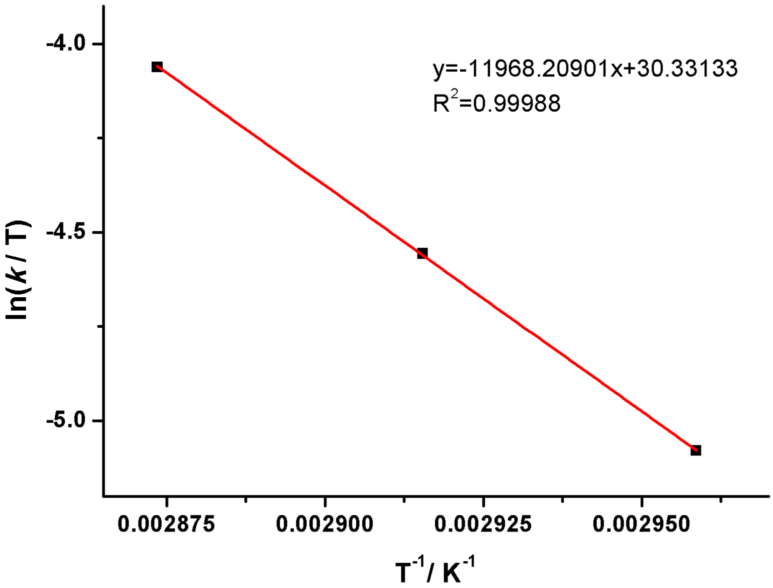

Supplementary Figure 55. Eyring plot of the rates of exchange between two diastereotopic methyl protons derived from VT 2D EXSY (from 338 K to 348 K) experiments on **3-DBU-2.0eq $KClO_4$** in acetonitrile-*d_3*.

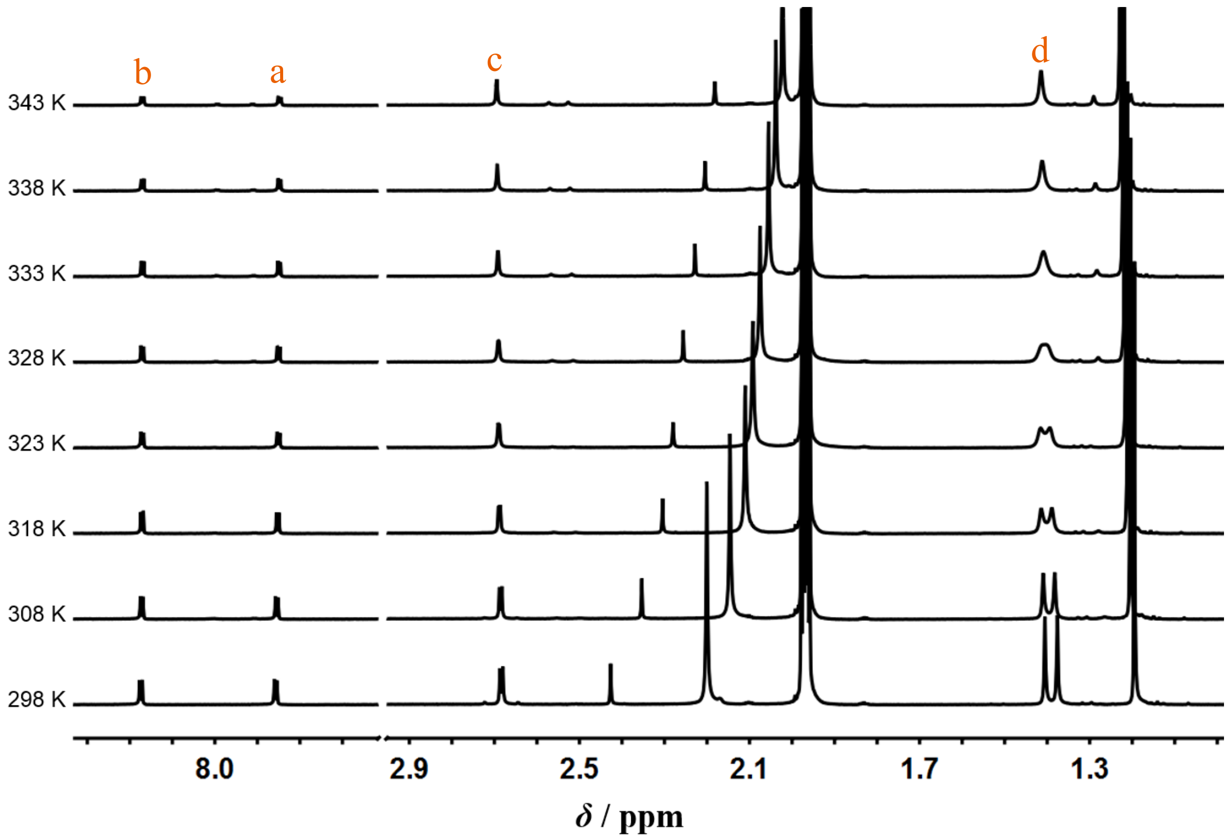

Supplementary Figure 56. VT ^{1}H NMR spectra (500 MHz, CD_3CN) of **3-*t*BuOLi** (4 mM).

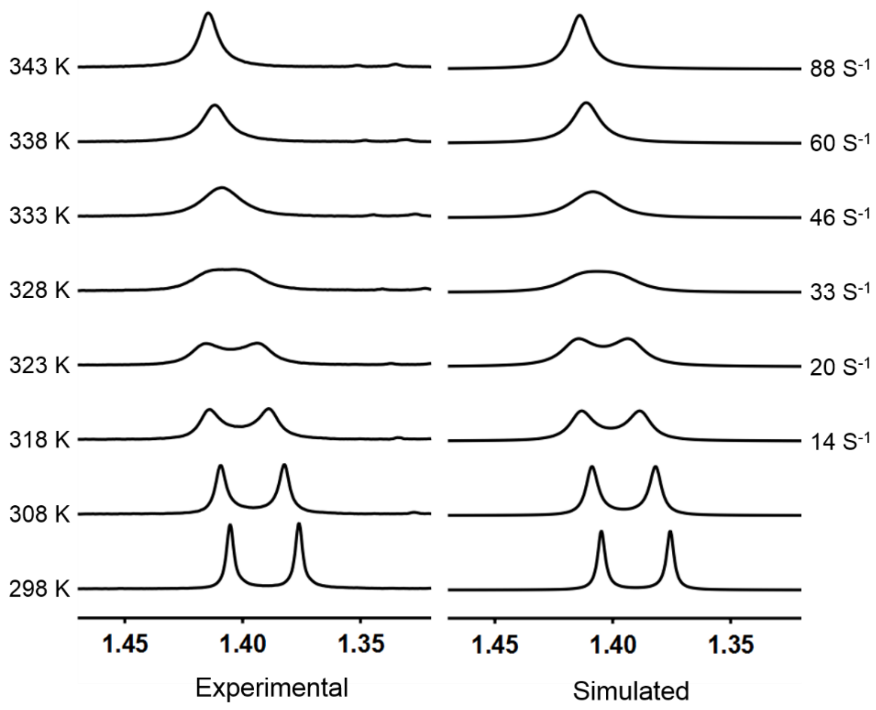

Supplementary Figure 57. Experimental and simulated VT ^{1}H NMR spectra (500 MHz, CD_3CN, 4 mM) of methyl peaks of **3-*t*BuOLi**. The temperature (K) and calculated interconversion rate constants (k_r, s^{-1}) are given for each trace.

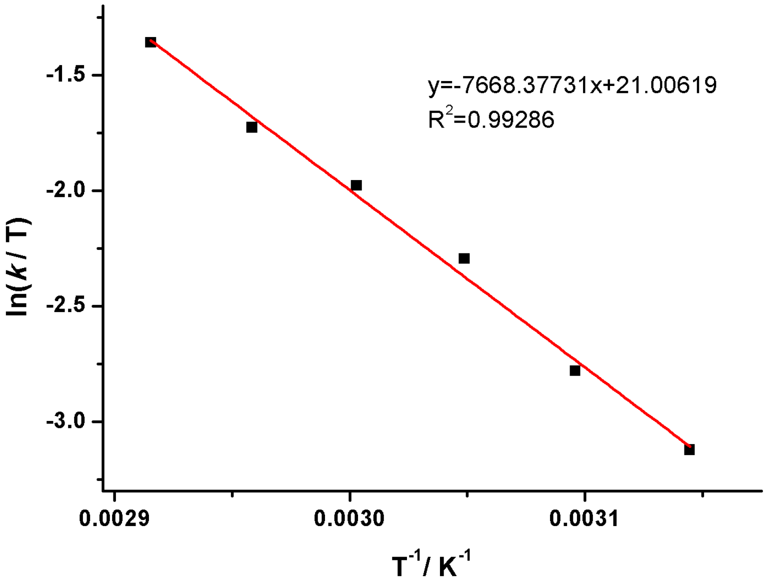

Supplementary Figure 58. Eyring plot of the rates of exchange obtained from line width analysis of methyl signals on VT ^{1}H NMR spectra of **3-*t*BuOLi** in acetonitrile-d_3.

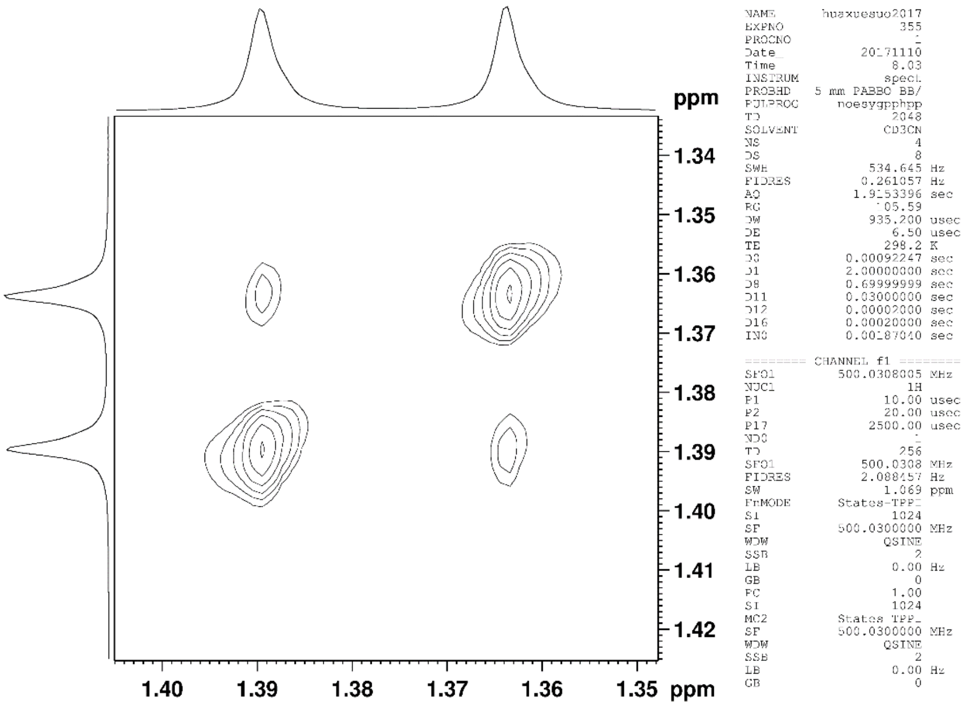

Supplementary Figure 59. EXSY NMR (500 MHz, t_m= 0.7 s) spectrum of **3-*t*BuONa** (4 mM) at 298 K in CD_3CN.

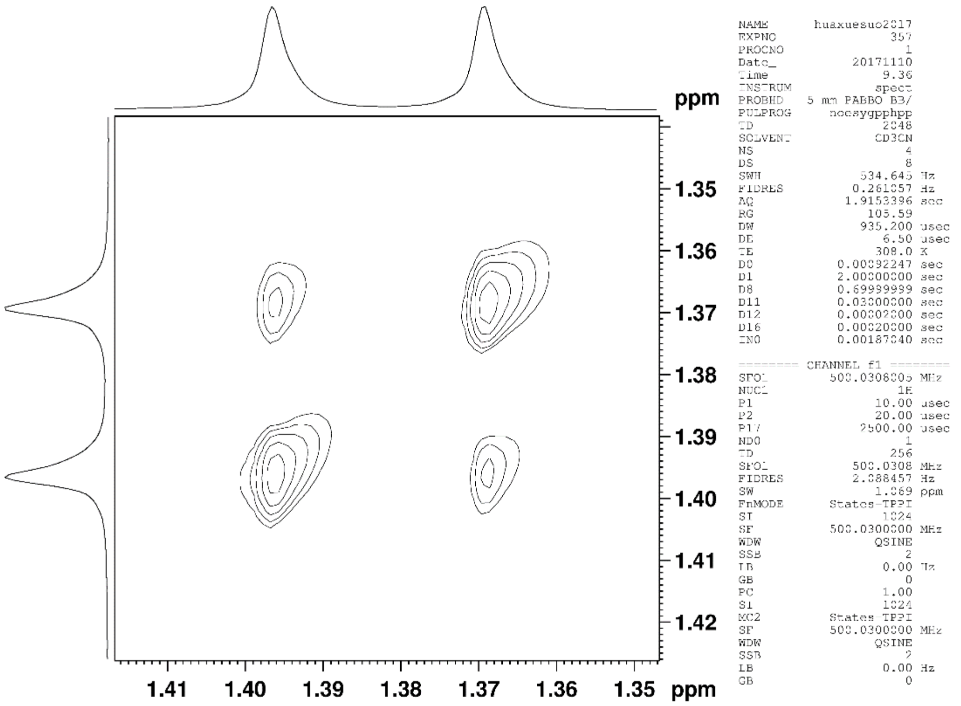

Supplementary Figure 60. EXSY NMR (500 MHz, t_m= 0.7 s) spectrum of **3-*t*BuONa** (4 mM) at 308 K in CD_3CN.

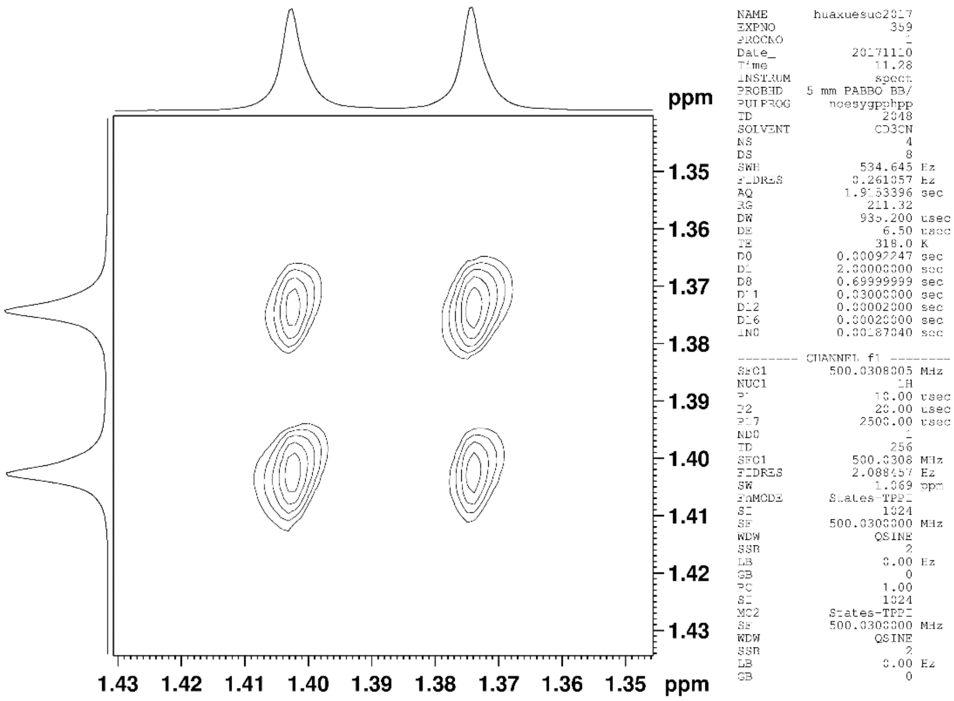

Supplementary Figure 61. EXSY NMR (500 MHz, t_m= 0.7 s) spectrum of **3-*t*BuONa** (4 mM) at 318 K in CD_3CN.

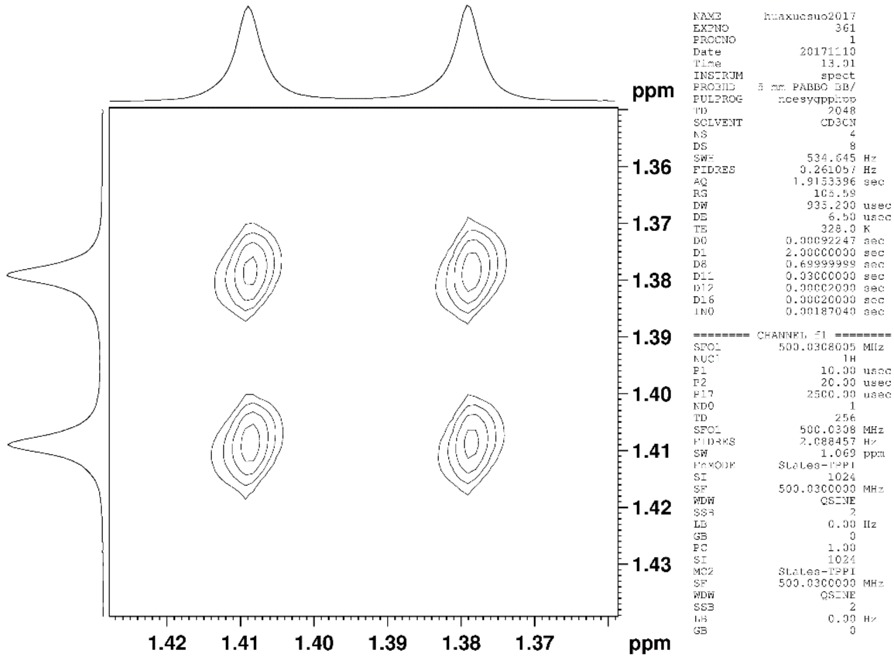

Supplementary Figure 62. EXSY NMR (500 MHz, t_m= 0.7 s) spectrum of **3-*t*BuONa** (4 mM) at 328 K in CD_3CN.

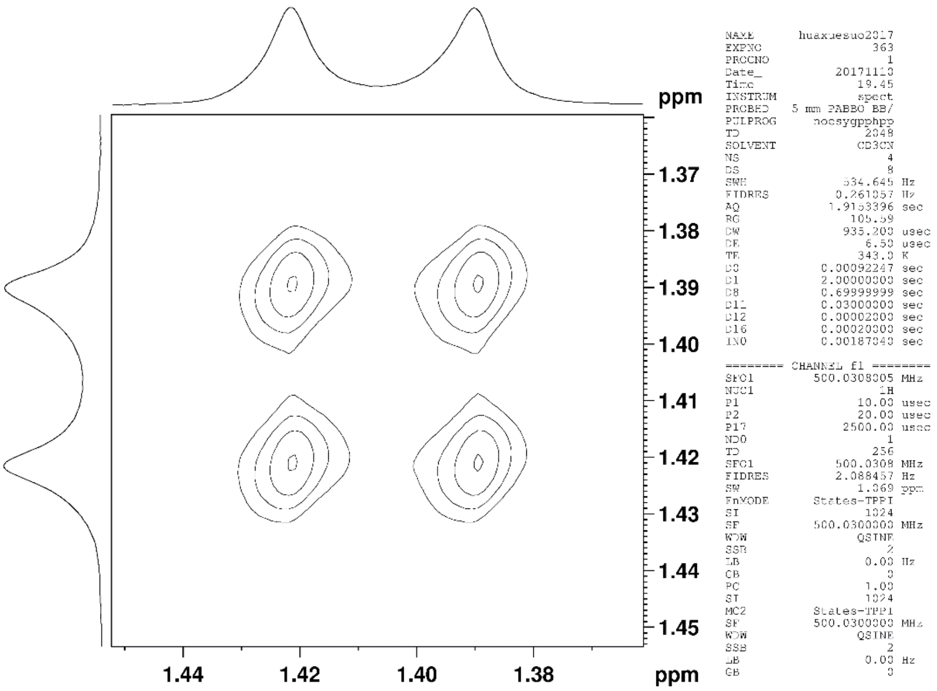

Supplementary Figure 63. EXSY NMR (500 MHz, t_m= 0.7 s) spectrum of **3-*t*BuONa** (4 mM) at 338 K in CD_3CN.

Supplementary Table 10. The integral parameters from 2D EXSY NMR and calculated *k* values of **3-*t*BuONa**.

T(K)	I_A	I_B	I_{AB}	I_{BA}	*k* (s^{-1})
**298**	1.0000	1.005	0.15594	0.1417	0.43
**308**	1.0000	0.9844	0.2757	0.2917	0.84
**318**	1.0000	1.0457	0.5673	0.5925	1.84
**328**	1.0000	0.9951	0.8682	0.8635	3.79
**338**	1.0000	0.99739	0.99034	0.99583	8.39

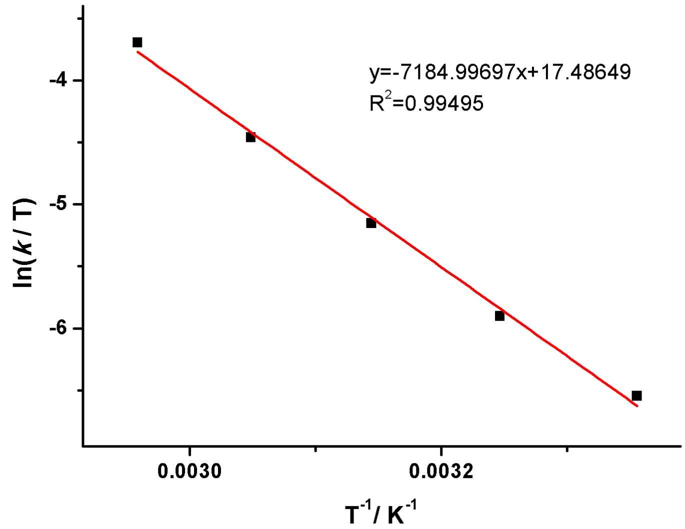

Supplementary Figure 64. Eyring plot of the rates of exchange between two diastereotopic methyl protons derived from VT 2D EXSY (from 298 K to 338 K) experiments on **3-*t*BuONa** in acetonitrile-d_3.

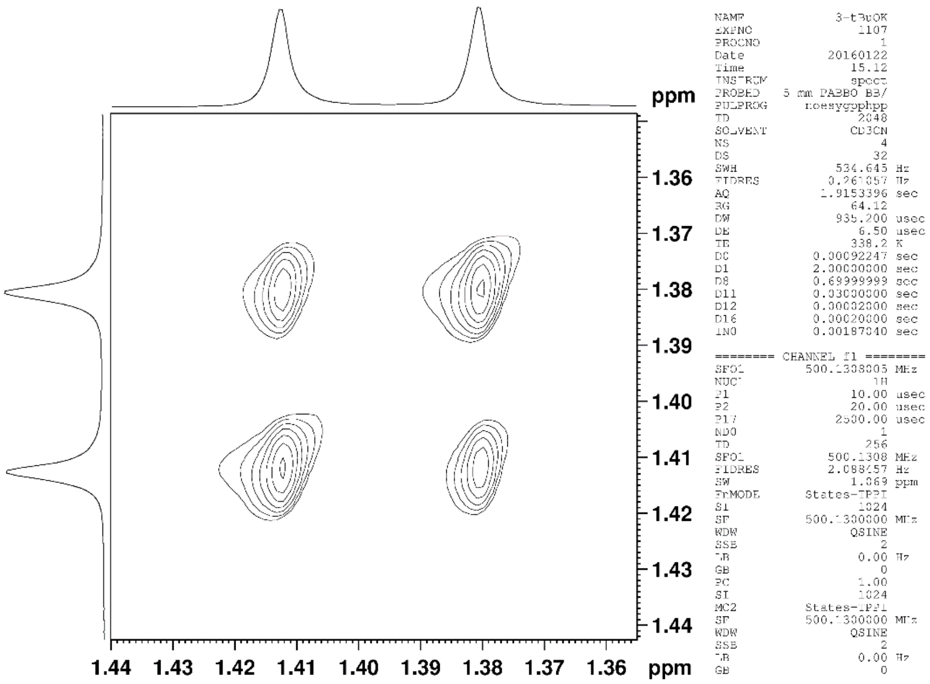

Supplementary Figure 65. EXSY NMR (500 MHz, t_m= 0.7 s) spectrum of **3-*t*BuOK** (4 mM) at 338 K in CD_3CN.

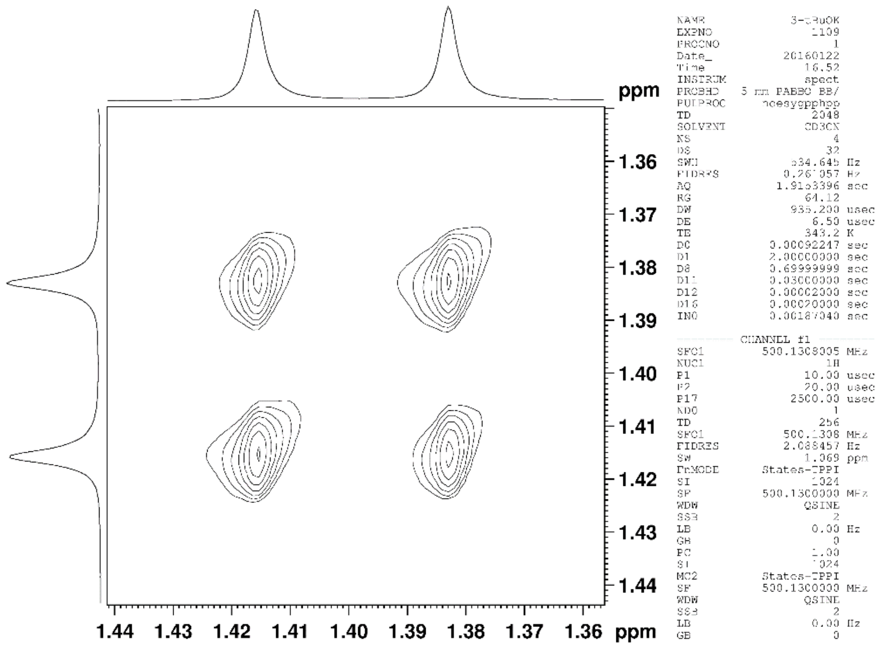

Supplementary Figure 66. EXSY NMR (500 MHz, t_m= 0.7 s) spectrum of **3-*t*BuOK** (4 mM) at 343 K in CD_3CN.

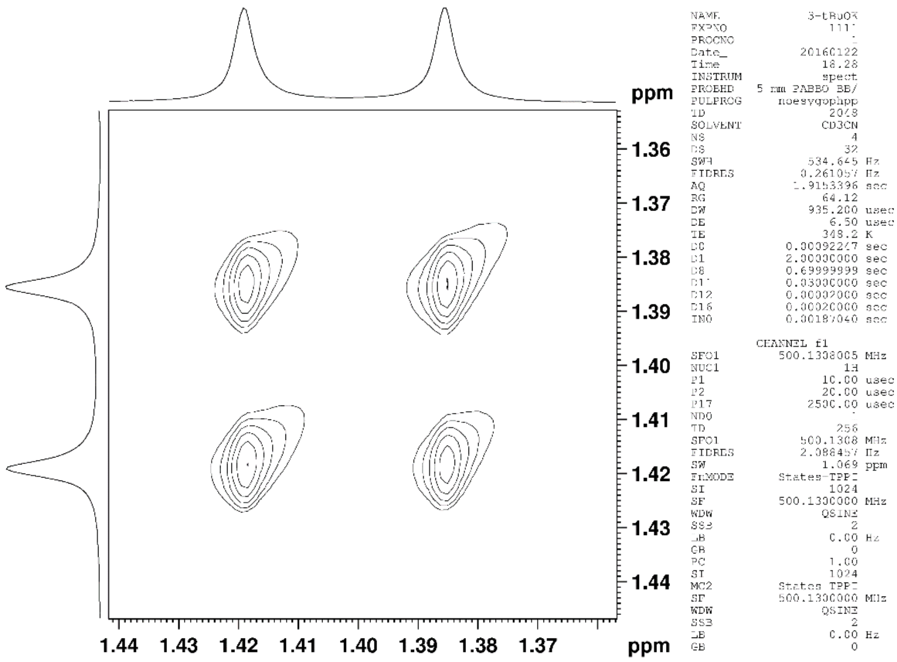

Supplementary Figure 67. EXSY NMR (500 MHz, t_m= 0.7 s) spectrum of **3-*t*BuOK** (4 mM) at 348 K in CD_3CN.

Supplementary Table 11. The integral parameters from 2D EXSY NMR and calculated *k* values of **3-*t*BuOK**.

T(K)	I_A	I_B	I_{AB}	I_{BA}	*k* (s^{-1})
**338**	1.0000	1.0037	0.63483	0.64646	2.16
**343**	1.0000	1.0041	0.83956	0.85186	3.53
**348**	1.0000	1.0084	0.96198	0.96306	5.51

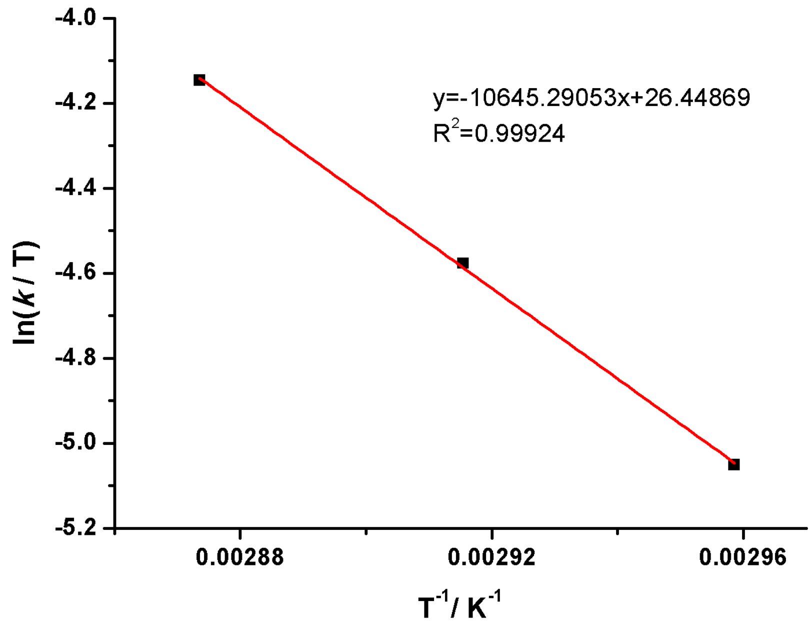

Supplementary Figure 68. Eyring plot of the rates of exchange between two diastereotopic methyl protons derived from VT 2D EXSY (from 338 K to 348 K) experiments on **3-*t*BuOK** in acetonitrile-*d*3.

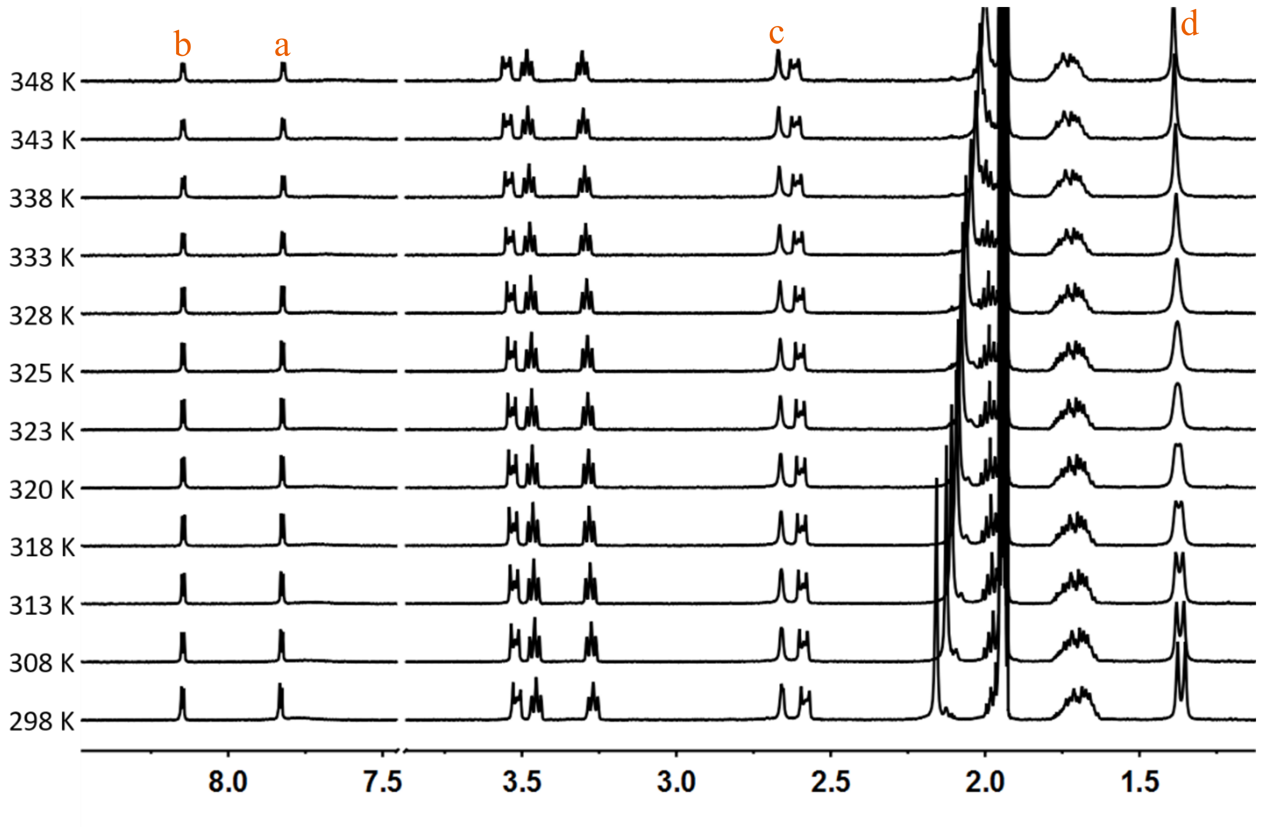

Supplementary Figure 69. VT ^{1}H NMR spectra (500 MHz, CD_3CN) of **3-DBU-LiClO$_4$**. [**3**] = 4 mM, [**DBU**] = 4.8 mM (1.2 equiv), [**LiClO$_4$**] = 4 mM (1.0 equiv).

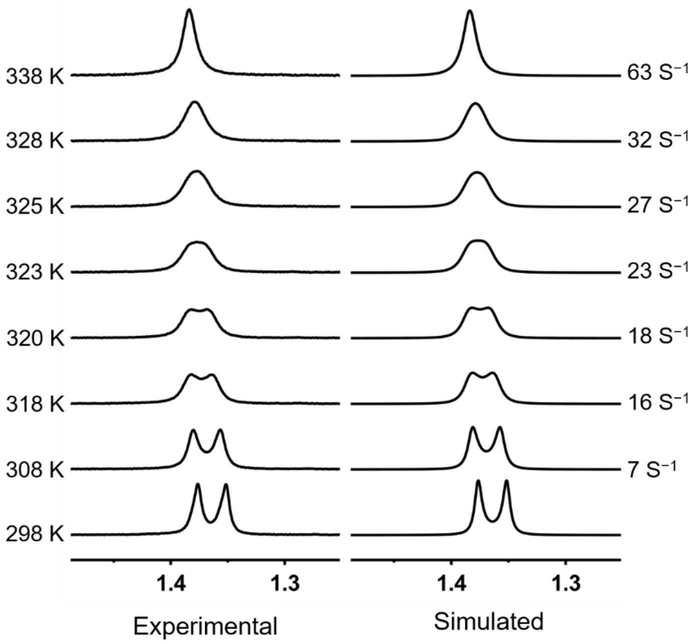

Supplementary Figure 70. Experimental and simulated VT ^{1}H NMR spectra (500 MHz, CD_3CN) of methyl peaks of **3-DBU-LiClO$_4$**; [**3**] = 4 mM, [**DBU**] = 4.8 mM (1.2 equiv), [**LiClO$_4$**] = 4 mM (1.0 equiv). The temperature (K) and calculated interconversion rate constants (k_r, s^{-1}) are given for each trace.

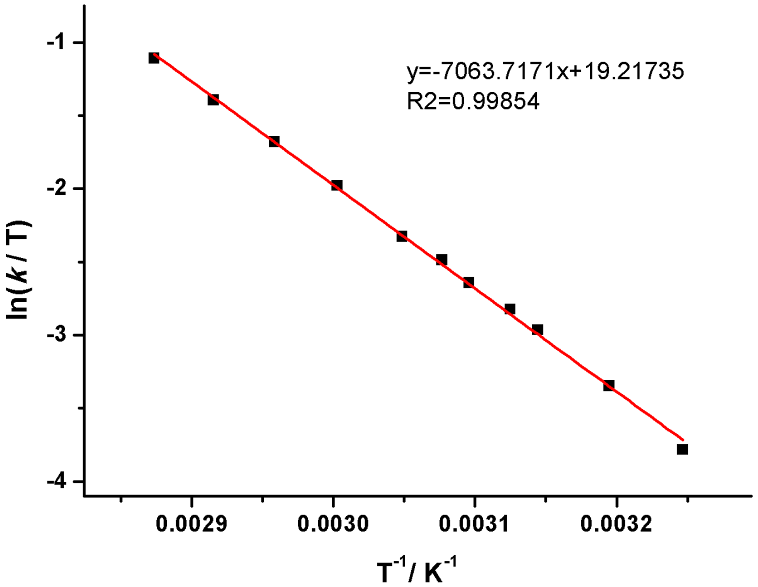

Supplementary Figure 71. Eyring plot of the rates of exchange obtained from line width analysis of methyl peaks signal in VT ^{1}H NMR spectra of **3-DBU-1.0eq LiClO$_4$** in acetonitrile-d_3.

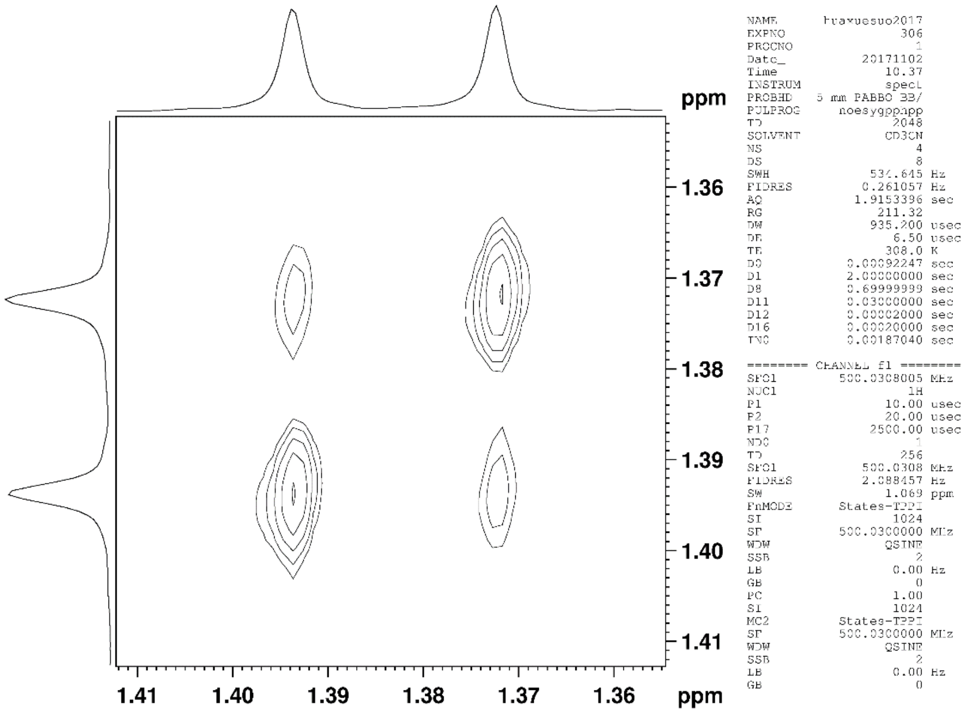

Supplementary Figure 72. Example of EXSY NMR (500 MHz, t_m = 0.7 s) spectrum of **3-DBU-NaClO$_4$** at 308 K in CD_3CN. [**3**] = 4 mM, [**DBU**] = 4.8 mM (1.2 equiv), [**NaClO$_4$**] = 4 mM (1.0 equiv).

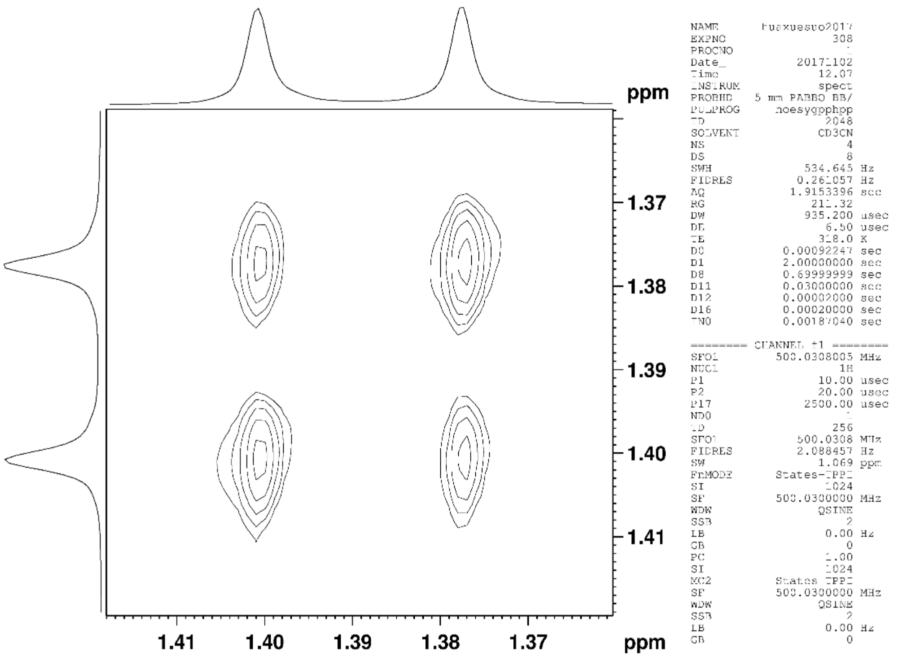

Supplementary Figure 73. Example of EXSY NMR (500 MHz, t_m = 0.7 s) spectrum of **3-DBU-NaClO4** at 318 K in CD_3CN. [**3**] = 4 mM, [**DBU**] = 4.8 mM (1.2 equiv), [**NaClO4**] = 4 mM (1.0 equiv).

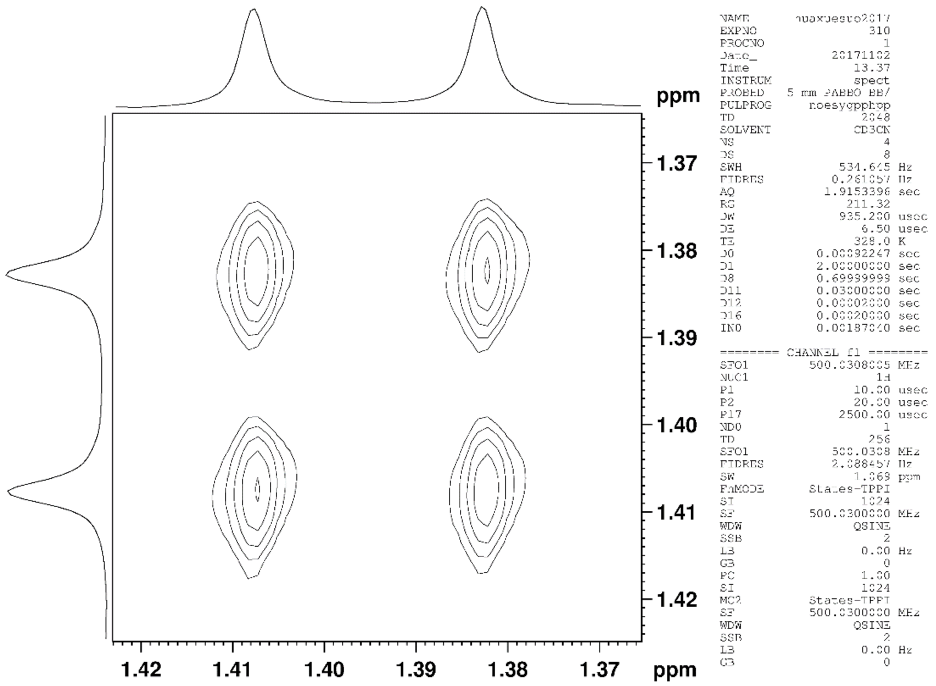

Supplementary Figure 74. Example of EXSY NMR (500 MHz, t_m = 0.7 s) spectrum of **3-DBU-NaClO4** at 328 K in CD_3CN. [**3**] = 4 mM, [**DBU**] = 4.8 mM (1.2 equiv), [**NaClO4**] = 4 mM (1.0 equiv).

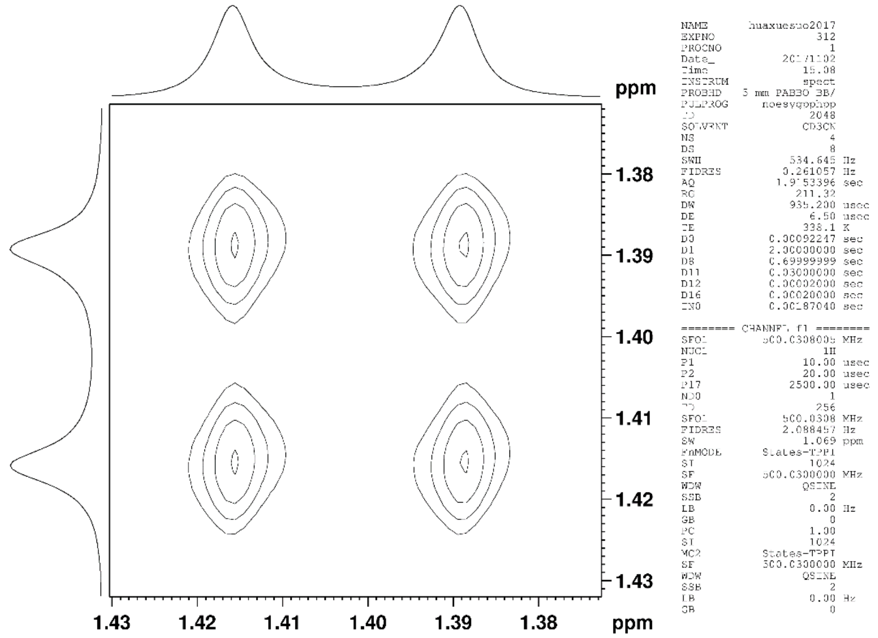

Supplementary Figure 75. Example of EXSY NMR (500 MHz, t_m = 0.7 s) spectrum of **3-DBU-NaClO$_4$** at 338 K in CD_3CN. [**3**] = 4 mM, [**DBU**] = 4.8 mM (1.2 equiv), [**NaClO$_4$**] = 4 mM (1.0 equiv).

Supplementary Table 12. The integral parameters from 2D EXSY NMR and calculated *k* values of **3-DBU-1.0eq NaClO$_4$**.

T(K)	I_A	I_B	I_{AB}	I_{BA}	*k* (s^{-1})
**308**	1.0000	0.9994	0.2777	0.2924	0.84
**318**	1.0000	1.0136	0.548	0.5592	1.77
**328**	1.0000	1.0209	0.8638	0.8971	3.82
**338**	1.0000	1.0013	0.9983	0.9948	8.84

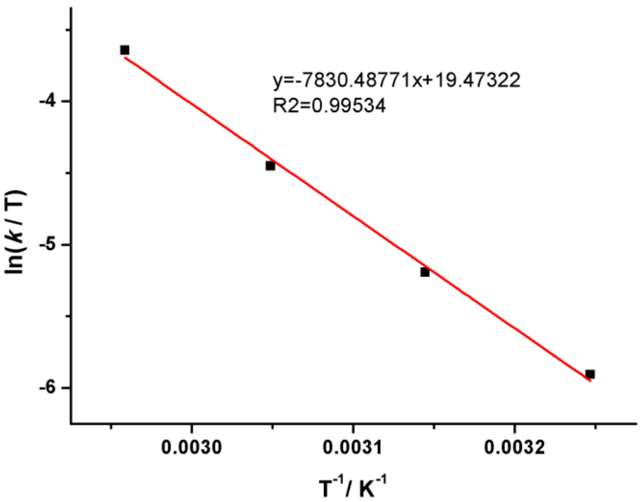

Supplementary Figure 76. Eyring plot of the rates of exchange between two diastereotopic methyl protons derived from VT 2D EXSY (from 308 K to 338 K) experiments on **3-DBU-1.0eq $NaClO_4$** in acetonitrile-d_3.

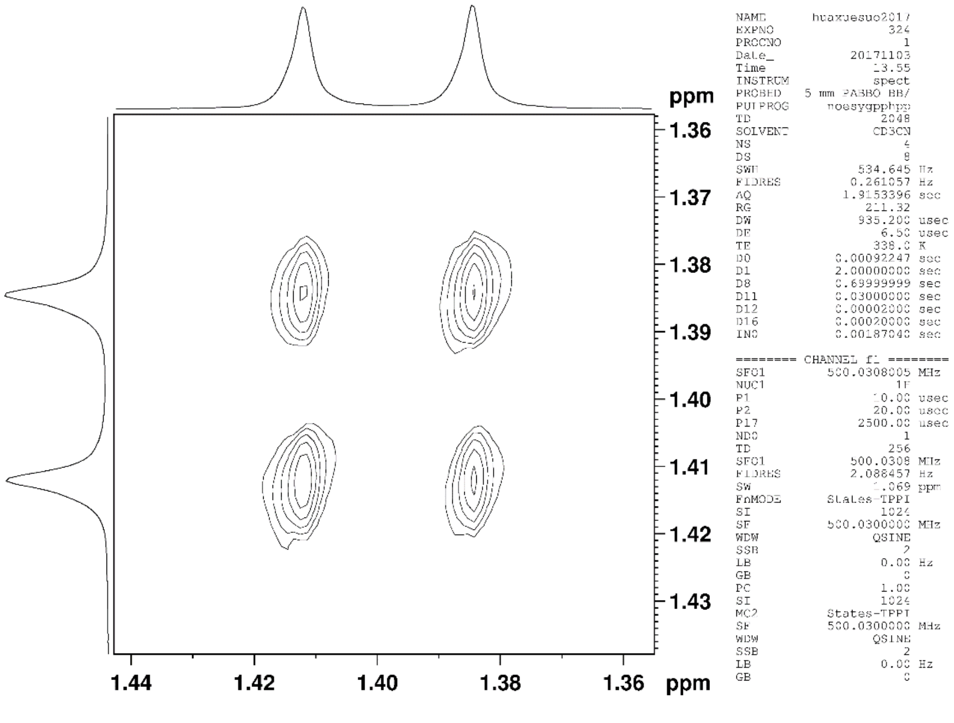

Supplementary Figure 77. Example of EXSY NMR (500 MHz, t_m = 0.7 s) spectrum of **3-DBU-$KClO_4$** at 338 K in CD_3CN. [**3**] = 4 mM, [**DBU**] = 4.8 mM (1.2 equiv), [**$KClO_4$**] = 4 mM (1.0 equiv).

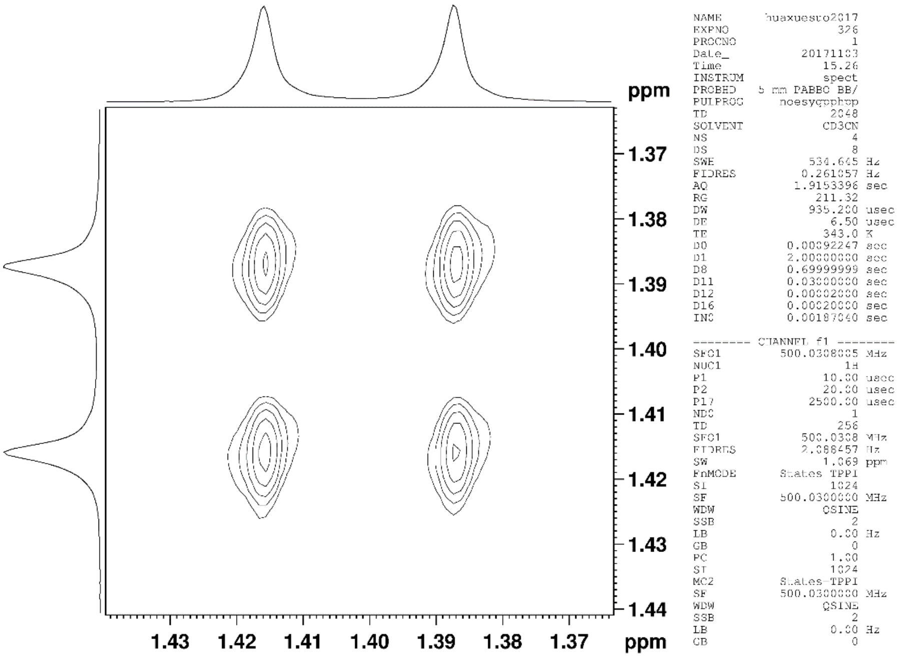

Supplementary Figure 78. Example of EXSY NMR (500 MHz, t_m = 0.7 s) spectrum of **3-DBU-KClO$_4$** at 343 K in CD_3CN. [**3**] = 4 mM, [**DBU**] = 4.8 mM (1.2 equiv), [**KClO$_4$**] = 4 mM (1.0 equiv).

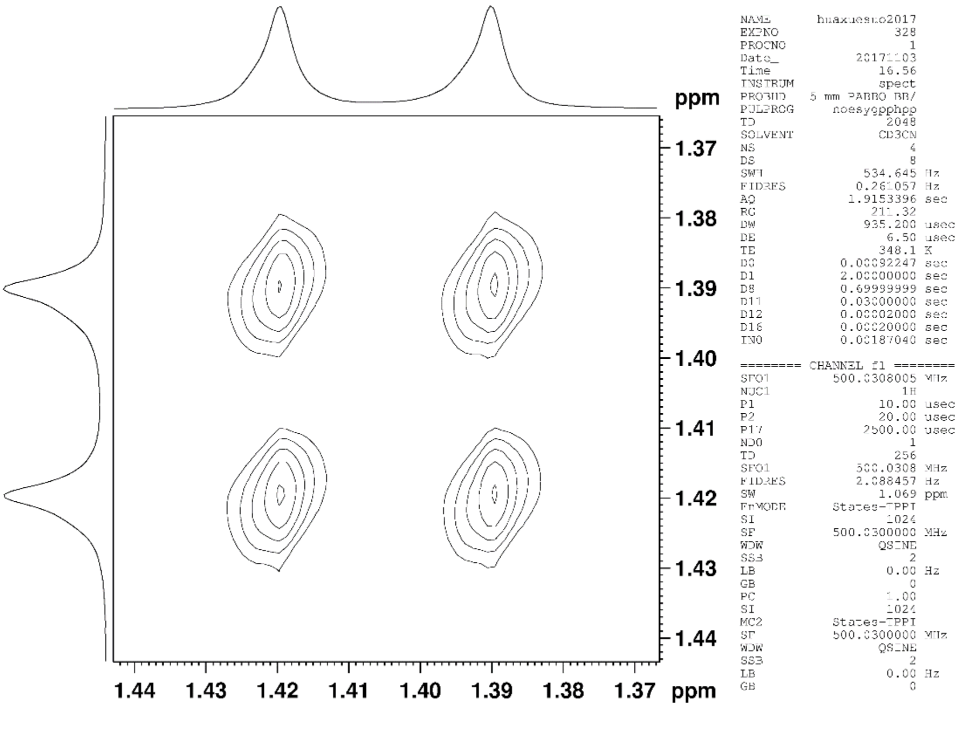

Supplementary Figure 79. Example of EXSY NMR (500 MHz, t_m = 0.7 s) spectrum of **3-DBU-KClO$_4$** at 348 K in CD_3CN. [**3**] = 4 mM, [**DBU**] = 4.8 mM (1.2 equiv), [**KClO$_4$**] = 4 mM (1.0 equiv).

Supplementary Table 13. The integral parameters from 2D EXSY NMR and calculated *k* values of **3-DBU-1.0eq $KClO_4$.**

T(K)	I_A	I_B	I_{AB}	I_{BA}	*k* (s^{-1})
**338**	1.0000	0.9994	0.6354	0.6325	2.14
**343**	1.0000	1.0086	0.8487	0.8326	3.46
**348**	1.0000	0.9961	0.9577	0.9600	5.59

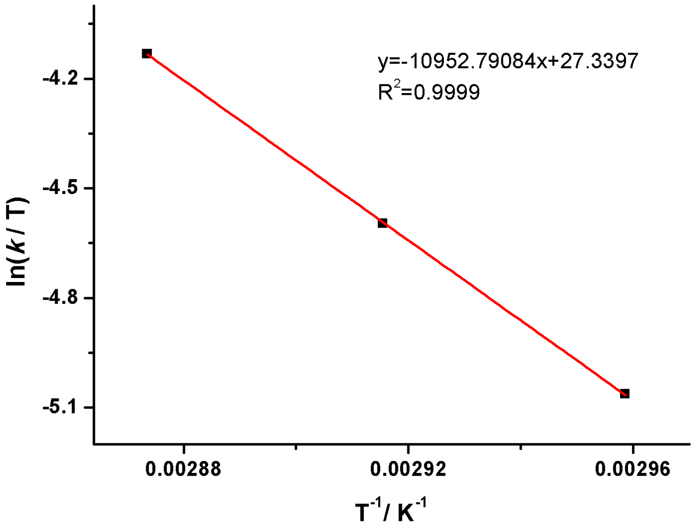

Supplementary Figure 80. Eyring plot of the rates of exchange between two diastereotopic methyl protons derived from VT 2D EXSY (from 338 K to 348 K) experiments on **3-DBU-1.0eq $KClO_4$** in acetonitrile-d_3.

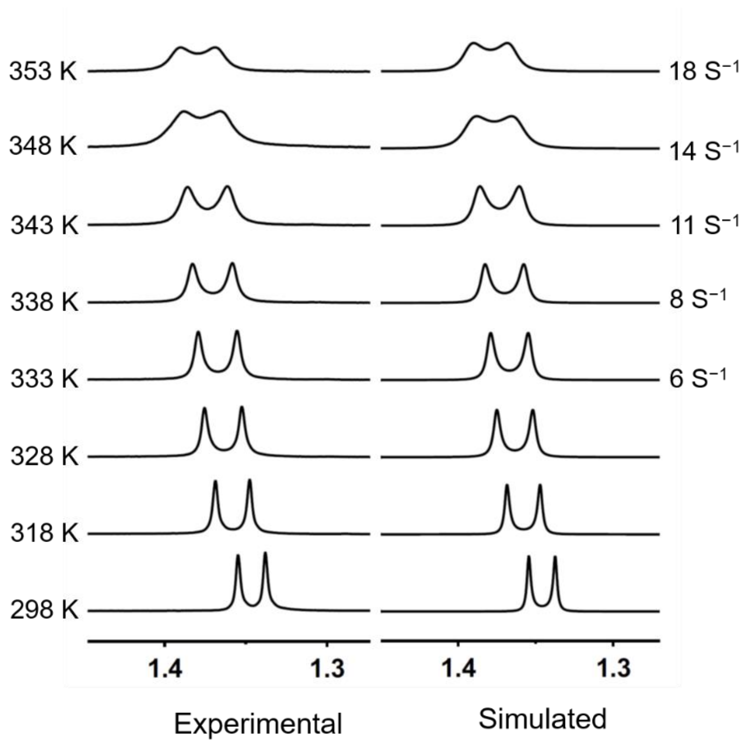

Supplementary Figure 81. Experimental and simulated VT ^{1}H NMR spectra (500 MHz, CD_3CN) of methyl peaks of **3-DBU-0.2eq LiClO$_4$**; [**3**] = 4 mM, [**LiClO$_4$**] = 0.8 mM (**0.2 equiv**). The temperature (K) and calculated interconversion rate constants (k_r, s^{-1}) are given for each trace.

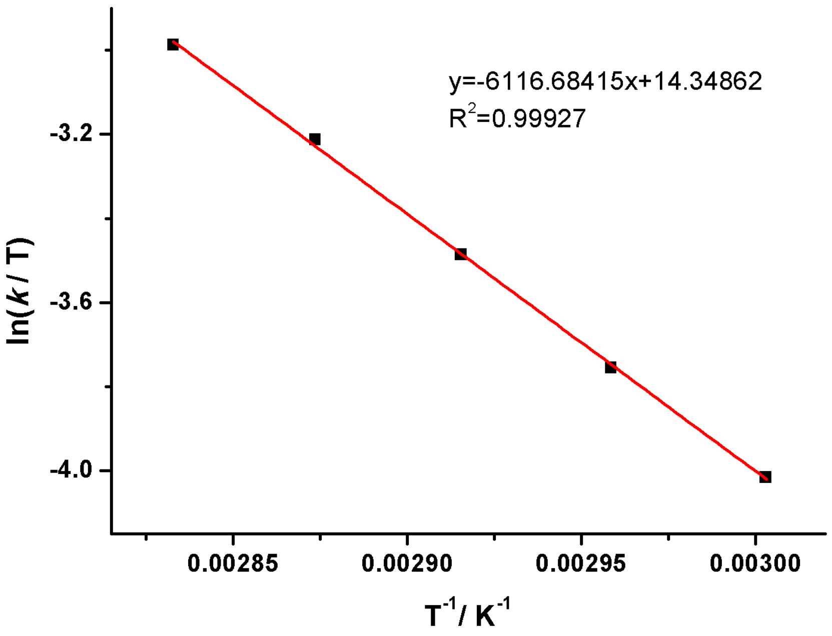

Supplementary Figure 82. Eyring plot of the rates of exchange obtained from line width analysis of methyl signals on VT ^{1}H NMR spectra of **3-DBU-0.2eq LiClO$_4$** in acetonitrile-d_3.

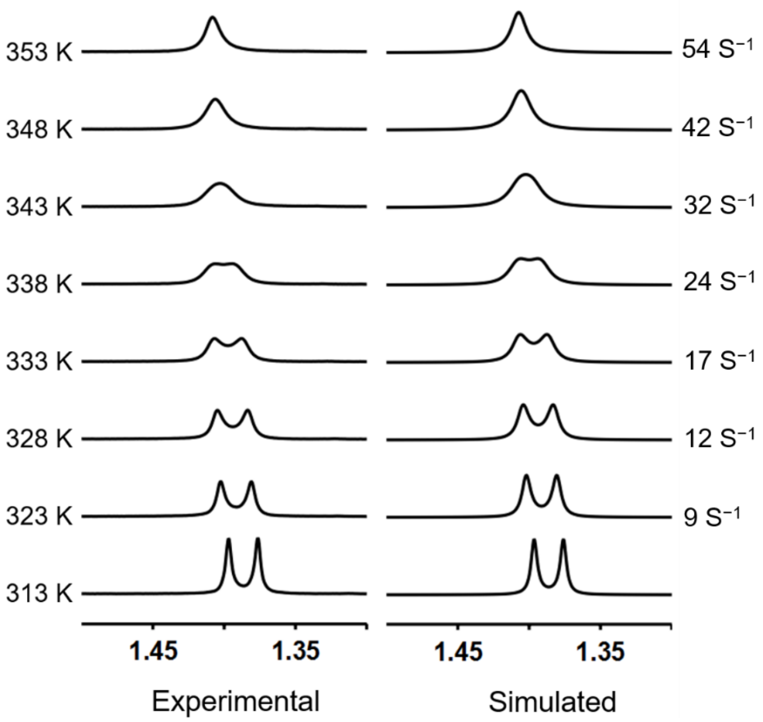

Supplementary Figure 83. Experimental and simulated VT ^{1}H NMR spectra (500 MHz, CD_3CN) of methyl peaks of **3-DBU-0.4eq LiClO$_4$**; [**3**] = 4 mM, [**LiClO$_4$**] = 1.6 mM (**0.4 equiv**). The temperature (K) and calculated interconversion rate constants (k_r, s^{-1}) are given for each trace.

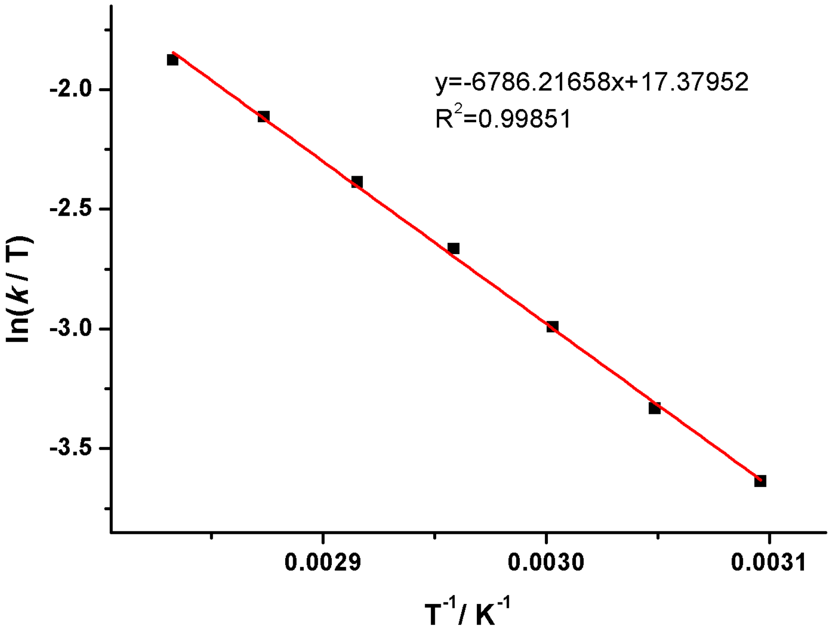

Supplementary Figure 84. Eyring plot of the rates of exchange obtained from line width analysis of methyl signals on VT ^{1}H NMR spectra of **3-DBU-0.4eq LiClO$_4$** in acetonitrile-d_3.

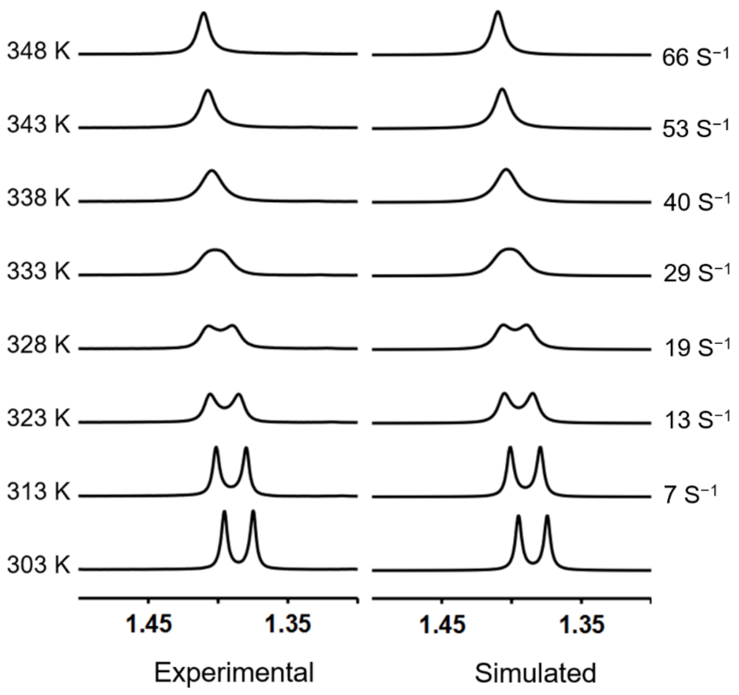

Supplementary Figure 85. Experimental and simulated VT ^{1}H NMR spectra (500 MHz, CD_3CN) of methyl peaks of **3-DBU-0.6eq $LiClO_4$**; [**3**] = 4 mM, [**$LiClO_4$**] = 2.4 mM (**0.6 equiv**). The temperature (K) and calculated interconversion rate constants (k_r, s^{-1}) are given for each trace.

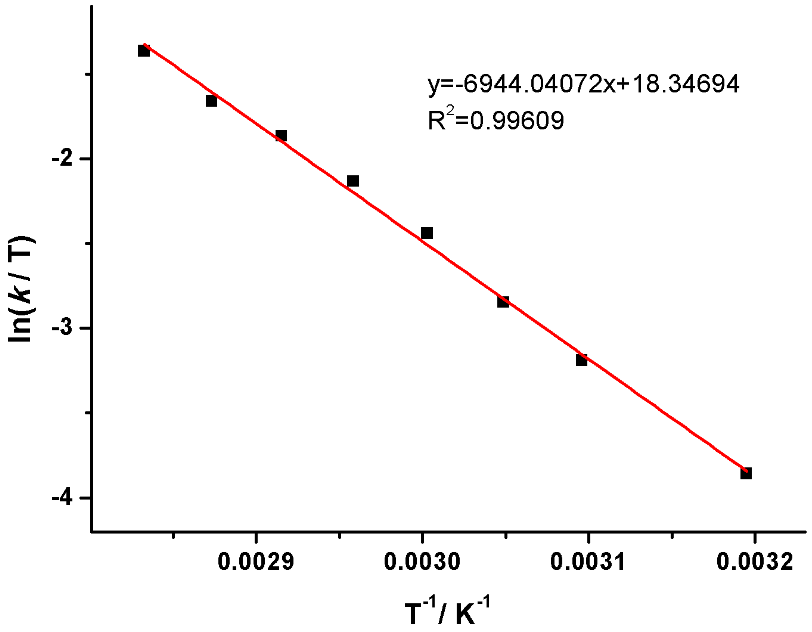

Supplementary Figure 86. Eyring plot of the rates of exchange obtained from line width analysis of methyl signals on VT ^{1}H NMR spectra of **3-DBU-0.6eq $LiClO_4$** in acetonitrile-d_3.

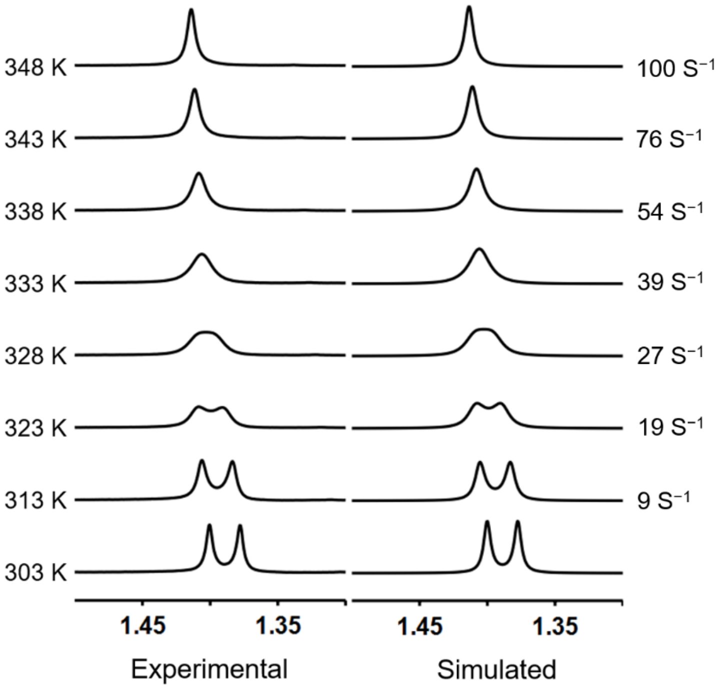

Supplementary Figure 87. Experimental and simulated VT ^{1}H NMR spectra (500 MHz, CD_3CN) of methyl peaks of **3-DBU-0.8eq** $\mathbf{LiClO_4}$; [**3**] = 4 mM, [$\mathbf{LiClO_4}$] = 3.2 mM (**0.8 equiv**). The temperature (K) and calculated interconversion rate constants (k_r, s^{-1}) are given for each trace.

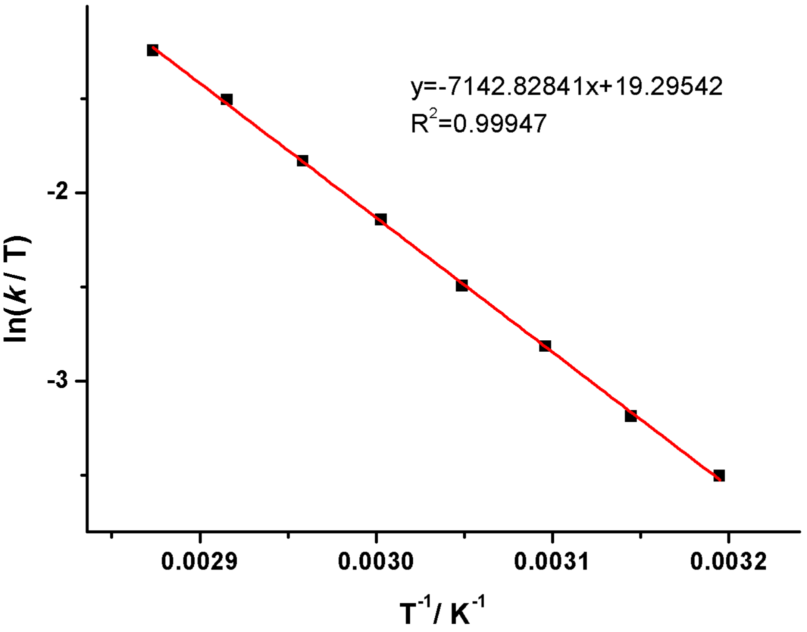

Supplementary Figure 88. Eyring plot of the rates of exchange obtained from line width analysis of methyl signals on VT ^{1}H NMR spectra of **3-DBU-0.8eq** $\mathbf{LiClO_4}$ in acetonitrile-d_3.

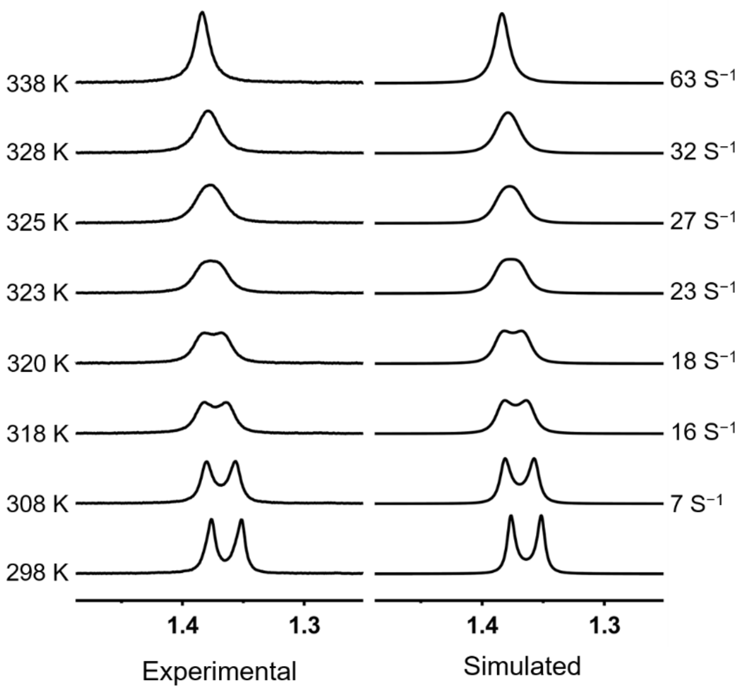

Supplementary Figure 89. Experimental and simulated VT ^{1}H NMR spectra (500 MHz, CD_3CN) of methyl peaks of **3-DBU-1.0eq LiClO$_4$**; [**3**] = 4 mM, [**LiClO$_4$**] = 4 mM (**1.0 equiv**). The temperature (K) and calculated interconversion rate constants (k_r, s^{-1}) are given for each trace.

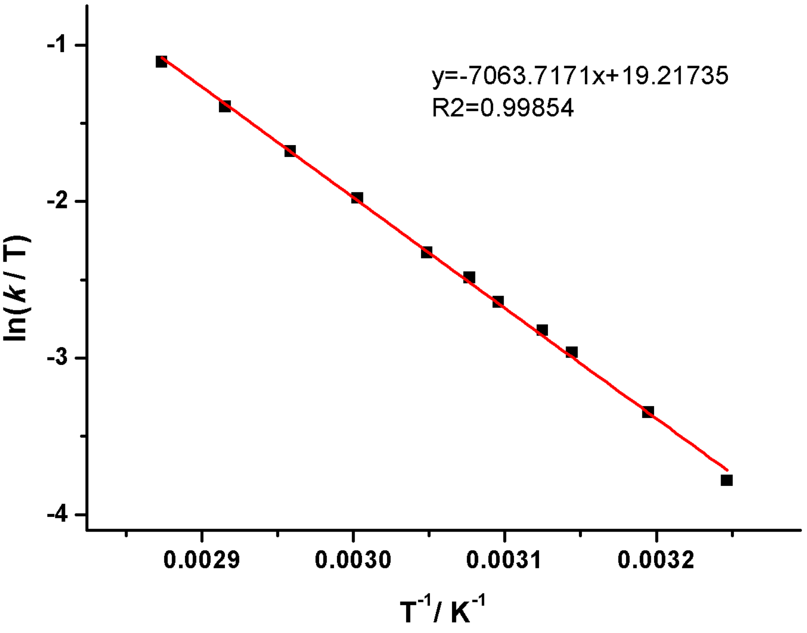

Supplementary Figure 90. Eyring plot of the rates of exchange obtained from line width analysis of methyl signals on VT ^{1}H NMR spectra of **3-DBU-1.0eq LiClO$_4$** in acetonitrile-d_3.

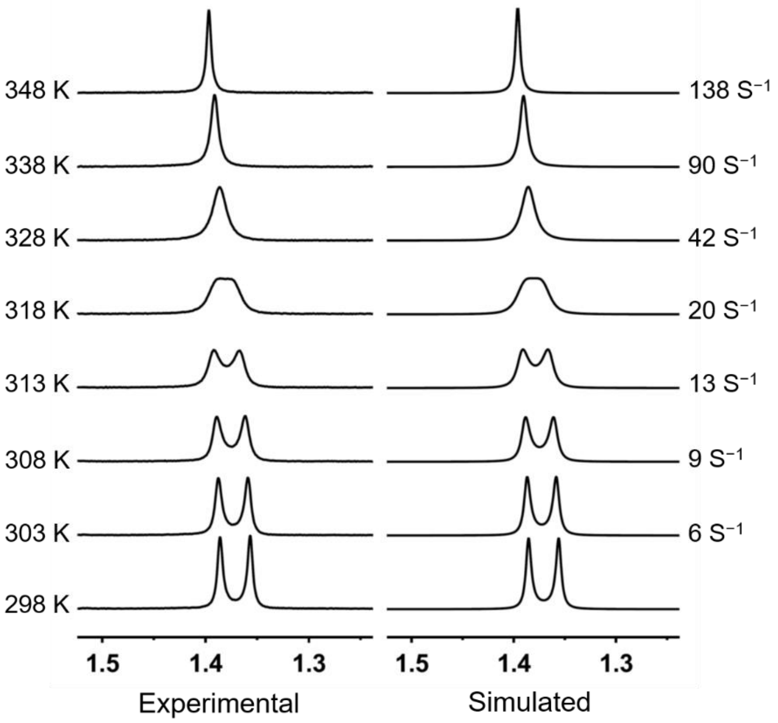

Supplementary Figure 91. Experimental and simulated VT ^{1}H NMR spectra (500 MHz, CD_3CN) of methyl peaks of **3-DBU-2.0eq $LiClO_4$**; [**3**] = 4 mM, [**$LiClO_4$**] = 8 mM (**2.0 equiv**). The temperature (K) and calculated interconversion rate constants (k_r, s^{-1}) are given for each trace.

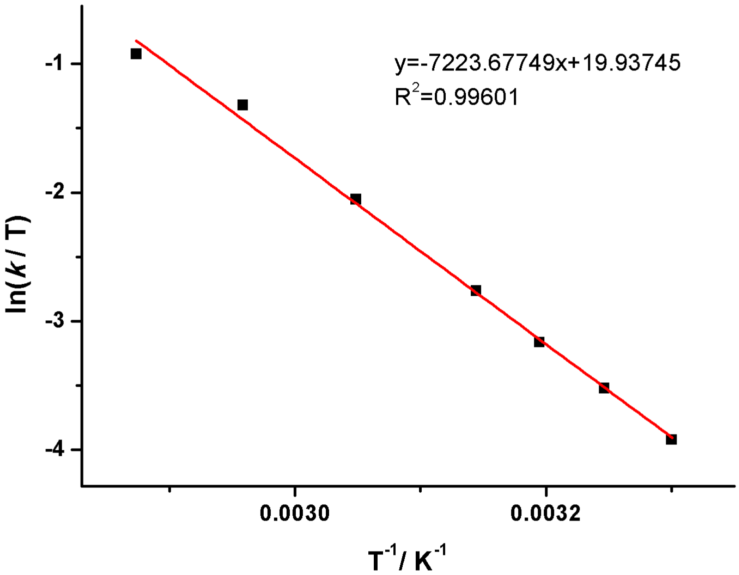

Supplementary Figure 92. Eyring plot of the rates of exchange obtained from line width analysis of methyl peaks signal in VT ^{1}H NMR spectra of **3-DBU-2.0eq $LiClO_4$** in acetonitrile-d_3.

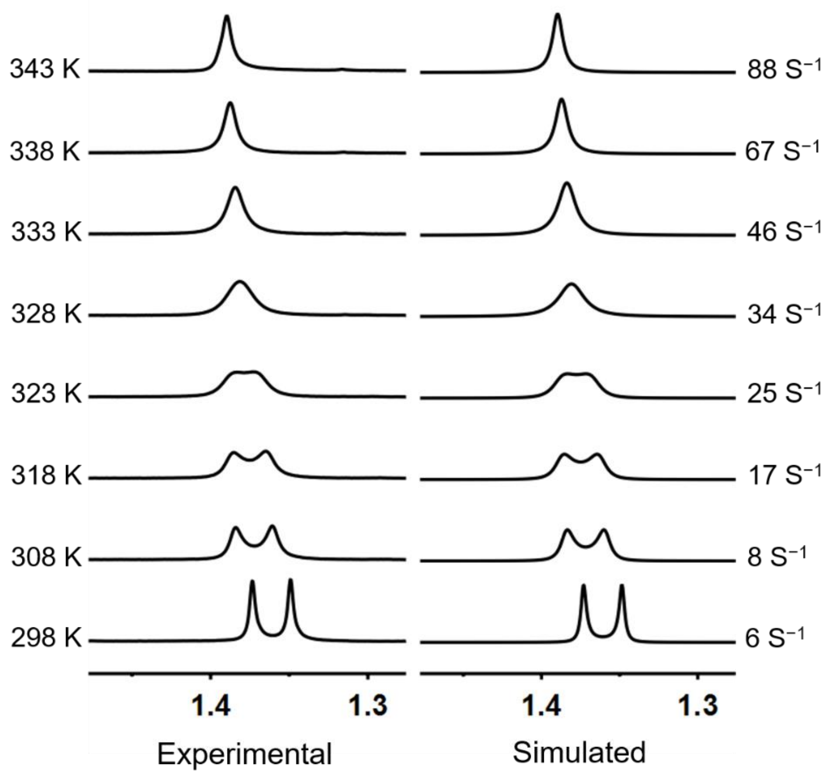

Supplementary Figure 93. Experimental and simulated VT ^{1}H NMR spectra (500 MHz, CD_3CN) of methyl peaks of **3-DBU-4.0eq LiClO$_4$**; [**3**] = 4 mM, [**LiClO$_4$**] = 16 mM (**4.0 equiv**). The temperature (K) and calculated interconversion rate constants (k_r, s^{-1}) are given for each trace.

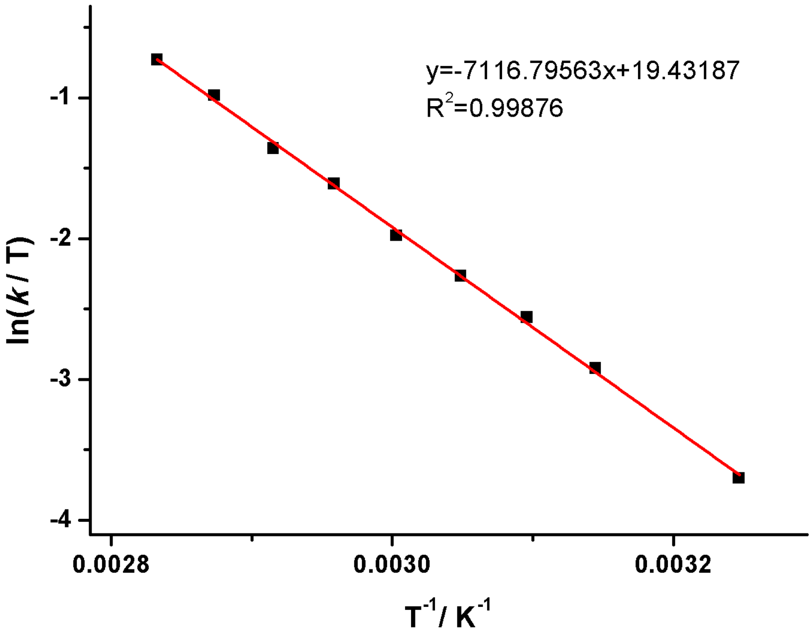

Supplementary Figure 94. Eyring plot of the rates of exchange obtained from line width analysis of methyl signals on VT ^{1}H NMR spectra of **3-DBU-4.0eq LiClO$_4$** in acetonitrile-d_3.

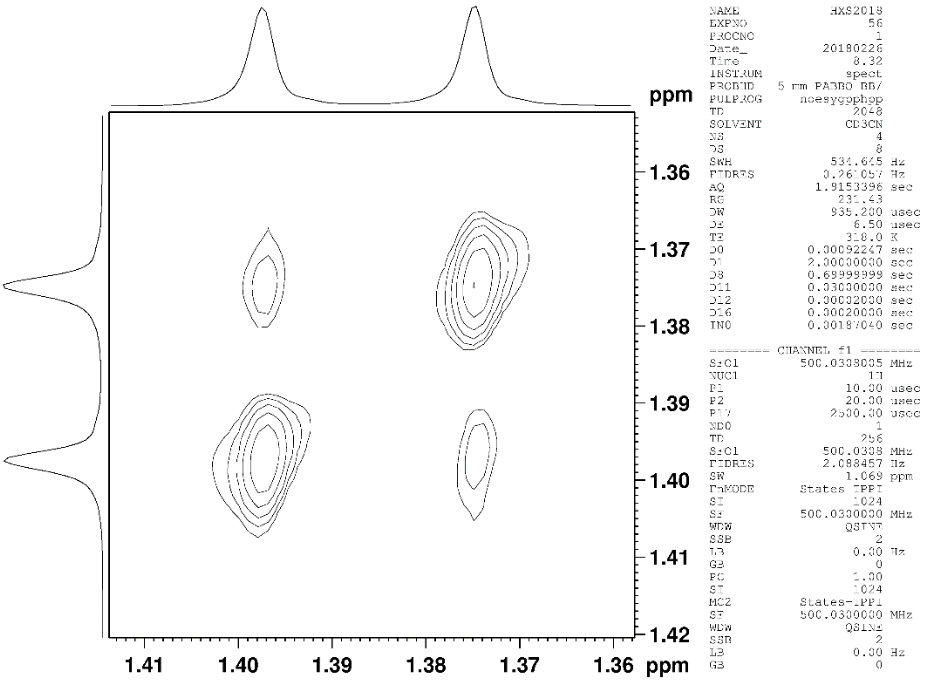

Supplementary Figure 95. 2D EXSY NMR (500 MHz, $t_m = 0.7$ s) spectrum of **3-DBU-NaClO$_4$** at 318 K in CD_3CN. [**3**] = 4 mM, [**DBU**] = 4.8 mM (1.2 equiv), [**NaClO$_4$**] = 1 mM (0.25 equiv).

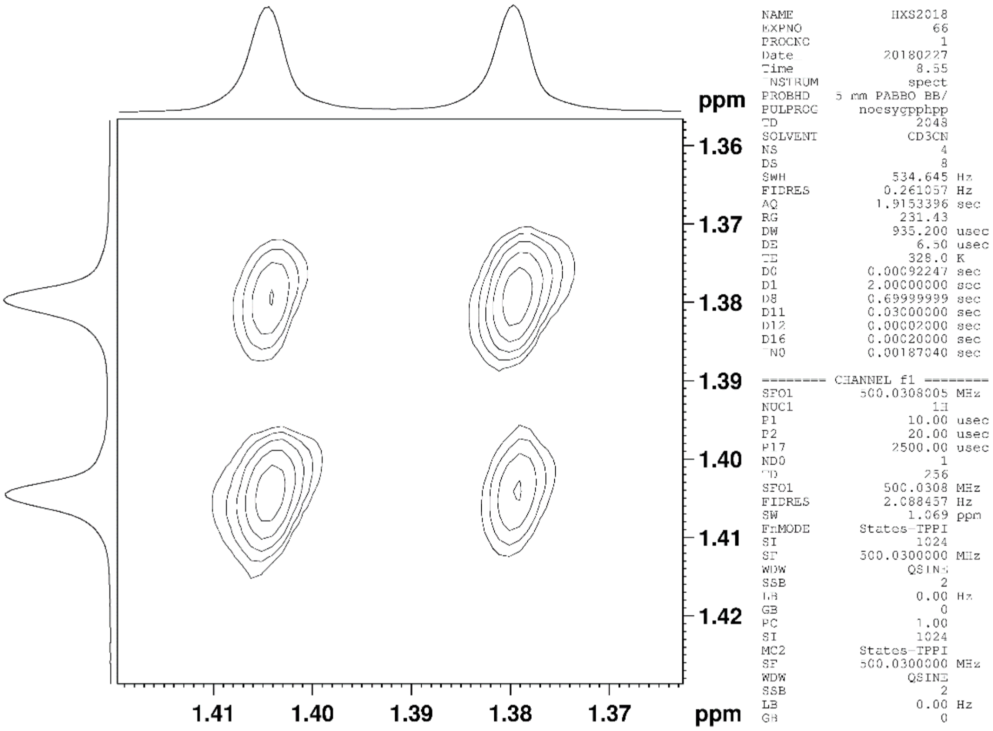

Supplementary Figure 96. 2D EXSY NMR (500 MHz, $t_m = 0.7$ s) spectrum of **3-DBU-NaClO$_4$** at 328 K in CD_3CN. [**3**] = 4 mM, [**DBU**] = 4.8 mM (1.2 equiv), [**NaClO$_4$**] = 1 mM (0.25 equiv).

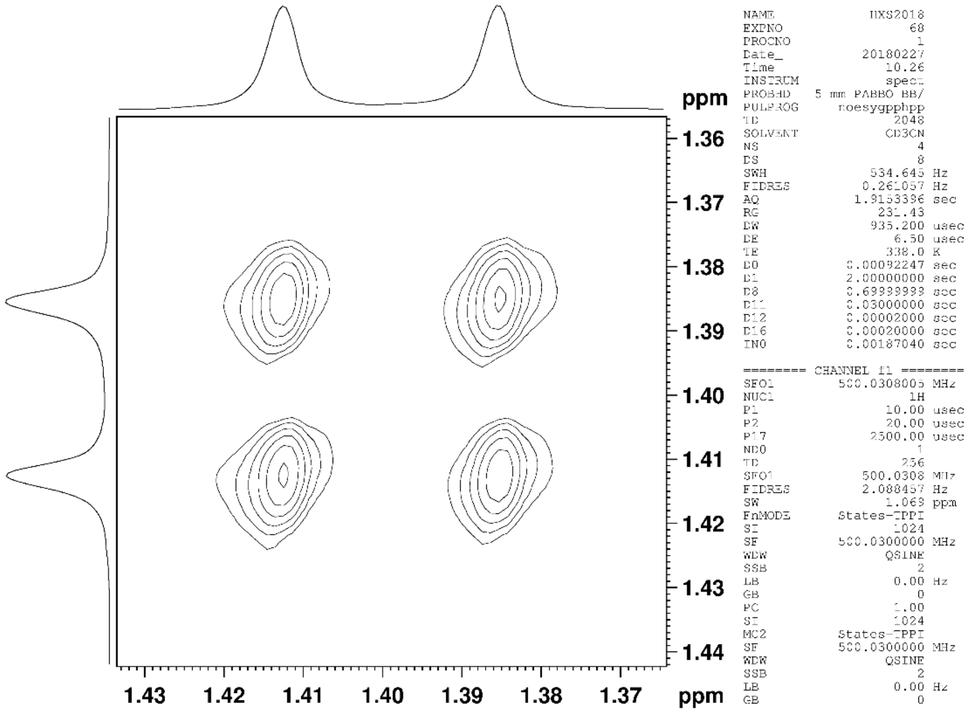

Supplementary Figure 97. 2D EXSY NMR (500 MHz, t_m = 0.7 s) spectrum of **3-DBU-NaClO$_4$** at 338 K in CD_3CN. [**3**] = 4 mM, [**DBU**] = 4.8 mM (1.2 equiv), [**NaClO$_4$**] = 1 mM (0.25 equiv).

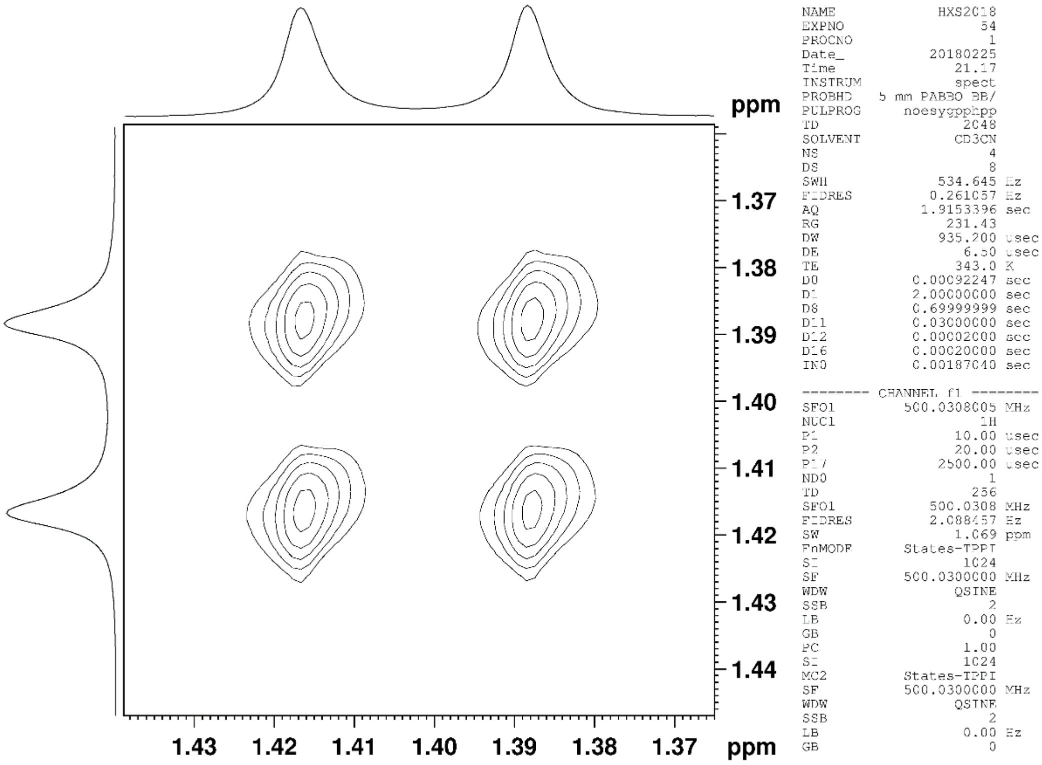

Supplementary Figure 98. 2D EXSY NMR (500 MHz, t_m = 0.7 s) spectrum of **3-DBU-NaClO$_4$** at 343 K in CD_3CN. [**3**] = 4 mM, [**DBU**] = 4.8 mM (1.2 equiv), [**NaClO$_4$**] = 1 mM (0.25 equiv).

Supplementary Table 14. The integral parameters from 2D EXSY NMR and calculated *k* values of **3-DBU-0.25eq** $NaClO_4$.

T(K)	I_A	I_B	I_{AB}	I_{BA}	k (s^{-1})
318	1.0000	0.9989	0.1948	0.2037	0.58
328	1.0000	1.0067	0.4299	0.4472	1.34
338	1.0000	1.0766	0.8402	0.8531	3.27
343	1.0000	1.0835	0.9449	0.9722	4.54

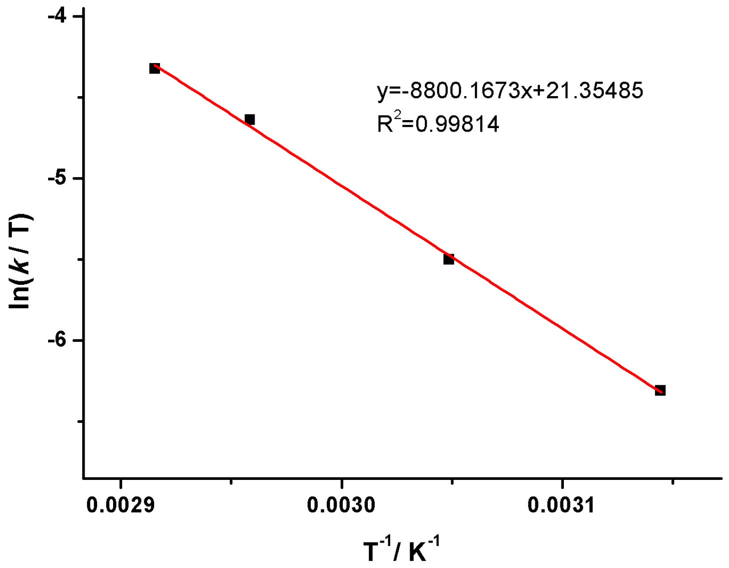

Supplementary Figure 99. Eyring plot of the rates of exchange between two diastereotopic methyl protons derived from VT 2D EXSY (from 318 K to 343 K) experiments on **3-DBU-0.25eq** $NaClO_4$ in acetonitrile-d_3.

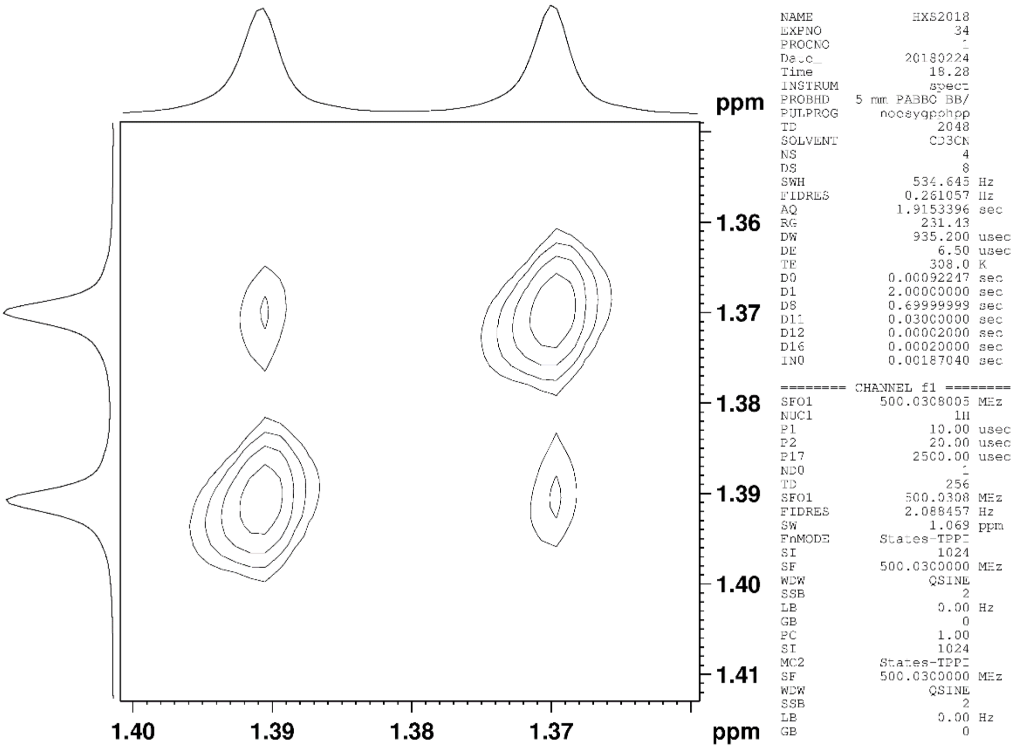

Supplementary Figure 100. 2D EXSY NMR (500 MHz, t_m = 0.7 s) spectrum of **3-DBU-NaClO$_4$** at 308 K in CD_3CN. [**3**] = 4 mM, [**DBU**] = 4.8 mM (1.2 equiv), [**NaClO$_4$**] = 2 mM (0.5 equiv).

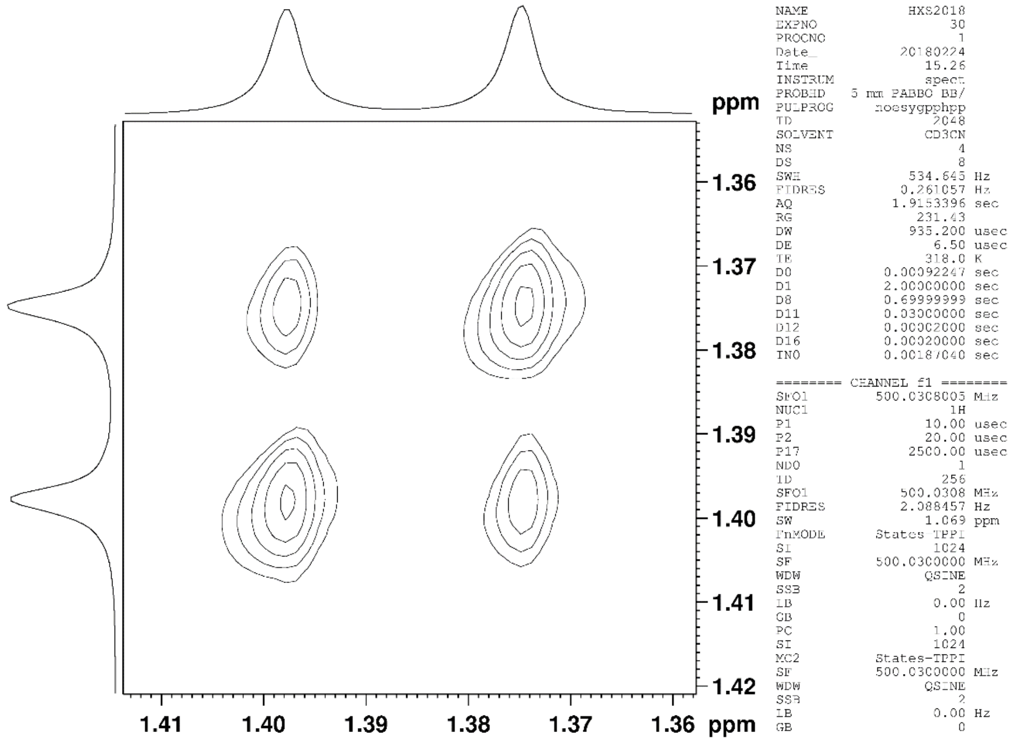

Supplementary Figure 101. 2D EXSY NMR (500 MHz, t_m = 0.7 s) spectrum of **3-DBU-NaClO$_4$** at 318 K in CD_3CN. [**3**] = 4 mM, [**DBU**] = 4.8 mM (1.2 equiv), [**NaClO$_4$**] = 2 mM (0.5 equiv).

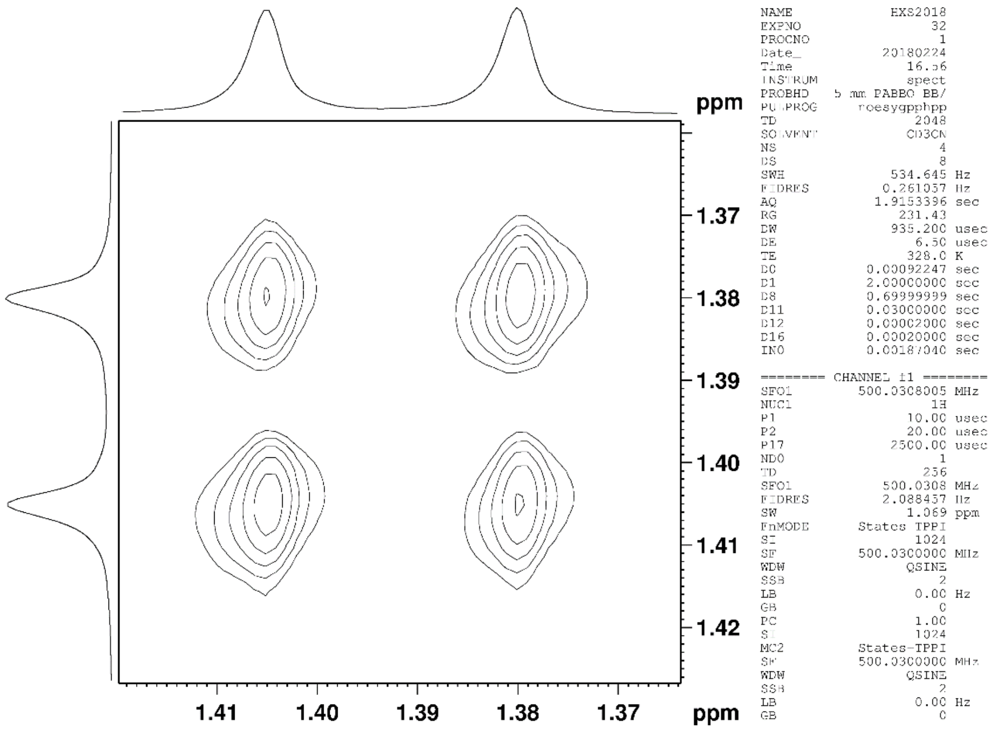

Supplementary Figure 102. 2D EXSY NMR (500 MHz, $t_m = 0.7$ s) spectrum of **3-DBU-NaClO$_4$** at 328 K in CD_3CN. [**3**] = 4 mM, [**DBU**] = 4.8 mM (1.2 equiv), [**NaClO$_4$**] = 2 mM (0.5 equiv).

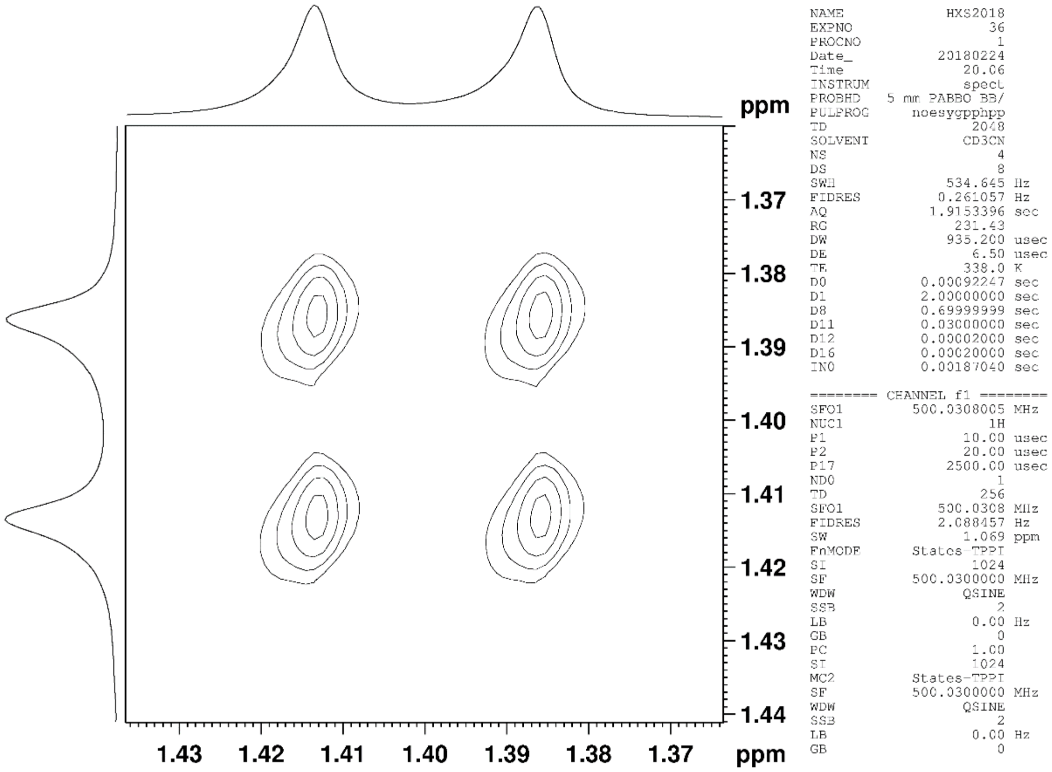

Supplementary Figure 103. 2D EXSY NMR (500 MHz, $t_m = 0.7$ s) spectrum of **3-DBU-NaClO$_4$** at 338 K in CD_3CN. [**3**] = 4 mM, [**DBU**] = 4.8 mM (1.2 equiv), [**NaClO$_4$**] = 2 mM (0.5 equiv).

Supplementary Table 15. The integral parameters from 2D EXSY NMR and calculated k values of **3-DBU-0.5eq $NaClO_4$**.

T(K)	I_A	I_B	I_{AB}	I_{BA}	k (s^{-1})
308	1.0000	0.9642	0.1836	0.2011	0.57
318	1.0000	0.9826	0.3776	0.4261	1.23
328	1.0000	0.9817	0.6834	0.6896	2.44
338	1.0000	0.9764	0.9598	0.9408	5.62

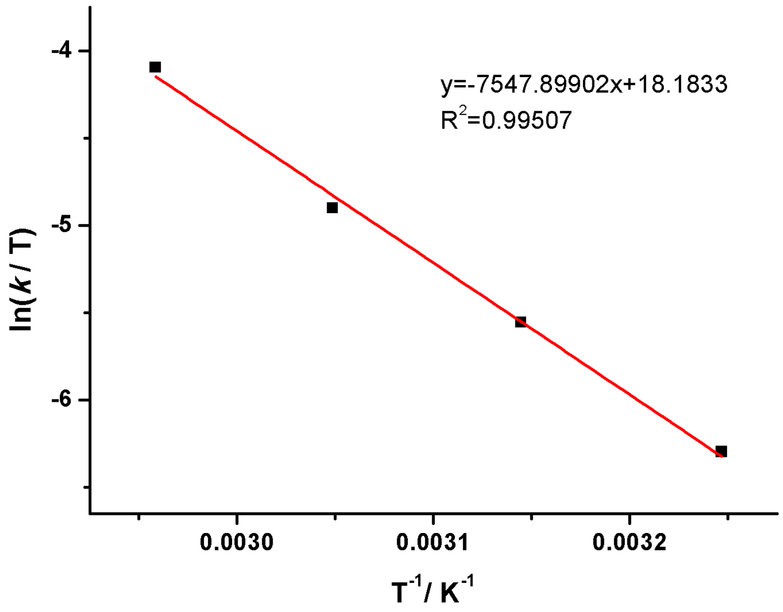

Supplementary Figure 104. Eyring plot of the rates of exchange between two diastereotopic methyl protons derived from VT 2D EXSY (from 308 K to 338 K) experiments on **3-DBU-0.5eq $NaClO_4$** in acetonitrile-d_3.

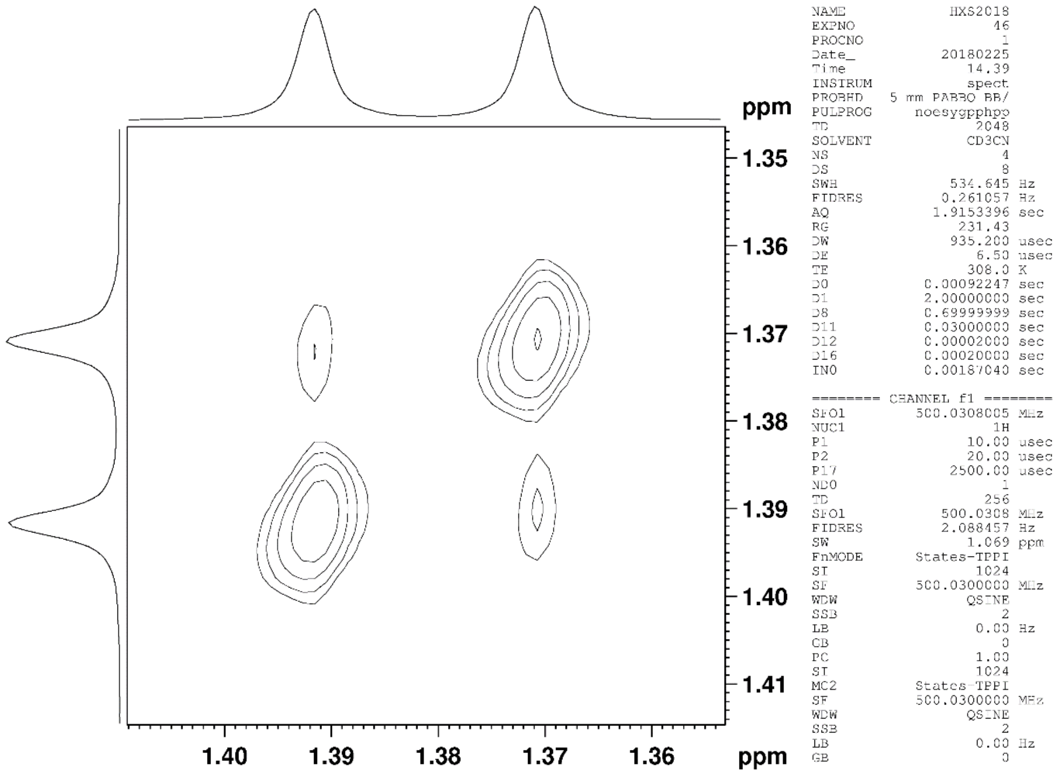

Supplementary Figure 105. 2D EXSY NMR (500 MHz, t_m = 0.7 s) spectrum of **3-DBU-NaClO$_4$** at 308 K in CD_3CN. [**3**] = 4 mM, [**DBU**] = 4.8 mM (1.2 equiv), [**NaClO$_4$**] = 3 mM (0.75 equiv).

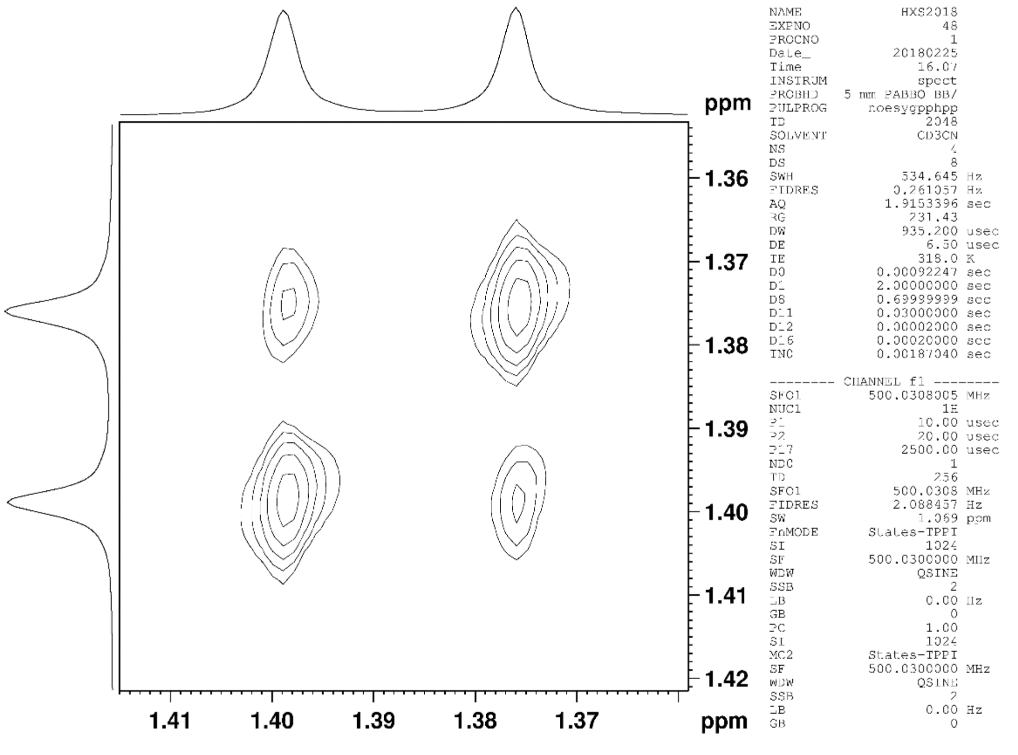

Supplementary Figure 106. 2D EXSY NMR (500 MHz, t_m = 0.7 s) spectrum of **3-DBU-NaClO$_4$** at 318 K in CD_3CN. [**3**] = 4 mM, [**DBU**] = 4.8 mM (1.2 equiv), [**NaClO$_4$**] = 3 mM (0.75 equiv).

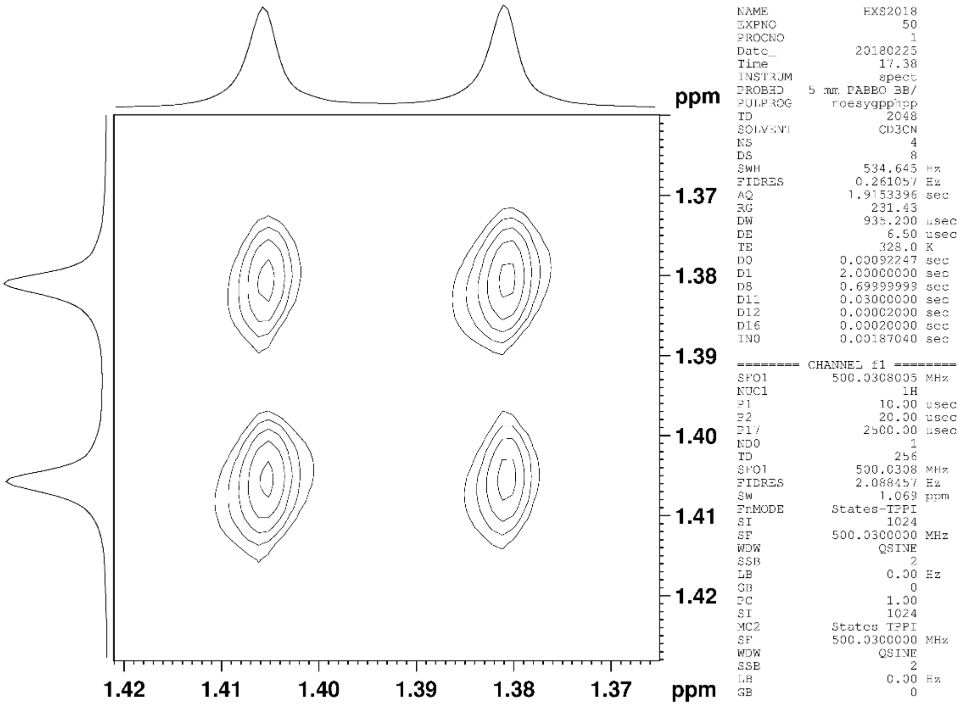

Supplementary Figure 107. 2D EXSY NMR (500 MHz, t_m = 0.7 s) spectrum of **3-DBU-NaClO$_4$** at 328 K in CD_3CN. [**3**] = 4 mM, [**DBU**] = 4.8 mM (1.2 equiv), [**NaClO$_4$**] = 3 mM (0.75 equiv).

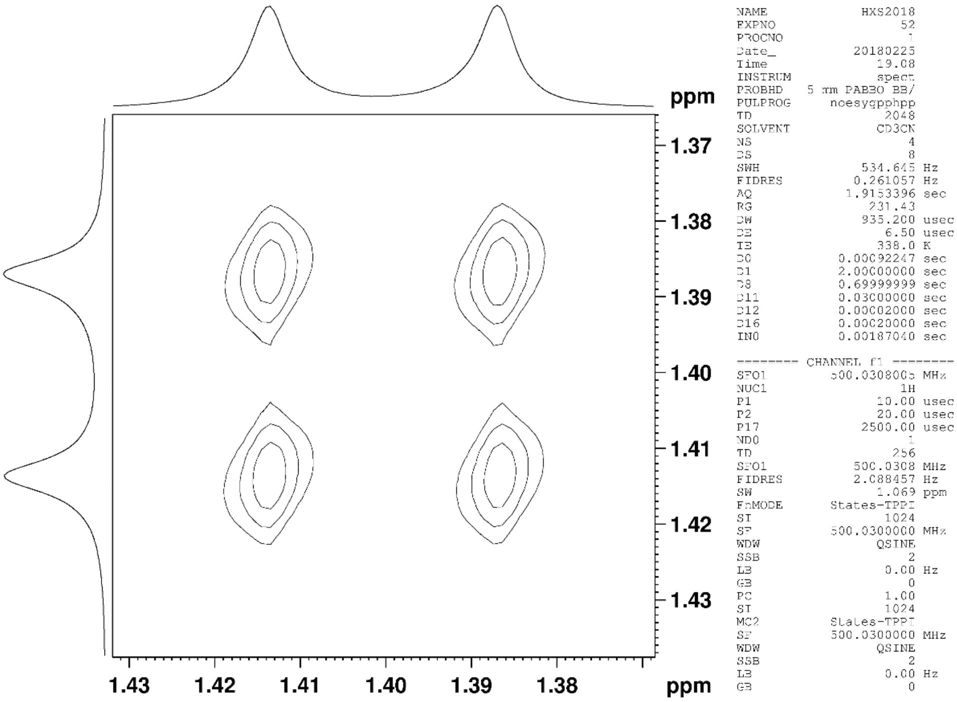

Supplementary Figure 108. 2D EXSY NMR (500 MHz, t_m = 0.7 s) spectrum of **3-DBU-NaClO$_4$** at 338 K in CD_3CN. [**3**] = 4 mM, [**DBU**] = 4.8 mM (1.2 equiv), [**NaClO$_4$**] = 3 mM (0.75 equiv).

Supplementary Table 16. The integral parameters from 2D EXSY NMR and calculated *k* values of **3-DBU-0.75eq $NaClO_4$**.

T(K)	I_A	I_B	I_{AB}	I_{BA}	k (s^{-1})
308	1.0000	0.9835	0.2365	0.2313	0.69
318	1.0000	0.9715	0.4595	0.4626	1.45
328	1.0000	0.9650	0.7681	0.7741	3.02
338	1.0000	0.9760	0.9697	0.9643	6.48

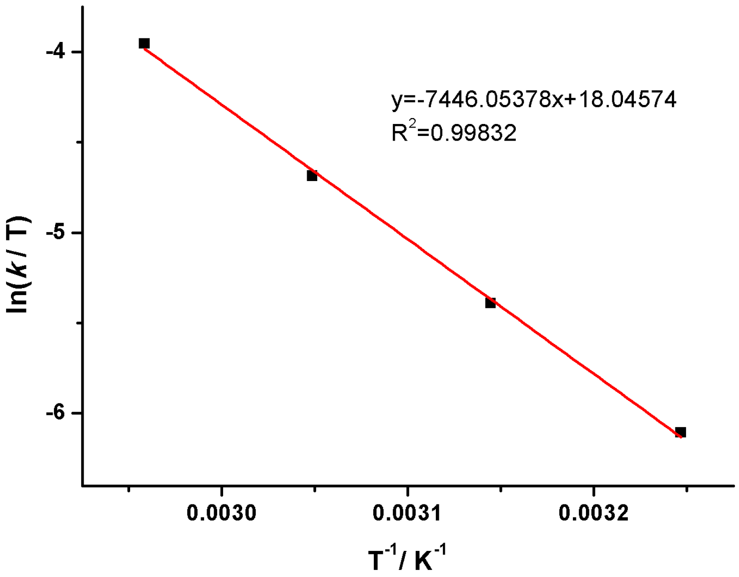

Supplementary Figure 109. Eyring plot of the rates of exchange between two diastereotopic methyl protons derived from VT 2D EXSY (from 308 K to 338 K) experiments on **3-DBU-0.75eq $NaClO_4$** in acetonitrile-*d*3.

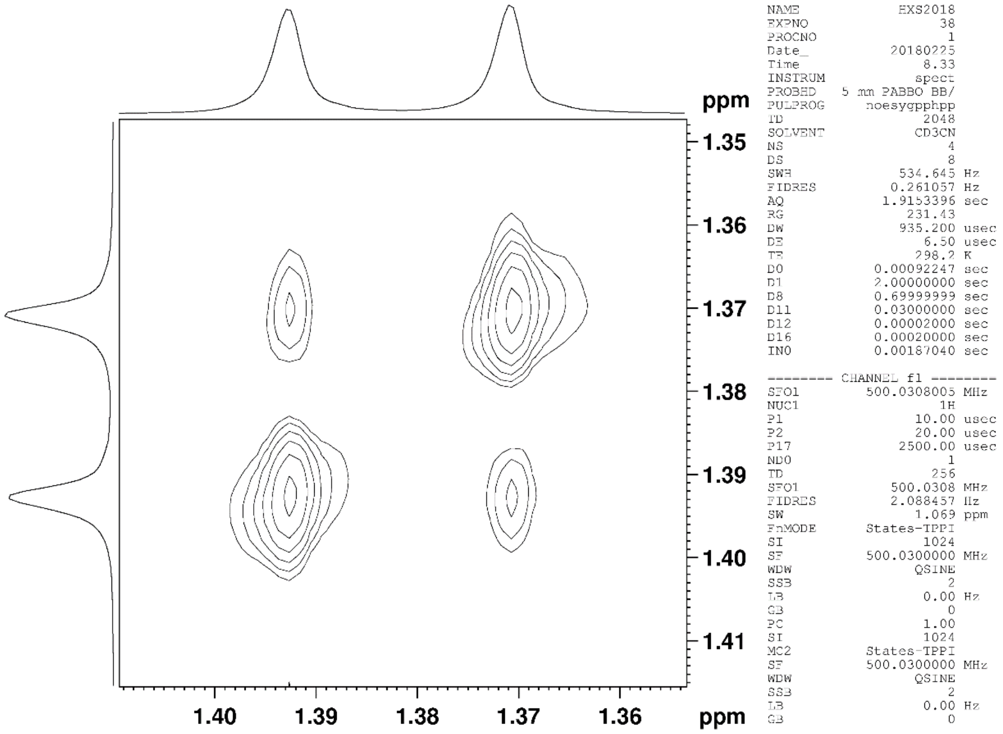

Supplementary Figure 110. 2D EXSY NMR (500 MHz, $t_m = 0.7$ s) spectrum of **3-DBU-NaClO$_4$** at 298 K in CD_3CN. [**3**] = 4 mM, [**DBU**] = 4.8 mM (1.2 equiv), [**NaClO$_4$**] = 16 mM (4.0 equiv).

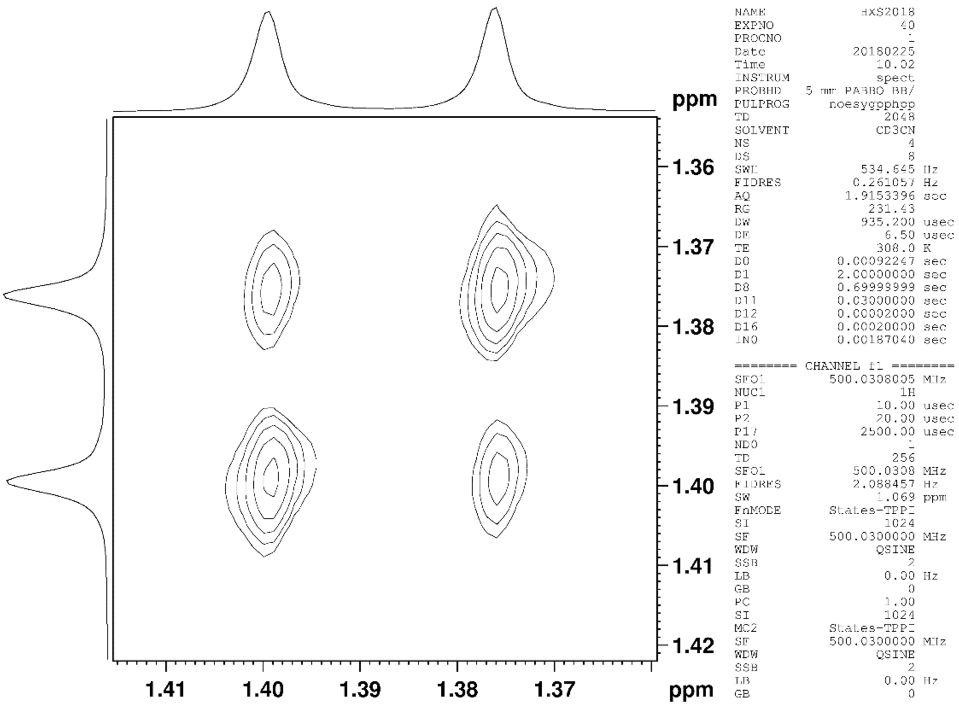

Supplementary Figure 111. 2D EXSY NMR (500 MHz, $t_m = 0.7$ s) spectrum of **3-DBU-NaClO$_4$** at 308 K in CD_3CN. [**3**] = 4 mM, [**DBU**] = 4.8 mM (1.2 equiv), [**NaClO$_4$**] = 16 mM (4.0 equiv).

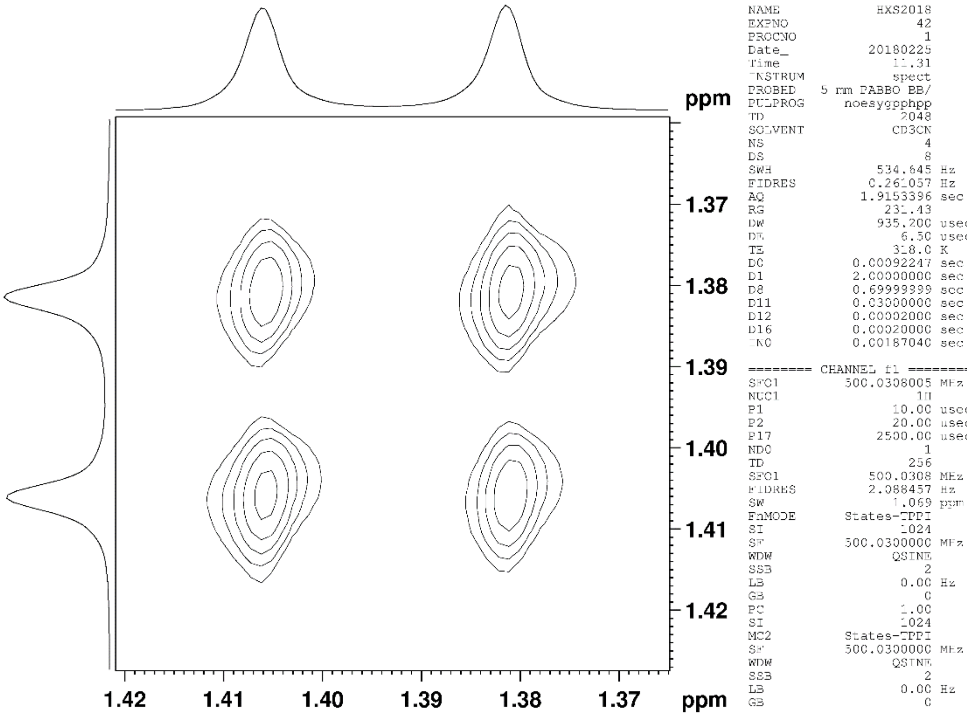

Supplementary Figure 112. 2D EXSY NMR (500 MHz, t_m = 0.7 s) spectrum of **3-DBU-NaClO$_4$** at 318 K in CD_3CN. [**3**] = 4 mM, [**DBU**] = 4.8 mM (1.2 equiv), [**NaClO$_4$**] = 16 mM (4.0 equiv).

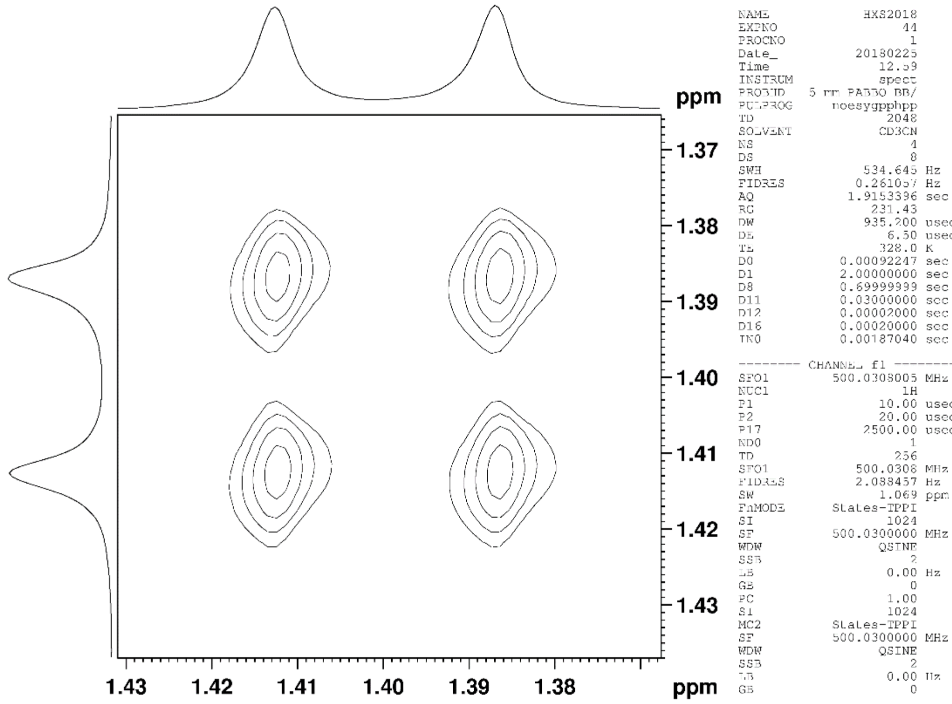

Supplementary Figure 113. 2D EXSY NMR (500 MHz, t_m = 0.7 s) spectrum of **3-DBU-NaClO$_4$** at 328 K in CD_3CN. [**3**] = 4 mM, [**DBU**] = 4.8 mM (1.2 equiv), [**NaClO$_4$**] = 16 mM (4.0 equiv).

Supplementary Table 17. The integral parameters from 2D EXSY NMR and calculated *k* values of **3-DBU-4.0eq $NaClO_4$**.

T(K)	I_A	I_B	I_{AB}	I_{BA}	k (s^{-1})
298	1.0000	0.9490	0.1688	0.1658	0.50
308	1.0000	0.9444	0.3435	0.3535	1.07
318	1.0000	0.9636	0.6949	0.7007	2.54
328	1.0000	0.9916	0.9347	1.0033	6.14

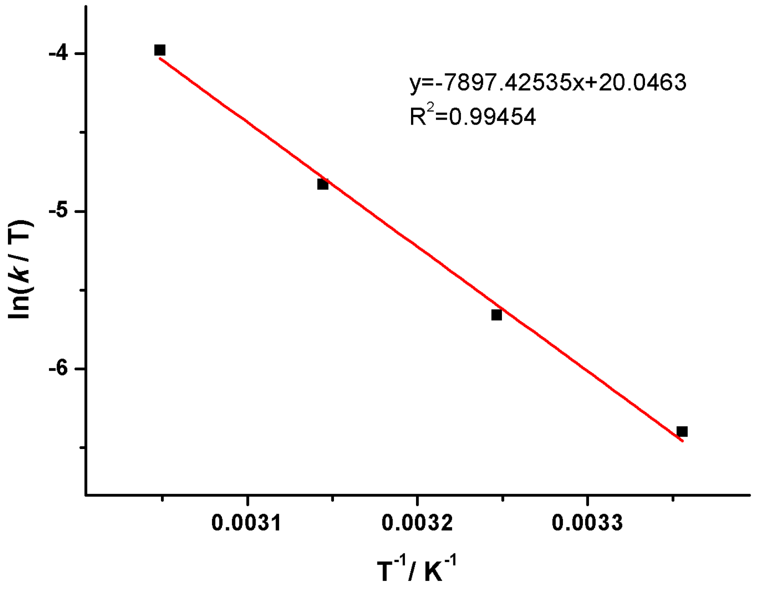

Supplementary Figure 114. Eyring plot of the rates of exchange between two diastereotopic methyl protons derived from VT 2D EXSY (from 298 K to 328 K) experiments on **3-DBU-4.0eq $NaClO_4$** in acetonitrile-d_3.

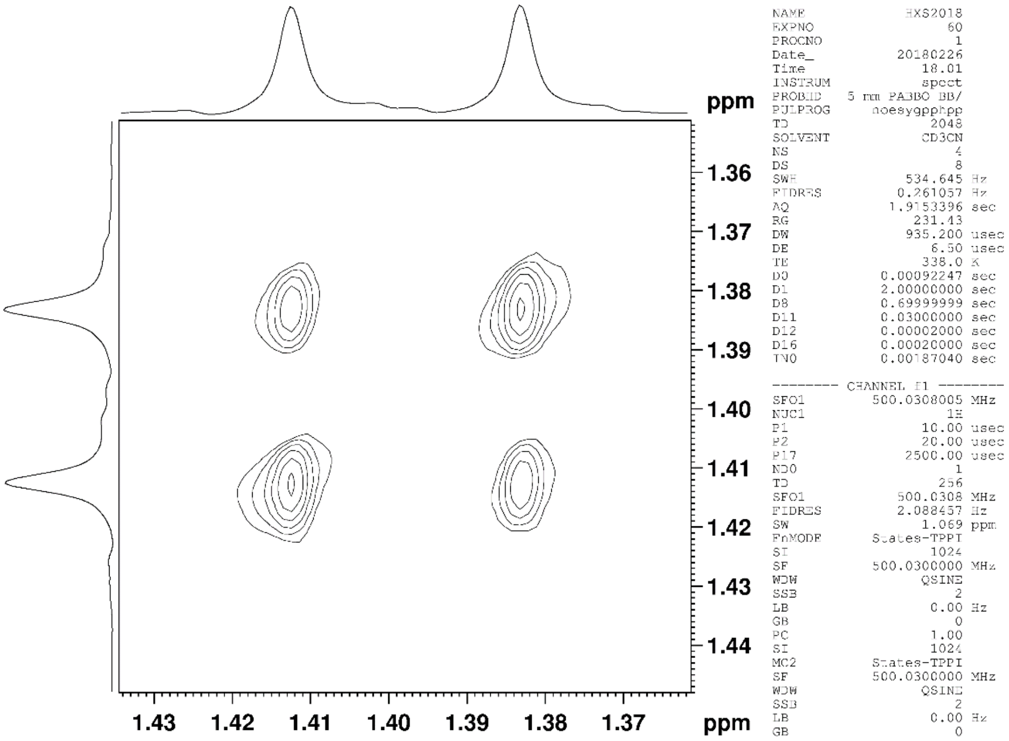

Supplementary Figure 115. 2D EXSY NMR (500 MHz, t_m = 0.7 s) spectrum of **3-DBU-KClO$_4$** at 338 K in CD_3CN. [**3**] = 4 mM, [**DBU**] = 4.8 mM (1.2 equiv), [**KClO$_4$**] = 2 mM (0.5 equiv).

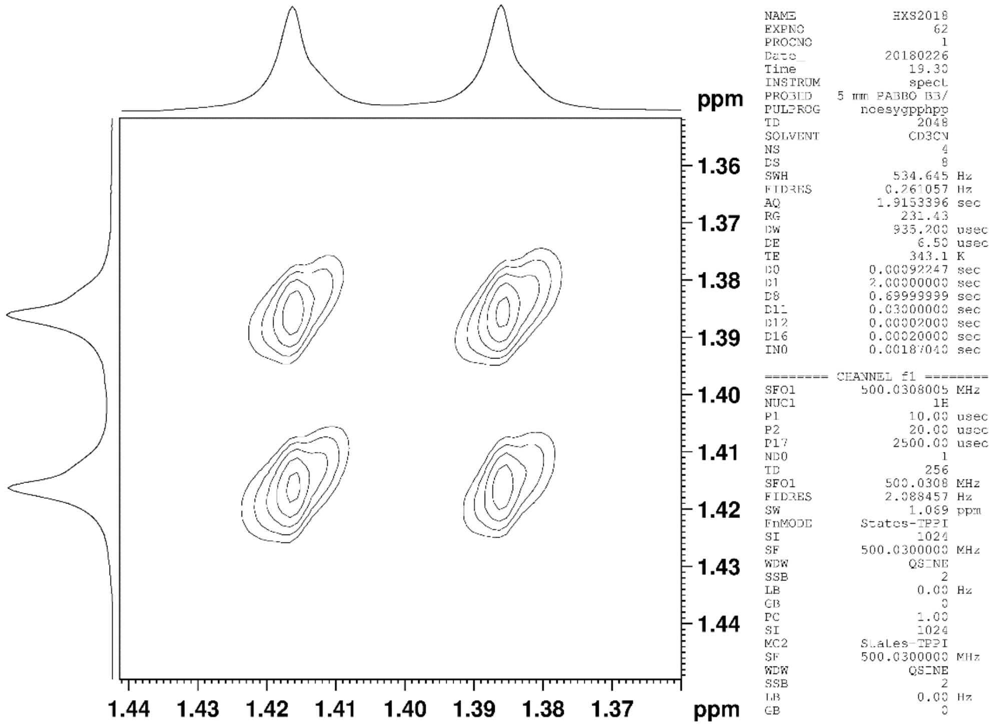

Supplementary Figure 116. 2D EXSY NMR (500 MHz, t_m = 0.7 s) spectrum of **3-DBU-KClO$_4$** at 343 K in CD_3CN. [**3**] = 4 mM, [**DBU**] = 4.8 mM (1.2 equiv), [**KClO$_4$**] = 2 mM (0.5 equiv).

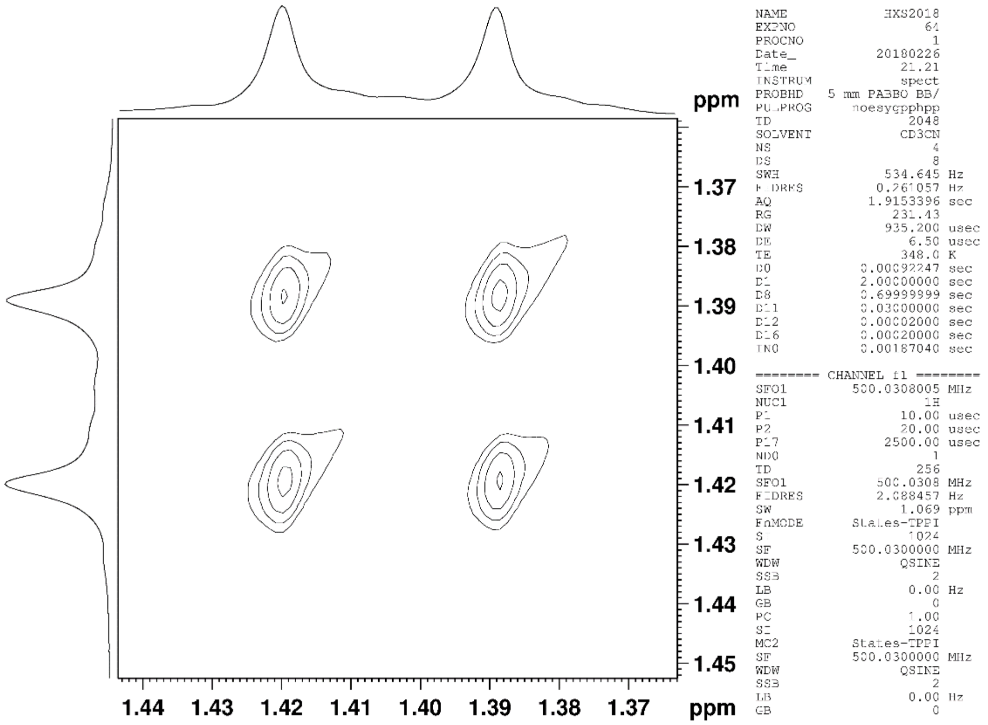

Supplementary Figure 117. 2D EXSY NMR (500 MHz, t_m = 0.7 s) spectrum of **3-DBU-KClO$_4$** at 348 K in CD_3CN. [**3**] = 4 mM, [**DBU**] = 4.8 mM (1.2 equiv), [**KClO$_4$**] = 2 mM (0.5 equiv).

Supplementary Table 18. The integral parameters from 2D EXSY NMR and calculated *k* values of **3-DBU-0.5eq KClO$_4$**.

T(K)	I_A	I_B	I_{AB}	I_{BA}	*k* (s^{-1})
338	1.0000	1.0061	0.5579	0.5621	1.80
343	1.0000	0.9727	0.7561	0.7626	2.91
348	1.0000	0.9817	0.9034	0.9196	4.54

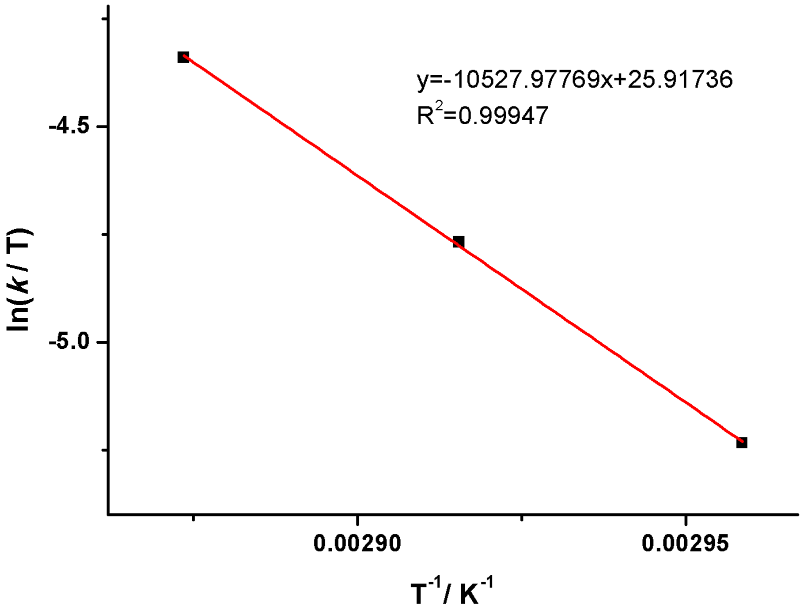

Supplementary Figure 118. Eyring plot of the rates of exchange between two diastereotopic methyl protons derived from VT 2D EXSY (from 338 K to 348 K) experiments on **3-DBU-0.5eq $KClO_4$** in acetonitrile-d_3.

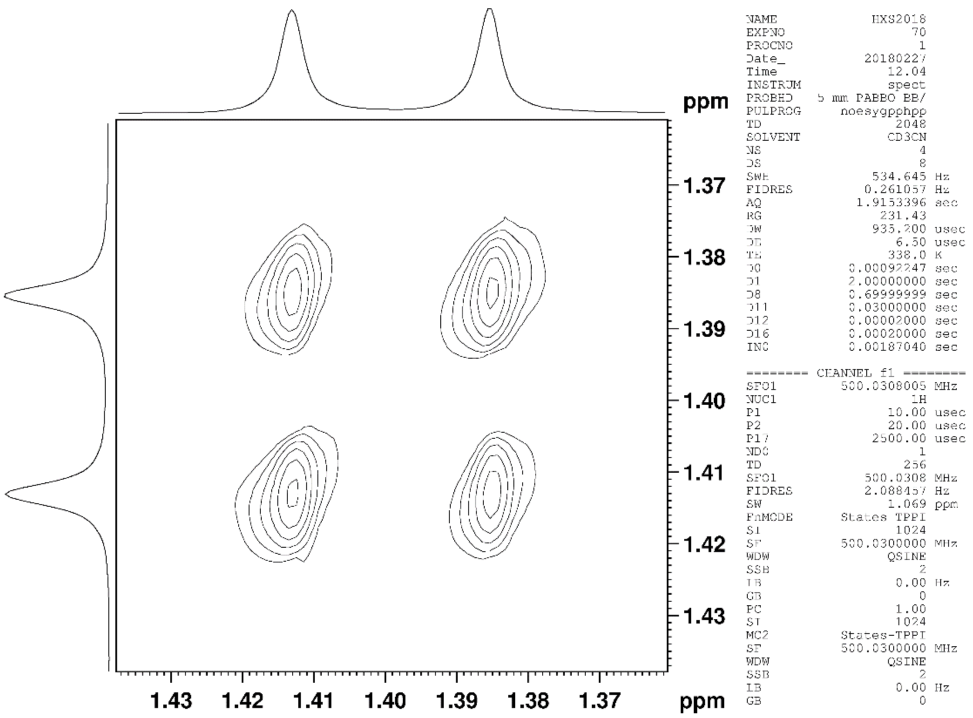

Supplementary Figure 119. 2D of EXSY NMR (500 MHz, t_m = 0.7 s) spectrum of **3-DBU-$KClO_4$** at 338 K in CD_3CN. [**3**] = 4 mM, [**DBU**] = 4.8 mM (1.2 equiv), [**$KClO_4$**] = 16 mM (4.0 equiv).

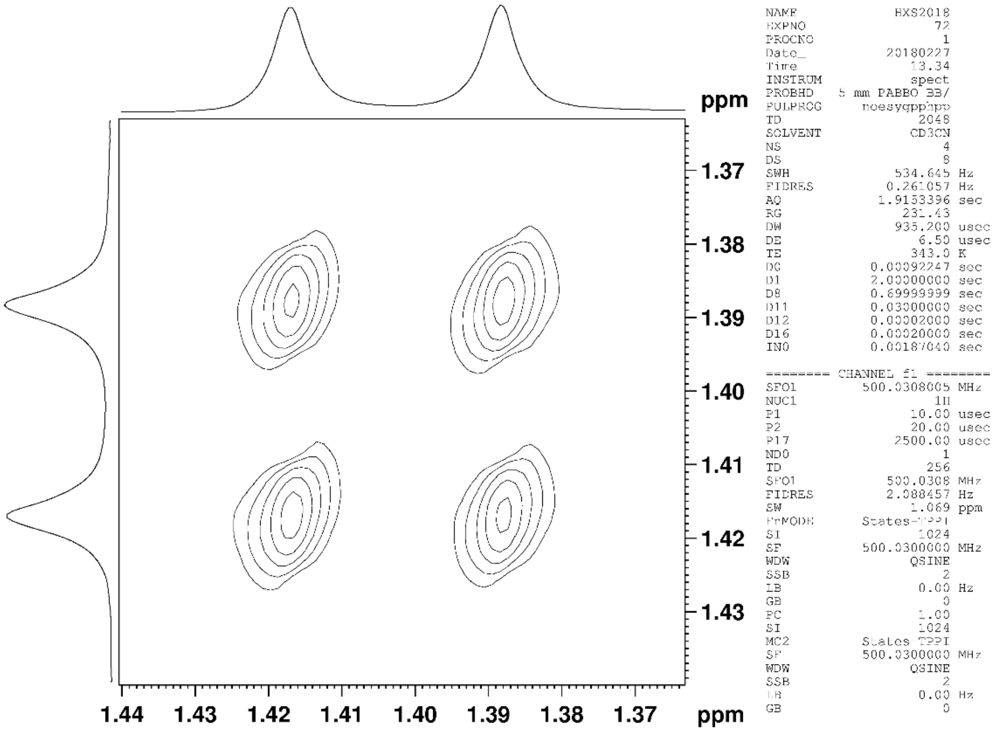

Supplementary Figure 120. 2D EXSY NMR (500 MHz, t_m = 0.7 s) spectrum of **3-DBU-KClO$_4$** at 343 K in CD_3CN. [**3**] = 4 mM, [**DBU**] = 4.8 mM (1.2 equiv), [**KClO$_4$**] = 16 mM (4.0 equiv).

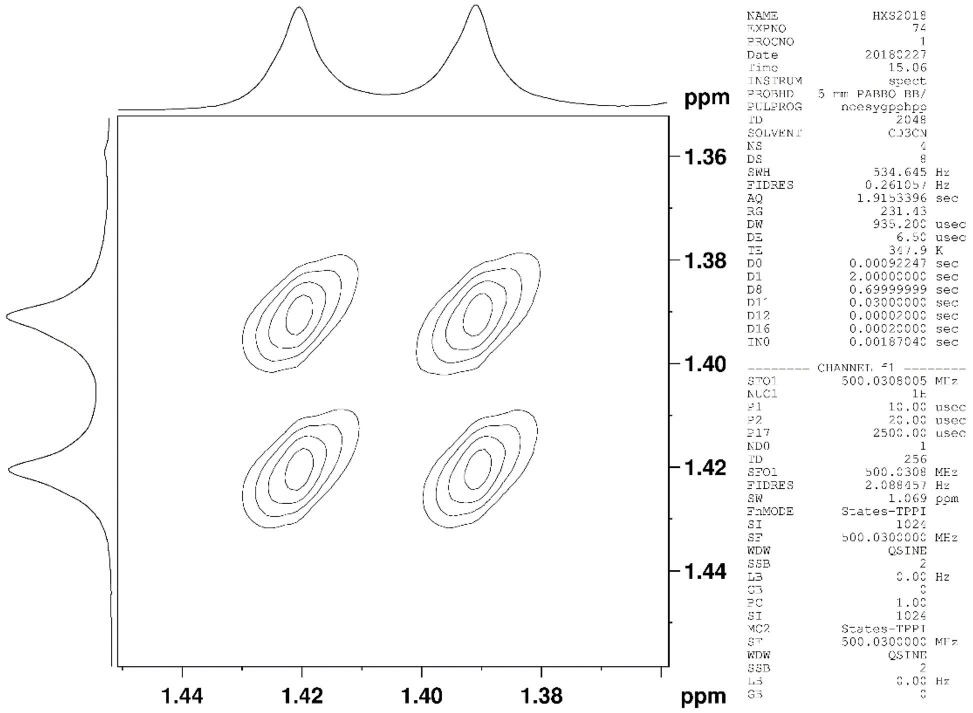

Supplementary Figure 121. 2D EXSY NMR (500 MHz, t_m = 0.7 s) spectrum of **3-DBU-KClO$_4$** at 348 K in CD_3CN. [**3**] = 4 mM, [**DBU**] = 4.8 mM (1.2 equiv), [**KClO$_4$**] = 16 mM (4.0 equiv).

Supplementary Table 19. The integral parameters from 2D EXSY NMR and calculated *k* values of **3-DBU-4.0eq $KClO_4$**.

T(K)	I_A	I_B	I_{AB}	I_{BA}	k (s^{-1})
338	1.0000	0.9938	0.6290	0.6340	2.13
343	1.0000	0.9898	0.8232	0.8319	3.41
348	1.0000	0.9457	0.9146	0.9350	5.25

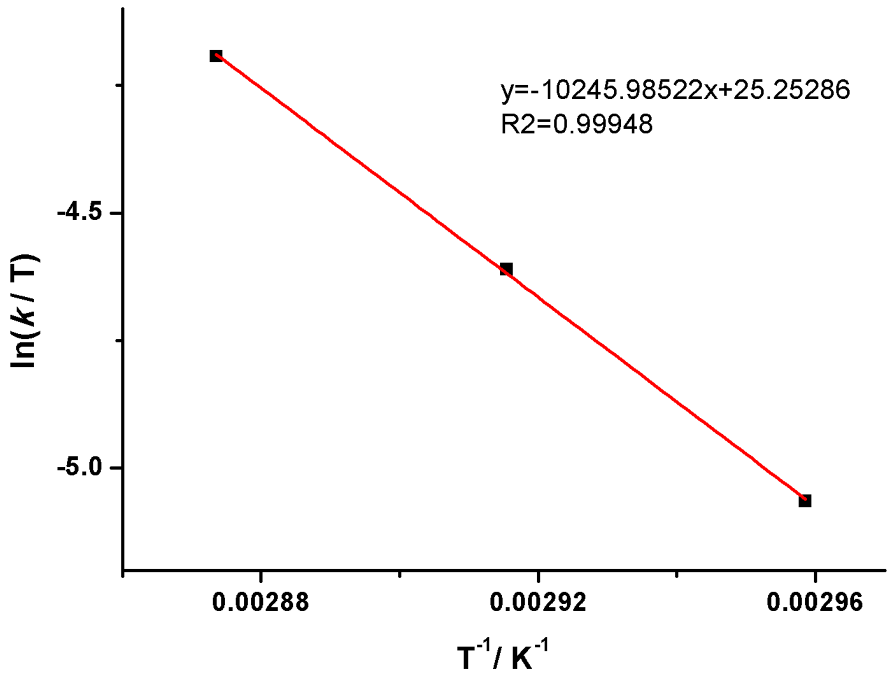

Supplementary Figure 122. Eyring plot of the rates of exchange between two diastereotopic methyl protons derived from VT 2D EXSY (from 338 K to 348 K) experiments on **3-DBU-4.0eq $KClO_4$** in acetonitrile-d_3.

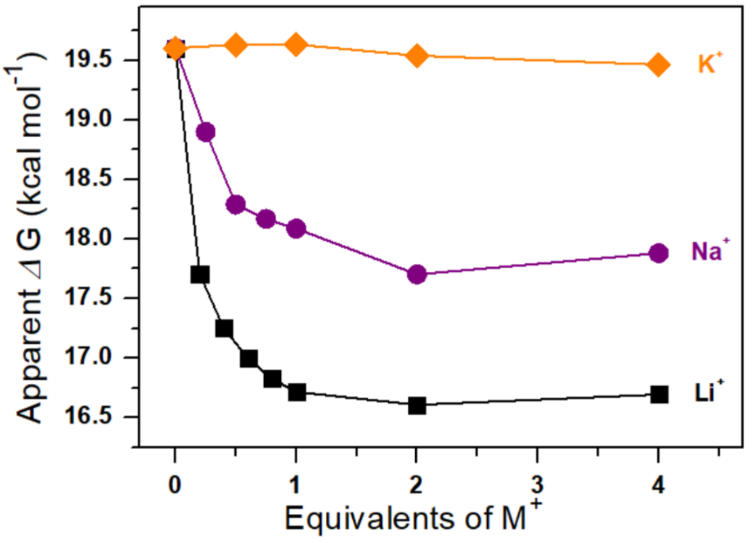

Supplementary Figure 123. Plots of the apparent free energy of activation ($\Delta G^{\ddagger}$) at 298 K for motion of (**3**–H)$^{-}$ versus the amount (equiv.) of Li^{+}, Na^{+} and K^{+} presented in the solution.

Supplementary Method 7. Transformations between High-speed Rotation and a Full Stop.

Inter-transformation between a high-speed and a highly-restricted rotation was verified on system of rotor **3**, by measuring the dynamics of **3** in response to base and acid alternating stimuli. The experiment was performed by a cyclic alternate addition of the concentrated solution of DBU (4 μL, 500 mM) and TFA (4 μL, 500 mM) in acetonitrile-d_3 to the solution of **3** (4 mM, 0.5 mL) in acetonitrile-d_3. In this process, a series of ^{1}H NMR spectra of the sample was recorded by a JEOL 600 MHz (^{1}H) spectrometer at 298 K. The obtained spectra are shown in Supplementary Fig. 124. Considering the highly reversible inter-transformation between a high-speed rotation and a highly-restricted rotation could be easily demonstrated by the reversible changes in the ^{1}H NMR spectrum pattern in the cyclic base-acid addition process, corresponding dynamic parameters relating to each trace are not calculated.

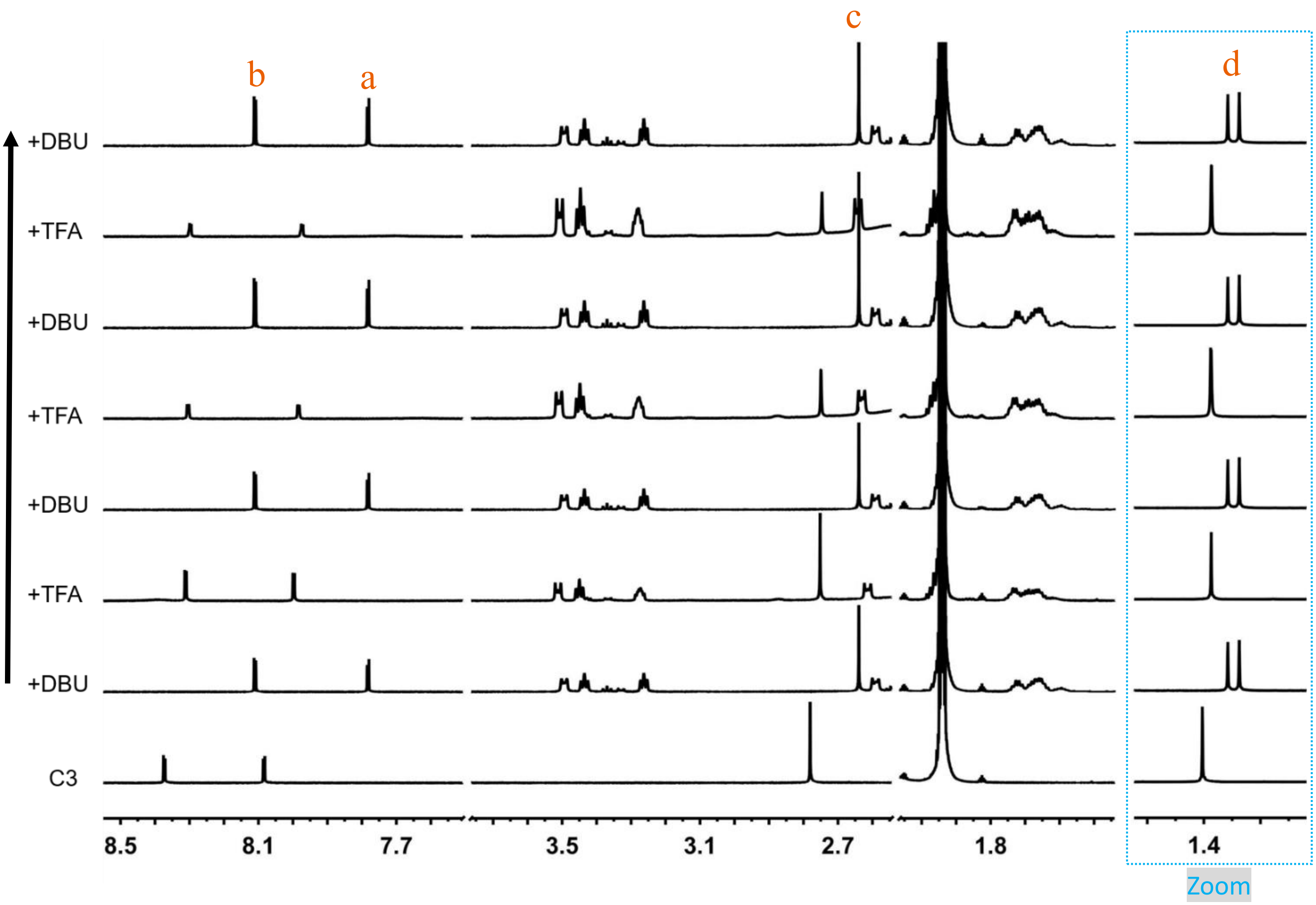

Supplementary Figure 124. Changes of ^{1}H NMR spectra (600 MHz, 298 K) of compound **3** (4 mM) in CD_3CN upon alternate addition of DBU and TFA. The arrow in the figure represents the sequence in addition.

Supplementary Method 8. Intertransformation between Stage-Stop and Stage-Li.

To a solution of **3** (4 mM, 0.5 mL) in acetonitrile-d_3, 2.0 equivalents of DBU (8 μL, 500 mM in acetonitrile-d_3) was added, giving rise to an acetonitrile-d_3 solution of deprotonated **3**. To the resultant solution, 1.0 equivalent of $LiClO_4$ (4 μL, 500 mM in acetonitrile-d_3) was added, followed the addition of 1.0 equivalent of tetraethylammonium fluoride (**TEAF**) (8 μL, 250 mM in acetonitrile-d_3; prepared by dissolving tetraethylammonium fluoride dihydrate into acetonitrile-d_3 then drying with 3A molecular sieve) to remove the Li^+ cations from the solution system. The cyclic chemical stimuli were repeated for 3 time. The whole process was monitored by a Bruker 500 MHz (^1H) spectrometer. The obtained ^1H NMR spectra are shown in Supplementary Fig. 125. 2D EXSY spectra relating to the base-regulated conditions are shown in Supplementary Fig. 126 – 129. Calculations of the exchange rates (k, s^{-1}) of the Li^+-regulated rotor were performed by line shape analysis (LSA) of the experimental ^1H NMR signals of the methyl protons, using the basic parameters obtained from ^1H NMR spectrum of **3** in the presence of 1.2 equivalents of DBU and 2.0 equivalents of $LiClO_4$ at 298 K (Supplementary Fig. 91). The exchange rates (k, s^{-1}) for the base-regulated rotor shown in Supplementary Fig. 125 were basically directly derived from the 2D EXSY spectra shown in Supplementary Fig. 126 – 129.

In another run for this experiment, with most of the conditions keeping the same as those in the previous run, an extra 0.3 equivalents of Li^+ and F^- ions was added in every Li^+/ F^-stimuli cycle. The results are shown in Supplementary Fig. 130 – 133, Supplementary Table 21 and Figure 5b in the main text. The results verified the slight hydrolysis of Li^+ cations at elevated temperature and, more importantly, showed that a reversible inter-transformation between Stage-Stop and Stage-Li could be achieved by addition of extra Li^+ cations in every Li^+/ F^-stimuli cycle. It is noteworthy that, due to the available spectrometer resources, the k value for **3** in the presence of only DBU (k = 1.41 s^{-1}) shown in Supplementary Fig. 130 was not directly measured but used the one derived from 2D EXSY spectrum shown in Supplementary Fig. 126.

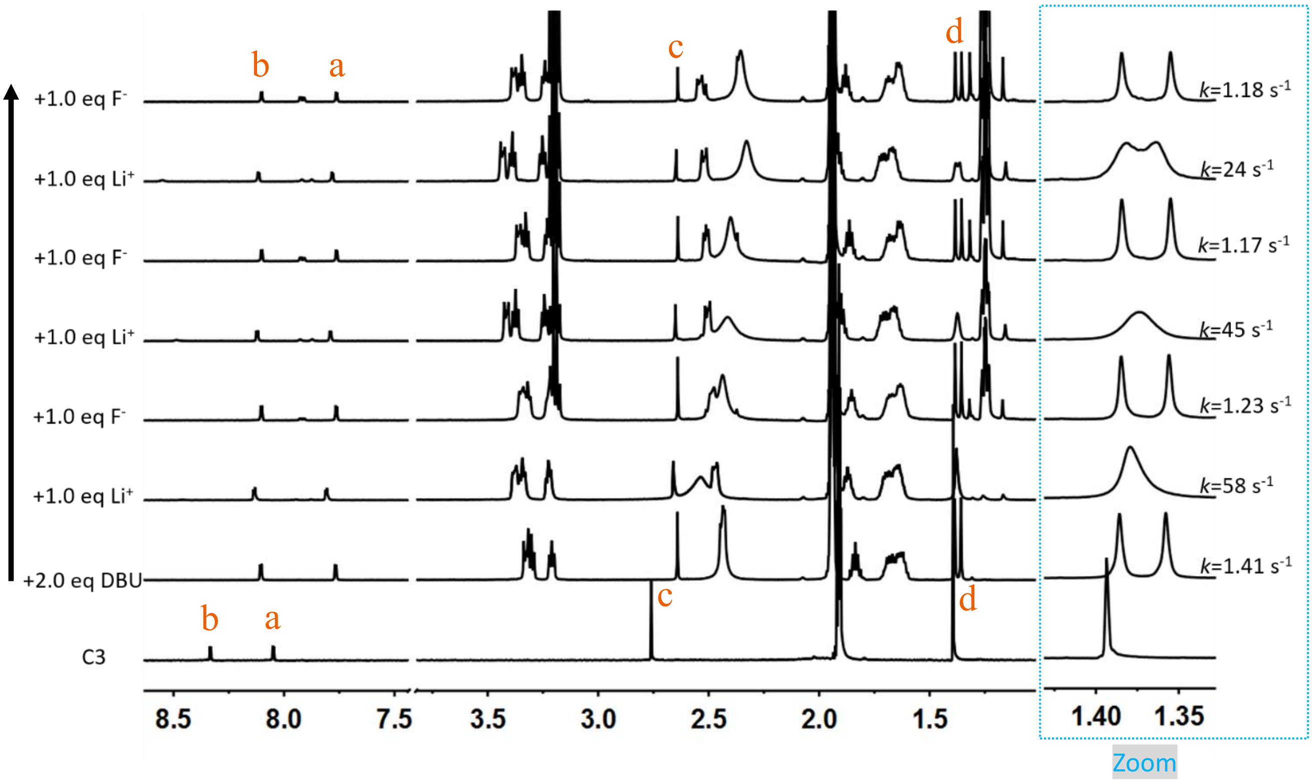

Supplementary Figure 125. Changes of ^{1}H NMR spectra (600 MHz, 338 K) of compound **3** (4 mM) in CD_3CN upon addition of DBU, followd the alternate addition of $LiClO_4$ and TEAF. The arrow in the figure represents the sequence in addition. The calculated exchange rates (k, s^{-1}) are given for each trace. The corresponding 2D EXSY spectra are shown in Supplementary Fig. 126 – 129.

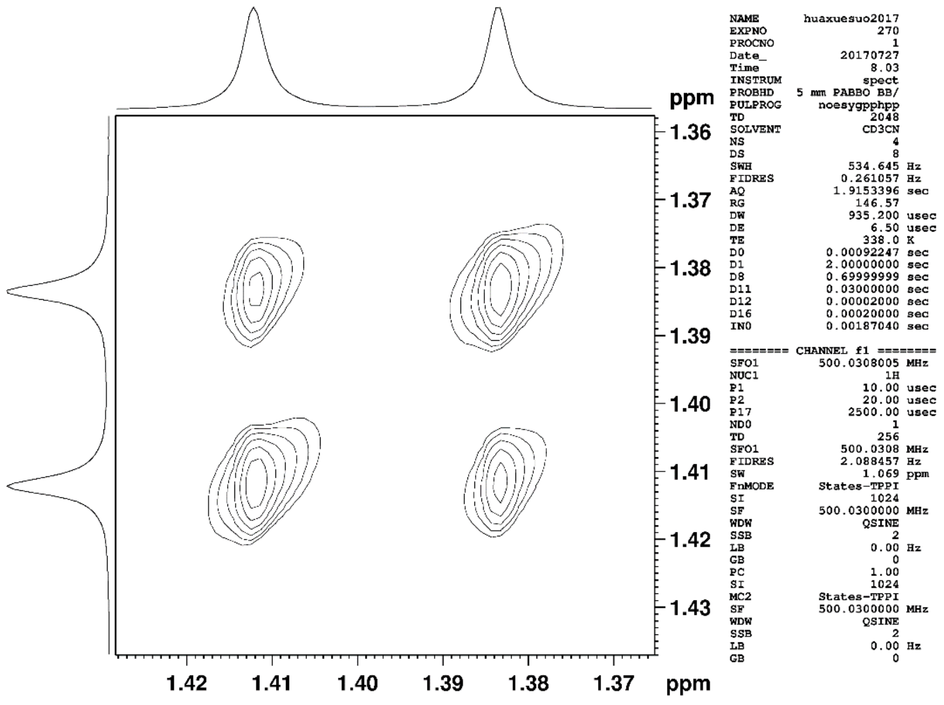

Supplementary Figure 126. EXSY NMR (500 MHz, t_m = 0.7 s) spectrum of **3-DBU** at 338 K in CD_3CN. [**3**] = 4 mM, [**DBU**] = 8 mM (2.0 equiv).

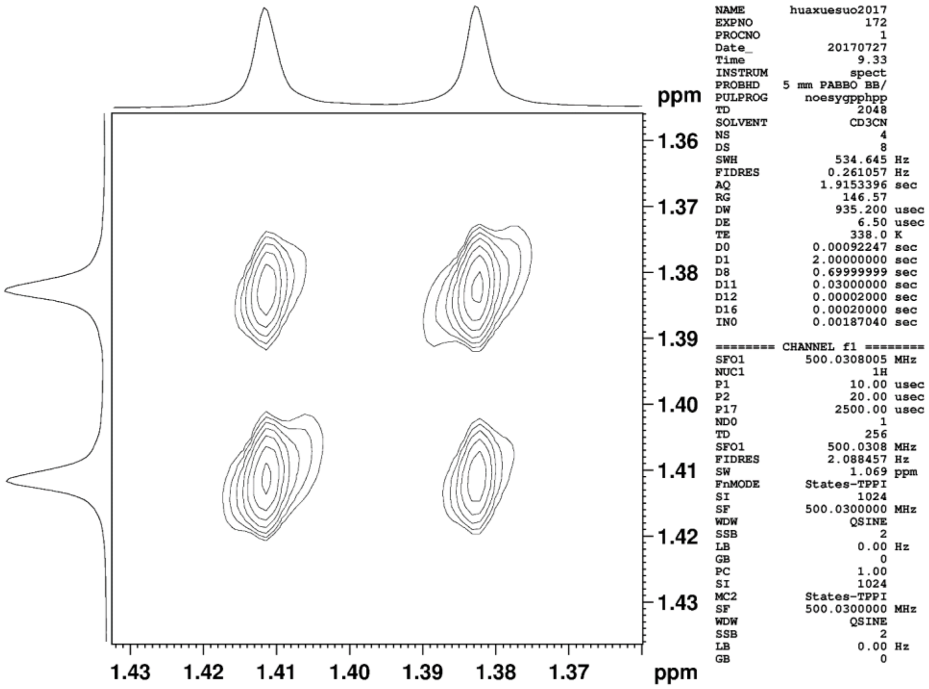

Supplementary Figure 127. EXSY NMR (500 MHz, t_m= 0.7 s) spectrum of **3→DBU→LiClO$_4$→TEAF** at 338 K in CD_3CN. [**3**] = 4 mM, [**DBU**] = 8 mM (2.0 equiv), [**LiClO$_4$**] = 4 mM (1.0 equiv), [**TEAF**] = 4 mM (1.0 equiv).

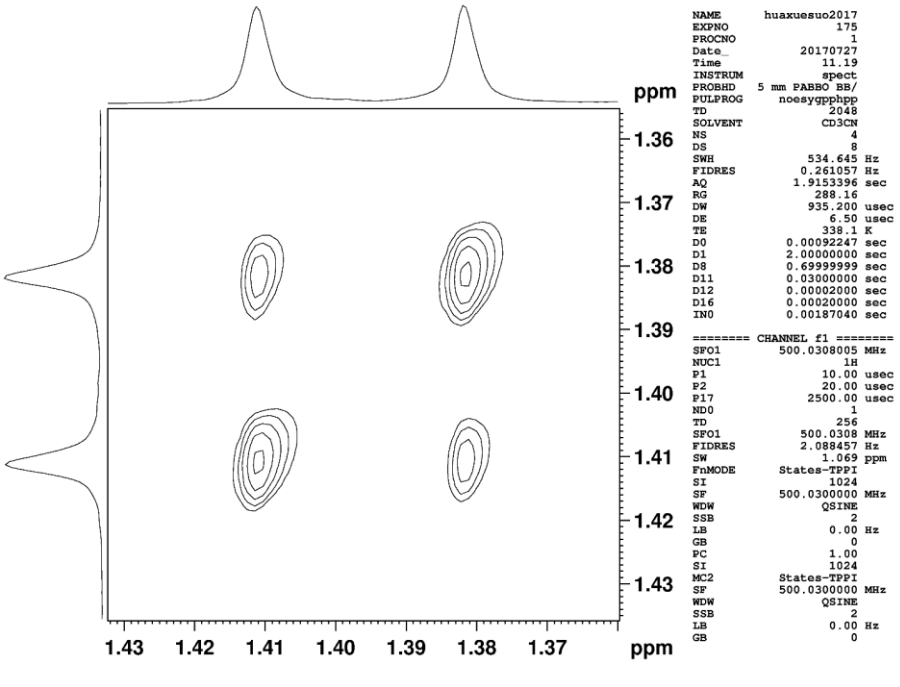

Supplementary Figure 128. EXSY NMR (500 MHz, t_m= 0.7 s) spectrum of **3→DBU→LiClO$_4$→TEAF→LiClO$_4$→TEAF** at 338 K in CD_3CN. [**3**] = 4 mM, [**DBU**] = 8 mM (2.0 equiv), [**LiClO$_4$**]$_{total}$ = 8 mM (2.0 equiv), [**TEAF**]$_{total}$ = 8 mM (2.0 equiv).

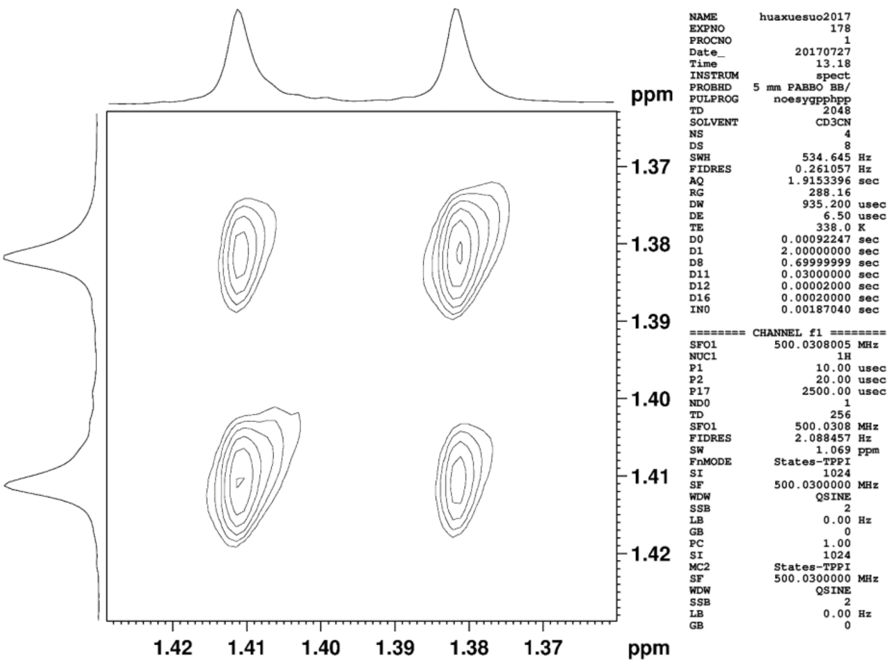

Supplementary Figure 129. EXSY NMR (500 MHz, t_m= 0.7 s) spectrum of **3→DBU→LiClO$_4$ →TEAF→LiClO$_4$→TEAF→LiClO$_4$→TEAF** at 338 K in CD$_3$CN. [**3**] = 4 mM, [**DBU**] = 8 mM (2.0 equiv), [**LiClO$_4$**]$_{total}$ = 12 mM (3.0 equiv), [**TEAF**]$_{total}$ = 12 mM (3.0 equiv).

Supplementary Table 20. The integrals derived from 2D EXSY NMR shown in Supplementary Fig. 126 – 129 and the corresponding calculated *k* values .

	I_A	I_B	I_{AB}	I_{BA}	*k* (s^{-1})
**3**→DBU	1.0000	0.9848	0.4579	0.4522	1.41
**3**→DBU→Li^+→F^-	1.0000	0.9873	0.4010	0.4039	1.23
**3**→DBU→Li^+→F^-→Li^+→F^-	1.0000	1.0103	0.3900	0.3909	1.17
**3**→DBU→Li^+→F^-→Li^+→F^-→Li^+→F^-	1.0000	1.0377	0.4030	0.3963	1.18

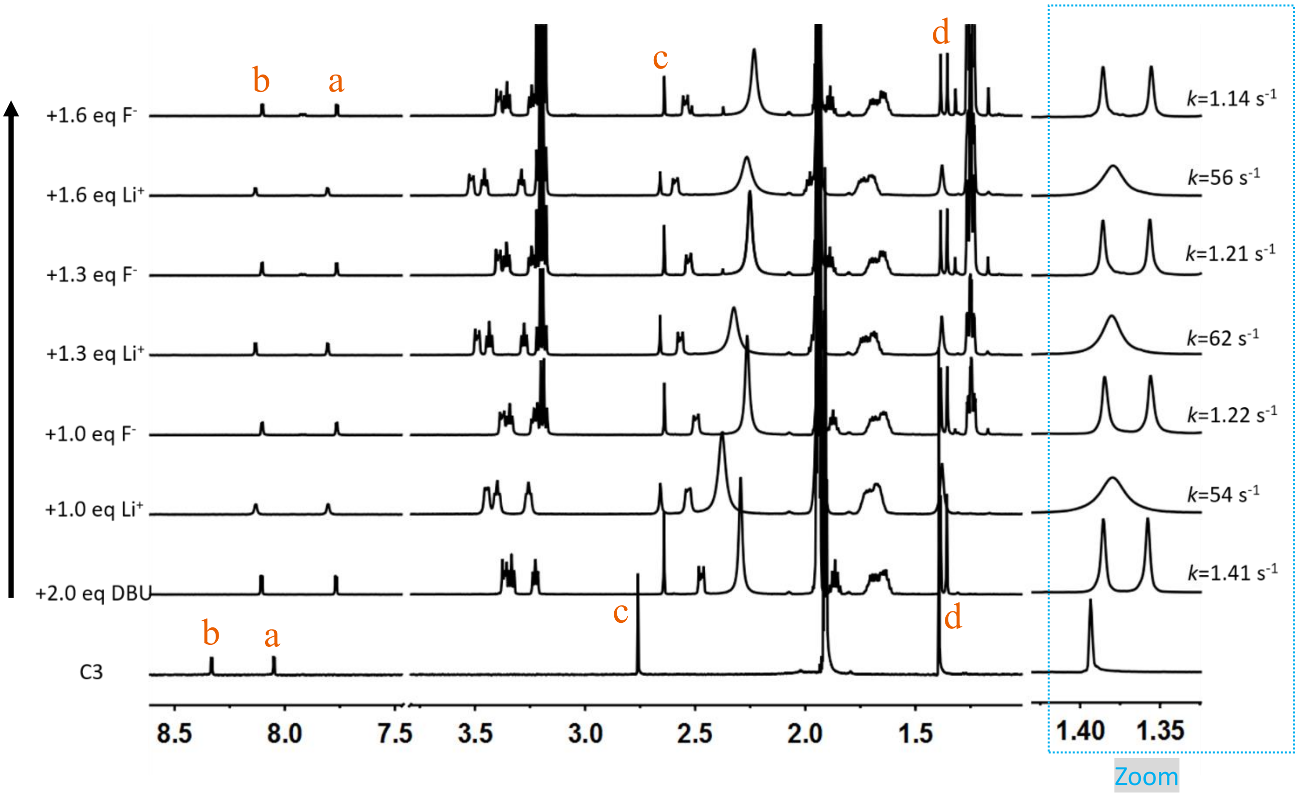

Supplementary Figure 130. Changes of ^{1}H NMR spectra (600 MHz, 338 K) of compound **3** (4 mM) in CD_3CN upon addition of DBU, followed the cyclic addition of $LiClO_4$ and TEAF. An extra 0.3 equivalents of $LiClO_4$ and TEAF were added in every followed cycle. The arrow in the figure represents the sequence in addition. The calculated exchange rates (k, s^{-1}) are given for each trace. The corresponding 2D EXSY spectra are shown in Supplementary Fig. 131 –133.

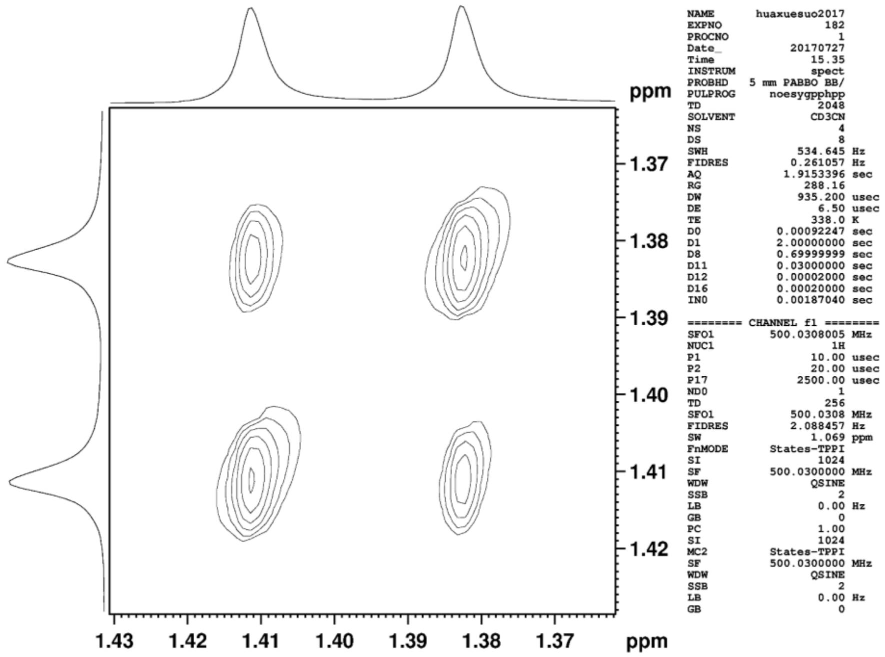

Supplementary Figure 131. EXSY NMR (500 MHz, t_m= 0.7 s) spectrum of **3→DBU→LiClO₄→TEAF** at 338 K in CD_3CN. [**3**] = 4 mM, [**DBU**] = 8 mM (2.0 equiv), $[\mathbf{LiClO_4}]_{total}$ = 4 mM (1.0 equiv), $[\mathbf{TEAF}]_{total}$ = 4 mM (1.0 equiv).

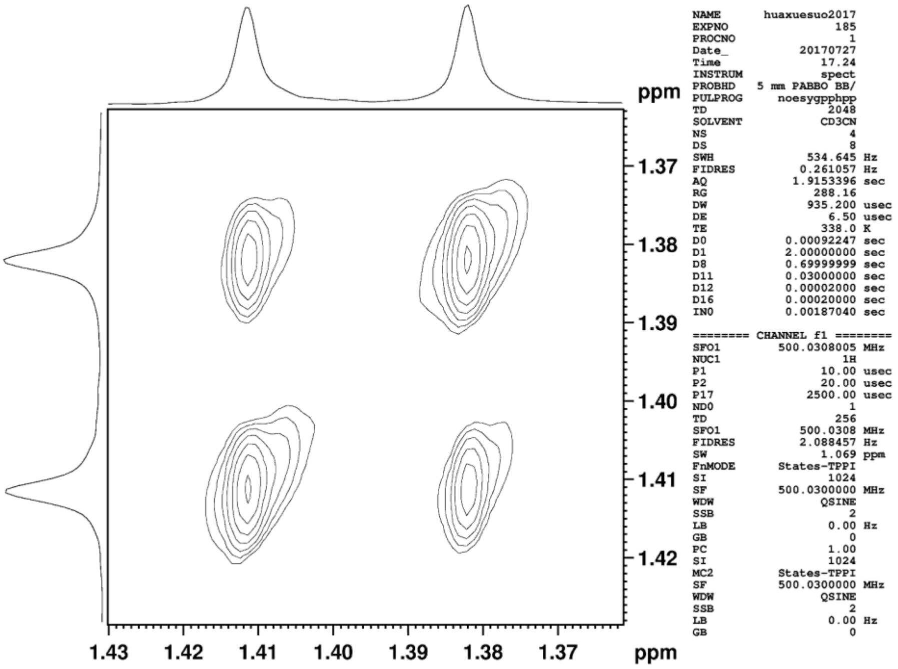

Supplementary Figure 132. EXSY NMR (500 MHz, t_m= 0.7 s) spectrum of **3→DBU→LiClO$_4$→TEAF→LiClO$_4$→TEAF** at 338 K in CD_3CN. [**3**] = 4 mM, [**DBU**] = 8 mM (2.0 equiv), [**LiClO$_4$**]$_{total}$ = 9.2 mM (2.3 equiv), [**TEAF**]$_{total}$ = 9.2 mM (2.3 equiv).

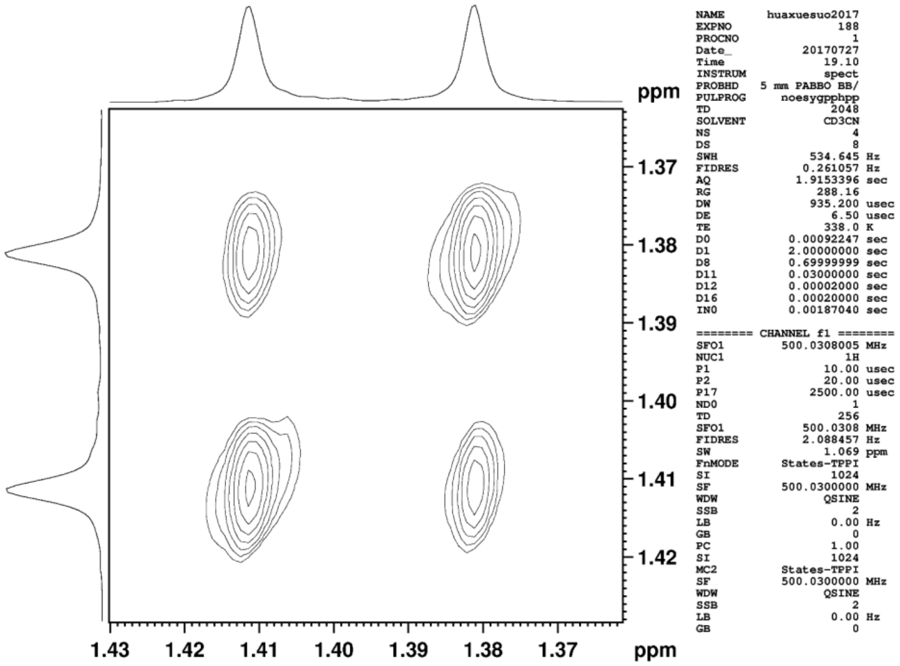

Supplementary Figure 133. EXSY NMR (500 MHz, t_m= 0.7 s) spectrum of **3→DBU→LiClO$_4$→TEAF→LiClO$_4$→TEAF→LiClO$_4$→TEAF** at 338 K in CD_3CN. [**3**] = 4 mM, [**DBU**] = 8 mM (2.0 equiv), [**LiClO$_4$**]$_{total}$ = 15.6 mM (3.9 equiv), [**TEAF**]$_{total}$ = 15.6 mM (3.9 equiv).

Supplementary Table 21. The integrals derived from 2D EXSY NMR shown in Supplementary Fig. 131 – 133 and calculated *k* values.

	**I_A**	**I_B**	**I_{AB}**	**I_{BA}**	***k*** **(s^{-1})**
**3**→DBU→Li^+→F^-	1.0000	1.0103	0.40711	0.40429	1.22
**3**→DBU→Li^+→F^-→1.3Li^+→1.3 F^-	1.0000	1.0015	0.39956	0.40222	1.21
**3**→DBU→Li^+→F^-→1.3Li^+→1.3F^- →1.6Li^+→1.6F^-	1.0000	1.0244	0.38569	0.38183	1.14

Supplementary Method 9. Intertransformations between Stage-Stop and Stage-Na.

To a solution of **3** (8 mM, 0.5 mL) in acetonitrile-*d*3 deprotonated with 2.0 equivalents of DBU (8 μL, 1000 mM in acetonitrile-*d*3), 1.0 equivalent of $NaClO_4$ (4 μL, 1000 mM in acetonitrile-*d*3) was added. To this solution, 1.0 equivalent of TEAF (4 μL, 1000 mM in acetonitrile-*d*3; prepared by dissolving tetraethylammonium fluoride dihydrate into acetonitrile-*d*3 then drying with 3A molecular sieve) was added. The sequential treatment of $NaClO_4$ and TEAF was further repeated for 2 times. The whole process was monitored by a Bruker 500 MHz (^1H) spectrometer. The obtained ^1H NMR spectra are shown in Supplementary Fig. 134. The corresponding 2D EXSY spectra are shown in Supplementary Fig. 135–140. Summaries of the integral parameters derived from 2D EXSY experiments and the calculated exchange rates (*k*, s^{-1}) are shown in Supplementary Table 22. The calculated exchange rates (*k*, s^{-1}) are also given for each trace on the ^1H NMR spectra shown in Supplementary Fig. 134. The *k* value for **3** in the presence of DBU only (k = 1.41 s^{-1}) shown in Supplementary Fig. 134 was used the one derived from 2D EXSY spectrum shown in Supplementary Fig. 126. Corresponding plots figure is shown in Fig. 5c in the main text.

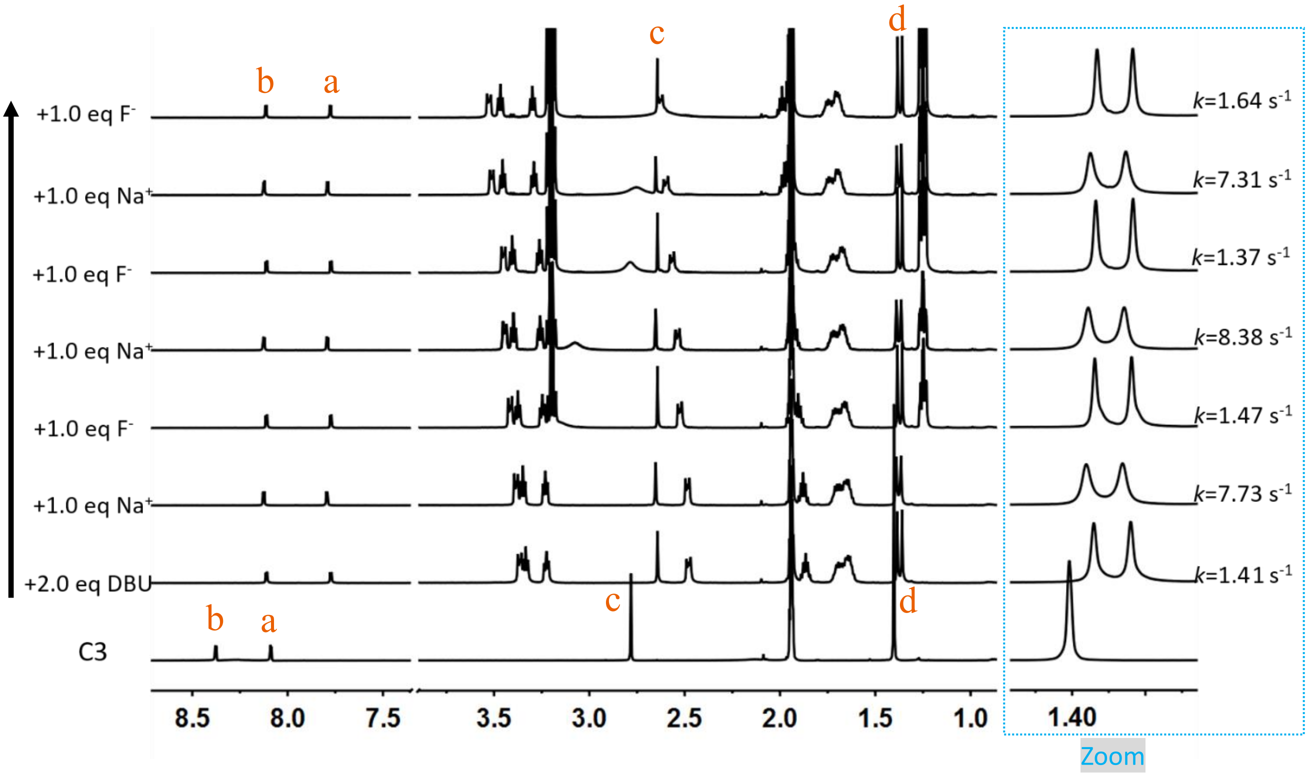

Supplementary Figure 134. Changes of ^{1}H NMR spectra (500 MHz, 338 K) of compound **3** (4 mM) in CD_3CN upon addition of DBU, followd the alternate addition of $NaClO_4$ and TEAF. The arrow in the figure represents the sequence in addition. The calculated exchange rates (k, s^{-1}) are given for each trace. The corresponding 2D EXSY spectra are shown in Supplementary Fig. 135 – 140.

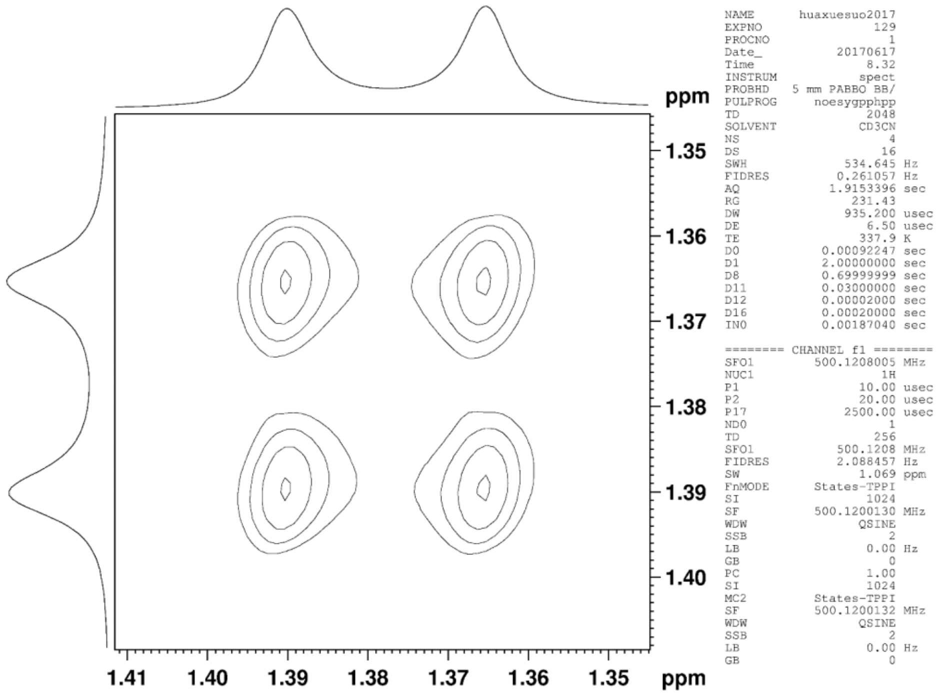

Supplementary Figure 135. EXSY NMR (500 MHz, t_m= 0.7 s) spectrum of **3→DBU→NaClO₄** at 338 K in CD_3CN. [**3**] = 4 mM, [**DBU**] = 8 mM (2.0 equiv), [**$NaClO_4$**] = 4 mM (1.0 equiv).

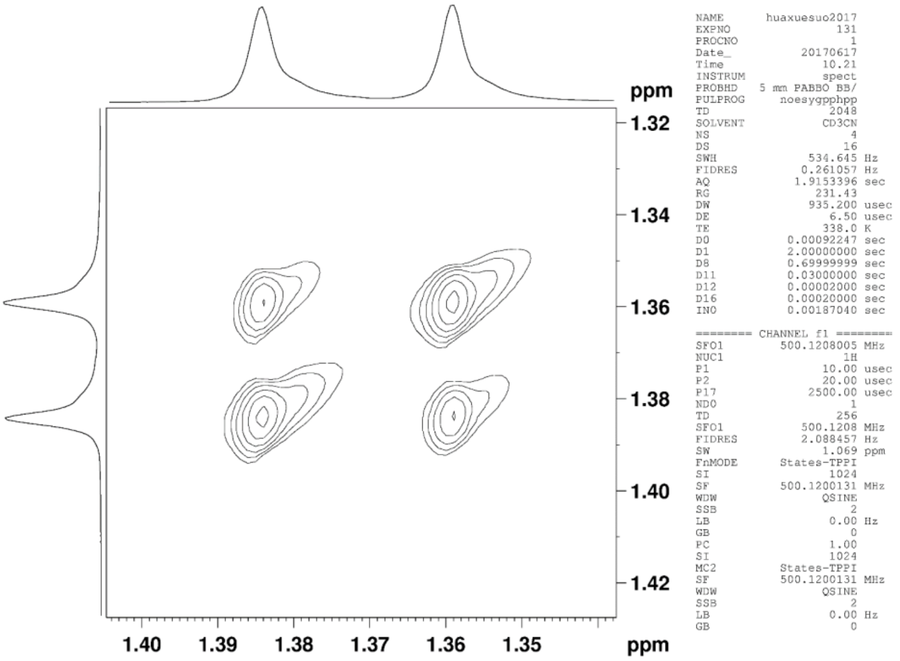

Supplementary Figure 136. EXSY NMR (500 MHz, t_m = 0.7 s) spectrum of **3→DBU→NaClO$_4$→TEAF** at 338 K in CD_3CN. [**3**] = 4 mM, [**DBU**] = 8 mM (2.0 equiv), [**NaClO$_4$**] = 4 mM (1.0 equiv), [**TEAF**] = 4 mM (1.0 equiv).

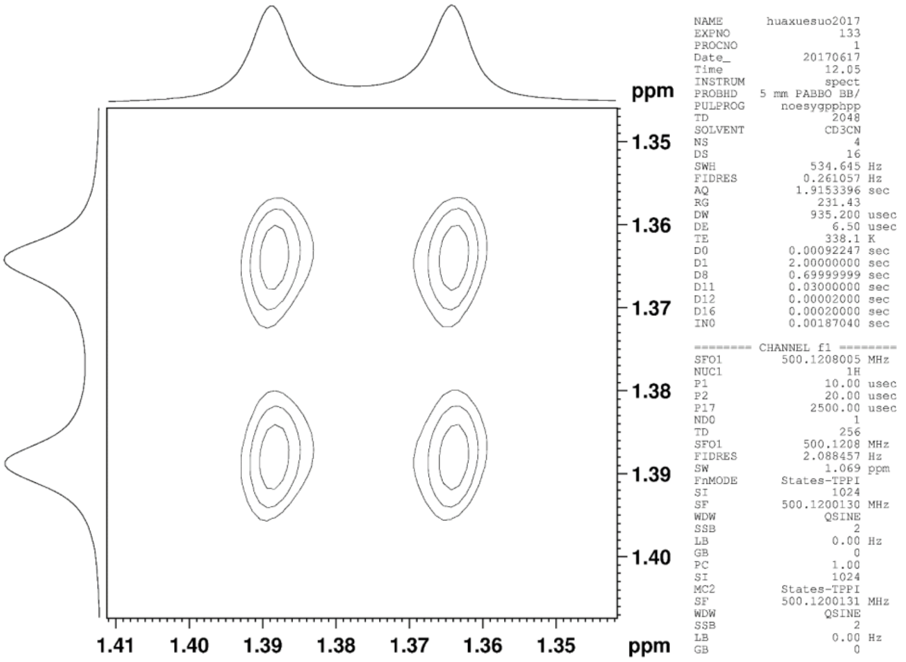

Supplementary Figure 137. EXSY NMR (500 MHz, t_m = 0.7 s) spectrum of **3→DBU→NaClO$_4$→TEAF→NaClO$_4$** at 338 K in CD_3CN. [**3**] = 4 mM, [**DBU**] = 8 mM (2.0 equiv), [**NaClO$_4$**]$_{total}$ = 8 mM (2.0 equiv), [**TEAF**] = 4 mM (1.0 equiv).

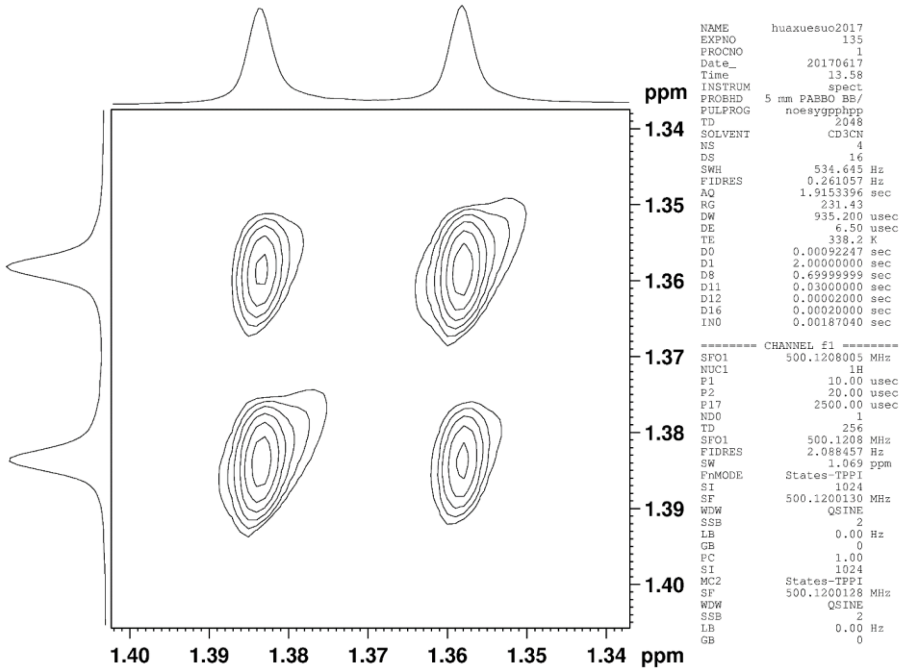

Supplementary Figure 138. EXSY NMR (500 MHz, t_m= 0.7 s) spectrum of **3→DBU→NaClO$_4$→ TEAF→NaClO$_4$→TEAF** at 338 K in CD_3CN. [**3**] = 4 mM, [**DBU**] = 8 mM (2.0 equiv), [**NaClO$_4$**]$_{total}$ = 8 mM (2.0 equiv), [**TEAF**]$_{total}$ = 8 mM (2.0 equiv).

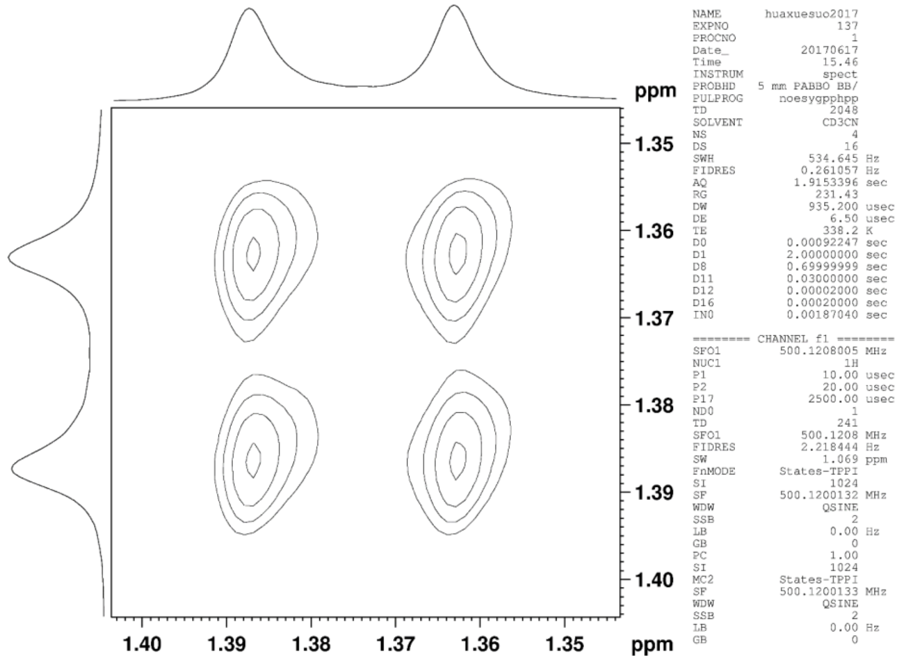

Supplementary Figure 139. EXSY NMR (500 MHz, t_m= 0.7 s) spectrum of **3→DBU→NaClO$_4$→ TEAF→NaClO$_4$→TEAF→NaClO$_4$** at 338 K in CD_3CN. [**3**] = 4 mM, [**DBU**] = 8 mM (2.0 equiv), [**NaClO$_4$**]$_{total}$ = 12 mM (3.0 equiv), [**TEAF**]$_{total}$ = 8 mM (2.0 equiv).

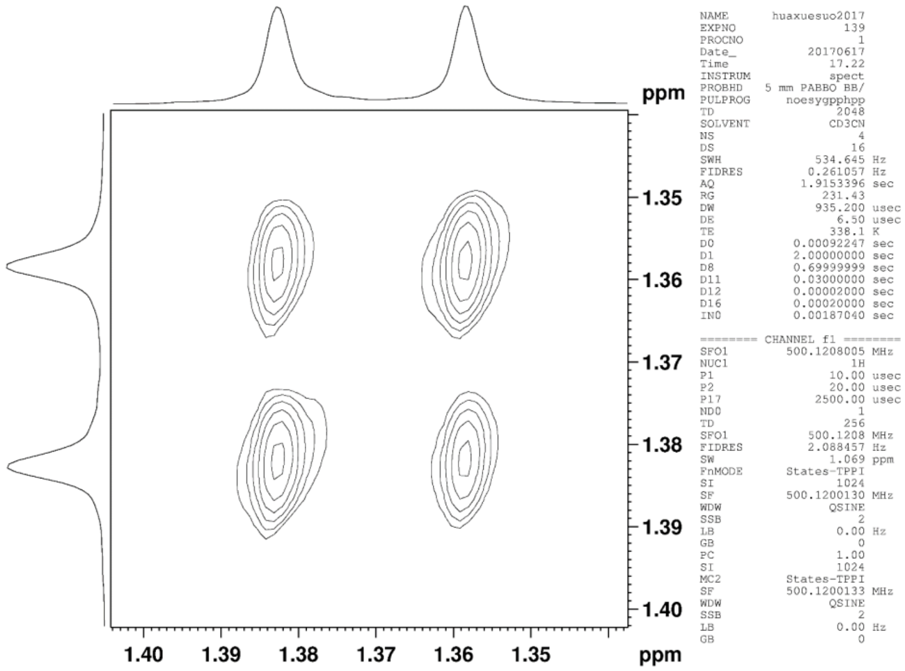

Supplementary Figure 140. EXSY NMR (500 MHz, $t_m = 0.7$ s) spectrum of **3→DBU→NaClO$_4$→TEAF→NaClO$_4$→TEAF→NaClO$_4$→TEAF** at 338 K in CD_3CN. [**3**] = 4 mM, [**DBU**] = 8 mM (2.0 equiv), [**NaClO$_4$**]$_{total}$ = 12 mM (3.0 equiv), [**TEAF**]$_{total}$ = 12 mM (3.0 equiv).

Supplementary Table 22. The integrals derived from 2D EXSY NMR shown in Supplementary Fig. 135 – 140 and calculated *k* values.

	I_A	I_B	I_{AB}	I_{BA}	k (s^{-1})
**3**→DBU→Na^+	1.0000	0.9813	0.9777	0.9859	7.73
**3**→DBU→Na^+→F^-	1.0000	1.0015	0.4685	0.4806	1.47
**3**→DBU→Na^+→F^-→Na^+	1.0000	0.9885	0.9699	1.0073	8.38
**3**→DBU→Na^+→F^-→Na^+→F^-	1.0000	0.9976	0.4435	0.4491	1.37
**3**→DBU→Na^+→F^-→Na^+→F^-→Na^+	1.0000	1.0106	0.9828	1.0039	7.31
**3**→DBU→Na^+→F^-→Na^+→F^-→Na^+→F^-	1.0000	0.9968	0.5135	0.5203	1.64

Supplementary Method 10. Intertransformations between Different Mid-speed Rotations.

Interconversion between Stage-Na and Stage-Li was verified by very similar experiments as that in the case between Stage-Stop and Stage-Li. To a solution of **3** (8 mM, 0.5 mL) in acetonitrile-d_3, 2.0 equivalents of DBU (8 μL, 1000 mM in acetonitrile-d_3) was added. To this solution, $NaClO_4$ (1.0 equivalent, 4 μL, 1000 mM in acetonitrile-d_3) was added, followed the addition of TEAF (1.0 equivalent, 4 μL, 1000 mM in acetonitrile-d_3; prepared by dissolving tetraethylammonium fluoride dihydrate into acetonitrile-d_3, then drying with 3A molecular sieve) to precipitate the Na^+ cations. $LiClO_4$ (1.0 equivalent, 4 μL, 1000 mM in acetonitrile-d_3) and TEAF (1.0 equivalent, 4 μL, 1000 mM in acetonitrile-d_3; dried with 3A molecular sieve) were added in sequence. The $Na^+ \rightarrow F^- \rightarrow Li^+ \rightarrow F^-$ chemical stimuli cycle was repeated for totally 2 times. The whole process was monitored by a Bruker 500 MHz (^{1}H) spectrometer. The obtained ^{1}H NMR spectra are shown in Supplementary Fig. 141. 2D EXSY spectra relating to Na^+- and base-regulated conditions are shown in Supplementary Fig. 142 – 147. Calculations of the exchange rates (k, s^{-1}) of the Li^+-regulated rotor were performed by line shape analysis of the experimental ^{1}H NMR signals of the methyl protons, using the basic parameters obtained from ^{1}H NMR spectrum of **3** in the presence of 1.2 equivalents of DBU and 2.0 equivalents of $LiClO_4$ at 338 K (Supplementary Fig. 91). The exchange rates (k, s^{-1}) for the Na^+- and base-regulated rotor were directly derived from the corresponding 2D EXSY spectra of the sample, except that the one for **3** in the presence of DBU only was derived from 2D EXSY spectrum shown in Supplementary Fig. 126. These exchange rates (k, s^{-1}) are shown in Supplementary Fig. 141 and Supplementary Table 23. Corresponding plots figure is shown in Fig. 5d in the main text.

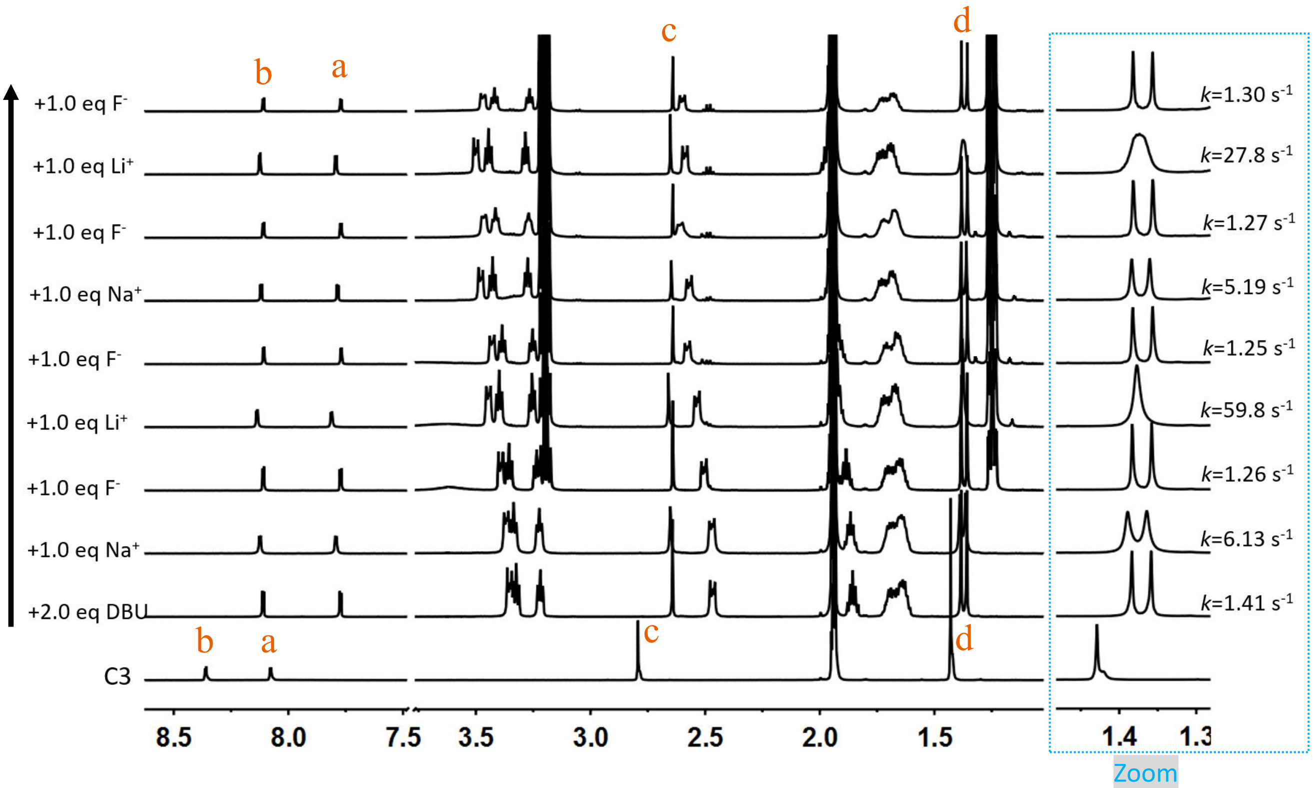

Supplementary Figure 141. Changes of ^{1}H NMR spectra (500 MHz, 338 K) of compound **3** (8 mM) in CD_3CN upon addition of DBU, followed the addition of $NaClO_4$, TEAF, $LiClO_4$, TEAF, $NaClO_4$, TEAF, $LiClO_4$ and TEAF in sequence. The arrow in the figure represents the sequence in addition. The calculated exchange rates (k, s^{-1}) are given for each trace. The corresponding 2D EXSY spectra are shown in Supplementary Fig. 142 – 147.

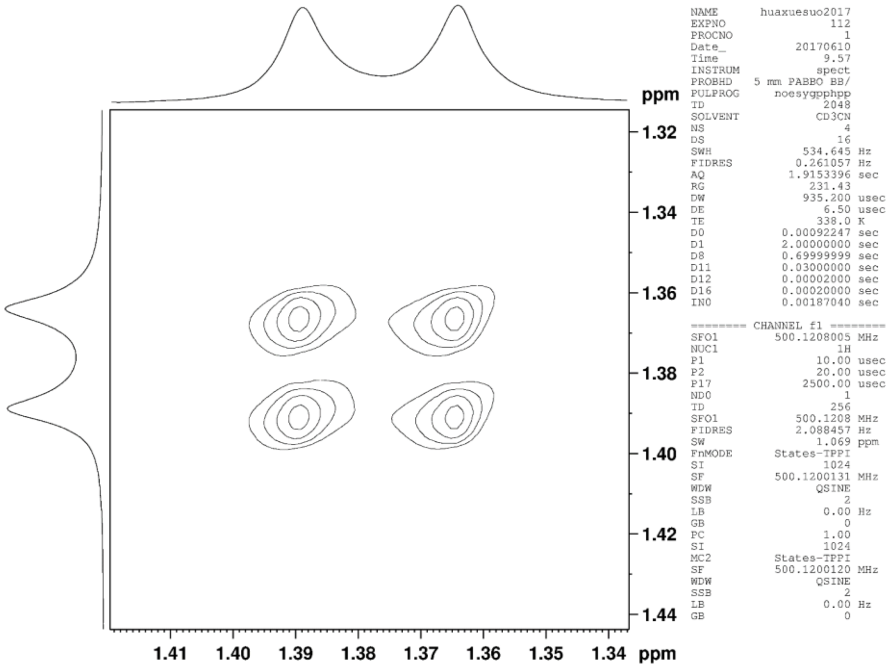

Supplementary Figure 142. EXSY NMR (500 MHz, t_m = 0.7 s) spectrum of **3→DBU→NaClO₄** at 338 K in CD_3CN. [**3**] = 4 mM, [**DBU**] = 8 mM (2.0 equiv), [$\mathbf{NaClO_4}$] = 4 mM (1.0 equiv).

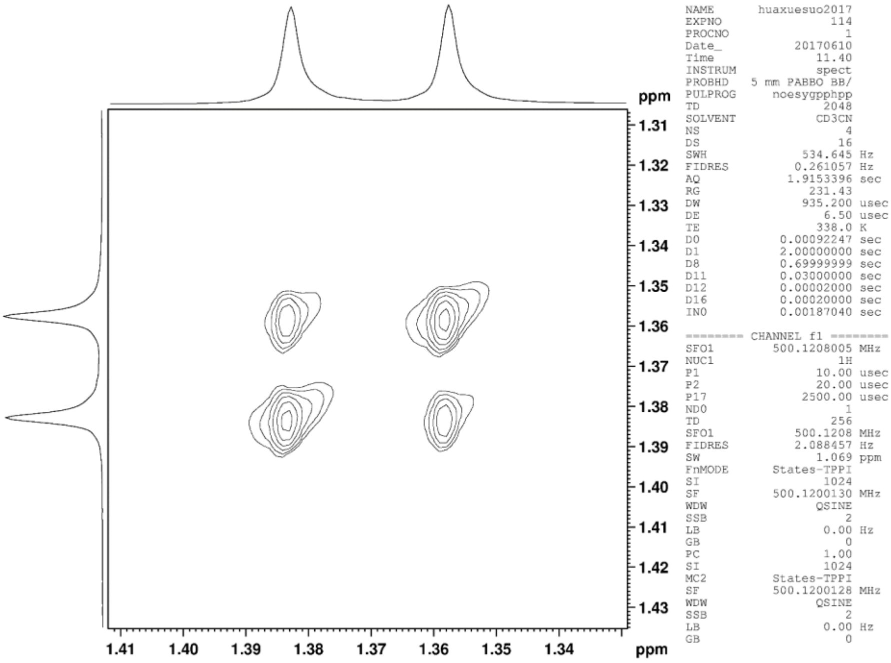

Supplementary Figure 143. EXSY NMR (500 MHz, t_m = 0.7 s) spectrum of **3→DBU→NaClO$_4$→ TEAF** at 338 K in CD_3CN. [**3**] = 4 mM, [**DBU**] = 8 mM (2.0 equiv), [**NaClO$_4$**] = 4 mM (1.0 equiv), [**TEAF**] = 4 mM (1.0 equiv).

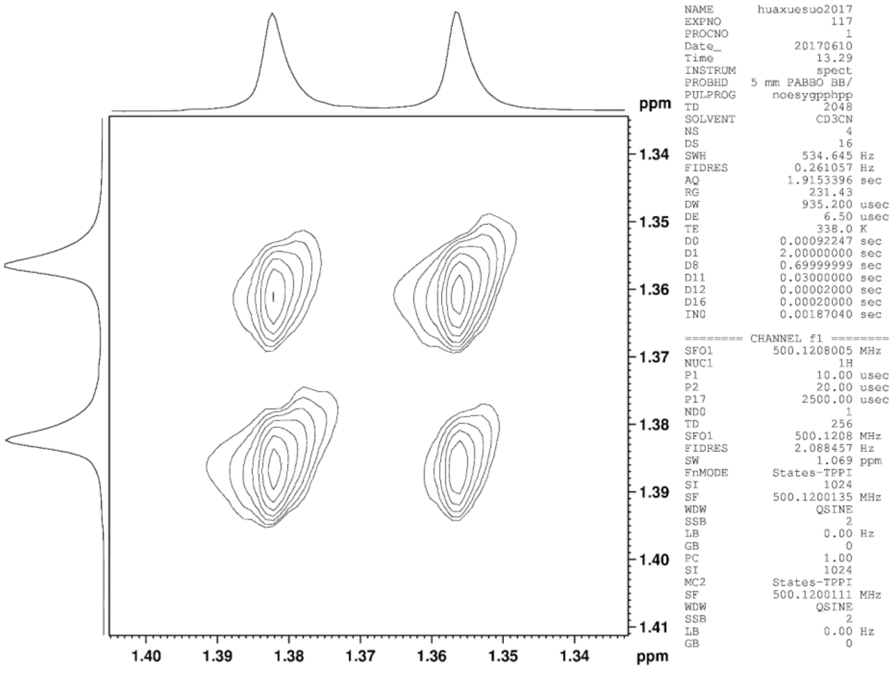

Supplementary Figure 144. EXSY NMR (500 MHz, t_m= 0.7 s) spectrum of **3→DBU→NaClO$_4$→ TEAF→LiClO$_4$→TEAF** at 338 K in CD_3CN. [**3**] = 4 mM, [**DBU**] = 8 mM (2.0 equiv), [**NaClO$_4$**] = 4 mM (1.0 equiv), [**TEAF**]$_{total}$ = 8 mM (2.0 equiv), [**LiClO$_4$**] = 4 mM (1.0 equiv).

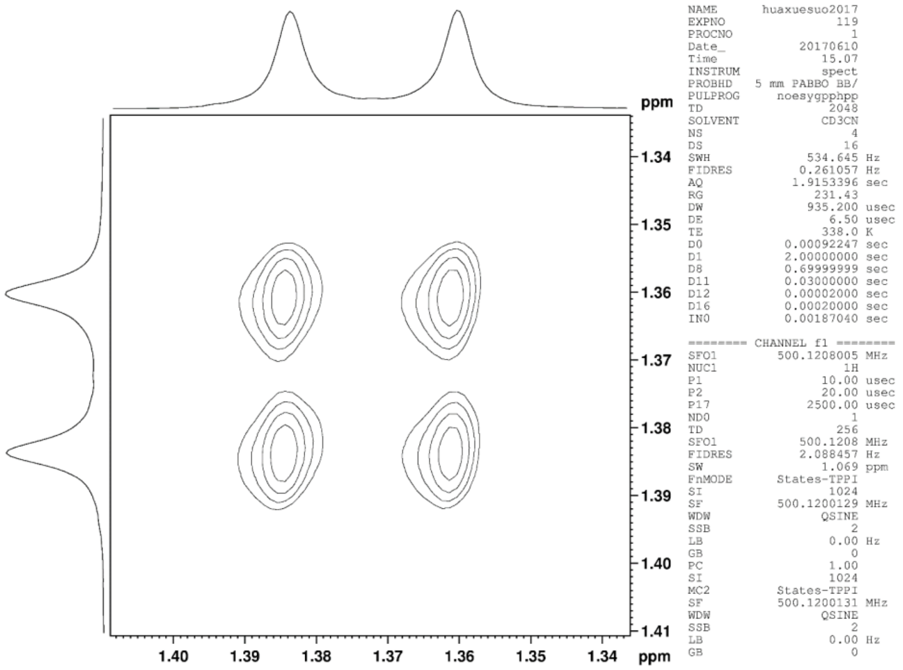

Supplementary Figure 145. EXSY NMR (500 MHz, t_m = 0.7 s) spectrum of **3→DBU→NaClO$_4$→ TEAF→LiClO$_4$→TEAF→NaClO$_4$** at 338 K in CD_3CN. [**3**] = 4 mM, [**DBU**] = 8 mM (2.0 equiv), [**NaClO$_4$**]$_{total}$ = 8 mM (2.0 equiv), [**TEAF**]$_{total}$ = 8 mM (2.0 equiv), [**LiClO$_4$**] = 4 mM (1.0 equiv).

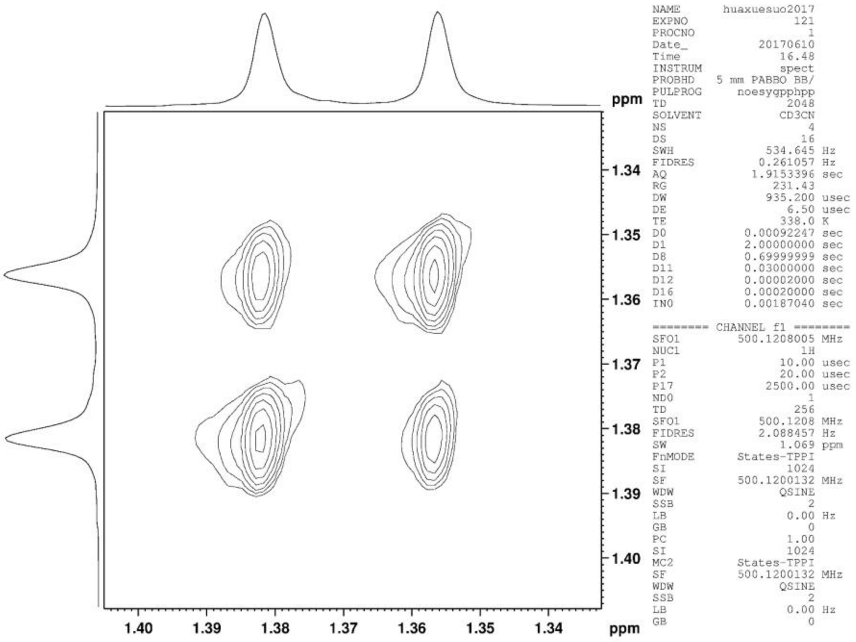

Supplementary Figure 146. EXSY NMR (500 MHz, t_m= 0.7 s) spectrum of **3→DBU→NaClO$_4$→ TEAF→LiClO$_4$→TEAF→NaClO$_4$→TEAF** at 338 K in CD_3CN. [**3**] = 4 mM, [**DBU**] = 8 mM (2.0 equiv), [**NaClO$_4$**]$_{total}$ = 8 mM (2.0 equiv), [**TEAF**]$_{total}$ = 12 mM (3.0 equiv), [**LiClO$_4$**] = 4 mM (1.0 equiv).

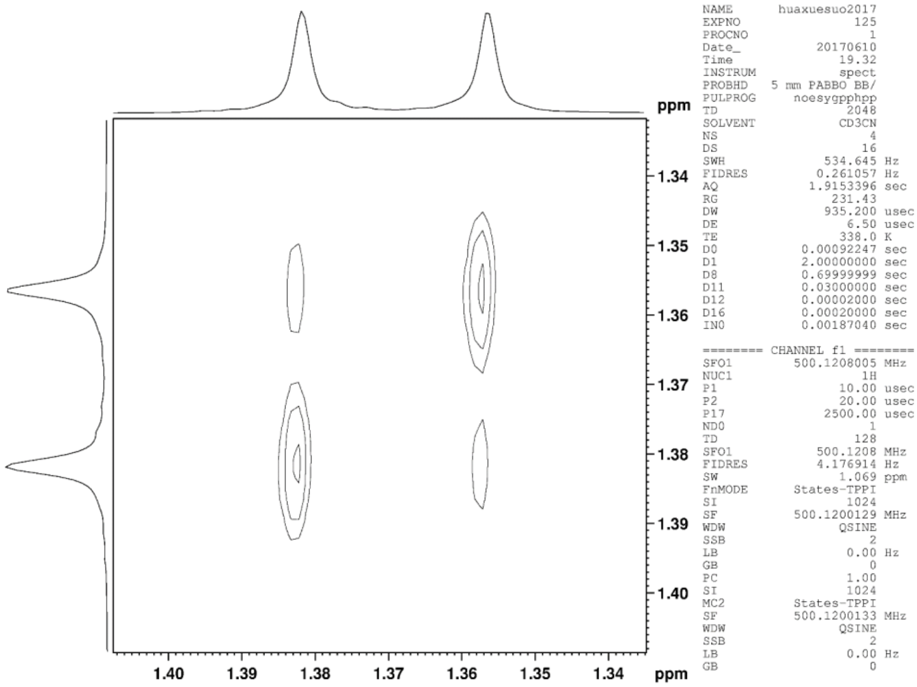

Supplementary Figure 147. EXSY NMR (500 MHz, t_m= 0.7 s) spectrum of **3→DBU→NaClO$_4$→ TEAF→LiClO$_4$→TEAF→NaClO$_4$→TEAF→LiClO$_4$→TEAF** at 338 K in CD_3CN. [**3**] = 4 mM, [**DBU**] = 8 mM (2.0 equiv), [**NaClO$_4$**]$_{total}$ = 8 mM (2.0 equiv), [**TEAF**]$_{total}$ = 16 mM (4.0 equiv), [**LiClO$_4$**]$_{total}$ = 8 mM (2.0 equiv).

Supplementary Table 23. The integrals derived from 2D EXSY NMR shown in Supplementary Fig. 142 – 147 and calculated *k* values.

	I_A	I_B	I_{AB}	I_{BA}	*k* (s^{-1})
**3**→DBU→Na^+	1.0000	1.0833	1.0572	0.9700	6.13
**3**→DBU→Na^+→$F^–$	1.0000	1.0263	0.4487	0.3923	1.26
**3**→DBU→Na^+→$F^–$→Li^+→$F^–$	1.0000	1.0434	0.4517	0.3871	1.25
**3**→DBU→Na^+→$F^–$→Li^+→$F^–$→Na^+	1.0000	1.0688	1.0088	0.9536	5.19
**3**→DBU→Na^+→$F^–$→Li^+→$F^–$→Na^+→$F^–$	1.0000	1.0384	0.4550	0.3980	1.27
**3**→DBU→Na^+→$F^–$→Li^+→F^-→Na^+→$F^–$ →Li^+→$F^–$	1.0000	1.0032	0.4396	0.4144	1.30

Supplementary Method 11. Intertransformations between a High-speed and Mid-speed Rotations.

As a representative example, studies on interconversion between Stage-HS and Stage-Li were carried out to verify the reversibility for transformations between a high-speed and mid-speed rotations. To a solution of **3** (4 mM, 0.5 mL) in acetonitrile-*d*3, 1.0 equivalents of DBU (4 μL, 500 mM in acetonitrile-*d*3) and 2.0 equivalent of $LiClO_4$ (8 μL, 500 mM in acetonitrile-*d*3) was added to give a Li^+-regulated rotor sytem. The solution was neutralized with 1.0 equivalent of TFA (4 μL, 500 mM in acetonitrile-*d*3), followed the basification with 1.0 equivalent of DBU (4 μL, 500 mM in acetonitrile-*d*3). The acid-base stimuli cycle was repeated for totally 2 times. The whole process was monitored by a Bruker 500 MHz (^{1}H) spectrometer. The obtained ^{1}H NMR spectra are shown in Supplementary Fig. 148. Considering the highly-reversible speed transformations could be easily verified by the reversible changes in the ^{1}H NMR spectrum pattern in the cyclic stimuli process, corresponding dynamic parameters relating to each trace are not calculated.

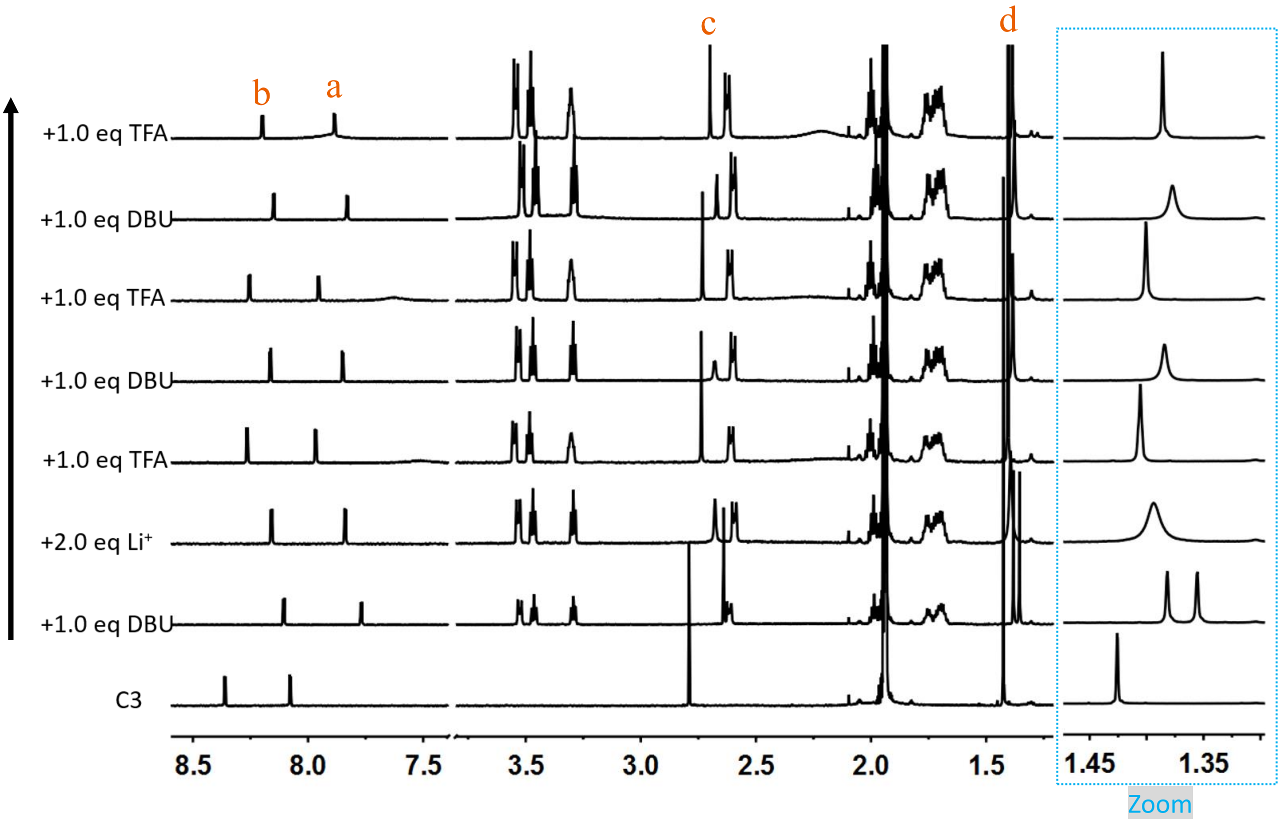

Supplementary Figure 148. Changes of ^{1}H NMR spectra (500 MHz, 338 K) of compound **3** (4 mM) in CD_3CN upon addition of DBU and $LiClO_4$, followd the alternate addition of TFA and DBU. The arrow in the figure represents the sequence in addition.

Supplementary Method 12. Computational Details.

DFT calculations were performed by the Gaussian 09 program package[6] in Linux operating system. All calculations were carried out using the B3LYP functional with the 6-31+G(d,p) basis set, and 12 processing cores with 24 GB physical memory (National Supercomputing Center−Shenzhen, China or Computing Center, College of Chemistry, Beijing Normal University).

(1) The Minima and the Transition States of 1 − 3

Ground states of 1−3. We utilized DFT to explore the ground state conformations of **1−3** by comparing the energy of several local minimum conformations. Starting from the X-ray structures, the unconstrained geometries of **1−3** were first optimized at B3LYP//6-31+G(d,p)//IEF-PCM-UFF levels of theory, using the default (Gaussian 09) polarizable continuum model for acetonitrile, acetone, and dichloromethane, respectively. Frequencies were also calculated for every conformation to ensure that each stationary point represented a minimum on the potential energy surface. All the optimized geometries of **1−3** (geometries denoted **1−3perpend**, Supplementary Table 24) closely resemble their corresponding single crystal geometries (Supplementary Table 24 and Table 26), except that the succinimide skeletons in **1** exhibit a more coplanar arrangement on the plane perpendicular to the one of the phenolic group. To confirm the optimized geometries to the global minima and estimate the stability of the global minimum conformation, we also examined other two conformations in which the planes of succinimide rotator and phenol stator are skewed so that the carbonyl O and the hydroxyl group could be close enough to form a H-bond between them. These two geometric isomers have a different arrangement of the 2,2-dimethylsuccinimide group: one possesses the dimethyl groups located at the same side as the hydroxyl group (denoted **1−3cis**, Supplementary Table 24); the another one has the dimethyl groups oriented in the opposite direction (denoted **1−3trans**, Supplementary Table 24). It is noteworthy that, a pair of enantiomers involve in all above cases (For example, there is an enantiomer of **1−perpend** where the atoms have the same spatial orientations as **1−perpend** except the hydroxyl group point to the left). In our studies, only one of enantiomers are calculated. The calculated results are summarized in Supplementary Table 25.

In polar solvent acetonitrile (dielectric constant, ε = 37)[7] and acetone (ε = 21)[7], the perpendicular conformation has been confirmed to the global minima for all the molecules **1−3**, whereas the skewed conformations **1−3cis** and **1−3trans** are calculated to be the local minima. Given a skewed conformation is not favorable for accommodating the sterics, intramolecular H-bonding should play a

very important role in decreasing the energy of **1−3cis** and **1−3trans**. In all cases, the energy gap between global minima conformation and the skewed conformations are pretty small (<1 kcal mol^{-1}), indicating that the rotors could at least oscillate unhindered between these conformations. On the other hand, the energy gaps are slightly different among **1−3**, which might be ascribed to a combined influence of the substituent and the solvation effects. For example, while the perpendicular conformation of **3** is ca. 0.6 kcal mol^{-1} lower in energy than that of the two skewed conformations, the three minima conformations of **2** locate at almost the same energy level. Besides, in all the cases of **1−3**, the two skewed conformations are almost isoenergetic, suggesting that there is very small influence of methyl groups on the intramolecular H-bonding.

The strength of H-bonding is reciprocally dependent on the dielectric constant of the solvent, becoming stronger in less polar solvent. In chloroform (ε = 4.8)[7], since that the solvation effect attenuates, the intramolecular H-bonding is more effective to stabilize the skewed conformations. Consequently, the skewed conformations are lightly lower in energy than the perpendicular one for **1** and **2**. For molecular **3**, the perpendicular geometry represents still the ground state conformation but the energy gap between it and the skewed conformations is slightly smaller than that in the cases of acetonitrile and acetone.

Transition states of 1−3. To obtain a good initial guess on the internal coordinate of the transition state (TS) conformations of **1−3**, calculation on the most energetically favorable conformational transformation pathway (MEP) for the rotation of the rotors were first roughly carried out. From one of the point of the skewed conformations, the MEPs were obtained by relaxed potential energy surface scan procedures as a function of the dihedral angle between the planes of the phenol and the succinimide ring. The highest energy conformations, in which the molecular skeletons are almost planer, were further optimized by conventional TS optimizations at B3LYP//6-31G(d,p)//IEF-PCM-UFF levels of theory. In all cases, the obtained geometry was confirmed to the TS conformation by frequency analysis, which yielded only one imaginary frequency corresponding to the relatively rotational motion of succinimide along the axle of the C(phenol)−N(amide) bond (Supplementary Table 24 and 25).

Ascribed to the asymmetrical structure of the compounds, two main TS conformations where the succinimide is coplanar with the phenol ring are obtained, with the difference in the direction of orientation of dimethyl groups with respective to that of the hydroxyl group (denoted **1−3TS-cis** and

1−3TS-trans, Supplementary Table 24) between them. Again, these two conformations possess a very close energy due to the weak substituent effect of methyl groups. TS conformations present in MEP between **1−3perpend** and **1−3cis** (or **1−3trans**) were not calculated.

(2) The GS and the TS of Complexes of (3–H)$^{–}$ with Metal Cations.

To theoretically illustrate the effect of metal cation on the activation energy of rotation of the protonated rotors, the minima, the intermediate and the transition state geometries identification for isolated deprotonated **3**, i.e., **(3–H)$^{–}$**, in the absence and the presence of three kinds of alkali metal cations, including Li^{+}, Na^{+} and K^{+}, in acetonitrile were carried out. The systems of **(3–H)$^{–}$** in the presence of alkali metal cations are generally denoted **(3–H)$^{–}$·M^{+}** (M^{+} = Li^{+}, Na^{+}, and K^{+}). It is noteworthy that, in the absence of metal cations, the system possesses a net charge of –1.

The minima and intermediates were first obtained by full system optimization at the DFT/B3LYP level using 6-31G(d,p) basis set and IEF-PCM-UFF solvent model for acetonitrile. The transition state (TS) geometries was optimized by conventional TS optimizations at the same level of theory. Frequencies were also calculated for the optimized structures to ensure that each stationary point truly represented a minimum or a saddle point on the potential energy surfaces. In cases of the presence of a pair of enantiomers, only one isomer was calculated.

Calculation on **(3–H)$^{–}$** would give us some idea about how much is the energy gap between the ground state conformation and a TS one for which there is completely no intramolecular interaction to stabilize the TS. The result, 21.9 kcal $mol^{–1}$ (Supplementary Table 28), is close to the experimental value for that of the system of the DBU-deprotonated **3** (19.6 kcal $mol^{–1}$, Table 1 in the main text).

In all cases of **(3–H)$^{–}$·M^{+}**, the TS of the rotors displays an approximate planar geometry, which is stabilized by an O(phenol)···M^{+}···O(carbonyl) bridge (Supplementary Table 27). Besides, DFT-calculations also predict a radius-dependent capability for the cations of decreasing the rotational barrier of **(3–H)$^{–}$**. Correspondingly, the distance between metal cation and the oxygens on the ligand is also cation-radius-dependent, increasing in the order of $Li^{+} < Na^{+} < K^{+}$ (Fig. 4a and Supplementary Table 28).

All the obtained optimized geometries are illustrated and summarized in Supplementary Table 27; the corresponding relative energies, imaginary and lowest vibrational frequencies, and some important structural parameters are summarized in Supplementary Table 28.

Supplementary Table 24. Summary of the illustration and the labels of DFT B3LYP/6-31G(d,p) /IEF-PCM-UFF optimized structures of **1**–**3**: perpendicular geometries (**1**–**3perpend**), the skewed cis and trans geometries (**1**–**3cis** and **1**–**3trans**), and transition state geometries (**1**–**3TS**). [a]

Mol. **1**	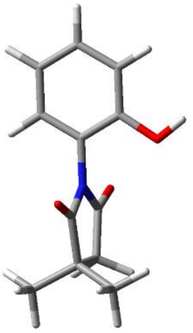 **1-perpend**	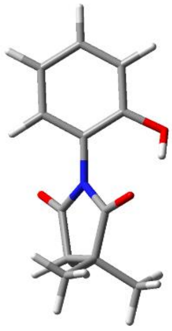 **1-cis**	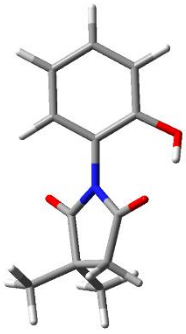 **1-trans**	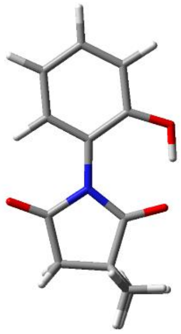 **1-TS-cis**	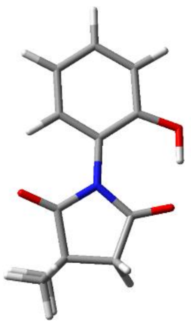 **1-TS-trans**
Mol. **2**	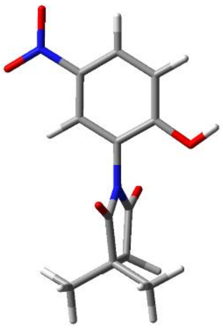 **2-perpend**	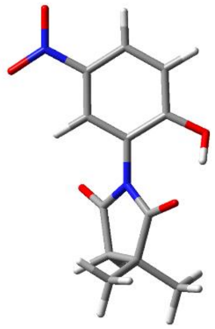 **2-cis**	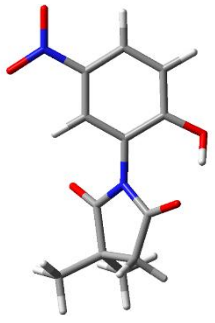 **2-trans**	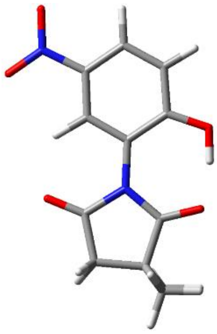 **2-TS-cis**	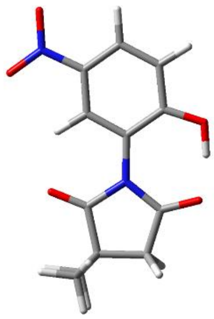 **2-TS-trans**
Mol. **3**	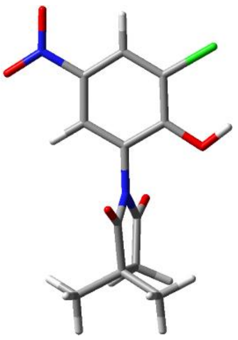 **3-perpend**	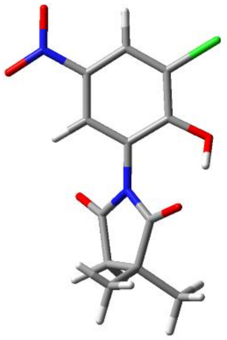 **3-cis**	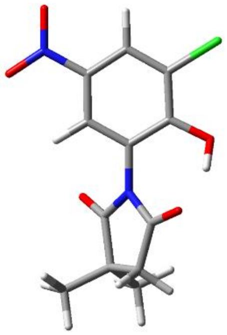 **3-trans**	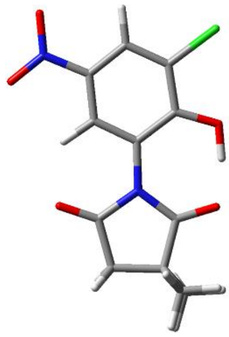 **3-TS-cis**	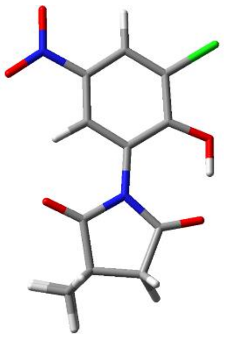 **3-TS-trans**

[a] Illustration based on the optimized geometries of **1**–**3** in acetone. The optimized structures in acetonitrile and chloroform bear a very close resemblance to the corresponding one in acetone. For geometries/coordinate outputs, see Section 10 in this Supplementary Information.

Supplementary Table 25. The DFT B3LYP/6-31G(d,p)/IEF-PCM-UFF+ZPVE calculations for **1−3perpend**, **1−3cis**, **1−3trans**, and **1−3TS-trans**: energies ($E°$, hartree), zero point vibrational energies (ZPVE, hartree), relative energies (kcal mol^{-1}), imaginary and lowest vibrational frequencies (cm^{-1}), dipole moments (μ, D) and the parameters of the intramolecular H-bonding.

geometry	Point grp.[a]	Medium	$E°$	ZPVE	$E°$+ZPVE	Rel. energies[b]	RMS gradient [c] ($\times 10^{-6}$)	Lowest vibrational frequencies	θ [d] (°)	μ	$d_{H\cdots O}$[e] (Å)	$\varphi_{O–H\cdots O}$ [f] (°)
**1**-perpend	C_1	acetonitrile	–745.631557617	0.231954	–745.3996036	**0.00**	2	21.2, 30.9	88.7	1.22	/	/
**1**-cis	C_1	acetonitrile	–745.631412583	0.232362	–745.3990506	0.35	1	31.0, 67.1	50.2	4.63	1.812	153
**1**-trans	C_1	acetonitrile	–745.631465059	0.232369	–745.3990961	0.32	1	25.1, 64.2	49.8	4.63	1.809	153
**1**-TS-cis	C_1	acetonitrile	–745.616594509	0.232327	–745.3842675	9.62	1	***i*88.7**, 25.9	0.56	5.37	1.505	167
**1**-TS-trans	C_1	acetonitrile	–745.616792138	0.232309	–745.3844831	9.49	14	***i*86.0**, 32.4	0.86	5.52	1.506	167
**1**-perpend	C_1	acetone	–745.63084145	0.231945	–745.398896	**0.00**	12	19.7, 29.7	88.7	1.21	/	/
**1**-cis	C_1	acetone	–745.63089569	0.232391	–745.398505	0.25	2	32.0, 67.2	50.2	4.58	1.811	153
**1**-trans	C_1	acetone	–745.63094199	0.232405	–745.398537	0.23	1	27.3, 64.7	49.7	4.58	1.807	153
**1**-TS-cis	C_1	acetone	–745.61615527	0.232352	–745.383803	9.47	2	***i*88.2**, 26.4	0.61	5.32	1.506	167
**1**-TS-trans	C_1	acetone	–745.61635184	0.232335	–745.384017	9.34	9	***i*85.9**, 34.3	0.84	5.48	1.507	167
**1**-perpend	C_1	chloroform	–745.62607543	0.231878	–745.394197	0.46	3	3.6, 25.6	88.8	1.22	/	/
**1**-cis	C_1	chloroform	–745.62744981	0.232524	–745.394926	**0.00**	2	30.2, 67.2	49.5	4.27	1.803	153
**1**-trans	C_1	chloroform	–745.62748013	0.232585	–745.394895	0.02	1	34.5, 65.8	49.1	4.27	1.800	153
**1**-TS-cis	C_1	chloroform	–745.61317840	0.232465	–745.380713	8.92	3	***i*85.9**, 26.7	0.81	5.00	1.514	166
**1**-TS-trans	C_1	chloroform	–745.61336365	0.232493	–745.380871	8.82	3	***i*85.6**, 37.0	0.85	5.18	1.515	167
**2**-perpend	C_1	acetonitrile	–950.145731843	0.234205	–949.9115268	**0.00**	1	4.22, 28.3	88.0	8.56	/	/
**2**-cis	C_1	acetonitrile	–950.145862039	0.234590	–949.911272	0.16	8	30.7, 48.5	47.2	9.33	1.737	156
**2**-trans	C_1	acetonitrile	–950.145976296	0.234664	–949.9113123	0.13	12	42.0, 50.8	47.6	9.06	1.743	156
**2**-TS-cis	C_1	acetonitrile	–950.134599573	0.234367	–949.9002326	7.09	1	***i*71.6**, 29.9	0.36	10.81	1.477	167
**2**-TS-trans	C_1	acetonitrile	–950.134843042	0.234272	-949.900571	6.87	3	***i*68.6**, 24.3	0.20	10.42	1.476	167
**2**-perpend	C_1	acetone	–950.14486349	0.234206	–949.910658	**0.00**	1	5.9, 31.3	90.0	8.50	/	/
**2**-cis	C_1	acetone	–950.14520763	0.234613	–949.910595	0.04	3	27.8, 48.6	47.1	9.26	1.736	156
**2**-trans	C_1	acetone	–950.14532151	0.234708	–949.910614	0.03	3	42.3, 51.0	47.6	8.97	1.742	156
**2**-TS-cis	C_1	acetone	–950.13394496	0.234375	–949.899570	6.96	10	***i*67.0**, 34.6	0.39	10.7	1.478	167
**2**-TS-trans	C_1	acetone	–950.13417142	0.234263	–949.899908	6.75	3	***i*68.4**, 19.5	0.28	10.3	1.478	167
**2**-perpend	C_1	chloroform	–950.13909380	0.234273	–949.904821	0.73	2	6.7, 33.3	89.9	8.01	/	/
**2**-cis	C_1	chloroform	–950.14076350	0.234784	–949.905980	**0.00**	3	18.6, 50.0	46.6	8.78	1.732	156
**2**-trans	C_1	chloroform	–950.14087970	0.234943	–949.905937	0.03	6	42.3, 52.4	47.3	8.44	1.739	156
**2**-TS-cis	C_1	chloroform	–950.12938840	0.234504	–949.894884	6.96	0	***i*71.2**, 21.7	0.78	10.1	1.487	167
**2**-TS-trans	C_1	chloroform	–950.12964243	0.234450	–949.895192	6.77	5	***i*66.5**, 16.8	0.65	9.64	1.487	167
**3**-perpend	C_1	acetonitrile	–1409.734121	0.224594	–1409.509527	**0.00**	1	15.5, 33.7	87.6	7.54	/	/
**3**-cis	C_1	acetonitrile	–1409.73325362	0.224746	–1409.508508	0.64	7	27.3, 47.0	47.2	9.81	1.705	157
**3**-trans	C_1	acetonitrile	–1409.73329612	0.224733	–1409.508563	0.60	9	35.0, 44.9	47.0	9.62	1.703	158
**3**-TS-cis	C_1	acetonitrile	–1409.72177659	0.224495	–1409.497282	7.68	2	***i*69.1**, 31.7	0.35	11.3	1.452	168
**3**-TS-trans	C_1	acetonitrile	–1409.72198824	0.224439	–1409.497549	7.52	4	***i*63.4**, 12.3	0.75	11.0	1.453	169
**3**-perpend	C_1	acetone	–1409.73340641	0.224612	–1409.508794	**0.00**	1	14.3, 34.0	87.7	7.48	/	/
**3**-cis	C_1	acetone	–1409.73258467	0.224798	–1409.507787	0.63	6	31.0, 47.0	47.2	9.74	1.705	157
**3**-trans	C_1	acetone	–1409.73262104	0.224749	–1409.507872	0.58	25	35.3, 45.3	47.1	9.54	1.703	158
**3**-TS-cis	C_1	acetone	–1409.72107682	0.224536	–1409.496541	7.69	3	***i*69.5**, 30.4	0.41	11.2	1.453	168
**3**-TS-trans	C_1	acetone	–1409.72128904	0.224503	–1409.496786	7.54	12	***i*62.4**, 23.1	0.95	10.9	1.455	169
**3**-perpend	C_1	chloroform	–1409.72856031	0.224793	–1409.503767	**0.00**	1	14.5, 26.4	83.7	7.08	/	/
**3**-cis	C_1	chloroform	–1409.72805474	0.225016	–1409.503039	0.46	6	38.0, 46.5	47.2	9.27	1.709	157
**3**-trans	C_1	chloroform	–1409.72809840	0.224928	–1409.503170	0.37	20	40.2, 46.9	47.1	9.04	1.707	157
**3**-TS-cis	C_1	chloroform	–1409.71636332	0.224638	–1409.491725	7.56	1	***i*67.5**, 23.4	0.84	10.6	1.464	168
**3**-TS-trans	C_1	chloroform	–1409.71660433	0.224708	–1409.491896	7.45	10	***i*66.4**, 20.9	0.71	10.3	1.465	168

[a] Symmetry point group of the geometry. [b] Relative energy with relative to the ground state geometry. 1 Hartree = 627.5095 kcal mol^{-1}. [c] In Cartesian coordinates. [d] Dihedral angle between the planes of the succinimide ring and the phenol ring. [e] The H(hydroxyl)···O(carbonyl) distance. [f] The bond angles of the O(phenol)−H···O(carbonyl) intramolecular hydrogen bond.

Supplementary Table 26. Selected angles, torsion angles and bond lengths in X-ray structures of **1−3** as well as their global minimum structures calculated at the DFT B3LYP/6-31G(d,p) level using the polarizable continuum model for acetone. [a]

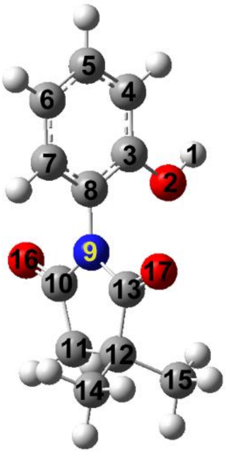

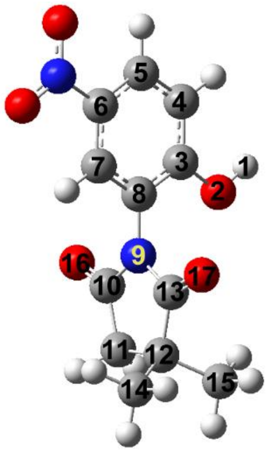

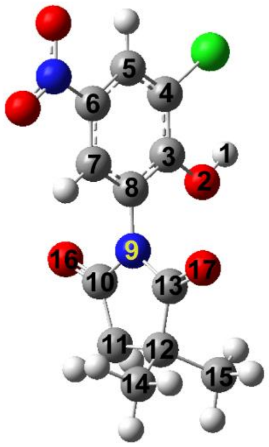

Compd.	Medium	$\theta_{ring\text{-}ring}$ [b] (°)	θ (N9,C10,C11)/(N9,C13,C12) [c] (°)	θ (O16,C10,N9)/(O17,C13,N9) [c] (°)	$\psi_{C7/C8/C9/C13}$ [d] (°)	$\psi_{C3/C8/C9/C10}$ [d] (°)	$d_{C8–N9}$ (Å)	$\varphi_{C3–O2–H1}$ [e] (°)	$d_{C3–O2}$ (Å)	$d_{O2–H1}$ (Å)
**1** (expt)	solid	78-86	8.9-11.0	5.3-7.4	101-108	100-109	1.431(7)	110	1.28-1.34	0.820(1)
**1**-perpend (DFT)	acetone	88	1.44	1.16	–90.0	–87.4	1.432	110	1.365	0.968
**2** (expt)	solid	88	3.99	3.20	–90.0(2)	–92.7(2)	1.430(2)	111.2(18)	1.342(2)	0.91(3)
**2**-perpend (DFT)	acetone	89	1.57	1.33	–91.6	–88.4	1.427	111	1.349	0.969
**3** (expt)	solid	84	6.80	4.07	–82.76(17)	–70.32(17)	1.427(2)	109.5	1.332(2)	0.820
**3**-perpend (DFT)	acetone	87	1.56	1.26	–88.8	–86.7	1.426	111	1.341	0.973

[a] Elaborations of labels of the molecular atoms are inserted in the table. For clarity and uniformity of the labelling, several atoms are ignored. [b] Dihedral angle between the planes of the succinimide ring (atoms N9, C11, and C12 located) and the phenol ring (atoms C4, C6, and C8 located). [c] Dihedral angle between the planes of the atoms those listed in parentheses located. [d] Torsion angles. [e] Bond angles.

Supplementary Table 27. Summary of the illustration and the labels of DFT B3LYP/6-31G(d,p) optimized structures of **(3–H)**$^{-}$ and **(3–H)**$^{-}$**·M**$^{+}$ (M^{+} = Li^{+}, Na^{+}, and K^{+}): perpendicular geometries (**perpend**), the skewed *cis* and *trans* geometries (**cis** and **trans**), and transition state in *cis* and *trans* geometries (**TS-cis** and **TS-trans**). [a]

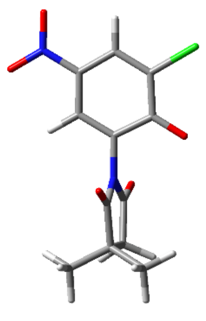
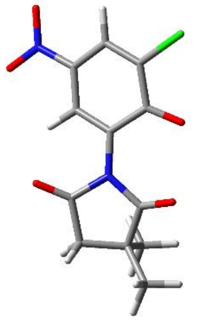
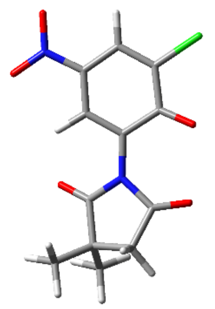

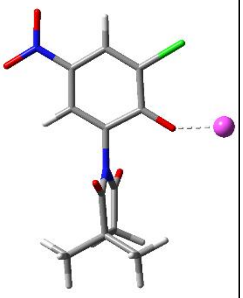
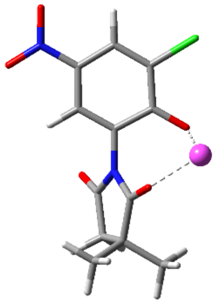
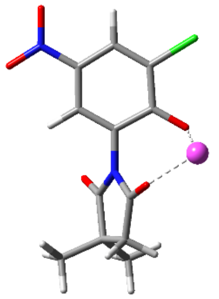
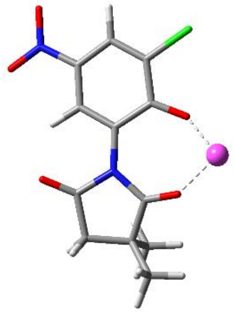
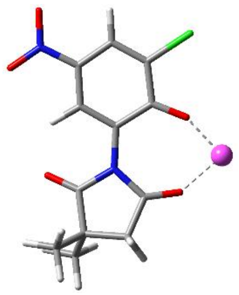

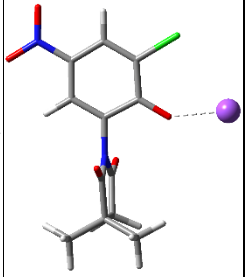
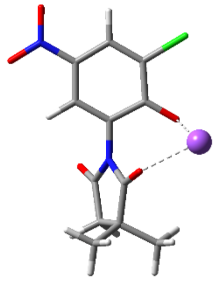
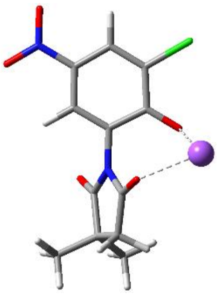
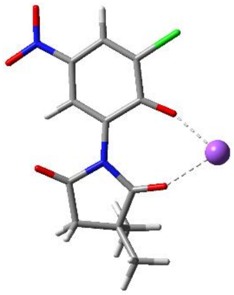
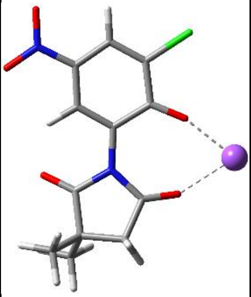

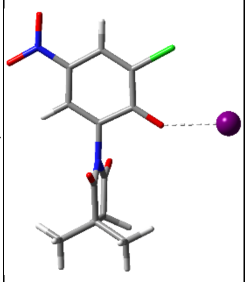
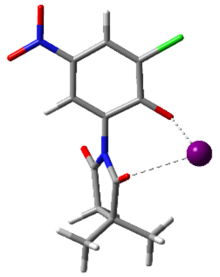
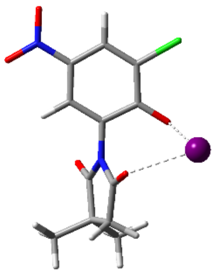
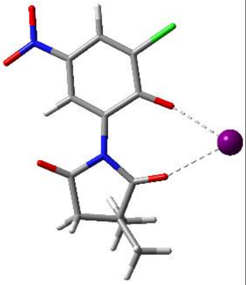
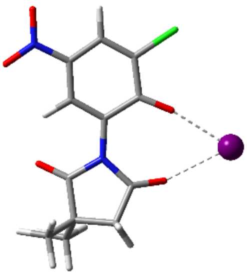

**(3–H)**$^{-}$	**(3–H)$^{-}$-perpend**	/	/	**(3–H)$^{-}$-TS-cis**	**(3–H)$^{-}$-TS-trans**
**(3–H)$^{-}$ ·Li^{+}**	**(3–H)$^{-}$·Li^{+}-perpend**	**(3–H)$^{-}$·Li^{+}-cis**	**(3–H)$^{-}$·Li^{+}-trans**	**(3–H)$^{-}$·Li^{+}-TS-cis**	**(3–H)$^{-}$·Li^{+}-TS-trans**
**(3–H)$^{-}$ ·Na^{+}**	**(3–H)$^{-}$·Na^{+}-perpend**	**(3–H)$^{-}$·Na^{+}-cis**	**(3–H)$^{-}$·Na^{+}-trans**	**(3–H)$^{-}$·Na^{+}-TS-cis**	**(3–H)$^{-}$·Na^{+}-TS-trans**
**(3–H)$^{-}$ ·K^{+}**	**(3–H)$^{-}$·K^{+}-perpend**	**(3–H)$^{-}$·K^{+}-cis**	**(3–H)$^{-}$·K^{+}-trans**	**(3–H)$^{-}$·K^{+}- TS-cis**	**(3–H)$^{-}$·K^{+}- TS-trans**

[a] Illustration based on the DFT-optimized geometries, using the B3LYP/6-31G(d,p) method and IEF-PCM-UFF solvent model for acetonitrile. For geometries/coordinate outputs, see Section 10 in this Supplementary Information.

Supplementary Table 28. The DFT B3LYP/6-31G(d,p)/IEF-PCM-UFF+ZPVE calculations for the minima, intermediates, and transition states of **(3–H)**$^-$ and **(3–H)**$^-$**·M**$^+$ (M^+ = Li^+, Na^+, and K^+): energies ($E°$, hartree), zero point vibrational energies (ZPVE, hartree), relative energies (kcal mol^{-1}), RMS gradient norms (a.u.), imaginary and lowest vibrational frequencies (cm^{-1}), dipole moments (μ, D) and some important structural parameters.

geometry	Char-ge, Spin	Point grp.^{a}	$E°$	ZPVE	$E°$+ZPVE	Rel. energies^b	RMS gradient^c (×10^{-6})	Lowest vibrational frequencies	θ^d (°)	μ	$d_{O(ph)\cdots M^+}$^{e} (Å)	$d_{O(car)\cdots M^+}$^{f} (Å)	$\varphi_{O\cdots M^+\cdots O}$^{g} (°)
**(3–H)**$^-$-perpend	–1, 1	C_1	–1409.28677855	0.211593	–1409.075186	0.00	2	17.3, 36.0	88	11.5	/	/	/
**(3–H)**$^-$-TS-cis	–1, 1	C_1	–1409.25151335	0.210869	–1409.040644	**21.67**	0	***i85.9***, 24.2	17	15.5	/	/	/
**(3–H)**$^-$-TS-trans	–1, 1	C_1	–1409.25121946	0.210951	–1409.040268	**21.91**	6	***i106.6***, 25.9	15	15.1	/	/	/
**(3–H)**$^-$·**K**$^+$-perpend	0, 1	C_1	–2009.14593184	0.212258	–2008.933674	0.29	1	17.2, 29.6	88	23.9	2.629	/	/
**(3–H)**$^-$·**K**$^+$-cis	0, 1	C_1	–2009.14652382	0.212604	–2008.933920	0.13	4	28.3, 33.9	69	23.1	2.687	2.812	70
**(3–H)**$^-$·**K**$^+$-trans	0, 1	C_1	–2009.14656292	0.212429	–2008.934134	0.00	2	24.5, 29.2	68	22.7	2.683	2.801	70
**(3–H)**$^-$·**K**$^+$-TS-cis	0, 1	C_1	–2009.11541722	0.211807	–2008.903610	**19.15**	1	***i83.3***, 22.3	12	26.1	2.593	2.694	59
**(3–H)**$^-$·**K**$^+$-TS-trans	0, 1	C_1	–2009.11514361	0.211868	–2008.903276	**19.36**	2	***i86.5***, 20.1	12	25.6	2.596	2.692	59
**(3–H)**$^-$·**Na**$^+$-perpend	0, 1	C_1	–1571.53891555	0.212614	–1571.326302	0.02	1	18.4, 34.4	88	21.7	2.226	/	/
**(3–H)**$^-$·**Na**$^+$-cis	0, 1	C_1	–1571.53891141	0.212968	–1571.325943	0.25	2	23.2, 36.0	65	20.6	2.284	2.380	82
**(3–H)**$^-$·**Na**$^+$-trans	0, 1	C_1	–1571.53905210	0.212718	–1571.326334	0.00	17	***i17***, 28.1	63	20.7	2.285	2.375	81
**(3–H)**$^-$·**Na**$^+$-TS-cis	0, 1	C_1	–1571.50964490	0.212321	–1571.297324	**18.20**	1	***i77.9***, 30.4	10	23.7	2.204	2.291	71
**(3–H)**$^-$·**Na**$^+$-TS-trans	0, 1	C_1	–1571.50949536	0.212441	–1571.297054	**18.37**	1	***i82.0***, 22.1	10	23.1	2.200	2.277	71
**(3–H)**$^-$·**Li**$^+$-perpend	0, 1	C_1	–1416.77905285	0.213758	–1416.565295	0.00	1	15.0, 35.4	89	19.5	1.851	/	/
**(3–H)**$^-$·**Li**$^+$-cis	0, 1	C_1	–1416.77842297	0.214221	–1416.564202	0.69	6	36.5, 43.7	59	18.3	1.900	2.048	95
**(3–H)**$^-$·**Li**$^+$-trans	0, 1	C_1	–1416.77846118	0.214021	–1416.564440	0.54	1	24.3, 39.5	58	17.9	1.919	2.015	95
**(3–H)**$^-$·**Li**$^+$-TS-cis	0, 1	C_1	–1416.75250993	0.213648	–1416.538862	**15.90**	1	***i82.7***, 32.5	7.9	20.4	1.839	1.915	88
**(3–H)**$^-$·**Li**$^+$-TS-trans	0, 1	C_1	–1416.75246168	0.213782	–1416.538680	**16.02**	1	***i78.8***, 30.5	6.3	20.0	1.832	1.914	88

^{a} Symmetry point group of the geometry. ^{b} Relative energy with relative to the ground state geometry. 1 Hartree = 627.5095 kcal mol^{-1}. ^{c} In Cartesian coordinates. ^{d} Dihedral angle between the planes of the succinimide ring and the phenol ring. ^{e} The O(phenol)···M^+ distance. ^{f} The O(carbonyl)···M^+ distance. ^{g} The bond angles of the O(phenol)···M^+···O(carbonyl) bonding.

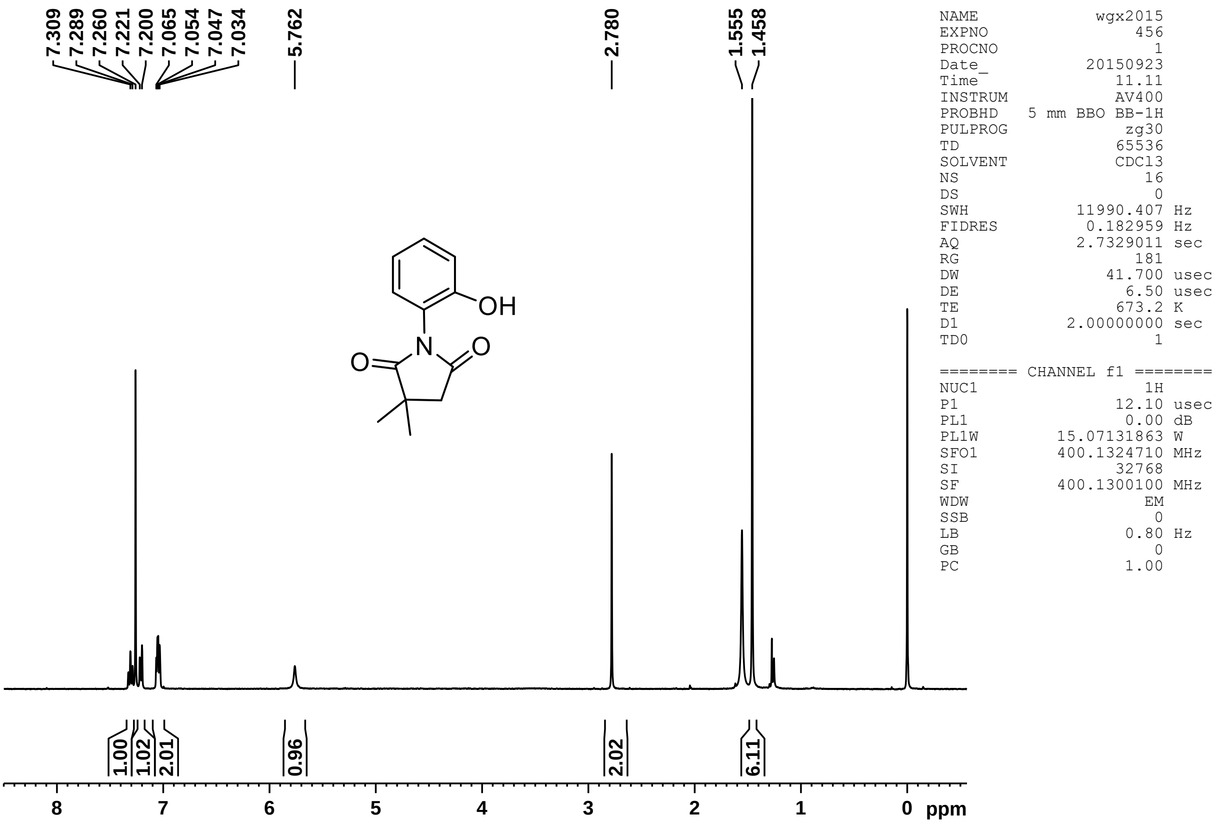

Supplementary Figure 149. ^{1}H NMR spectrum (400 MHz, $CDCl_3$) of compound **1**.

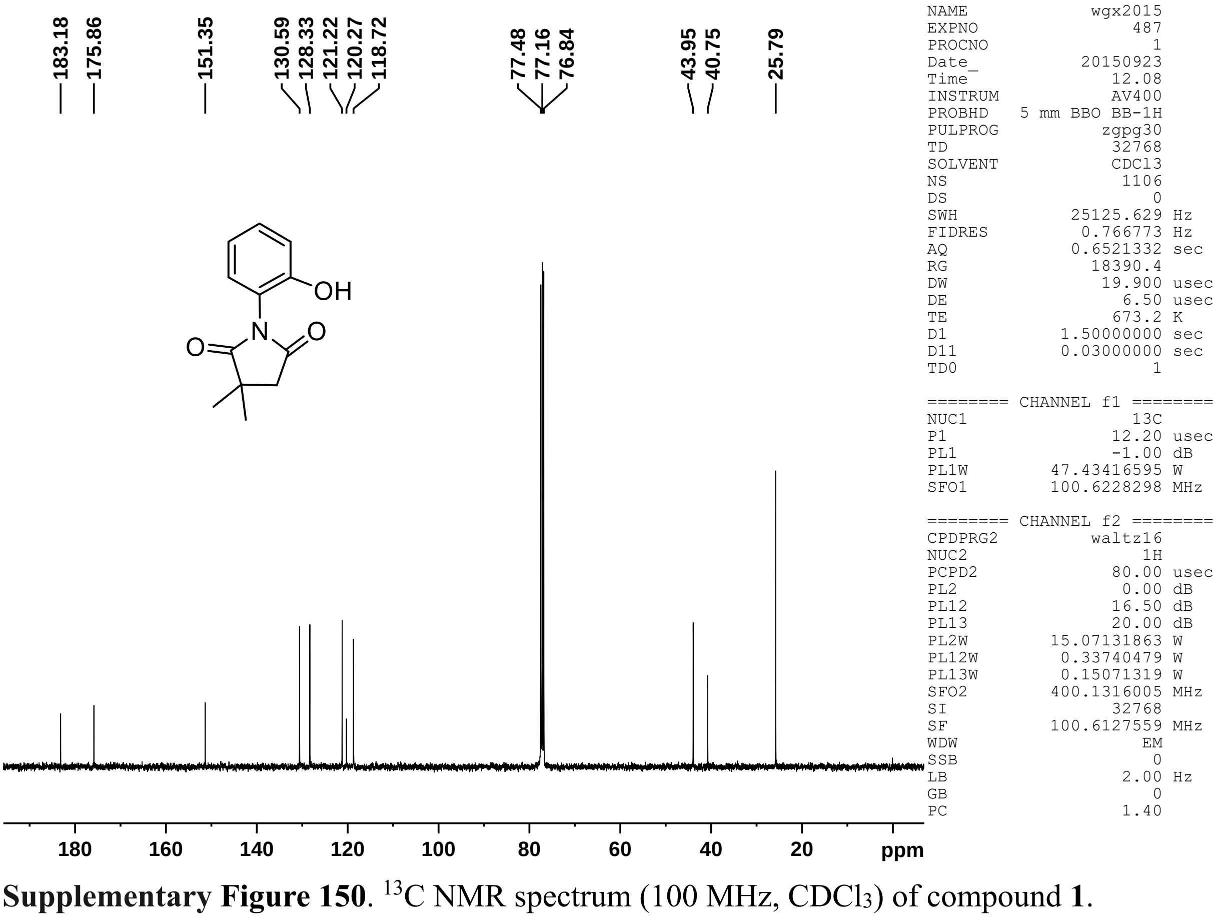

Supplementary Figure 150. ^{13}C NMR spectrum (100 MHz, $CDCl_3$) of compound **1**.

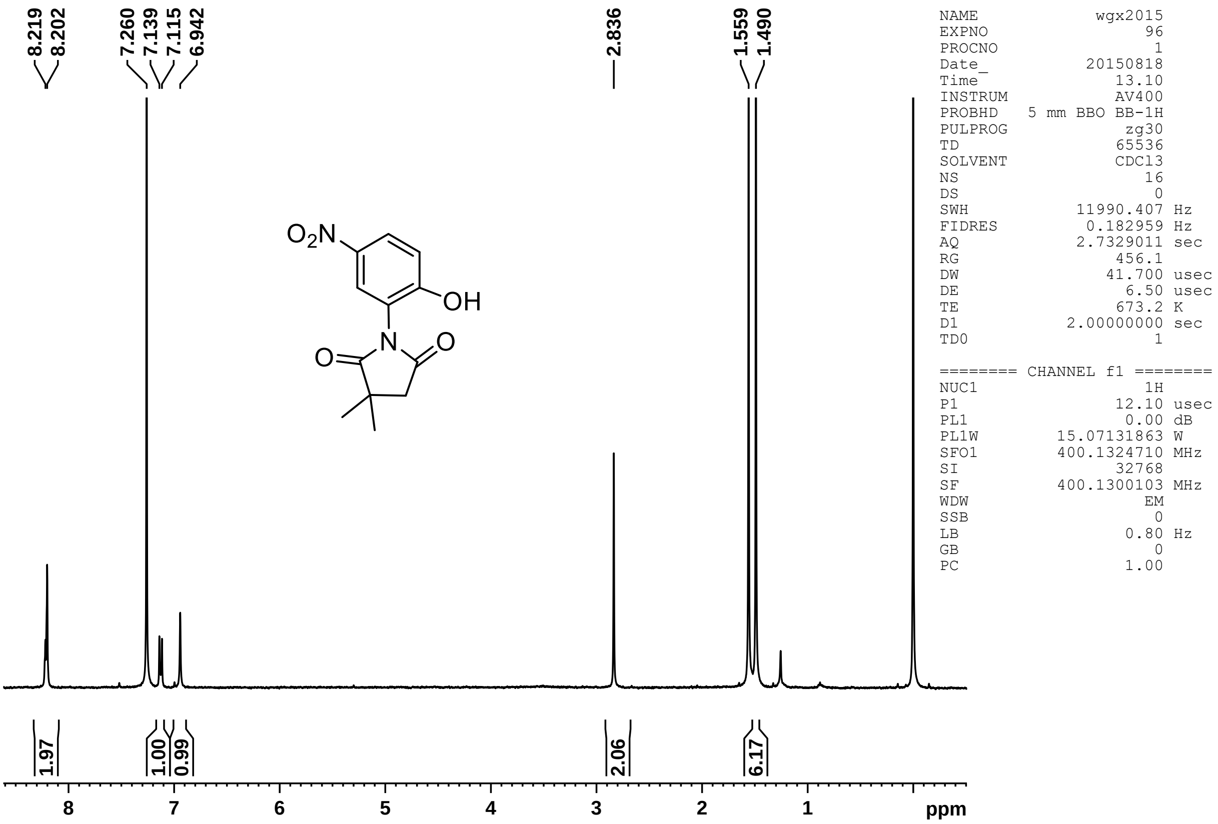

Supplementary Figure 151. ^{1}H NMR spectrum (400 MHz, CDCl$_3$) of compound **2**.

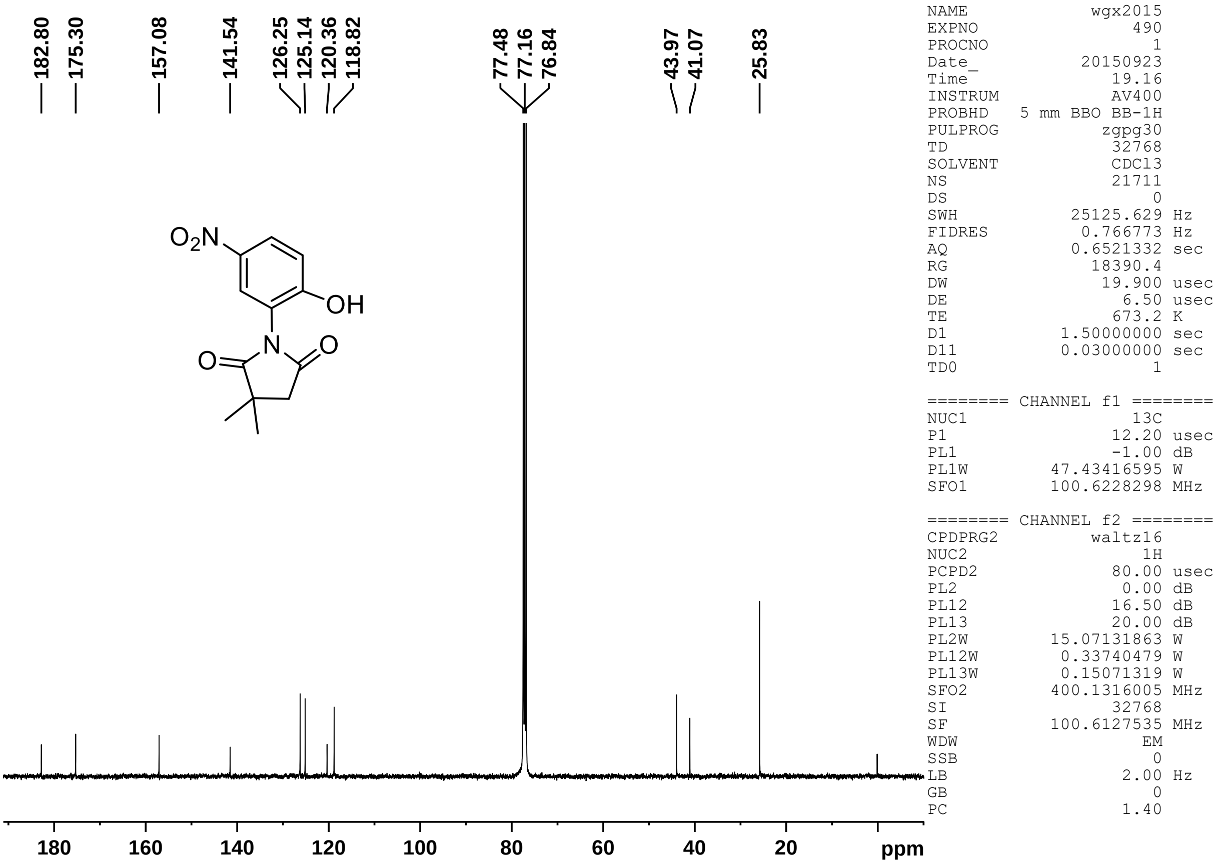

Supplementary Figure 152. ^{13}C NMR spectrum (100 MHz, CDCl$_3$) of compound **2**.

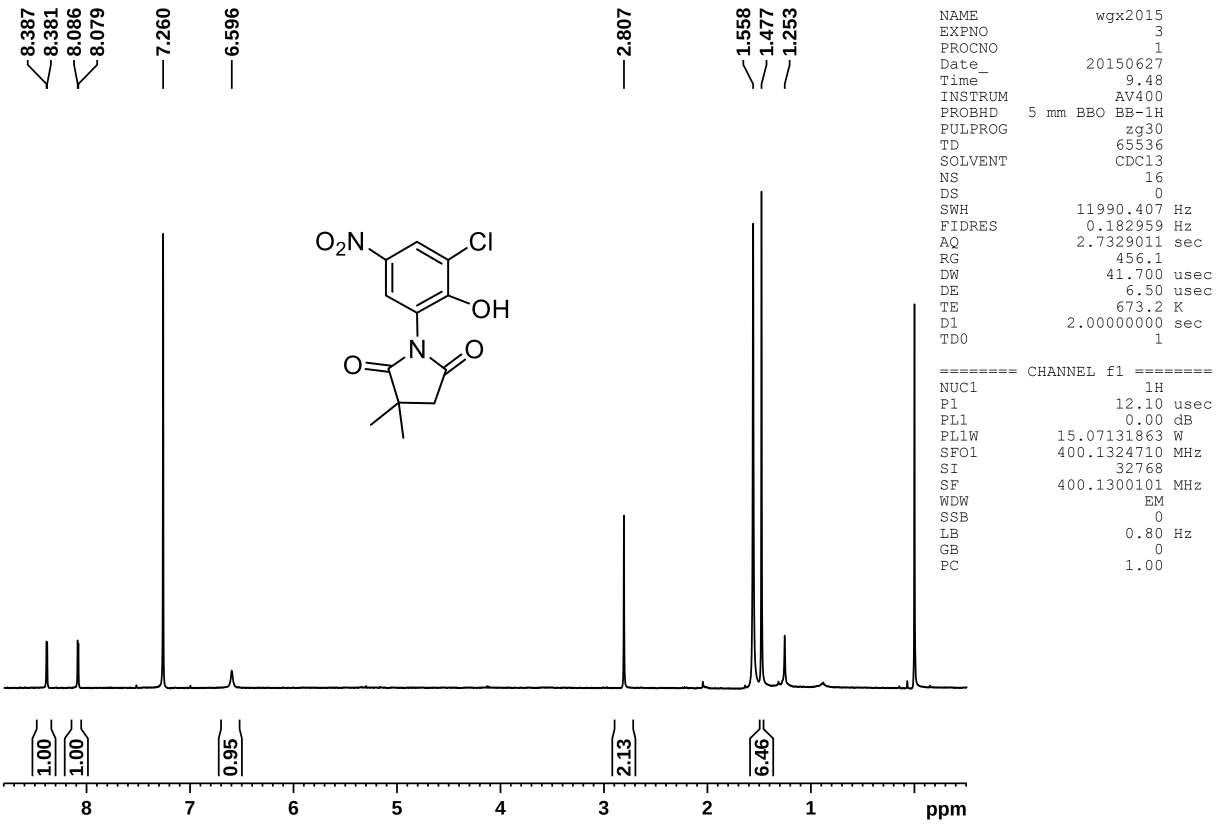

Supplementary Figure 153. ^{1}H NMR spectrum (400 MHz, $CDCl_3$) of compound **3**.

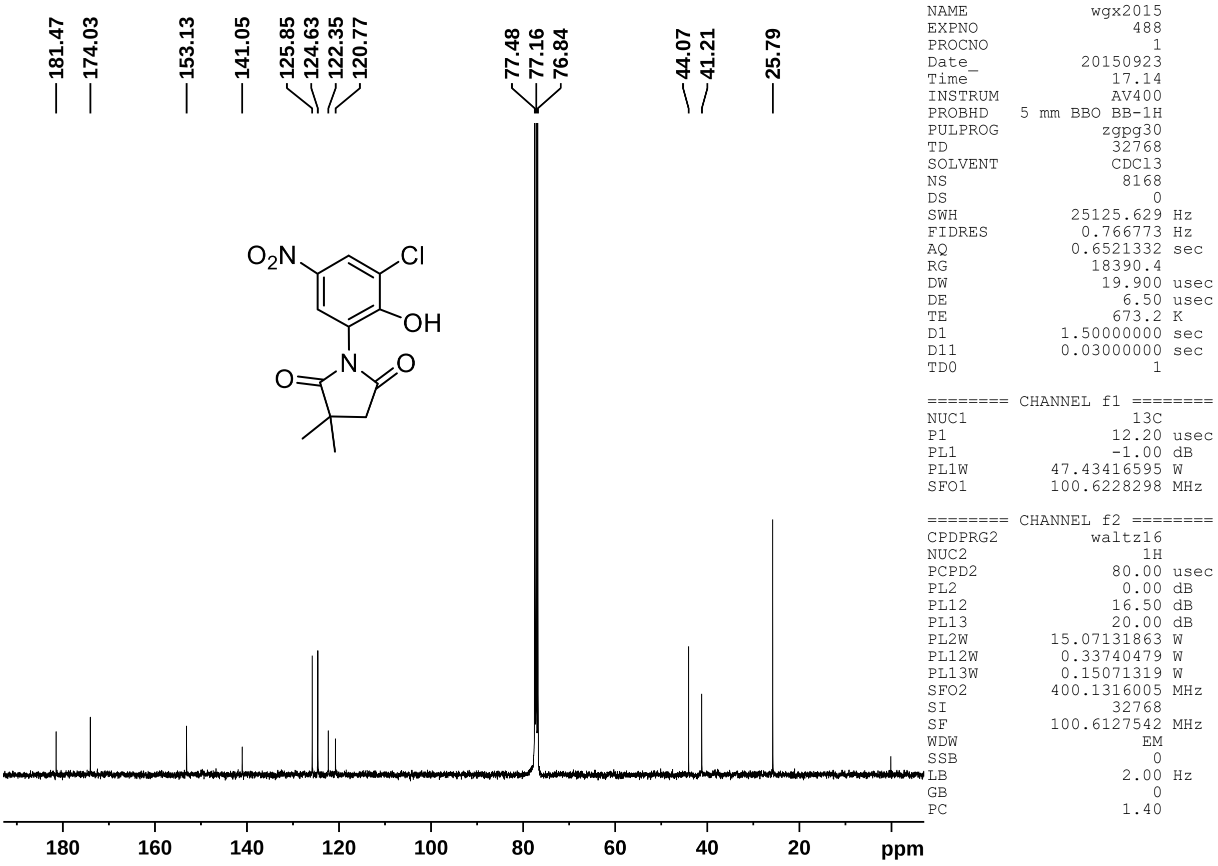

Supplementary Figure 154. ^{13}C NMR spectrum (100 MHz, $CDCl_3$) of compound **3**.

Supplementary Table 29. Optimized Geometries/Coordinate Outputs of DFT Calculations

1-perpend (in acetonitrile):

Center Number	Atomic Number	Atomic Type	Coordinates (Angstroms) X	Y	Z
1	8	0	1.195378	1.785110	-1.100719
2	1	0	1.789388	2.385514	-1.573850
3	8	0	-0.199169	1.227514	2.234089
4	8	0	-0.872755	-1.252880	-1.563709
5	7	0	-0.230049	-0.068590	0.319315
6	6	0	3.285567	0.606200	-0.715922
7	6	0	3.942022	-0.483309	-0.140439
8	1	0	5.016228	-0.580339	-0.264633
9	6	0	3.230392	-1.441536	0.588576
10	6	0	1.849455	-1.300983	0.738437
11	1	0	1.273363	-2.030117	1.299539
12	6	0	1.186004	-0.214546	0.166505
13	6	0	1.901099	0.747036	-0.565752
14	6	0	-0.824697	0.663300	1.352771
15	6	0	-2.349046	0.623398	1.187646
16	6	0	-2.547561	-0.206775	-0.103077
17	1	0	-3.131738	-1.117932	0.059441
18	1	0	-3.043343	0.356444	-0.899919
19	6	0	-1.163672	-0.595747	-0.578274
20	6	0	-2.878599	2.064385	1.047250
21	1	0	-2.644016	2.649288	1.940912
22	1	0	-3.965776	2.044788	0.921778
23	1	0	-2.442753	2.568995	0.178926
24	6	0	-2.968699	-0.059166	2.424055
25	1	0	-2.598031	-1.082219	2.545730
26	1	0	-4.056759	-0.101089	2.312290
27	1	0	-2.735215	0.503965	3.331933
28	1	0	3.742714	-2.287124	1.034947
29	1	0	3.841689	1.348514	-1.281977

1-cis (in acetonitrile):

Center Number	Atomic Number	Atomic Type	Coordinates (Angstroms) X	Y	Z
1	8	0	-0.874478	-1.770100	0.699604
2	8	0	-0.216605	2.413527	-1.055828
3	7	0	-0.220224	0.303659	-0.073077
4	6	0	1.886058	-0.975003	-0.384011
5	6	0	3.281408	-1.025641	-0.271728
6	1	0	3.791544	-1.909326	-0.641341
7	6	0	3.986776	0.026814	0.307803
8	1	0	5.068232	-0.030620	0.384846
9	6	0	3.302716	1.141626	0.805918
10	1	0	3.842904	1.955995	1.277252
11	6	0	1.915164	1.203081	0.696458
12	1	0	1.374690	2.063852	1.072834
13	6	0	1.201122	0.163206	0.085758
14	6	0	-1.165354	-0.645933	0.297041
15	6	0	-2.575943	-0.089093	0.118157
16	6	0	-2.327910	1.270349	-0.572404
17	1	0	-2.707605	1.295651	-1.598779
18	1	0	-2.774884	2.116005	-0.041508
19	6	0	-0.828480	1.458231	-0.619984
20	6	0	-3.414619	-1.051846	-0.743065
21	1	0	-2.969666	-1.195050	-1.732895
22	1	0	-4.419718	-0.640029	-0.875919
23	1	0	-3.501611	-2.027511	-0.257750
24	6	0	-3.212167	0.076932	1.516761
25	1	0	-3.285506	-0.889160	2.023627
26	1	0	-4.219933	0.489366	1.408689
27	1	0	-2.629556	0.756890	2.146542
28	8	0	1.252437	-2.030010	-0.979314
29	1	0	0.421643	-2.205224	-0.489744

1-trans (in acetonitrile):

Center Number	Atomic Number	Atomic Type	Coordinates (Angstroms) X	Y	Z
1	8	0	-0.948945	-1.860856	0.835908
2	8	0	-0.174593	2.253729	-1.031539
3	7	0	-0.235619	0.114535	-0.160185
4	6	0	2.095079	0.760598	0.404325
5	6	0	3.449329	0.409269	0.473753
6	1	0	4.142804	1.130802	0.893586
7	6	0	3.888181	-0.827786	0.006788
8	1	0	4.941814	-1.081992	0.070375
9	6	0	2.978650	-1.728099	-0.559785
10	1	0	3.315387	-2.684978	-0.944719
11	6	0	1.629173	-1.389469	-0.631336
12	1	0	0.914665	-2.080730	-1.062739
13	6	0	1.174621	-0.160208	-0.134718
14	6	0	-1.210974	-0.794699	0.315657
15	6	0	-2.607134	-0.212098	0.076364
16	6	0	-2.317530	1.142168	-0.608133
17	1	0	-2.694086	1.188355	-1.635345
18	1	0	-2.737051	1.998724	-0.072489
19	6	0	-0.816763	1.277015	-0.650288
20	6	0	-3.405664	-1.172365	-0.828136
21	1	0	-2.911502	-1.318128	-1.794088
22	1	0	-4.400897	-0.755981	-1.012535
23	1	0	-3.521315	-2.147199	-0.346768
24	6	0	-3.315459	-0.039119	1.436035
25	1	0	-3.419299	-1.003941	1.939753
26	1	0	-4.314347	0.378143	1.276121
27	1	0	-2.762679	0.638311	2.094865
28	8	0	1.723910	1.980479	0.897033
29	1	0	1.043811	2.358809	0.300905

1-TS-cis (in acetonitrile):

Center Number	Atomic Number	Atomic Type	Coordinates (Angstroms) X	Y	Z
1	8	0	-1.034730	-1.883615	0.043841
2	8	0	-0.351296	2.693252	-0.098688
3	7	0	-0.227711	0.322367	-0.062739
4	6	0	1.935246	-1.097086	-0.000367
5	6	0	3.343735	-1.093417	0.005534
6	1	0	3.822246	-2.066961	0.041326
7	6	0	4.093614	0.070967	-0.033920
8	1	0	5.178282	0.022598	-0.028094
9	6	0	3.423273	1.293047	-0.081565
10	1	0	3.967267	2.231418	-0.114841
11	6	0	2.032237	1.318389	-0.086031
12	1	0	1.544402	2.275664	-0.120733
13	6	0	1.238326	0.150125	-0.044548
14	6	0	-1.217317	-0.668589	-0.016842
15	6	0	-2.624239	-0.085358	-0.023944
16	6	0	-2.374029	1.406704	-0.230102
17	1	0	-2.679164	1.754186	-1.223365
18	1	0	-2.867994	2.048736	0.503822
19	6	0	-0.884750	1.602186	-0.121654
20	6	0	-3.444198	-0.725603	-1.160453
21	1	0	-2.981252	-0.548302	-2.136434
22	1	0	-4.446569	-0.286844	-1.172348
23	1	0	-3.538394	-1.803940	-1.010543
24	6	0	-3.278034	-0.381199	1.345461
25	1	0	-3.346182	-1.458769	1.515169
26	1	0	-4.288111	0.039124	1.357666
27	1	0	-2.711110	0.066864	2.167801
28	8	0	1.407959	-2.342145	0.033664
29	1	0	0.412757	-2.294180	0.042869

1-TS-trans (in acetonitrile):

Center Number	Atomic Number	Atomic Type	Coordinates (Angstroms) X	Y	Z
1	8	0	-0.443384	2.597502	0.164233
2	8	0	-0.996721	-1.993807	0.113830
3	7	0	-0.249645	0.238227	0.031671
4	6	0	1.949581	-1.116036	-0.061398
5	6	0	3.356117	-1.073001	-0.118656
6	1	0	3.861568	-2.033029	-0.148987
7	6	0	4.071815	0.113285	-0.141701
8	1	0	5.156479	0.095927	-0.187204
9	6	0	3.367851	1.316797	-0.105971
10	1	0	3.884861	2.270710	-0.123533
11	6	0	1.977887	1.302978	-0.044439
12	1	0	1.464148	2.246796	-0.012200
13	6	0	1.219120	0.111520	-0.016529
14	6	0	-0.949544	1.500720	0.049806
15	6	0	-2.451107	1.281746	-0.120702
16	6	0	-2.598549	-0.218629	0.122520
17	1	0	-3.014919	-0.444747	1.110683
18	1	0	-3.213159	-0.740223	-0.615984

1-perpend (in acetone):

Center Number	Atomic Number	Atomic Type	Coordinates (Angstroms) X	Y	Z
1	8	0	1.231742	1.817886	-1.102684
2	1	0	1.836644	2.409568	-1.573016
3	8	0	-0.868635	-1.203561	-1.593744
4	8	0	-0.202885	1.267668	2.212247
5	7	0	-0.229572	-0.027601	0.293026
6	6	0	3.302600	0.607929	-0.710106
7	6	0	3.940300	-0.493746	-0.136718
8	1	0	5.014034	-0.604179	-0.253320
9	6	0	3.210415	-1.447070	0.580376
10	6	0	1.830456	-1.288943	0.720698
11	1	0	1.240129	-2.013927	1.272190
12	6	0	1.185773	-0.190475	0.150456
13	6	0	1.919175	0.766724	-0.569177
14	6	0	-1.163849	-0.546522	-0.610474
15	6	0	-2.576364	-0.160739	-0.153104
16	6	0	-2.322562	0.685661	1.117401
17	1	0	-2.677500	1.716592	1.020541
18	1	0	-2.791392	0.265740	2.012636

19	6	0	-1.206094	-0.780869	0.081873
20	6	0	-3.235376	2.145128	0.880034
21	1	0	-2.966899	1.902588	1.913012
22	1	0	-4.307484	1.966130	0.753286
23	1	0	-3.039121	3.206846	0.711414
24	6	0	-2.821337	1.666946	-1.573311
25	1	0	-2.600660	2.721694	-1.758371
26	1	0	-3.892563	1.503343	-1.725512
27	1	0	-2.275759	1.063474	-2.306025
28	8	0	1.454969	-2.374802	-0.066713
29	1	0	0.461194	-2.356368	0.009707

19	6	0	-0.822506	0.712202	1.320956
20	6	0	-3.371647	-1.447033	0.150006
21	1	0	-3.454952	-2.068578	-0.745868
22	1	0	-4.380124	-1.183858	0.484320
23	1	0	-2.893830	-2.037927	0.938234
24	6	0	-3.266926	0.646766	-1.269846
25	1	0	-2.711953	1.560237	-1.506564
26	1	0	-4.273180	0.931787	-0.946745
27	1	0	-3.352643	0.048826	-2.181402
28	1	0	3.708067	-2.302180	1.025146
29	1	0	3.872740	1.346634	-1.266684

1-cis (in acetone):

Center Number	Atomic Number	Atomic Type	Coordinates (Angstroms) X	Y	Z
1	8	0	-0.871836	-1.753855	0.755957
2	8	0	-0.223153	2.388825	-1.098485
3	7	0	-0.220700	0.300821	-0.069515
4	6	0	1.882063	-0.987428	-0.364664
5	6	0	3.278178	-1.036674	-0.261850
6	1	0	3.784449	-1.929870	-0.613441
7	6	0	3.988906	0.029037	0.285901
8	1	0	5.070889	-0.027584	0.355996
9	6	0	3.309720	1.156643	0.761246
10	1	0	3.854252	1.982078	1.207684
11	6	0	1.921471	1.216905	0.660888
12	1	0	1.384936	2.087785	1.019124
13	6	0	1.201793	0.162835	0.082020
14	6	0	-1.163930	-0.639082	0.329296
15	6	0	-2.575328	-0.084494	0.148688
16	6	0	-2.331362	1.255480	-0.580127
17	1	0	-2.714356	1.251384	-1.605654
18	1	0	-2.777635	2.115353	-0.072030
19	6	0	-0.832073	1.443136	-0.638808
20	6	0	-3.424409	-1.068294	-0.677571
21	1	0	-2.989580	-1.239508	-1.667476
22	1	0	-4.430020	-0.658155	-0.811851
23	1	0	-3.508385	-2.030247	-0.165146
24	6	0	-3.197082	0.120799	1.548673
25	1	0	-3.266549	-0.830922	2.082525
26	1	0	-4.205294	0.531999	1.439763
27	1	0	-2.607092	0.816647	2.153834
28	8	0	1.243071	-2.056027	-0.928905
29	1	0	0.416961	-2.219425	-0.427404

1-trans (in acetone):

Center Number	Atomic Number	Atomic Type	Coordinates (Angstroms) X	Y	Z
1	8	0	-0.949167	-1.865073	0.827664
2	8	0	-0.175832	2.253922	-1.031698
3	7	0	-0.235503	0.113424	-0.162640
4	6	0	2.094292	0.761150	0.403870
5	6	0	3.448641	0.410619	0.474794
6	1	0	4.141051	1.132889	0.895083
7	6	0	3.888712	-0.826351	0.008889
8	1	0	4.942431	-1.079925	0.073730
9	6	0	2.980314	-1.727520	-0.558018
10	1	0	3.317940	-2.684558	-0.941786
11	6	0	1.630723	-1.389799	-0.631086
12	1	0	0.917094	-2.082142	-1.062165
13	6	0	1.174978	-0.160476	-0.135801
14	6	0	-1.210855	-0.797102	0.311503
15	6	0	-2.607032	-0.212086	0.077266
16	6	0	-2.317838	1.139454	-0.612600
17	1	0	-2.691401	1.179605	-1.641230
18	1	0	-2.740381	1.998104	-0.082791
19	6	0	-0.816962	1.276114	-0.651929
20	6	0	-3.413835	-1.173141	-0.818613
21	1	0	-2.926307	-1.323539	-1.787260
22	1	0	-4.409250	-0.755055	-0.998216
23	1	0	-3.528722	-2.146106	-0.333342
24	6	0	-3.306288	-0.032236	1.440920
25	1	0	-3.407775	-0.994654	1.949653
26	1	0	-4.305728	0.385616	1.285846
27	1	0	-2.748341	0.647438	2.093090
28	8	0	1.721877	1.980798	0.895915
29	1	0	1.043781	2.359422	0.297658

1-TS-cis (in acetone):

Center Number	Atomic Number	Atomic Type	Coordinates (Angstroms) X	Y	Z
1	8	0	-1.035300	-1.884372	0.035963
2	8	0	-0.352631	2.693073	-0.084677
3	7	0	-0.227922	0.321946	-0.063037
4	6	0	1.935108	-1.097478	-0.004541
5	6	0	3.343606	-1.093464	0.002373
6	1	0	3.822084	-2.067136	0.034204
7	6	0	4.093167	0.071214	-0.031635
8	1	0	5.177850	0.023035	-0.025321
9	6	0	3.422703	1.293416	-0.074504
10	1	0	3.966570	2.232005	-0.103388
11	6	0	2.031730	1.318573	-0.079645
12	1	0	1.543713	2.275915	-0.110243
13	6	0	1.238185	0.149883	-0.043649
14	6	0	-1.217516	-0.669277	-0.021270
15	6	0	-2.624348	-0.085437	-0.026365
16	6	0	-2.373541	1.405687	-0.238515
17	1	0	-2.669168	1.746317	-1.237147
18	1	0	-2.874866	2.052273	0.486253
19	6	0	-0.885102	1.601965	-0.118146
20	6	0	-3.449436	-0.729771	-1.156476
21	1	0	-2.989576	-0.558472	-2.135000
22	1	0	-4.451131	-0.289374	-1.167263
23	1	0	-3.544926	-1.807174	-1.000947
24	6	0	-3.272430	-0.375104	1.347215
25	1	0	-3.338957	-1.451901	1.522338
26	1	0	-4.282798	0.044495	1.361840
27	1	0	-2.702406	0.077144	2.165135
28	8	0	1.408286	-2.342715	0.023280
29	1	0	0.413316	-2.295647	0.033108

1-TS-trans (in acetone):

Center Number	Atomic Number	Atomic Type	Coordinates (Angstroms) X	Y	Z
1	8	0	-0.443059	2.597177	0.172783
2	8	0	-0.998348	-1.993602	0.116288
3	7	0	-0.249947	0.238106	0.034288
4	6	0	1.948586	-1.116922	-0.062673
5	6	0	3.355059	-1.074149	-0.122035
6	1	0	3.859979	-2.034382	-0.153899
7	6	0	4.070946	0.111938	-0.145275
8	1	0	5.155541	0.094317	-0.192481
9	6	0	3.367471	1.315685	-0.107464
10	1	0	3.884772	2.269440	-0.124963
11	6	0	1.977668	1.302282	-0.043725
12	1	0	1.464230	2.246218	-0.009547
13	6	0	1.218771	0.110912	-0.015687
14	6	0	-0.949273	1.500970	0.054543
15	6	0	-2.450661	1.282617	-0.119270
16	6	0	-2.599083	-0.217377	0.125720
17	1	0	-3.014889	-0.442050	1.114468
18	1	0	-3.214619	-0.739534	-0.611631
19	6	0	-1.206817	-0.780655	0.084491
20	6	0	-3.237315	2.148090	0.877590
21	1	0	-2.972717	1.906721	1.911825
22	1	0	-4.309264	1.970364	0.747489
23	1	0	-3.038996	3.209320	0.708455
24	6	0	-2.816783	1.665534	-1.573567
25	1	0	-2.594846	2.719794	-1.759871
26	1	0	-3.887730	1.502470	-1.728472
27	1	0	-2.269709	1.060528	-2.303934
28	8	0	1.453826	-2.375453	-0.068545
29	1	0	0.460475	-2.357511	0.009930

1-perpend (in chloroform):

Center Number	Atomic Number	Atomic Type	Coordinates (Angstroms) X	Y	Z
1	8	0	1.215735	1.806668	-1.111124
2	1	0	1.815114	2.398242	-1.587362
3	8	0	-0.872537	-1.223766	-1.580822
4	8	0	-0.202103	1.270074	2.212267
5	7	0	-0.228306	-0.036648	0.299197
6	6	0	3.295548	0.612338	-0.718494
7	6	0	3.942402	-0.481811	-0.141339
8	1	0	5.016521	-0.586177	-0.260882

1-cis (in chloroform):

Center Number	Atomic Number	Atomic Type	Coordinates (Angstroms) X	Y	Z
1	8	0	-0.877857	-1.756173	0.752222
2	8	0	-0.224608	2.401287	-1.074621
3	7	0	-0.220128	0.300757	-0.067814
4	6	0	1.880789	-0.991723	-0.359478
5	6	0	3.277071	-1.041047	-0.259925
6	1	0	3.780154	-1.937172	-0.608366
7	6	0	3.990118	0.026955	0.279369
8	1	0	5.072348	-0.029953	0.346571

9	6	0	3.221026	-1.435286	0.583132
10	6	0	1.840734	-1.284561	0.727071
11	1	0	1.256962	-2.010113	1.284744
12	6	0	1.186891	-0.194331	0.153191
13	6	0	1.911990	0.762888	-0.574045
14	6	0	-1.163200	-0.557009	-0.604659
15	6	0	-2.575268	-0.158643	-0.152604
16	6	0	-2.320340	0.693312	1.113536
17	1	0	-2.668827	1.725915	1.010839
18	1	0	-2.793718	0.281640	2.010388
19	6	0	-0.819310	0.713729	1.322089
20	6	0	-3.379817	-1.437921	0.153815
21	1	0	-3.458210	-2.065651	-0.738048
22	1	0	-4.389910	-1.168919	0.479280
23	1	0	-2.910575	-2.026289	0.949433
24	6	0	-3.255526	0.646815	-1.276972
25	1	0	-2.692794	1.554855	-1.516654
26	1	0	-4.261848	0.940922	-0.961356
27	1	0	-3.339186	0.044401	-2.185663
28	1	0	3.725392	-2.284816	1.031097
29	1	0	3.859676	1.351602	-1.281082

9	6	0	3.313485	1.158206	0.748873
10	1	0	3.860080	1.986897	1.186749
11	6	0	1.925346	1.219513	0.651444
12	1	0	1.391983	2.095658	1.000992
13	6	0	1.203009	0.162009	0.082591
14	6	0	-1.164242	-0.640925	0.325512
15	6	0	-2.575881	-0.084645	0.143718
16	6	0	-2.330901	1.249229	-0.594899
17	1	0	-2.695660	1.229997	-1.627205
18	1	0	-2.792544	2.112049	-0.106004
19	6	0	-0.830832	1.447066	-0.633086
20	6	0	-3.429742	-1.073645	-0.670322
21	1	0	-2.999470	-1.253842	-1.660776
22	1	0	-4.436124	-0.664892	-0.804272
23	1	0	-3.511066	-2.031756	-0.150490
24	6	0	-3.191299	0.133411	1.544612
25	1	0	-3.256303	-0.813095	2.088060
26	1	0	-4.200588	0.543172	1.438206
27	1	0	-2.598835	0.835106	2.140984
28	8	0	1.240459	-2.061922	-0.916971
29	1	0	0.421450	-2.229423	-0.405243

1-trans (in chloroform):

Center Number	Atomic Number	Atomic Type	Coordinates (Angstroms) X	Y	Z
1	8	0	-0.951102	-1.882205	0.791484
2	8	0	-0.182402	2.258969	-1.027252
3	7	0	-0.234944	0.111256	-0.170501
4	6	0	2.093063	0.763779	0.399705
5	6	0	3.447258	0.413977	0.475955
6	1	0	4.136423	1.139139	0.896377
7	6	0	3.889646	-0.824062	0.016213
8	1	0	4.943317	-1.076916	0.085682
9	6	0	2.983778	-1.728260	-0.549302
10	1	0	3.322844	-2.687498	-0.926316
11	6	0	1.634361	-1.392207	-0.627501
12	1	0	0.922484	-2.089376	-1.053392
13	6	0	1.176412	-0.160777	-0.139900
14	6	0	-1.210334	-0.805814	0.295157
15	6	0	-2.606897	-0.211670	0.080894
16	6	0	-2.319644	1.135822	-0.616552
17	1	0	-2.688461	1.167024	-1.647472
18	1	0	-2.747169	1.997747	-0.096125
19	6	0	-0.817641	1.276722	-0.652087
20	6	0	-3.438290	-1.169695	-0.794140
21	1	0	-2.971893	-1.327317	-1.772240
22	1	0	-4.435054	-0.747167	-0.955889
23	1	0	-3.548206	-2.140602	-0.303852
24	6	0	-3.277037	-0.021160	1.457964
25	1	0	-3.370824	-0.980236	1.974230
26	1	0	-4.277855	0.400867	1.322761
27	1	0	-2.701925	0.658956	2.094833
28	8	0	1.718798	1.983444	0.887782
29	1	0	1.050535	2.364991	0.280368

1-TS-cis (in chloroform):

Center Number	Atomic Number	Atomic Type	Coordinates (Angstroms) X	Y	Z
1	8	0	-1.038295	-1.887654	-0.002694
2	8	0	-0.359714	2.691628	-0.039255
3	7	0	-0.228997	0.319997	-0.067297
4	6	0	1.934678	-1.098738	-0.019938
5	6	0	3.343247	-1.092524	-0.006831
6	1	0	3.821402	-2.066560	0.010751
7	6	0	4.090773	0.073333	-0.018667
8	1	0	5.175543	0.026361	-0.008553
9	6	0	3.419535	1.295644	-0.044382
10	1	0	3.962556	2.235091	-0.055240
11	6	0	2.028991	1.319688	-0.054758
12	1	0	1.539924	2.276950	-0.070329
13	6	0	1.237716	0.148988	-0.041311
14	6	0	-1.218200	-0.672510	-0.042921
15	6	0	-2.624952	-0.085879	-0.036737
16	6	0	-2.372010	1.401253	-0.272337
17	1	0	-2.633115	1.715558	-1.289609
18	1	0	-2.898616	2.064900	0.417912
19	6	0	-0.886822	1.600736	-0.109309
20	6	0	-3.472752	-0.745562	-1.139336
21	1	0	-3.029505	-0.595123	-2.129042
22	1	0	-4.473086	-0.301474	-1.141630
23	1	0	-3.569325	-1.819670	-0.963791
24	6	0	-3.246836	-0.351904	1.354129
25	1	0	-3.306525	-1.425543	1.549742
26	1	0	-4.258069	0.065371	1.382058
27	1	0	-2.662335	0.115356	2.153413
28	8	0	1.411245	-2.344512	-0.016038
29	1	0	0.417809	-2.303581	-0.008061

1-TS-trans (in chloroform):

Center Number	Atomic Number	Atomic Type	Coordinates (Angstroms) X	Y	Z
1	8	0	-0.451126	2.592996	0.161498
2	8	0	-0.999343	-1.999513	0.083301
3	7	0	-0.251254	0.233680	0.021396
4	6	0	1.950376	-1.118166	-0.060984
5	6	0	3.357303	-1.071633	-0.109840
6	1	0	3.862936	-2.031397	-0.139768
7	6	0	4.070244	0.115761	-0.124776
8	1	0	5.155265	0.100493	-0.163785
9	6	0	3.364552	1.318312	-0.089040
10	1	0	3.880224	2.273042	-0.099503
11	6	0	1.974763	1.302345	-0.036112
12	1	0	1.459208	2.245193	-0.002313
13	6	0	1.218883	0.108834	-0.017408
14	6	0	-0.952361	1.495867	0.038855
15	6	0	-2.452733	1.274593	-0.147626
16	6	0	-2.600449	-0.224335	0.102676
17	1	0	-3.013412	-0.445504	1.093608
18	1	0	-3.217579	-0.750750	-0.630344
19	6	0	-1.206605	-0.787220	0.060571
20	6	0	-3.249346	2.145014	0.836453
21	1	0	-2.996772	1.908292	1.874823
22	1	0	-4.320542	1.969574	0.695789
23	1	0	-3.045728	3.204961	0.666271
24	6	0	-2.805066	1.649641	-1.607218
25	1	0	-2.578325	2.702062	-1.797816
26	1	0	-3.874978	1.488562	-1.772382
27	1	0	-2.252482	1.039461	-2.329345
28	8	0	1.460819	-2.377446	-0.071954
29	1	0	0.468279	-2.366569	-0.006142

2-perpend (in acetonitrile):

Center Number	Atomic Number	Atomic Type	Coordinates (Angstroms) X	Y	Z
1	8	0	-0.247784	2.654407	-0.255778
2	1	0	0.083101	3.545976	-0.443942
3	8	0	4.950128	-0.858331	-0.175361
4	8	0	3.435764	-2.370035	0.229588
5	8	0	-1.445193	-0.430640	-1.947274
6	8	0	-1.053792	0.478114	2.530173
7	7	0	3.774415	-1.202000	0.000157
8	7	0	-0.948556	0.068474	0.254810
9	6	0	2.108274	2.113840	-0.391562
10	6	0	3.098607	1.144648	-0.325841
11	1	0	4.138277	1.407525	-0.473247
12	6	0	2.740533	-0.182396	-0.066762
13	6	0	1.407987	-0.553797	0.129086
14	1	0	1.146539	-1.584849	0.329775
15	6	0	0.421619	0.418546	0.062486
16	6	0	0.762168	1.762668	-0.200649
17	6	0	-1.800053	-0.324305	-0.787183
18	6	0	-3.198996	-0.586334	-0.217557
19	6	0	-3.039876	-0.272041	1.290200
20	1	0	-3.686146	0.544643	1.626231
21	1	0	-3.250037	-1.134289	1.930669
22	6	0	-1.598075	0.140235	1.494073
23	6	0	-3.575946	-2.059658	-0.473136
24	1	0	-3.593577	-2.271916	-1.545655
25	1	0	-4.571383	-2.258111	-0.063880
26	1	0	-2.866834	-2.743574	0.004354
27	6	0	-4.205939	0.355154	-0.908748
28	1	0	-3.948982	1.406782	-0.745952
29	1	0	-5.206928	0.180551	-0.502186
30	1	0	-4.231962	0.167350	-1.985613
31	1	0	2.372117	3.147127	-0.592542

2-cis (in acetonitrile):

Center Number	Atomic Number	Atomic Type	Coordinates (Angstroms) X	Y	Z
1	8	0	0.232624	2.738248	-0.504975
2	1	0	1.038579	2.502173	0.011271
3	8	0	-4.995873	-0.591809	0.331486
4	8	0	-3.520654	-2.171427	0.595852
5	8	0	2.031967	1.523900	1.046715
6	8	0	0.470744	-1.875105	-1.604855
7	7	0	-3.826102	-0.994513	0.364196
8	7	0	0.931138	-0.083572	-0.190768
9	6	0	-2.052689	2.208025	-0.321822
10	6	0	-3.082531	1.305516	-0.121437
11	1	0	-4.116105	1.626462	-0.130774
12	6	0	-2.760967	-0.035452	0.118646
13	6	0	-1.440517	-0.481086	0.130914
14	1	0	-1.227408	-1.525561	0.308137
15	6	0	-0.407551	0.424087	-0.104745
16	6	0	-0.708688	1.794186	-0.303525
17	6	0	2.048783	0.457654	0.435292
18	6	0	3.259599	-0.448259	0.235661
19	6	0	2.743105	-1.504689	-0.766689
20	1	0	3.220589	-1.419996	-1.748400
21	1	0	2.883644	-2.534622	-0.426393
22	6	0	1.265962	-1.242600	-0.941608
23	6	0	3.623961	-1.074887	1.601519
24	1	0	3.885527	-0.297577	2.324578
25	1	0	4.486957	-1.735269	1.474609
26	1	0	2.797184	-1.665854	2.008309
27	6	0	4.441697	0.371105	-0.314517
28	1	0	4.193260	0.838734	-1.272505
29	1	0	5.300689	-0.289038	-0.467636
30	1	0	4.728825	1.155330	0.390658
31	1	0	-2.258449	3.259751	-0.485760

2-trans (in acetonitrile):

Center Number	Atomic Number	Atomic Type	Coordinates (Angstroms) X	Y	Z
1	8	0	0.101367	2.935604	0.541816
2	1	0	-0.656478	2.885209	-0.086215
3	8	0	4.806082	-1.146384	-0.040976
4	8	0	3.147530	-2.424645	-0.638144
5	8	0	-0.996959	-1.635897	1.128299
6	8	0	-1.646580	2.176213	-1.333911
7	7	0	3.601805	-1.340826	-0.249465
8	7	0	-0.983740	0.310644	-0.143386
9	6	0	2.280248	2.045199	0.564908
10	6	0	3.172695	1.002073	0.388582
11	1	0	4.233868	1.141780	0.549314
12	6	0	2.678343	-0.239864	-0.026909
13	6	0	1.316974	-0.453569	-0.235204
14	1	0	0.966518	-1.428143	-0.543886
15	6	0	0.417694	0.589666	-0.024263
16	6	0	0.901744	1.867176	0.350701
17	6	0	-1.594608	-0.827810	0.448866
18	6	0	-3.084603	-0.838533	0.098613
19	6	0	-3.242525	0.384650	-0.832379
20	1	0	-3.474875	0.101221	-1.864862
21	1	0	-4.016312	1.085842	-0.508056
22	6	0	-1.909444	1.084577	-0.833297
23	6	0	-3.897254	-0.681446	1.402373
24	1	0	-3.687790	-1.508861	2.085547
25	1	0	-4.965453	-0.687768	1.165813
26	1	0	-3.665228	0.258288	1.913652
27	6	0	-3.436266	-2.167806	-0.597329
28	1	0	-2.856476	-2.306463	-1.515280
29	1	0	-4.498719	-2.170739	-0.859230
30	1	0	-3.241168	-3.013265	0.067603
31	1	0	2.626138	3.029179	0.860902

2-TS-cis (in acetonitrile):

Center Number	Atomic Number	Atomic Type	Coordinates (Angstroms) X	Y	Z
1	8	0	-1.018001	-1.876894	0.066466
2	8	0	-0.326348	2.690027	-0.243318
3	7	0	-0.219186	0.326677	-0.089140
4	6	0	1.939739	-1.093438	0.018789
5	6	0	3.354410	-1.094464	0.023890
6	1	0	3.830101	-2.066041	0.095252
7	6	0	4.108482	0.054457	-0.055426
8	1	0	5.190162	0.024301	-0.049124
9	6	0	3.416320	1.264018	-0.145009
10	6	0	2.024816	1.313737	-0.153221
11	1	0	1.556103	2.278592	-0.224032
12	6	0	1.238597	0.157609	-0.072405
13	6	0	-1.207149	-0.663779	-0.012477
14	6	0	-2.612065	-0.079987	-0.019222
15	6	0	-2.365215	1.414640	-0.222757
16	1	0	-2.723973	1.779342	-1.190951
17	1	0	-2.815609	2.046691	0.547572
18	6	0	-0.873542	1.608575	-0.187707
19	6	0	-3.426074	-0.717054	-1.163291
20	1	0	-2.962657	-0.529519	-2.136928
21	1	0	-4.430498	-0.283417	-1.172752
22	1	0	-3.514320	-1.796812	-1.020744
23	6	0	-3.270782	-0.384027	1.345945
24	1	0	-3.346449	-1.462474	1.505742
25	1	0	-4.277992	0.042781	1.357778
26	1	0	-2.703485	0.053551	2.173498
27	8	0	1.410520	-2.314356	0.102413
28	1	0	0.405829	-2.268394	0.094661
29	7	0	4.153199	2.508614	-0.232567
30	8	0	5.392380	2.456283	-0.225921
31	8	0	3.525694	3.574933	-0.310516

2-TS-trans (in acetonitrile):

Center Number	Atomic Number	Atomic Type	Coordinates (Angstroms) X	Y	Z
1	8	0	-0.404627	2.583786	-0.283695
2	8	0	-0.968721	-1.978740	0.261398
3	7	0	-0.230370	0.238721	-0.009095
4	6	0	1.966377	-1.120894	0.080921
5	6	0	3.380004	-1.087486	0.037212
6	1	0	3.882664	-2.043476	0.130794
7	6	0	4.101216	0.075344	-0.113294
8	1	0	5.182923	0.071805	-0.143183
9	6	0	3.375715	1.263265	-0.225516
10	6	0	1.983814	1.278860	-0.188645
11	1	0	1.488615	2.228230	-0.283187
12	6	0	1.230829	0.108143	-0.036569
13	6	0	-0.925559	1.500338	-0.125865
14	6	0	-2.434051	1.299448	-0.013206
15	6	0	-2.578392	-0.218645	0.087304
16	1	0	-3.108575	-0.555937	0.982853
17	1	0	-3.088278	-0.662148	-0.774310
18	6	0	-1.185359	-0.774109	0.127657
19	6	0	-2.927851	2.022035	1.259657
20	1	0	-2.449436	1.623759	2.160240
21	1	0	-4.008883	1.882957	1.355764
22	1	0	-2.718137	3.093040	1.198726
23	6	0	-3.117424	1.883812	-1.265418
24	1	0	-2.920079	2.955568	-1.346578
25	1	0	-4.198584	1.733123	-1.190576
26	1	0	-2.765052	1.394203	-2.178919
27	8	0	1.471262	-2.349158	0.233694
28	1	0	0.465383	-2.328599	0.261016
29	7	0	4.076879	2.521050	-0.385918
30	8	0	5.316510	2.499415	-0.420843
31	8	0	3.419757	3.567936	-0.481886

2-perpend (in acetone):

Center Number	Atomic Number	Atomic Type	Coordinates (Angstroms) X	Y	Z
1	8	0	-0.261391	2.647777	-0.186927
2	1	0	0.064025	3.545757	-0.352412
3	8	0	4.959099	-0.832104	-0.197563
4	8	0	3.454219	-2.364586	0.162937
5	8	0	-1.467617	-0.329093	-1.964908
6	8	0	-1.026042	0.327463	2.552137
7	7	0	3.785669	-1.188792	-0.034144
8	7	0	-0.945646	0.045328	0.256168
9	6	0	2.097445	2.124983	-0.341895
10	6	0	3.094154	1.160868	-0.301173
11	1	0	4.132166	1.433788	-0.441920
12	6	0	2.744699	-0.174482	-0.075900
13	6	0	1.414643	-0.559511	0.109743
14	1	0	1.160135	-1.597231	0.283318
15	6	0	0.421960	0.407950	0.069020
16	6	0	0.753969	1.760563	-0.158273
17	6	0	-1.809756	-0.285839	-0.797181

2-cis (in acetone):

Center Number	Atomic Number	Atomic Type	Coordinates (Angstroms) X	Y	Z
1	8	0	0.233805	2.737368	-0.504911
2	1	0	1.038345	2.502569	0.014075
3	8	0	-4.996802	-0.590571	0.328163
4	8	0	-3.522249	-2.169983	0.598122
5	8	0	2.032822	1.523668	1.046526
6	8	0	0.469848	-1.877783	-1.601367
7	7	0	-3.827445	-0.993742	0.363760
8	7	0	0.930883	-0.084489	-0.189531
9	6	0	-2.051979	2.208151	-0.321896
10	6	0	-3.082317	1.306039	-0.121861
11	1	0	-4.115876	1.627101	-0.131531
12	6	0	-2.761407	-0.034941	0.118390
13	6	0	-1.441271	-0.481132	0.131084
14	1	0	-1.229031	-1.525940	0.307448
15	6	0	-0.407746	0.423708	-0.104037
16	6	0	-0.708165	1.793838	-0.303161
17	6	0	2.048994	0.457393	0.435413

Center Number	Atomic Number	Atomic Type	X	Y	Z
18	6	0	-3.204808	-0.570031	-0.228289
19	6	0	-3.022174	-0.365429	1.295692
20	1	0	-3.671604	0.414903	1.703761
21	1	0	-3.210102	-1.276430	1.872626
22	6	0	-1.580671	0.045004	1.505229
23	6	0	-3.611534	-2.013882	-0.584231
24	1	0	-3.646360	-2.148083	-1.668852
25	1	0	-4.605227	-2.224318	-0.176686
26	1	0	-2.909482	-2.743036	-0.167269
27	6	0	-4.204178	0.435650	-0.836171
28	1	0	-3.930804	1.468251	-0.596720
29	1	0	-5.204033	0.244124	-0.434362
30	1	0	-4.241964	0.331014	-1.923909
31	1	0	2.354450	3.164786	-0.516570

Center Number	Atomic Number	Atomic Type	X	Y	Z
18	6	0	3.260148	-0.448084	0.234760
19	6	0	2.742116	-1.507772	-0.763252
20	1	0	3.219696	-1.428610	-1.745344
21	1	0	2.880963	-2.536472	-0.418394
22	6	0	1.265054	-1.244787	-0.938906
23	6	0	3.629787	-1.070203	1.601116
24	1	0	3.892012	-0.290475	2.321304
25	1	0	4.493528	-1.729565	1.473704
26	1	0	2.805223	-1.661473	2.012036
27	6	0	4.439325	0.371511	-0.321595
28	1	0	4.187460	0.835155	-1.280651
29	1	0	5.299192	-0.287506	-0.474780
30	1	0	4.726828	1.158915	0.379851
31	1	0	-2.257048	3.260009	-0.485921

2-trans (in acetone):

Center Number	Atomic Number	Atomic Type	Coordinates (Angstroms) X	Y	Z
1	8	0	0.101070	2.935811	0.540926
2	1	0	-0.655184	2.886134	-0.089129
3	8	0	4.805961	-1.146950	-0.038144
4	8	0	3.148149	-2.423448	-0.641368
5	8	0	-0.997038	-1.636484	1.127653
6	8	0	-1.647571	2.176748	-1.332992
7	7	0	3.602293	-1.340942	-0.249504
8	7	0	-0.983732	0.310677	-0.143482
9	6	0	2.280149	2.045365	0.564586
10	6	0	3.172663	1.002129	0.388651
11	1	0	4.233886	1.141648	0.549328
12	6	0	2.678422	-0.239745	-0.026674
13	6	0	1.317160	-0.453632	-0.234805
14	1	0	0.967002	-1.428621	-0.542505
15	6	0	0.417850	0.589731	-0.024273
16	6	0	0.901691	1.867414	0.350294
17	6	0	-1.594476	-0.828483	0.448365
18	6	0	-3.084709	-0.838795	0.098704
19	6	0	-3.242880	0.384910	-0.831480
20	1	0	-3.475821	0.102334	-1.864089
21	1	0	-4.016444	1.086085	-0.506524
22	6	0	-1.909620	1.084890	-0.832542
23	6	0	-3.896644	-0.682431	1.402967
24	1	0	-3.686145	-1.509779	2.085867
25	1	0	-4.965082	-0.689194	1.167360
26	1	0	-3.664694	0.257303	1.914377
27	6	0	-3.436468	-2.167766	-0.597760
28	1	0	-2.857123	-2.305855	-1.516136
29	1	0	-4.499073	-2.171115	-0.859162
30	1	0	-3.240476	-3.013494	0.066526
31	1	0	2.625843	3.029478	0.860186

2-TS-cis (in acetone):

Center Number	Atomic Number	Atomic Type	Coordinates (Angstroms) X	Y	Z
1	8	0	-1.016073	-1.853751	0.298992
2	8	0	-0.331527	2.676022	-0.367268
3	7	0	-0.219134	0.332633	-0.023110
4	6	0	1.940229	-1.083856	0.112072
5	6	0	3.354414	-1.088446	0.077118
6	1	0	3.830811	-2.054975	0.196341
7	6	0	4.107002	0.051288	-0.095130
8	1	0	5.188416	0.018993	-0.117660
9	6	0	3.413960	1.254952	-0.239098
10	6	0	2.022919	1.308005	-0.212521
11	1	0	1.553719	2.267823	-0.332820
12	6	0	1.238232	0.160784	-0.039135
13	6	0	-1.205931	-0.650557	0.127018
14	6	0	-2.611193	-0.075098	0.033161
15	6	0	-2.362438	1.431888	-0.024599
16	1	0	-2.860019	1.935564	-0.857338
17	1	0	-2.666914	1.945153	0.894083
18	6	0	-0.875086	1.609898	-0.169289
19	6	0	-3.263931	-0.608924	-1.263776
20	1	0	-2.696848	-0.316220	-2.153124
21	1	0	-4.273192	-0.195838	-1.350395
22	1	0	-3.334253	-1.699277	-1.238335
23	6	0	-3.431969	-0.506610	1.263785
24	1	0	-3.525149	-1.594412	1.305379
25	1	0	-4.434468	-0.073593	1.195938
26	1	0	-2.971116	-0.159880	2.194008
27	8	0	1.412765	-2.295423	0.290723
28	1	0	0.408810	-2.247303	0.315785
29	7	0	4.150173	2.490147	-0.422428
30	8	0	5.388735	2.433750	-0.447560
31	8	0	3.522062	3.551431	-0.546316

2-TS-trans (in acetone):

Center Number	Atomic Number	Atomic Type	Coordinates (Angstroms) X	Y	Z
1	8	0	-0.406061	2.580479	-0.313703
2	8	0	-0.969872	-1.977943	0.265038
3	7	0	-0.230958	0.238059	-0.016136
4	6	0	1.966076	-1.119659	0.091871
5	6	0	3.379765	-1.085778	0.053124
6	1	0	3.882390	-2.040869	0.155639
7	6	0	4.100914	0.076410	-0.103120
8	1	0	5.182739	0.073540	-0.129012
9	6	0	3.375351	1.263059	-0.226627
10	6	0	1.983447	1.278340	-0.195444
11	1	0	1.488430	2.226922	-0.299148
12	6	0	1.230563	0.108087	-0.037748
13	6	0	-0.926426	1.499004	-0.142963
14	6	0	-2.434309	1.299168	-0.019384
15	6	0	-2.579145	-0.219510	0.070563
16	1	0	-3.120895	-0.563725	0.956341
17	1	0	-3.077450	-0.656957	-0.801059
18	6	0	-1.186006	-0.774289	0.122878
19	6	0	-2.913305	2.010094	1.266094
20	1	0	-2.427364	1.601089	2.157832
21	1	0	-3.993779	1.873253	1.371513
22	1	0	-2.700750	3.081012	1.214269
23	6	0	-3.130102	1.896212	-1.258101
24	1	0	-2.933098	2.968610	-1.330653
25	1	0	-4.210579	1.745266	-1.174065
26	1	0	-2.787102	1.415847	-2.180098
27	8	0	1.471224	-2.347159	0.252634
28	1	0	0.465569	-2.327695	0.274890
29	7	0	4.077023	2.520444	-0.393118
30	8	0	5.316598	2.498430	-0.422771
31	8	0	3.419681	3.565907	-0.499003

2-perpend (in chloroform):

Center Number	Atomic Number	Atomic Type	Coordinates (Angstroms) X	Y	Z
1	8	0	-0.269130	2.640871	-0.189567
2	1	0	0.055196	3.538456	-0.355484
3	8	0	4.963219	-0.822366	-0.198919
4	8	0	3.463465	-2.359115	0.170674
5	8	0	-1.465470	-0.342730	-1.963067
6	8	0	-1.031679	0.335230	2.553295
7	7	0	3.793176	-1.185011	-0.031745
8	7	0	-0.944310	0.038081	0.258300
9	6	0	2.091309	2.123340	-0.347292
10	6	0	3.091805	1.162363	-0.305520
11	1	0	4.129444	1.435821	-0.448562
12	6	0	2.746668	-0.172348	-0.075750
13	6	0	1.418553	-0.560596	0.112615
14	1	0	1.169695	-1.599195	0.289319
15	6	0	0.422173	0.403246	0.070600
16	6	0	0.749967	1.755373	-0.160278
17	6	0	-1.807866	-0.292720	-0.797140
18	6	0	-3.205686	-0.569453	-0.228632
19	6	0	-3.027037	-0.353646	1.294085
20	1	0	-3.671877	0.435058	1.693546
21	1	0	-3.223832	-1.257559	1.879300
22	6	0	-1.582540	0.050098	1.506854
23	6	0	-3.613364	-2.015080	-0.575540
24	1	0	-3.640033	-2.157977	-1.659182
25	1	0	-4.610070	-2.221913	-0.172988
26	1	0	-2.915223	-2.742656	-0.148803
27	6	0	-4.200852	0.433315	-0.847269
28	1	0	-3.927354	1.466939	-0.611698
29	1	0	-5.203781	0.245544	-0.450744
30	1	0	-4.231336	0.323929	-1.934675
31	1	0	2.345099	3.163600	-0.525617

2-cis (in chloroform):

Center Number	Atomic Number	Atomic Type	Coordinates (Angstroms) X	Y	Z
1	8	0	0.239367	2.733763	-0.503280

2-trans (in chloroform):

Center Number	Atomic Number	Atomic Type	Coordinates (Angstroms) X	Y	Z
1	8	0	0.099626	2.936947	0.538228

Center Number	Atomic Number	Atomic Type	X	Y	Z
2	1	0	1.036846	2.504747	0.028903
3	8	0	-5.001217	-0.584475	0.309513
4	8	0	-3.529369	-2.162031	0.611402
5	8	0	2.038843	1.522976	1.044282
6	8	0	0.464816	-1.893459	-1.581160
7	7	0	-3.834405	-0.990154	0.361457
8	7	0	0.929586	-0.089004	-0.182774
9	6	0	-2.048162	2.209144	-0.322346
10	6	0	-3.081034	1.308806	-0.124604
11	1	0	-4.114649	1.629974	-0.136433
12	6	0	-2.763624	-0.032157	0.116760
13	6	0	-1.445233	-0.481240	0.131782
14	1	0	-1.238357	-1.527899	0.303895
15	6	0	-0.408945	0.422024	-0.100273
16	6	0	-0.705402	1.792576	-0.300843
17	6	0	2.049881	0.456325	0.434834
18	6	0	3.262857	-0.447218	0.229363
19	6	0	2.737937	-1.521773	-0.748463
20	1	0	3.213828	-1.465571	-1.732898
21	1	0	2.871463	-2.544920	-0.384778
22	6	0	1.260238	-1.256543	-0.925463
23	6	0	3.656039	-1.048159	1.597995
24	1	0	3.921589	-0.257061	2.304305
25	1	0	4.522505	-1.703957	1.469087
26	1	0	2.841125	-1.639190	2.028509
27	6	0	4.428485	0.372998	-0.354469
28	1	0	4.161092	0.819324	-1.317739
29	1	0	5.292592	-0.280472	-0.508590
30	1	0	4.717266	1.174539	0.330132
31	1	0	-2.249312	3.261700	-0.486868

Center Number	Atomic Number	Atomic Type	X	Y	Z
2	1	0	-0.648041	2.893007	-0.102271
3	8	0	4.804988	-1.149010	-0.023726
4	8	0	3.150845	-2.417646	-0.656510
5	8	0	-0.996215	-1.642455	1.119715
6	8	0	-1.653832	2.179608	-1.328606
7	7	0	3.604822	-1.341963	-0.249511
8	7	0	-0.983703	0.311130	-0.143787
9	6	0	2.279612	2.046760	0.561991
10	6	0	3.172386	1.002857	0.387883
11	1	0	4.234049	1.140779	0.547811
12	6	0	2.678625	-0.239153	-0.025611
13	6	0	1.318052	-0.453938	-0.232314
14	1	0	0.969759	-1.431555	-0.533902
15	6	0	0.418522	0.590420	-0.024319
16	6	0	0.901424	1.868731	0.347986
17	6	0	-1.593386	-0.831703	0.446033
18	6	0	-3.085082	-0.840110	0.099462
19	6	0	-3.244404	0.384753	-0.828522
20	1	0	-3.476992	0.104254	-1.861947
21	1	0	-4.018865	1.085195	-0.503759
22	6	0	-1.910817	1.087006	-0.828819
23	6	0	-3.892251	-0.684582	1.406632
24	1	0	-3.675869	-1.510163	2.089650
25	1	0	-4.962189	-0.693746	1.177100
26	1	0	-3.659920	0.256028	1.916759
27	6	0	-3.438564	-2.168509	-0.596721
28	1	0	-2.862471	-2.305519	-1.517621
29	1	0	-4.502225	-2.174640	-0.854569
30	1	0	-3.237086	-3.014538	0.065337
31	1	0	2.624004	3.031989	0.855605

2-TS-cis (in chloroform):

Center Number	Atomic Number	Atomic Type	X	Y	Z
1	8	0	-1.022777	-1.882765	0.010227
2	8	0	-0.333685	2.690765	-0.190296
3	7	0	-0.221083	0.323025	-0.103636
4	6	0	1.938689	-1.096869	-0.013826
5	6	0	3.352834	-1.095843	-0.001296
6	1	0	3.828112	-2.068918	0.049600
7	6	0	4.105660	0.056160	-0.050437
8	1	0	5.187503	0.029457	-0.039737
9	6	0	3.413230	1.266308	-0.115656
10	6	0	2.022823	1.315823	-0.128613
11	1	0	1.555576	2.283236	-0.177753
12	6	0	1.238088	0.155404	-0.078520
13	6	0	-1.208869	-0.668773	-0.047476
14	6	0	-2.613244	-0.081109	-0.035400
15	6	0	-2.365399	1.405928	-0.287318
16	1	0	-2.670892	1.721347	-1.291355
17	1	0	-2.860228	2.071278	0.424837
18	6	0	-0.876341	1.607533	-0.186924
19	6	0	-3.468730	-0.750864	-1.126965
20	1	0	-3.034526	-0.606609	-2.121498
21	1	0	-4.469505	-0.308316	-1.123201
22	1	0	-3.561955	-1.823624	-0.942169
23	6	0	-3.222987	-0.337857	1.362947
24	1	0	-3.291276	-1.410225	1.561992
25	1	0	-4.229508	0.089560	1.398876
26	1	0	-2.625806	0.126207	2.154540
27	8	0	1.412069	-2.321693	0.036354
28	1	0	0.409857	-2.280886	0.029391
29	7	0	4.152539	2.516376	-0.172092
30	8	0	5.390074	2.459883	-0.159299
31	8	0	3.522767	3.580232	-0.230800

2-TS-trans (in chloroform):

Center Number	Atomic Number	Atomic Type	X	Y	Z
1	8	0	-0.414886	2.559773	-0.460449
2	8	0	-0.976404	-1.976062	0.273324
3	7	0	-0.233808	0.233491	-0.050961
4	6	0	1.964430	-1.112044	0.141680
5	6	0	3.378030	-1.073957	0.128376
6	1	0	3.880365	-2.023739	0.273071
7	6	0	4.098197	0.085622	-0.053214
8	1	0	5.180402	0.088030	-0.058992
9	6	0	3.372261	1.264567	-0.229969
10	6	0	1.980885	1.276737	-0.227117
11	1	0	1.487033	2.220655	-0.374187
12	6	0	1.229259	0.107878	-0.044859
13	6	0	-0.931018	1.490022	-0.225697
14	6	0	-2.435084	1.295868	-0.047836
15	6	0	-2.581924	-0.224571	-0.005623
16	1	0	-3.170151	-0.598852	0.836727
17	1	0	-3.030170	-0.633468	-0.918232
18	6	0	-1.188916	-0.777133	0.096755
19	6	0	-2.843188	1.953465	1.291374
20	1	0	-2.323160	1.499993	2.141509
21	1	0	-3.919629	1.825635	1.441751
22	1	0	-2.617571	3.022867	1.277624
23	6	0	-3.189402	1.949923	-1.218870
24	1	0	-2.993723	3.024399	-1.250864
25	1	0	-4.265190	1.795584	-1.089979
26	1	0	-2.893415	1.516309	-2.179514
27	8	0	1.471649	-2.335978	0.338745
28	1	0	0.468169	-2.325009	0.334150
29	7	0	4.076625	2.520895	-0.424098
30	8	0	5.315259	2.496966	-0.429597
31	8	0	3.417647	3.557780	-0.574494

3-perpend (in acetonitrile):

Center Number	Atomic Number	Atomic Type	X	Y	Z
1	17	0	2.890611	2.914943	-0.232497
2	8	0	-0.046601	2.331504	0.072583
3	1	0	0.474233	3.149716	-0.002970
4	8	0	4.348271	-2.134220	-0.187701
5	8	0	2.554429	-3.351381	0.025562
6	8	0	-1.772223	-0.265631	-1.972373
7	8	0	-1.357439	0.083543	2.581312
8	7	0	3.124640	-2.258930	-0.067160
9	7	0	-1.261280	-0.100477	0.276405
10	6	0	2.154347	1.331942	-0.103246
11	6	0	2.938018	0.190199	-0.137870
12	1	0	4.012778	0.255639	-0.244396
13	6	0	2.309079	-1.051967	-0.031964
14	6	0	0.925491	-1.169666	0.107009
15	1	0	0.458760	-2.142688	0.188365
16	6	0	0.155380	-0.016952	0.139755
17	6	0	0.753110	1.256343	0.035174
18	6	0	-2.138371	-0.215048	-0.812607
19	6	0	-3.577655	-0.270545	-0.288254
20	6	0	-3.410735	-0.128645	1.244650
21	1	0	-3.892849	0.769436	1.643219
22	1	0	-3.810261	-0.983110	1.799303
23	6	0	-1.924331	-0.032042	1.509941
24	6	0	-4.198068	-1.626288	-0.685223

3-cis (in acetonitrile):

Center Number	Atomic Number	Atomic Type	X	Y	Z
1	17	0	2.826471	3.009585	0.245707
2	8	0	-0.023391	2.402632	0.259810
3	1	0	-0.870786	2.283246	-0.236519
4	8	0	4.441649	-1.936812	-0.356678
5	8	0	2.681506	-3.212230	-0.490667
6	8	0	-2.054999	1.472924	-1.157087
7	8	0	-1.106441	-1.970199	1.717509
8	7	0	3.217380	-2.107067	-0.352286
9	7	0	-1.251153	-0.230708	0.175711
10	6	0	2.122908	1.416809	0.102905
11	6	0	2.951212	0.317034	-0.038565
12	1	0	4.027060	0.427708	-0.061364
13	6	0	2.361004	-0.941188	-0.174289
14	6	0	0.980999	-1.117599	-0.142997
15	1	0	0.560472	-2.108084	-0.238959
16	6	0	0.157751	-0.007915	0.032986
17	6	0	0.714595	1.291570	0.126601
18	6	0	-2.260443	0.471947	-0.473169
19	6	0	-3.615551	-0.169494	-0.195940
20	6	0	-3.287020	-1.225876	0.882429
21	1	0	-3.712523	-0.972413	1.858948
22	1	0	-3.634508	-2.231369	0.628840
23	6	0	-1.783477	-1.246180	1.019088
24	6	0	-4.119045	-0.817137	-1.507181

25	1	0	-4.221841	-1.734639	-1.773041
26	1	0	-5.224118	-1.682070	-0.308675
27	1	0	-3.633556	-2.465176	-0.265202
28	6	0	-4.383749	0.891485	-0.901866
29	1	0	-3.951561	1.862288	-0.638995
30	1	0	-5.411267	0.861494	-0.526218
31	1	0	-4.410818	0.807520	-1.991696

25	1	0	-4.245731	-0.060535	-2.286180
26	1	0	-5.087604	-1.291344	-1.322906
27	1	0	-3.426823	-1.582077	-1.872769
28	6	0	-4.611595	0.897287	0.294654
29	1	0	-4.258656	1.384001	1.209155
30	1	0	-5.574063	0.422927	0.508255
31	1	0	-4.765041	1.663344	-0.469729

3-trans (in acetonitrile):

Center Number	Atomic Number	Atomic Type	Coordinates (Angstroms) X	Y	Z
1	17	0	-3.311156	-2.591300	0.505446
2	8	0	-0.422276	-2.559250	0.134674
3	1	0	0.358430	-2.618136	-0.470317
4	8	0	-4.001722	2.566870	-0.094411
5	8	0	-2.059514	3.467533	-0.494662
6	8	0	1.671130	1.536613	1.255451
7	8	0	1.548869	-2.078714	-1.561509
8	7	0	-2.777394	2.492118	-0.247401
9	7	0	1.271927	-0.222904	-0.212883
10	6	0	-2.337474	-1.170962	0.210083
11	6	0	-2.948086	0.068456	0.129207
12	1	0	-4.018644	0.172239	0.245552
13	6	0	-2.147794	1.183077	-0.130265
14	6	0	-0.767779	1.083604	-0.279937
15	1	0	-0.179499	1.970162	-0.466925
16	6	0	-0.159605	-0.164294	-0.165675
17	6	0	-0.941053	-1.326290	0.050898
18	6	0	2.100291	0.678933	0.513882
19	6	0	3.567151	0.366194	0.212512
20	6	0	3.484658	-0.758139	-0.844248
21	1	0	3.819765	-0.429447	-1.834392
22	1	0	4.064278	-1.648408	-0.585970
23	6	0	2.029247	-1.126420	-0.949244
24	6	0	4.242423	-0.116015	1.515365
25	1	0	4.194421	0.661937	2.281949
26	1	0	5.293838	-0.342339	1.315030
27	1	0	3.765629	-1.020108	1.907386
28	6	0	4.262643	1.634824	-0.318109
29	1	0	3.781287	2.006752	-1.228247
30	1	0	5.306588	1.405031	-0.551931
31	1	0	4.242203	2.427676	0.434104

3-TS-cis (in acetonitrile):

Center Number	Atomic Number	Atomic Type	Coordinates (Angstroms) X	Y	Z
1	8	0	-1.025520	-1.882726	0.057535
2	8	0	-0.322131	2.685772	-0.225279
3	7	0	-0.216932	0.318730	-0.090034
4	6	0	1.930677	-1.106811	0.011878
5	6	0	3.354481	-1.093648	0.016964
6	6	0	4.107517	0.057457	-0.057582
7	1	0	5.188471	0.028559	-0.050918
8	6	0	3.410314	1.259557	-0.142427
9	6	0	2.021072	1.311145	-0.150622
10	1	0	1.551057	2.275486	-0.217342
11	6	0	1.241298	0.152337	-0.073985
12	6	0	-1.209121	-0.667896	-0.017453
13	6	0	-2.611599	-0.080623	-0.021242
14	6	0	-2.361428	1.412395	-0.230130
15	1	0	-2.709239	1.771060	-1.204692
16	1	0	-2.819055	2.049968	0.531119
17	6	0	-0.870690	1.605489	-0.182586
18	6	0	-3.432649	-0.719756	-1.159052
19	1	0	-2.971743	-0.539820	-2.135282
20	1	0	-4.434642	-0.280641	-1.166836
21	1	0	-3.526186	-1.798156	-1.010282
22	6	0	-3.264291	-0.377967	1.348759
23	1	0	-3.342188	-1.455568	1.512721
24	1	0	-4.270128	0.051806	1.363221
25	1	0	-2.691958	0.061223	2.171900
26	8	0	1.388119	-2.312771	0.087279
27	1	0	0.377841	-2.254446	0.079881
28	7	0	4.154010	2.504532	-0.225791
29	8	0	5.391250	2.446231	-0.219302
30	8	0	3.526563	3.568971	-0.299724
31	17	0	4.187254	-2.630946	0.123593

3-TS-trans (in acetonitrile):

Center Number	Atomic Number	Atomic Type	Coordinates (Angstroms) X	Y	Z
1	8	0	-0.415823	2.579209	-0.041609
2	8	0	-0.977996	-2.014034	0.087096
3	7	0	-0.233959	0.215936	-0.031171
4	6	0	1.955171	-1.144248	-0.029292
5	6	0	3.377345	-1.089234	-0.065848
6	6	0	4.094332	0.085781	-0.122882
7	1	0	5.175409	0.088914	-0.148972
8	6	0	3.360620	1.268854	-0.145061
9	6	0	1.970746	1.279497	-0.110780
10	1	0	1.471793	2.231307	-0.127983
11	6	0	1.227804	0.096000	-0.051933
12	6	0	-0.933808	1.484730	-0.074968
13	6	0	-2.437297	1.260225	-0.195285
14	6	0	-2.580768	-0.238767	0.064938
15	1	0	-3.000916	-0.457623	1.053084
16	1	0	-3.190910	-0.769313	-0.670807
17	6	0	-1.191277	-0.801932	0.036787
18	6	0	-3.182857	2.131555	0.830197
19	1	0	-2.878409	1.892627	1.853945
20	1	0	-4.258902	1.954507	0.741546
21	1	0	-2.989377	3.191530	0.648493
22	6	0	-2.857693	1.638624	-1.635800
23	1	0	-2.647323	2.693331	-1.831189
24	1	0	-3.932669	1.470107	-1.749845
25	1	0	-2.334837	1.034394	-2.384094
26	8	0	1.449006	-2.367223	0.018571
27	1	0	0.437436	-2.339626	0.051671
28	7	0	4.065116	2.537639	-0.206314
29	8	0	5.302995	2.516172	-0.243677
30	8	0	3.405187	3.584812	-0.218979
31	17	0	4.256248	-2.604165	-0.038673

3-perpend (in acetone):

Center Number	Atomic Number	Atomic Type	Coordinates (Angstroms) X	Y	Z
1	17	0	2.887201	2.917092	-0.229613
2	8	0	-0.048858	2.330234	0.075399
3	1	0	0.471671	3.148629	0.000290
4	8	0	4.350566	-2.130691	-0.191642
5	8	0	2.558171	-3.349930	0.023271
6	8	0	-1.772209	-0.259069	-1.972830
7	8	0	-1.357567	0.071979	2.582476
8	7	0	3.127383	-2.257136	-0.069673
9	7	0	-1.261042	-0.103385	0.276732
10	6	0	2.152879	1.332894	-0.101837
11	6	0	2.937935	0.192100	-0.137824
12	1	0	4.012623	0.258658	-0.244572
13	6	0	2.310386	-1.050767	-0.032970
14	6	0	0.927016	-1.170028	0.106155
15	1	0	0.461656	-2.143785	0.186440
16	6	0	0.155513	-0.018269	0.140191
17	6	0	0.751853	1.255666	0.036796
18	6	0	-2.138137	-0.212990	-0.813035
19	6	0	-3.577553	-0.270201	-0.288780
20	6	0	-3.410710	-0.134092	1.244671
21	1	0	-3.892368	0.762667	1.646783
22	1	0	-3.810723	-0.990392	1.796180
23	6	0	-1.924085	-0.039209	1.510689
24	6	0	-4.198337	-1.624204	-0.690986
25	1	0	-4.221213	-1.728715	-1.779179
26	1	0	-5.224724	-1.681021	-0.315433
27	1	0	-3.634634	-2.465037	-0.273664
28	6	0	-4.383187	0.894184	-0.898432
29	1	0	-3.950878	1.863945	-0.631987
30	1	0	-5.410973	0.863200	-0.523520
31	1	0	-4.409489	0.814263	-1.988562

3-cis (in acetone):

Center Number	Atomic Number	Atomic Type	Coordinates (Angstroms) X	Y	Z
1	17	0	2.823695	3.010988	0.246515
2	8	0	-0.025738	2.401370	0.258801
3	1	0	-0.871444	2.282238	-0.240233
4	8	0	4.444087	-1.934013	-0.352150
5	8	0	2.685068	-3.210358	-0.494268
6	8	0	-2.055668	1.469804	-1.159967
7	8	0	-1.105144	-1.972820	1.714991
8	7	0	3.220118	-2.105452	-0.351904

3-trans (in acetone):

Center Number	Atomic Number	Atomic Type	Coordinates (Angstroms) X	Y	Z
1	17	0	-3.307546	-2.593860	0.506984
2	8	0	-0.418842	-2.558617	0.132764
3	1	0	0.359413	-2.617324	-0.475209
4	8	0	-4.004544	2.564152	-0.090049
5	8	0	-2.064711	3.466088	-0.498777
6	8	0	1.670390	1.540199	1.252360
7	8	0	1.550520	-2.075334	-1.565052
8	7	0	-2.780788	2.490621	-0.247133

9	7	0	-1.250792	-0.232872	0.173612
10	6	0	2.121748	1.417728	0.103015
11	6	0	2.951121	0.318569	-0.037851
12	1	0	4.026919	0.430069	-0.059753
13	6	0	2.362256	-0.940143	-0.174215
14	6	0	0.982485	-1.117919	-0.143845
15	1	0	0.563326	-2.109053	-0.239207
16	6	0	0.158083	-0.008908	0.031450
17	6	0	0.713571	1.291090	0.125601
18	6	0	-2.260465	0.469718	-0.474745
19	6	0	-3.615928	-0.170016	-0.194531
20	6	0	-3.286106	-1.229868	0.880043
21	1	0	-3.712402	-0.981400	1.857476
22	1	0	-3.631774	-2.234984	0.622301
23	6	0	-1.782416	-1.248984	1.016956
24	6	0	-4.126449	-0.812226	-1.505554
25	1	0	-4.253554	-0.052957	-2.281860
26	1	0	-5.095851	-1.283968	-1.319246
27	1	0	-3.438044	-1.578265	-1.876111
28	6	0	-4.607143	0.898212	0.303067
29	1	0	-4.249361	1.381181	1.217729
30	1	0	-5.570246	0.426103	0.518905
31	1	0	-4.761042	1.667142	-0.458313

9	7	0	1.271912	-0.220539	-0.215038
10	6	0	-2.335843	-1.172577	0.210455
11	6	0	-2.947872	0.066344	0.130255
12	1	0	-4.018408	0.169035	0.247756
13	6	0	-2.149358	1.182006	-0.130138
14	6	0	-0.769411	1.084105	-0.280792
15	1	0	-0.182570	1.971780	-0.467062
16	6	0	-0.159736	-0.163259	-0.167437
17	6	0	-0.939498	-1.326261	0.049629
18	6	0	2.099780	0.681767	0.512179
19	6	0	3.566948	0.367036	0.214050
20	6	0	3.485116	-0.754083	-0.846250
21	1	0	3.819210	-0.421881	-1.835621
22	1	0	4.066087	-1.644502	-0.591448
23	6	0	2.029872	-1.123781	-0.951116
24	6	0	4.236574	-0.121181	1.517695
25	1	0	4.186970	0.654069	2.286862
26	1	0	5.288353	-0.348952	1.320686
27	1	0	3.756463	-1.025707	1.904737
28	6	0	4.267465	1.635592	-0.309836
29	1	0	3.790215	2.012100	-1.220272
30	1	0	5.311730	1.404310	-0.541006
31	1	0	4.246219	2.425974	0.444904

3-TS-cis (in acetone):

Center Number	Atomic Number	Atomic Type	Coordinates (Angstroms) X	Y	Z
1	8	0	-1.026394	-1.883897	0.044625
2	8	0	-0.323165	2.685888	-0.216864
3	7	0	-0.217304	0.318087	-0.093512
4	6	0	1.930436	-1.107485	0.005979
5	6	0	3.354207	-1.094149	0.013256
6	6	0	4.107013	0.057563	-0.055548
7	1	0	5.187981	0.028969	-0.047519
8	6	0	3.409831	1.259872	-0.136461
9	6	0	2.020702	1.311425	-0.146205
10	1	0	1.550905	2.276177	-0.209297
11	6	0	1.241122	0.151921	-0.075453
12	6	0	-1.209586	-0.668836	-0.025248
13	6	0	-2.611843	-0.080772	-0.024554
14	6	0	-2.361486	1.410894	-0.242728
15	1	0	-2.699637	1.760272	-1.224209
16	1	0	-2.827364	2.055001	0.507810
17	6	0	-0.871164	1.605233	-0.183407
18	6	0	-3.441746	-0.725986	-1.151976
19	1	0	-2.986427	-0.554946	-2.132457
20	1	0	-4.442646	-0.284290	-1.157031
21	1	0	-3.537233	-1.802979	-0.994685
22	6	0	-3.254190	-0.369236	1.352460
23	1	0	-3.330377	-1.445764	1.524051
24	1	0	-4.260037	0.060331	1.372112
25	1	0	-2.675591	0.075310	2.168348
26	8	0	1.388152	-2.314054	0.075213
27	1	0	0.378304	-2.256474	0.066882
28	7	0	4.153844	2.505645	-0.213829
29	8	0	5.390894	2.446869	-0.205343
30	8	0	3.526202	3.569919	-0.284863
31	17	0	4.187020	-2.631564	0.114739

3-TS-trans (in acetone):

Center Number	Atomic Number	Atomic Type	Coordinates (Angstroms) X	Y	Z
1	8	0	-0.417578	2.580062	0.013374
2	8	0	-0.980587	-2.013120	0.094917
3	7	0	-0.235021	0.216757	-0.015494
4	6	0	1.953085	-1.144055	-0.043956
5	6	0	3.374878	-1.089451	-0.091984
6	6	0	4.091828	0.086017	-0.142398
7	1	0	5.172653	0.089118	-0.177883
8	6	0	3.358762	1.269640	-0.144996
9	6	0	1.969354	1.280770	-0.098163
10	1	0	1.471142	2.233210	-0.099422
11	6	0	1.226530	0.096703	-0.046364
12	6	0	-0.934830	1.486591	-0.044406
13	6	0	-2.436338	1.261095	-0.186403
14	6	0	-2.581450	-0.235196	0.088126
15	1	0	-2.990626	-0.442315	1.083526
16	1	0	-3.201335	-0.771359	-0.635116
17	6	0	-1.192710	-0.800674	0.050844
18	6	0	-3.202769	2.144578	0.811789
19	1	0	-2.918677	1.920521	1.844637
20	1	0	-4.276703	1.965020	0.704317
21	1	0	-3.007031	3.202279	0.619782
22	6	0	-2.829993	1.619133	-1.640153
23	1	0	-2.615085	2.670755	-1.847016
24	1	0	-3.902966	1.449961	-1.770940
25	1	0	-2.294799	1.003770	-2.370461
26	8	0	1.446416	-2.367333	-0.006455
27	1	0	0.435846	-2.340006	0.039885
28	7	0	4.063880	2.539041	-0.198362
29	8	0	5.301172	2.516677	-0.247190
30	8	0	3.404363	3.586273	-0.193423
31	17	0	4.252977	-2.604876	-0.089446

3-perpend (in chloroform):

Center Number	Atomic Number	Atomic Type	Coordinates (Angstroms) X	Y	Z
1	17	0	2.891401	2.905918	-0.285730
2	8	0	-0.043765	2.331400	0.023678
3	1	0	0.482629	3.144133	-0.065966
4	8	0	4.345757	-2.143523	-0.155980
5	8	0	2.551914	-3.354328	0.100650
6	8	0	-1.761176	-0.441062	-1.950453
7	8	0	-1.373533	0.288447	2.562308
8	7	0	3.124292	-2.267308	-0.022843
9	7	0	-1.261241	-0.093562	0.281372
10	6	0	2.154963	1.324418	-0.125304
11	6	0	2.938603	0.181856	-0.139302
12	1	0	4.013541	0.242809	-0.247773
13	6	0	2.308417	-1.056731	-0.009989
14	6	0	0.925413	-1.169999	0.133632
15	1	0	0.460318	-2.141967	0.233691
16	6	0	0.155412	-0.016627	0.145335
17	6	0	0.754401	1.252901	0.013553
18	6	0	-2.132241	-0.289780	-0.802735
19	6	0	-3.576202	-0.286678	-0.285129
20	6	0	-3.419155	-0.002288	1.228949
21	1	0	-3.884439	0.940594	1.533896
22	1	0	-3.842174	-0.788773	1.861391
23	6	0	-1.931917	0.091822	1.500239
24	6	0	-4.202476	-1.670009	-0.556866
25	1	0	-4.209595	-1.885514	-1.628691
26	1	0	-5.234652	-1.682907	-0.192699
27	1	0	-3.651103	-2.467115	-0.047372
28	6	0	-4.369291	0.816413	-1.013010
29	1	0	-3.933864	1.805529	-0.837532
30	1	0	-5.401387	0.827300	-0.648579
31	1	0	-4.383593	0.632139	-2.090532

3-cis (in chloroform):

Center Number	Atomic Number	Atomic Type	Coordinates (Angstroms) X	Y	Z
1	17	0	2.815863	3.014947	0.247649
2	8	0	-0.032965	2.397956	0.256528
3	1	0	-0.870000	2.282376	-0.256261
4	8	0	4.450897	-1.926781	-0.333037
5	8	0	2.694550	-3.203817	-0.512772
6	8	0	-2.058817	1.460575	-1.169011
7	8	0	-1.103061	-1.980521	1.708719
8	7	0	3.228543	-2.102018	-0.351251
9	7	0	-1.249777	-0.237939	0.169038
10	6	0	2.118450	1.420891	0.103200
11	6	0	2.950483	0.322739	-0.035685
12	1	0	4.026267	0.435840	-0.055518
13	6	0	2.365670	-0.937278	-0.173149
14	6	0	0.986920	-1.119109	-0.144224
15	1	0	0.572852	-2.112952	-0.234496
16	6	0	0.159515	-0.011403	0.028093
17	6	0	0.710738	1.290120	0.122886
18	6	0	-2.260209	0.464417	-0.478479
19	6	0	-3.616926	-0.171473	-0.190252
20	6	0	-3.284888	-1.234737	0.880042
21	1	0	-3.709091	-0.991193	1.859692
22	1	0	-3.630465	-2.239441	0.619997
23	6	0	-1.779691	-1.255110	1.014010
24	6	0	-4.139602	-0.807066	-1.499216
25	1	0	-4.263749	-0.045555	-2.273698
26	1	0	-5.111966	-1.272047	-1.310377
27	1	0	-3.458982	-1.577749	-1.875203
28	6	0	-4.599034	0.901056	0.316394
29	1	0	-4.233646	1.379034	1.230974
30	1	0	-5.564600	0.435346	0.536025
31	1	0	-4.751027	1.674359	-0.440816

3-trans (in chloroform):

Center Number	Atomic Number	Atomic Type	Coordinates (Angstroms)		
			X	Y	Z
1	17	0	-3.295957	-2.602152	0.510004
2	8	0	-0.407142	-2.555452	0.130564
3	1	0	0.359170	-2.615916	-0.490972
4	8	0	-4.013235	2.554829	-0.069877
5	8	0	-2.082006	3.459370	-0.517327
6	8	0	1.669334	1.554518	1.239245
7	8	0	1.555753	-2.064804	-1.576772
8	7	0	-2.792736	2.486343	-0.246189
9	7	0	1.271824	-0.212609	-0.222410
10	6	0	-2.330986	-1.177947	0.211341
11	6	0	-2.947462	0.059581	0.133054
12	1	0	-4.018101	0.159090	0.253304
13	6	0	-2.154520	1.178160	-0.129351
14	6	0	-0.774932	1.086512	-0.282783
15	1	0	-0.193468	1.978634	-0.465004
16	6	0	-0.160571	-0.159223	-0.172278
17	6	0	-0.934615	-1.325489	0.046596
18	6	0	2.098686	0.691783	0.506199
19	6	0	3.567195	0.368817	0.219131
20	6	0	3.487183	-0.746006	-0.847643
21	1	0	3.823723	-0.408903	-1.834644
22	1	0	4.067059	-1.638673	-0.597674
23	6	0	2.030779	-1.115246	-0.957930
24	6	0	4.218025	-0.132350	1.527440
25	1	0	4.163355	0.637898	2.301144
26	1	0	5.270822	-0.366663	1.342986
27	1	0	3.726896	-1.035865	1.903393
28	6	0	4.283047	1.634954	-0.288451
29	1	0	3.821117	2.019844	-1.203468
30	1	0	5.329423	1.399886	-0.506798
31	1	0	4.255053	2.422155	0.469239

3-TS-cis (in chloroform):

Center Number	Atomic Number	Atomic Type	Coordinates (Angstroms)		
			X	Y	Z
1	8	0	-1.030952	-1.891203	-0.012626
2	8	0	-0.332943	2.684522	-0.149489
3	7	0	-0.219263	0.313027	-0.106999
4	6	0	1.930352	-1.111069	-0.029045
5	6	0	3.353893	-1.096025	-0.014513
6	6	0	4.104112	0.059524	-0.050905
7	1	0	5.185207	0.033630	-0.039064
8	6	0	3.405995	1.262334	-0.104229
9	6	0	2.017587	1.312974	-0.117621
10	1	0	1.548412	2.279736	-0.155689
11	6	0	1.240442	0.149096	-0.080171
12	6	0	-1.211457	-0.675559	-0.061057
13	6	0	-2.612704	-0.082654	-0.042381
14	6	0	-2.359732	1.399135	-0.317044
15	1	0	-2.633565	1.688859	-1.338209
16	1	0	-2.876811	2.080023	0.363314
17	6	0	-0.874706	1.602222	-0.179261
18	6	0	-3.487388	-0.763985	-1.110431
19	1	0	-3.065784	-0.640242	-2.113050
20	1	0	-4.484383	-0.313181	-1.100528
21	1	0	-3.586917	-1.832525	-0.906097
22	6	0	-3.203636	-0.313964	1.369356
23	1	0	-3.273261	-1.382640	1.586907
24	1	0	-4.207951	0.117891	1.410930
25	1	0	-2.594602	0.161054	2.145213
26	8	0	1.390701	-2.321073	0.003967
27	1	0	0.383637	-2.268788	-0.001330
28	7	0	4.150834	2.513268	-0.147202
29	8	0	5.386627	2.452202	-0.133983
30	8	0	3.520276	3.575420	-0.195425
31	17	0	4.188407	-2.633067	0.050948

3-TS-trans (in chloroform):

Center Number	Atomic Number	Atomic Type	Coordinates (Angstroms)		
			X	Y	Z
1	8	0	-0.406316	2.515727	-0.666058
2	8	0	-0.986887	-2.004126	0.153764
3	7	0	-0.232574	0.200808	-0.180888
4	6	0	1.950337	-1.124169	0.175764
5	6	0	3.370861	-1.059628	0.243608
6	6	0	4.089210	0.104722	0.076786
7	1	0	5.169091	0.117606	0.134469
8	6	0	3.360790	1.264654	-0.170021
9	6	0	1.973598	1.265605	-0.250215
10	1	0	1.480742	2.200170	-0.449613
11	6	0	1.228868	0.091993	-0.084143
12	6	0	-0.927971	1.450571	-0.427114
13	6	0	-2.436587	1.249634	-0.316429
14	6	0	-2.576094	-0.271202	-0.281830
15	1	0	-3.228335	-0.649952	0.509420
16	1	0	-2.943869	-0.683387	-1.228699
17	6	0	-1.191641	-0.811263	-0.069286
18	6	0	-2.900908	1.898571	1.009316
19	1	0	-2.411446	1.443460	1.876646
20	1	0	-3.981409	1.762855	1.116510
21	1	0	-2.682011	2.969428	1.009404
22	6	0	-3.144828	1.903449	-1.514986
23	1	0	-2.956371	2.979432	-1.534002
24	1	0	-4.223868	1.740468	-1.433221
25	1	0	-2.804050	1.476254	-2.463575
26	8	0	1.442690	-2.332895	0.369358
27	1	0	0.436101	-2.320469	0.303738
28	7	0	4.071740	2.522608	-0.353166
29	8	0	5.306844	2.505595	-0.280496
30	8	0	3.414667	3.546726	-0.571413
31	17	0	4.244016	-2.543659	0.557538

(3–H)$^-$-perpend:

Center Number	Atomic Number	Atomic Type	Coordinates (Angstroms)		
			X	Y	Z
1	17	0	2.935691	2.926006	-0.223833
2	8	0	-0.004896	2.363309	0.074264
3	8	0	4.356821	-2.112057	-0.194088
4	8	0	2.559935	-3.340077	0.018294
5	8	0	-1.785848	-0.247165	-1.972981
6	8	0	-1.367262	0.072737	2.582473
7	7	0	3.114891	-2.220652	-0.072313
8	7	0	-1.263232	-0.118166	0.276910
9	6	0	2.160317	1.349184	-0.098551
10	6	0	2.934779	0.218004	-0.136183
11	1	0	4.010272	0.282549	-0.241354
12	6	0	2.319958	-1.050485	-0.035664
13	6	0	0.921764	-1.153852	0.102683
14	1	0	0.458028	-2.129644	0.180010
15	6	0	0.156935	-0.013427	0.139241
16	6	0	0.709845	1.331727	0.040826
17	6	0	-2.141581	-0.199718	-0.807049
18	6	0	-3.585044	-0.236529	-0.288553
19	6	0	-3.417179	-0.086770	1.241948
20	1	0	-3.872455	0.829650	1.630655
21	1	0	-3.842493	-0.923158	1.804964
22	6	0	-1.926586	-0.028462	1.502542
23	6	0	-4.217886	-1.589140	-0.676098
24	1	0	-4.238468	-1.707449	-1.763151
25	1	0	-5.246152	-1.633167	-0.303309
26	1	0	-3.662866	-2.429962	-0.246911
27	6	0	-4.380266	0.926981	-0.911408
28	1	0	-3.939349	1.895728	-0.655219
29	1	0	-5.409173	0.910065	-0.537961
30	1	0	-4.406032	0.835965	-2.000848

(3–H)$^-$-TS-cis:

Center Number	Atomic Number	Atomic Type	Coordinates (Angstroms)		
			X	Y	Z
1	8	0	-1.547031	-1.979390	0.863576
2	8	0	-0.642690	2.468651	0.622460
3	7	0	-0.620850	0.136133	0.294073
4	6	0	1.506352	-1.327002	0.205730
5	6	0	2.968641	-1.227775	0.107004
6	6	0	3.662208	-0.107617	-0.250296
7	1	0	4.739733	-0.109776	-0.350019
8	6	0	2.920399	1.063689	-0.505294
9	6	0	1.524129	1.084263	-0.329165
10	1	0	1.024541	2.019345	-0.511454
11	6	0	0.812034	-0.027536	0.090026
12	6	0	-1.664935	-0.850422	0.450458
13	6	0	-3.001335	-0.198607	0.078059
14	6	0	-2.727532	1.270325	0.415552
15	1	0	-3.133220	1.981901	-0.309773
16	1	0	-3.118490	1.546391	1.401190
17	6	0	-1.223162	1.406672	0.450608
18	6	0	-3.228540	-0.396441	-1.439949

(3–H)$^-$-TS-trans:

Center Number	Atomic Number	Atomic Type	Coordinates (Angstroms)		
			X	Y	Z
1	8	0	-0.861890	2.280369	-0.174855
2	8	0	-1.540965	-2.153706	0.585429
3	7	0	-0.592861	0.027534	0.447827
4	6	0	1.489210	-1.410521	-0.075889
5	6	0	2.868179	-1.302796	-0.571261
6	6	0	3.579711	-0.143185	-0.681010
7	1	0	4.611184	-0.134096	-1.007979
8	6	0	2.925696	1.056670	-0.335237
9	6	0	1.569508	1.054890	0.040875
10	1	0	1.125276	2.009285	0.261190
11	6	0	0.815947	-0.106102	0.100006
12	6	0	-1.281639	1.264092	0.356798
13	6	0	-2.674351	1.164399	0.983922
14	6	0	-2.687022	-0.260268	1.546724
15	1	0	-2.461397	-0.272194	2.620153
16	1	0	-3.623180	-0.799147	1.390355
17	6	0	-1.559265	-0.970081	0.833346
18	6	0	-2.834347	2.250155	2.063243

19	1	0	-2.419922	0.042521	-2.032889
20	1	0	-4.166349	0.087758	-1.731027
21	1	0	-3.299518	-1.460186	-1.686604
22	6	0	-4.158500	-0.805647	0.878599
23	1	0	-4.283917	-1.865415	0.641307
24	1	0	-5.089402	-0.285099	0.631788
25	1	0	-3.989293	-0.714158	1.956157
26	8	0	0.961408	-2.440675	0.338465
27	7	0	3.585165	2.234966	-0.925029
28	8	0	4.833992	2.213085	-1.063293
29	8	0	2.923382	3.276911	-1.155905
30	17	0	3.865144	-2.719274	0.398257

19	1	0	-2.067697	2.158084	2.839622
20	1	0	-3.815428	2.150150	2.538844
21	1	0	-2.763537	3.248490	1.623038
22	6	0	-3.732614	1.348958	-0.125384
23	1	0	-3.613820	2.322551	-0.608481
24	1	0	-4.734540	1.297463	0.311968
25	1	0	-3.654209	0.571048	-0.892318
26	8	0	1.003667	-2.528122	0.187663
27	7	0	3.631517	2.277059	-0.384883
28	8	0	4.839048	2.271387	-0.732900
29	8	0	3.047677	3.346156	-0.079822
30	17	0	3.670598	-2.824740	-0.962580

$(3\text{–H})^{-}\cdot Li^{+}$-perpend:

Center Number	Atomic Number	Atomic Type	Coordinates (Angstroms) X	Y	Z
1	17	0	2.907646	2.862487	-0.202743
2	8	0	-0.027220	2.271106	0.098218
3	8	0	4.372322	-2.163983	-0.212182
4	8	0	2.587699	-3.404587	-0.008336
5	8	0	-1.778053	-0.272370	-1.974660
6	8	0	-1.360675	-0.080432	2.587406
7	7	0	3.137550	-2.288355	-0.090113
8	7	0	-1.258264	-0.208237	0.277945
9	6	0	2.147510	1.279563	-0.087169
10	6	0	2.934242	0.152267	-0.134080
11	1	0	4.008820	0.227245	-0.240176
12	6	0	2.326935	-1.114341	-0.042084
13	6	0	0.934601	-1.233182	0.096013
14	1	0	0.476620	-2.211994	0.165303
15	6	0	0.160474	-0.094651	0.141900
16	6	0	0.710282	1.238510	0.053448
17	6	0	-2.136181	-0.264659	-0.809022
18	6	0	-3.578653	-0.326236	-0.291146
19	6	0	-3.410740	-0.236725	1.244536
20	1	0	-3.884141	0.652057	1.673371
21	1	0	-3.817901	-1.105203	1.771377
22	6	0	-1.921341	-0.162273	1.507079
23	6	0	-4.210220	-1.662381	-0.733530
24	1	0	-4.232540	-1.735118	-1.824483
25	1	0	-5.237705	-1.723150	-0.361200
26	1	0	-3.653373	-2.519572	-0.340740
27	6	0	-4.375150	0.861176	-0.866371
28	1	0	-3.935658	1.819292	-0.570653
29	1	0	-5.404015	0.827698	-0.494200
30	1	0	-4.400621	0.814721	-1.958605
31	3	0	-0.115868	4.115869	-0.029731

$(3\text{–H})^{-}\cdot Li^{+}$-cis:

Center Number	Atomic Number	Atomic Type	Coordinates (Angstroms) X	Y	Z
1	17	0	2.814436	2.988727	0.304243
2	8	0	-0.084996	2.269508	0.397931
3	8	0	4.482200	-1.916856	-0.458001
4	8	0	2.742642	-3.233314	-0.524352
5	8	0	-1.964556	1.081315	-1.493990
6	8	0	-1.139982	-1.726672	2.041119
7	7	0	3.250922	-2.101946	-0.400998
8	7	0	-1.228065	-0.296621	0.212856
9	6	0	2.120671	1.380987	0.144699
10	6	0	2.954700	0.305365	-0.051589
11	1	0	4.027941	0.433907	-0.107757
12	6	0	2.393736	-0.978481	-0.187715
13	6	0	1.006194	-1.168035	-0.120290
14	1	0	0.592616	-2.162477	-0.225355
15	6	0	0.182297	-0.079477	0.084570
16	6	0	0.682126	1.268456	0.225527
17	6	0	-2.198211	0.279182	-0.592623
18	6	0	-3.581708	-0.245216	-0.204521
19	6	0	-3.292405	-1.115582	1.038956
20	1	0	-3.735364	-0.706088	1.952568
21	1	0	-3.647827	-2.145519	0.940993
22	6	0	-1.790526	-1.127263	1.207118
23	6	0	-4.133671	-1.080541	-1.381054
24	1	0	-4.239272	-0.462437	-2.276735
25	1	0	-5.118611	-1.474911	-1.113240
26	1	0	-3.479161	-1.925924	-1.616710
27	6	0	-4.520239	0.937982	0.099959
28	1	0	-4.138670	1.552691	0.921542
29	1	0	-5.505261	0.557221	0.386762
30	1	0	-4.638805	1.572362	-0.782546
31	3	0	-1.114417	2.895560	-1.071511

$(3\text{–H})^{-}\cdot Li^{+}$-trans:

Center Number	Atomic Number	Atomic Type	Coordinates (Angstroms) X	Y	Z
1	17	0	-3.219491	2.621665	-0.624086
2	8	0	-0.259585	2.421217	-0.273928
3	8	0	-4.125689	-2.476794	0.160326
4	8	0	-2.216160	-3.465900	0.534480
5	8	0	1.690919	-1.409640	-1.504532
6	8	0	1.492800	1.652984	1.907497
7	7	0	-2.885638	-2.444869	0.282918
8	7	0	1.263057	0.100862	0.204458
9	6	0	-2.294572	1.165322	-0.284891
10	6	0	-2.954069	-0.033722	-0.149701
11	1	0	-4.030167	-0.093769	-0.249429
12	6	0	-2.209640	-1.195129	0.130843
13	6	0	-0.815412	-1.140245	0.266971
14	1	0	-0.260044	-2.043820	0.481369
15	6	0	-0.166507	0.069619	0.121908
16	6	0	-0.861551	1.305417	-0.158733
17	6	0	2.102581	-0.667464	-0.634940
18	6	0	3.565037	-0.412513	-0.258143
19	6	0	3.462059	0.645424	0.863430
20	1	0	3.908518	0.320531	1.808252
21	1	0	3.932190	1.597540	0.596936
22	6	0	1.988598	0.886309	1.083522
23	6	0	4.324490	0.108053	-1.494645
24	1	0	4.299837	-0.631601	-2.299402
25	1	0	5.369263	0.296686	-1.229070
26	1	0	3.892756	1.041774	-1.869205
27	6	0	4.188986	-1.733583	0.239190
28	1	0	3.661794	-2.124415	1.115595
29	1	0	5.232954	-1.559729	0.517472
30	1	0	4.161708	-2.491737	-0.548192
31	3	0	0.427441	3.256136	1.311294

$(3\text{–H})^{-}\cdot Li^{+}$-TS-cis:

Center Number	Atomic Number	Atomic Type	Coordinates (Angstroms) X	Y	Z
1	8	0	-1.548948	-1.975519	0.816157
2	8	0	-0.674016	2.493440	0.465418
3	7	0	-0.625048	0.137693	0.260892
4	6	0	1.501795	-1.330684	0.134739
5	6	0	2.947298	-1.268954	-0.027171
6	6	0	3.664436	-0.136237	-0.303063
7	1	0	4.738308	-0.157306	-0.432537
8	6	0	2.945501	1.062291	-0.422656
9	6	0	1.559151	1.100162	-0.231387
10	1	0	1.079544	2.057148	-0.325974
11	6	0	0.819825	-0.033902	0.087645
12	6	0	-1.656370	-0.827467	0.422927
13	6	0	-3.008352	-0.209715	0.069347
14	6	0	-2.744403	1.279075	0.295045
15	1	0	-3.141583	1.932203	-0.487271
16	1	0	-3.145666	1.631147	1.251511
17	6	0	-1.245027	1.425593	0.339955
18	6	0	-3.286900	-0.518178	-1.423128
19	1	0	-2.508290	-0.109237	-2.074733
20	1	0	-4.242312	-0.066599	-1.707581
21	1	0	-3.350907	-1.597105	-1.591542
22	6	0	-4.127464	-0.774234	0.953521
23	1	0	-4.248541	-1.848368	0.793347
24	1	0	-5.071435	-0.279555	0.704997
25	1	0	-3.920868	-0.605023	2.014845
26	8	0	0.957829	-2.461520	0.271321
27	7	0	3.636637	2.268675	-0.738876
28	8	0	4.874547	2.225936	-0.901933
29	8	0	2.993475	3.333370	-0.846548
30	17	0	3.813519	-2.796693	0.102086
31	3	0	-0.361683	-3.455407	1.078710

$(3\text{–H})^{-}\cdot Li^{+}$-TS-trans:

Center Number	Atomic Number	Atomic Type	Coordinates (Angstroms) X	Y	Z
1	8	0	-0.877584	2.272027	-0.106395
2	8	0	-1.491584	-2.194052	0.705485
3	7	0	-0.600742	-0.003398	0.472057

$(3\text{–H})^{-}\cdot Na^{+}$-perpend:

Center Number	Atomic Number	Atomic Type	Coordinates (Angstroms) X	Y	Z
1	17	0	2.911578	2.628965	-0.210421
2	8	0	-0.015308	2.049137	0.094347
3	8	0	4.363983	-2.405271	-0.207076

Center Number	Atomic Number	Atomic Type	X	Y	Z
4	6	0	1.538227	-1.415767	0.086473
5	6	0	2.938542	-1.320198	-0.302149
6	6	0	3.618061	-0.155758	-0.536719
7	1	0	4.665997	-0.149357	-0.805486
8	6	0	2.897856	1.040402	-0.404522
9	6	0	1.536498	1.036885	-0.078744
10	1	0	1.051064	1.992677	-0.008336
11	6	0	0.819942	-0.137219	0.128335
12	6	0	-1.297505	1.251520	0.404911
13	6	0	-2.684808	1.148645	1.034179
14	6	0	-2.752427	-0.315127	1.466113
15	1	0	-2.670213	-0.434894	2.553186
16	1	0	-3.655820	-0.837602	1.144836
17	6	0	-1.549013	-0.984404	0.853838
18	6	0	-2.776285	2.135791	2.214996
19	1	0	-2.013398	1.927315	2.971957
20	1	0	-3.759670	2.045022	2.687062
21	1	0	-2.650948	3.165078	1.868784
22	6	0	-3.745438	1.489041	-0.033971
23	1	0	-3.593619	2.502403	-0.414412
24	1	0	-4.742731	1.429716	0.412376
25	1	0	-3.705926	0.792478	-0.877788
26	8	0	1.072370	-2.552793	0.372802
27	7	0	3.559152	2.286636	-0.611341
28	8	0	4.772528	2.279972	-0.908889
29	8	0	2.915677	3.349639	-0.490207
30	17	0	3.817243	-2.840850	-0.431494
31	3	0	-0.355505	-3.699752	0.378894

Center Number	Atomic Number	Atomic Type	X	Y	Z
4	8	0	2.574191	-3.639804	0.000716
5	8	0	-1.780758	-0.515022	-1.973874
6	8	0	-1.365790	-0.280449	2.586508
7	7	0	3.126824	-2.523209	-0.084493
8	7	0	-1.261571	-0.430875	0.278391
9	6	0	2.151056	1.044779	-0.090684
10	6	0	2.934136	-0.083003	-0.135424
11	1	0	4.008981	-0.012263	-0.242338
12	6	0	2.322704	-1.349605	-0.040115
13	6	0	0.928189	-1.461840	0.098701
14	1	0	0.467646	-2.439390	0.170427
15	6	0	0.157741	-0.322292	0.142331
16	6	0	0.709930	1.014162	0.051818
17	6	0	-2.138967	-0.494279	-0.808248
18	6	0	-3.582200	-0.545637	-0.291096
19	6	0	-3.415194	-0.437259	1.243309
20	1	0	-3.882720	0.460615	1.659580
21	1	0	-3.828976	-1.295708	1.781268
22	6	0	-1.925465	-0.369162	1.505999
23	6	0	-4.216182	-1.886121	-0.716650
24	1	0	-4.237155	-1.973325	-1.806586
25	1	0	-5.244339	-1.939677	-0.344995
26	1	0	-3.661829	-2.739259	-0.311696
27	6	0	-4.375714	0.635777	-0.882320
28	1	0	-3.934133	1.596713	-0.599094
29	1	0	-5.404888	0.609690	-0.510362
30	1	0	-4.400666	0.574900	-1.973865
31	11	0	0.004886	4.272913	-0.006649

(3–H)$^-$·Na$^+$-cis:

Center Number	Atomic Number	Atomic Type	Coordinates (Angstroms) X	Y	Z
1	17	0	2.714820	2.927603	0.572704
2	8	0	-0.140854	2.028447	0.672298
3	8	0	4.639124	-1.797524	-0.609213
4	8	0	2.975509	-3.205728	-0.748314
5	8	0	-1.894317	0.755200	-1.483097
6	8	0	-1.017066	-1.957855	2.109820
7	7	0	3.419629	-2.056734	-0.544622
8	7	0	-1.141336	-0.557378	0.263712
9	6	0	2.108517	1.299345	0.296123
10	6	0	2.995847	0.293373	-0.000875
11	1	0	4.058820	0.487683	-0.065058
12	6	0	2.507569	-1.009124	-0.229255
13	6	0	1.131978	-1.281403	-0.155219
14	1	0	0.770570	-2.285635	-0.336614
15	6	0	0.254470	-0.263581	0.149676
16	6	0	0.676411	1.100158	0.399547
17	6	0	-2.121828	-0.018897	-0.560063
18	6	0	-3.497552	-0.549095	-0.146845
19	6	0	-3.179932	-1.444282	1.073195
20	1	0	-3.656231	-1.094337	1.994267
21	1	0	-3.480125	-2.486935	0.930518
22	6	0	-1.679876	-1.392046	1.259937
23	6	0	-4.094438	-1.351544	-1.321968
24	1	0	-4.224863	-0.711762	-2.199090
25	1	0	-5.073041	-1.746849	-1.032645
26	1	0	-3.453518	-2.194617	-1.599668
27	6	0	-4.414974	0.636266	0.213959
28	1	0	-4.004267	1.225418	1.040170
29	1	0	-5.396068	0.258639	0.518130
30	1	0	-4.550572	1.294213	-0.648856
31	11	0	-1.246091	3.008034	-1.070180

(3–H)$^-$·Na$^+$-trans:

Center Number	Atomic Number	Atomic Type	Coordinates (Angstroms) X	Y	Z
1	17	0	-3.102991	2.516685	-0.911027
2	8	0	-0.147444	2.160293	-0.577900
3	8	0	-4.299376	-2.431484	0.315848
4	8	0	-2.448393	-3.497469	0.771427
5	8	0	1.626645	-1.691933	-1.545200
6	8	0	1.475585	1.380646	1.857236
7	7	0	-3.056884	-2.460463	0.434842
8	7	0	1.229193	-0.167329	0.155731
9	6	0	-2.260795	1.044387	-0.442990
10	6	0	-2.987081	-0.096111	-0.199786
11	1	0	-4.065536	-0.101439	-0.292093
12	6	0	-2.311622	-1.273981	0.178960
13	6	0	-0.913708	-1.287090	0.310944
14	1	0	-0.409259	-2.197973	0.606957
15	6	0	-0.197804	-0.136682	0.059839
16	6	0	-0.816200	1.111507	-0.342410
17	6	0	2.050225	-0.951316	-0.677829
18	6	0	3.518822	-0.717086	-0.306394
19	6	0	3.436193	0.338575	0.820192
20	1	0	3.873002	-0.002370	1.763904
21	1	0	3.929193	1.280547	0.559361
22	6	0	1.965806	0.609840	1.039061
23	6	0	4.275954	-0.197607	-1.545063
24	1	0	4.238432	-0.931727	-2.354447
25	1	0	5.324446	-0.021376	-1.285269
26	1	0	3.851652	0.742968	-1.911034
27	6	0	4.131946	-2.046697	0.179454
28	1	0	3.605907	-2.436550	1.057060
29	1	0	5.179701	-1.886585	0.452028
30	1	0	4.091343	-2.800529	-0.611606
31	11	0	0.629022	3.474462	1.123040

(3–H)$^-$·Na$^+$-TS-cis:

Center Number	Atomic Number	Atomic Type	Coordinates (Angstroms) X	Y	Z
1	8	0	-1.550108	-1.979490	0.856593
2	8	0	-0.665341	2.479176	0.529300
3	7	0	-0.624602	0.131390	0.282876
4	6	0	1.501060	-1.334209	0.169703
5	6	0	2.952509	-1.263335	0.024126
6	6	0	3.662260	-0.135729	-0.282483
7	1	0	4.737119	-0.152412	-0.404928
8	6	0	2.936011	1.055631	-0.449958
9	6	0	1.546371	1.090043	-0.261492
10	1	0	1.061044	2.040739	-0.388547
11	6	0	0.816068	-0.035583	0.097197
12	6	0	-1.661029	-0.842237	0.446832
13	6	0	-3.006182	-0.213561	0.077266
14	6	0	-2.739975	1.269928	0.340149
15	1	0	-3.137507	1.942432	-0.425616
16	1	0	-3.141136	1.597558	1.305421
17	6	0	-1.238980	1.412713	0.386397
18	6	0	-3.259218	-0.486488	-1.425935
19	1	0	-2.466405	-0.068280	-2.054099
20	1	0	-4.206707	-0.023575	-1.719158
21	1	0	-3.325872	-1.561125	-1.620054
22	6	0	-4.143758	-0.790838	0.927860
23	1	0	-4.267052	-1.860863	0.742490
24	1	0	-5.081410	-0.286728	0.673756
25	1	0	-3.955552	-0.646279	1.996291
26	8	0	0.952020	-2.454304	0.310048

(3–H)$^-$·Na$^+$-TS-trans:

Center Number	Atomic Number	Atomic Type	Coordinates (Angstroms) X	Y	Z
1	8	0	-0.895828	2.232282	-0.194620
2	8	0	-1.509793	-2.198132	0.679436
3	7	0	-0.601367	-0.011133	0.469186
4	6	0	1.525921	-1.424936	0.055313
5	6	0	2.919189	-1.323696	-0.372067
6	6	0	3.603882	-0.156957	-0.568974
7	1	0	4.646079	-0.147307	-0.859391
8	6	0	2.901556	1.042650	-0.363142
9	6	0	1.541539	1.036277	-0.020493
10	1	0	1.067659	1.993241	0.101661
11	6	0	0.816720	-0.138715	0.132377
12	6	0	-1.303333	1.229772	0.364357
13	6	0	-2.680400	1.141476	1.020919
14	6	0	-2.728357	-0.307132	1.511036
15	1	0	-2.589851	-0.381755	2.596534
16	1	0	-3.647912	-0.837482	1.256161
17	6	0	-1.553905	-0.996887	0.861368
18	6	0	-2.760804	2.173330	2.163503
19	1	0	-1.980410	2.004389	2.912685
20	1	0	-3.733271	2.090693	2.659352
21	1	0	-2.654980	3.189763	1.774969
22	6	0	-3.762973	1.430425	-0.040030
23	1	0	-3.624844	2.428044	-0.464850
24	1	0	-4.751968	1.384000	0.425997
25	1	0	-3.734234	0.700166	-0.855409
26	8	0	1.055572	-2.551426	0.347548

27	7	0	3.618799	2.248812	-0.806818
28	8	0	4.860576	2.211093	-0.961250
29	8	0	2.970250	3.307463	-0.960956
30	17	0	3.826622	-2.783005	0.205279
31	11	0	-0.431301	-3.958982	1.133982

27	7	0	3.573924	2.284406	-0.516193
28	8	0	4.784162	2.280669	-0.836252
29	8	0	2.948316	3.351250	-0.328035
30	17	0	3.778801	-2.847612	-0.583875
31	11	0	-0.427379	-4.176915	0.365394

$(3–H)^{-}·K^{+}$-perpend:

Center	Atomic	Atomic	Coordinates (Angstroms)		
Number	Number	Type	X	Y	Z
1	17	0	-2.894297	-2.421190	-0.144642
2	8	0	0.025958	-1.807332	0.127263
3	8	0	-4.398247	2.597093	-0.234718
4	8	0	-2.620758	3.854540	-0.053760
5	8	0	1.772539	0.761150	-1.969767
6	8	0	1.350534	0.562984	2.591770
7	7	0	-3.161153	2.729419	-0.116400
8	7	0	1.249049	0.692850	0.282170
9	6	0	-2.149234	-0.826245	-0.058999
10	6	0	-2.944044	0.291408	-0.121578
11	1	0	-4.018486	0.207694	-0.223478
12	6	0	-2.345930	1.567123	-0.051105
13	6	0	-0.951656	1.695821	0.084296
14	1	0	-0.501933	2.679515	0.139509
15	6	0	-0.168937	0.566265	0.145853
16	6	0	-0.705437	-0.780297	0.075358
17	6	0	2.128051	0.754509	-0.803049
18	6	0	3.569776	0.824894	-0.283589
19	6	0	3.400847	0.726312	1.251196
20	1	0	3.875063	-0.164637	1.674747
21	1	0	3.806345	1.592010	1.783805
22	6	0	1.911207	0.647242	1.511348
23	6	0	4.190338	2.168909	-0.717831
24	1	0	4.212618	2.248287	-1.808341
25	1	0	5.217100	2.236366	-0.344544
26	1	0	3.626045	3.019063	-0.320331
27	6	0	4.377197	-0.352303	-0.864122
28	1	0	3.945262	-1.315699	-0.574367
29	1	0	5.405231	-0.312320	-0.490205
30	1	0	4.403900	-0.299621	-1.956056
31	19	0	0.089650	-4.428191	-0.067116

$(3–H)^{-}·K^{+}$-cis:

Center	Atomic	Atomic	Coordinates (Angstroms)		
Number	Number	Type	X	Y	Z
1	17	0	2.555651	2.916505	0.817663
2	8	0	-0.210095	1.754523	0.895095
3	8	0	4.858979	-1.511348	-0.768688
4	8	0	3.324640	-3.052746	-0.978192
5	8	0	-1.840139	0.399623	-1.447796
6	8	0	-0.754340	-2.251775	2.133552
7	7	0	3.668060	-1.883974	-0.698323
8	7	0	-0.988710	-0.863016	0.290629
9	6	0	2.086288	1.266643	0.420025
10	6	0	3.050875	0.370414	0.030277
11	1	0	4.090812	0.663699	-0.036436
12	6	0	2.676387	-0.951765	-0.289226
13	6	0	1.330826	-1.351543	-0.212497
14	1	0	1.055508	-2.367865	-0.464843
15	6	0	0.374838	-0.443791	0.183410
16	6	0	0.677463	0.932833	0.533581
17	6	0	-2.009784	-0.390413	-0.528519
18	6	0	-3.343219	-1.014288	-0.103969
19	6	0	-2.950668	-1.907675	1.095844
20	1	0	-3.453910	-1.622766	2.024788
21	1	0	-3.162400	-2.967981	0.925909
22	6	0	-1.458856	-1.736710	1.283571
23	6	0	-3.913798	-1.830486	-1.281618
24	1	0	-4.094679	-1.185694	-2.146090
25	1	0	-4.863569	-2.285728	-0.984062
26	1	0	-3.230458	-2.631046	-1.583178
27	6	0	-4.322304	0.108214	0.295520
28	1	0	-3.929327	0.708304	1.122644
29	1	0	-5.272507	-0.331745	0.613844
30	1	0	-4.515880	0.770519	-0.553102
31	19	0	-1.633987	3.151782	-0.905055

$(3–H)^{-}·K^{+}$-trans:

Center	Atomic	Atomic	Coordinates (Angstroms)		
Number	Number	Type	X	Y	Z
1	17	0	2.996445	2.369001	1.215934
2	8	0	0.063048	1.884355	0.827814
3	8	0	4.467221	-2.355912	-0.494613
4	8	0	2.677183	-3.478955	-1.050225
5	8	0	-1.503075	-1.997019	1.566800
6	8	0	-1.503367	1.118846	-1.800169
7	7	0	3.226256	-2.444249	-0.614223
8	7	0	-1.182404	-0.440285	-0.119240
9	6	0	2.236215	0.905600	0.599814
10	6	0	3.024568	-0.162310	0.248960
11	1	0	4.101866	-0.115389	0.343579
12	6	0	2.416970	-1.335979	-0.244402
13	6	0	1.019869	-1.415239	-0.377375
14	1	0	0.565963	-2.320949	-0.759436
15	6	0	0.241062	-0.338125	-0.018821
16	6	0	0.787315	0.903302	0.500985
17	6	0	-1.962046	-1.268152	0.706072
18	6	0	-3.440960	-1.102279	0.337763
19	6	0	-3.411784	-0.029009	-0.775081
20	1	0	-3.834089	-0.379108	-1.722019
21	1	0	-3.949876	0.884005	-0.500580
22	6	0	-1.955748	0.316800	-0.993195
23	6	0	-4.223077	-0.638215	1.582401
24	1	0	-4.150413	-1.380705	2.381796
25	1	0	-5.278842	-0.509825	1.323935
26	1	0	-3.844886	0.316648	1.961838
27	6	0	-3.985942	-2.454657	-0.166145
28	1	0	-3.438709	-2.806383	-1.046967
29	1	0	-5.040024	-2.344795	-0.439616
30	1	0	-3.908760	-3.215300	0.615645
31	19	0	-0.766079	3.691031	-0.973553

$(3–H)^{-}·K^{+}$-TS-cis:

Center	Atomic	Atomic	Coordinates (Angstroms)		
Number	Number	Type	X	Y	Z
1	8	0	-1.545226	-1.960912	0.878762
2	8	0	-0.661756	2.491818	0.551304
3	7	0	-0.622398	0.147019	0.291947
4	6	0	1.503758	-1.319434	0.189533
5	6	0	2.957318	-1.241994	0.043546
6	6	0	3.661409	-0.117142	-0.283484
7	1	0	4.736216	-0.131119	-0.407283
8	6	0	2.930757	1.069881	-0.470389
9	6	0	1.540152	1.100926	-0.279468
10	1	0	1.050306	2.047485	-0.421098
11	6	0	0.816008	-0.020367	0.100461
12	6	0	-1.660362	-0.830442	0.457330
13	6	0	-3.002235	-0.199664	0.076829
14	6	0	-2.737175	1.281317	0.358542
15	1	0	-3.137330	1.963019	-0.397703
16	1	0	-3.137539	1.595063	1.328848
17	6	0	-1.235215	1.424810	0.403680
18	6	0	-3.239093	-0.455307	-1.431724
19	1	0	-2.439533	-0.030441	-2.046695
20	1	0	-4.183430	0.010534	-1.730709
21	1	0	-3.303320	-1.527763	-1.638863
22	6	0	-4.149009	-0.785837	0.908229
23	1	0	-4.270352	-1.853754	0.709043
24	1	0	-5.084288	-0.279151	0.650312
25	1	0	-3.972137	-0.654493	1.980337
26	8	0	0.956528	-2.436230	0.344988
27	7	0	3.606876	2.257235	-0.848520
28	8	0	4.849623	2.223056	-1.006024
29	8	0	2.954072	3.312171	-1.019272
30	17	0	3.838828	-2.755176	0.248970
31	19	0	-0.482881	-4.415153	1.202843

(3–H)$^{-}$·K^{+}-TS-trans:

Center Number	Atomic Number	Atomic Type	Coordinates (Angstroms) X	Y	Z
1	8	0	-0.875145	2.275456	-0.138519
2	8	0	-1.522708	-2.163263	0.654315
3	7	0	-0.593574	0.019348	0.481526
4	6	0	1.510605	-1.409108	0.012395
5	6	0	2.899508	-1.310121	-0.437006
6	6	0	3.596849	-0.146630	-0.603028
7	1	0	4.635435	-0.140617	-0.906352
8	6	0	2.914030	1.056241	-0.348669
9	6	0	1.556387	1.055543	0.008780
10	1	0	1.095084	2.013618	0.166763
11	6	0	0.818918	-0.113878	0.131288
12	6	0	-1.288641	1.261064	0.396687
13	6	0	-2.675241	1.160194	1.033853
14	6	0	-2.694101	-0.273116	1.570177
15	1	0	-2.492484	-0.307312	2.647988
16	1	0	-3.623853	-0.813581	1.383896
17	6	0	-1.550253	-0.970235	0.874644
18	6	0	-2.814179	2.227459	2.135201
19	1	0	-2.044503	2.110838	2.905191
20	1	0	-3.793777	2.130505	2.614279
21	1	0	-2.733159	3.232739	1.713192
22	6	0	-3.741792	1.375537	-0.061860
23	1	0	-3.621703	2.359123	-0.523526
24	1	0	-4.739642	1.319352	0.383849
25	1	0	-3.674359	0.614743	-0.846707
26	8	0	1.027821	-2.533103	0.283864
27	7	0	3.600842	2.290839	-0.466833
28	8	0	4.809800	2.282961	-0.796696
29	8	0	2.991560	3.360904	-0.239111
30	17	0	3.736275	-2.837716	-0.711258
31	19	0	-0.492292	-4.635037	0.376142

Supplementary References

1 Dial, B. E., Pellechia, P. J., Smith, M. D. & Shimizu, K. D. Proton grease: an acid accelerated molecular rotor. *J. Am. Chem. Soc.* **134**, 3675−3678 (2012).

2 Amman, C.; Meier, P. & Merbach, A. E. A simple multinuclear NMR thermometer. *J. Magn. Reson.* **46**, 319–321 (1982).

3 Sheldrick, G. M. A short history of SHELX. *Acta Crystallogr. A.* **64**, 112–122 (2008).

4 Rohonczy, J. *DNMR line shape analysis*, Software Manual, version 1.1 (Bruker BioSpin GmbH: Rheinstetten, 2007).

5 Sandstrom, J. *Dynamic NMR spectroscopy* (Academic Press: London, 1982).

6 Frisch, M. J., Trucks, G. W., Schlegel, H. B., Scuseria, G. E., Robb, M. A., Cheeseman, J., Scalmani, G., Barone, V., Mennucci, B., Petersson, G. A., Nakatsuji, H., Caricato, M., Li, X., Hratchian, H. P., Izmaylov, A. F., Bloino, J., Zheng, G., Sonnenberg, J. L., Hada, M., Ehara, M., Toyota, K., Fukuda, R., Hasegawa, J., Ishida, M., Nakajima, T., Honda, Y., Kitao, O., Nakai, H., Vreven, T., Montgomery, J. A., Peralta, Jr., J. E., Ogliaro, F., Bearpark, M., Heyd, J. J., Brothers, E., Kudin, K. N., Staroverov, V. N., Keith, T., Kobayashi, R., Normand, J., Raghavachari, K., Rendell, A., Burant, J. C., Iyengar, S. S., Tomasi, J., Cossi, M., Rega, N., Millam, J. M., Klene, M., Knox, J. E., Cross, J. B., Bakken, V., Adamo, C., Jaramillo, J., Gomperts, R., Stratmann, R. E., Yazyev, O., Austin, A. J., Cammi, R., Pomelli, C., Ochterski, J. W., Martin, R. L., Morokuma, K., Zakrzewski, V. G., Voth, G. A., Salvador, P., Dannenberg, J. J., Dapprich, S., Daniels, A. D., Farkas, O., Foresman, J. B., Ortiz, J. V., Cioslowski, J. & Fox, D. J. *Gaussian 09, Revision D.01*; Gaussian, Inc., Wallingford CT, 2013.

7 Wohlfarth, C. Permittivity (dielectric constant) of liquids in CRC handbook of chemistry and physics (Ed.: D. R. Lide), CRC Press, Boca Raton 2005, online version, http://www.hbcpnetbase.com.
